# Construction of Potential Glioblastoma Multiforme-Related miRNA-mRNA Regulatory Network

**DOI:** 10.3389/fnmol.2019.00066

**Published:** 2019-03-26

**Authors:** Weiyang Lou, Bisha Ding, Liang Xu, Weimin Fan

**Affiliations:** ^1^Program of Innovative Cancer Therapeutics, Division of Hepatobiliary and Pancreatic Surgery, Department of Surgery, Key Laboratory of Organ Transplantation, First Affiliated Hospital, College of Medicine, Zhejiang University, Hangzhou, China; ^2^Key Laboratory of Organ Transplantation, Hangzhou, China; ^3^Key Laboratory of Combined Multi-organ Transplantation, Ministry of Public Health, Hangzhou, China; ^4^Department of Pathology and Laboratory Medicine, Medical University of South Carolina, Charleston, SC, United States

**Keywords:** glioblastoma multiform (GBM), microRNAs (miRNAs), GEO, TCGA, bioinformatic analysis

## Abstract

**Background:** Glioblastoma multiforme (GBM), the most common and aggressive human malignant brain tumor, is notorious for its limited treatment options and poor prognosis. MicroRNAs (miRNAs) are found to be involved in tumorigenesis of GBM. However, a comprehensive miRNA-mRNA regulatory network has still not been established.

**Methods:** A miRNA microarray dataset (GSE90603) was obtained from GEO database. Then, we employed GEO2R tool to perform differential expression analysis. Potential transcription factors and target genes of screened differentially expressed miRNAs (DE-miRNAs) were predicted. The GBM mRNA dataset were downloaded from TCGA database for identifying differentially expressed genes (DEGs). Next, GO annotation and KEGG pathway enrichment analysis was conducted. PPI network was then established, and hub genes were identified via Cytoscape software. The expression and prognostic roles of hub genes was further evaluated.

**Results:** Total 33 DE-miRNAs, consisting of 10 upregulated DE-miRNAs and 23 downregulated DE-miRNAs, were screened. SP1 was predicted to potentially regulate most of screened DE-miRNAs. Three thousand and twenty seven and 3,879 predicted target genes were obtained for upregulated and downregulated DE-miRNAs, respectively. Subsequently, 1,715 upregulated DEGs and 1,259 downregulated DEGs were identified. Then, 149 and 295 potential downregulated and upregulated genes commonly appeared in target genes of DE-miRNAs and DEGs were selected for GO annotation and KEGG pathway enrichment analysis. The downregulated genes were significantly enriched in cGMP-PKG signaling pathway and calcium signaling pathway whereas the upregulated genes were enriched in pathways in cancer and PI3K-Akt signaling pathway. Construction and analysis of PPI network showed that STXBP1 and TP53 were recognized as hub genes with the highest connectivity degrees. Expression analytic result of the top 20 hub genes in GBM using GEPIA database was generally identical with previous differential expression analysis for TCGA data. EGFR, PPP3CB, and MYO5A expression was significantly associated with patients' OS.

**Conclusions:** In this study, we established a potential GBM-related miRNA-mRNA regulatory network, which explores a comprehensive understanding of the molecular mechanisms and provides key clues in seeking novel therapeutic targets for GBM. In the future, more experiments need to be performed to validate our current findings.

## Introduction

Glioblastoma multiforme (GBM), a highly prevalent malignant astrocytic tumor, is composed of poorly differentiated neoplastic astrocytes, accounting for nearly 70% of all diffuse astrocytic tumors and 10–15% of all intracranial neoplastic lesions (Ohgaki and Kleihues, 2005). GBM is generally ranked as the most aggressive type among all brain tumors according to WHO classification (Møller et al., 2013). Despite GBM hardly metastasizes to distant organs, it possesses the tendency to infiltrate the surrounding brain tissues partially attributing to the capacity of rapid proliferation (Zhong et al., 2010). With the highly invasive nature, most of patients with GBM will eventually recurs after surgical resection, owing to the impossibility that all tumors cells in patients are completely removed (Koo et al., 2012). Subsequent to surgery, radiotherapy along with giving temozolomide are the mainstay of treatment for patients with GBM (Khosla, 2016). However, the prognosis of GBM is still dismal, with a median survival ranging from 12 to 18 months (Roy et al., 2015). Although intensive efforts have been spent in basic, translational and clinical studies, there is still no obvious clinical improvement in GBM over the past decades. Therefore, it seems meaningful to seek and develop an effective therapeutic modality for GBM.

MicroRNAs (miRNAs) are a class of small endogenous non-coding RNA molecules containing approximately 22 nucleotides (Lou et al., 2018). Through base-pairing with complementary sequences within messenger RNA (mRNA) molecules, miRNA plays a pivotal role in RNA silencing and post-transcriptional regulation of gene expression, thereby taking part in numerous cellular processes, such as cell proliferation (Yan et al., 2018), apoptosis (Yang et al., 2018), cell cycle (Li et al., 2014), migration (Xiao et al., 2015), differentiation (Dotto and Karine, 2014), and energy metabolism (Singh et al., 2012). The dysregulation of miRNA has a link with multiple human diseases, including obesity (Carreras-Badosa et al., 2015), kidney disease (Trionfini et al., 2015), heart disease (Miller et al., 2014) and nervous system disorder (Bekris and Leverenz, 2015). Many studies have well supported that there is a close relationship between miRNA and human cancer (Tutar, 2014). MiRNAs are also reported to exert key functions in the onset and progression of GBM. For example, miR-454-3p suppresses cell migration and invasion through negatively regulating CPEB1 expression in GBM (Hui et al., 2018); miR-574 inhibits cell proliferation and invasion via direct suppression of zinc finger E-box-binding homeobox 1 in GBM (Mao et al., 2018); miR-124-3p exerts a crucial role in mediating growth and angiogenesis of GBM by targeting NRP-1 (Zhang et al., 2018). Despite a lot of investigations regarding miRNA expression and function in GBM have been launched, to our knowledge, a systematic and comprehensive analysis of miRNA-mRNA regulatory network based on clinical samples of GBM is still absent. Construction of potential miRNA-mRNA regulatory network contributing to GBM will bring to light a relatively all-round molecular mechanism of miRNAs' impact in GBM.

Herein, we first screened several differentially expressed miRNAs (DE-miRNAs) in GBM tissues compared with normal tissues by analyzing GSE90603 dataset downloaded from GEO database. FunRich was employed to predict the upstream transcription factors of DE-miRNAs. Next, miRNet database was introduced to predict potential target genes. Then, DE-mRNAs between GBM tissues and normal tissues were obtained using GBM mRNA dataset downloaded from TCGA database. Subsequently, GO functional annotation and KEGG pathway enrichment analysis was conducted by the Enrichr database. Hub genes were identified with the help of Cytoscape software and the expression levels and prognostic roles of these hub genes were further determined by GEPIA database and PrognoScan database. Finally, a potential miRNA-mRNA regulatory network contributing to the onset and progression of GBM was successfully established.

## Materials and Methods

### Microarray Dataset

At the discovery step, we searched datasets focusing on the miRNA expression in GBM in the National Center for Biotechnology Information (NCBI) Gene Expression Omnibus (GEO) database (https://www.ncbi.nlm.nih.gov/geo/). Besides, we only included those datasets based on GBM clinical patients, and the datasets based on GBM cell lines or animal models were excluded. Additionally, those datasets containing <10 GBM samples were also not included. Finally, only microarray dataset GSE90603 met these criteria mentioned above and was selected for our subsequent analysis. Dataset GSE90603 was based on the platform of GPL21572 (Affymetrix Multispecies miRNA-4 Array) and contained 16 fresh-frozen GBM samples, 4 adjacent health samples from patients with GBM and 3 health samples from healthy volunteer.

### Identification of Differentially Expressed miRNAs (DE-miRNAs)

GEO2R online tool (https://www.ncbi.nlm.nih.gov/geo/geo2r/) based on the R software “LIMMA” package was used to identify DE-miRNAs between GBM tissues and normal tissues. Four different groups, including A [GBM tumor tissue samples (*n* = 16)], B [adjacent normal brain tissue samples from GBM patients (*n* = 4)], C [normal brain tissue samples from healthy volunteer (*n* = 3)] and D [normal samples from group B and C (*n* = 7)], were defined. Through comparing with various normal group, different DE-miRNAs were generated. To identify the most possible miRNAs that are involved in the pathogenesis of GBM, GBM tumor samples (group A) were compared with group B, C, and D, respectively. Then, the DE-miRNAs that were commonly appeared in three differential expression analyses were finally selected for subsequent investigation. |log_2_FC| > 2 and adj *P* < 0.05 were set as the thresholds for identifying DE-miRNAs. Venn diagrams were produced by two online tools (http://bioinfogp.cnb.csic.es/tools/venny/index.html and http://bioinformatics.psb.ugent.be/webtools/Venn/).

### Prediction of Potential Transcription Factors, and Target Genes of DE-miRNAs

The upstream transcription factors of screened DE-miRNAs were predicted using FunRich software, which is a tool used mainly for functional enrichment and interaction network analysis of genes and proteins (Pathan et al., 2017). The screened upregulated and downregulated DE-miRNAs were typed into this software. The top 10 predicted transcription factors were shown.

Subsequently, miRNet database, an easy-to use tool for comprehensive statistical analysis and functional interpretation of data from miRNAs studies, was employed to predict the downstream target genes of screened DE-miRNAs (Fan et al., 2016).

### The Cancer Genome Atlas (TCGA) Data Processing

To improve the reliability of our subsequent analysis for target genes of screened DE-miRNAs, we next verified these target genes expression in TCGA datasets. TCGA RNA-seq raw data from 5 normal samples and 169 tumor samples of GBM were firstly downloaded from TCGA database. Then, the raw data were standardized by the method of log2(x+1) and normalized through the normalizeBetweenArray function from R package “LIMMA” from the Bioconductor project (Smyth et al., 2005).

### Identification of Differentially Expressed Genes (DEGs)

After normalization, the differential expression analysis was conducted using the LIMMA software package (Ritchie et al., 2015). |log_2_FC| > 2 and adj *P* < 0.05 were set as the thresholds for identifying DEGs.

### GO Annotation and KEGG Pathway Enrichment Analysis

The Enrichr database (http://amp.pharm.mssm.edu/Enrichr/) was utilized to perform GO functional annotation and KEGG pathway enrichment analysis for the intersection of target genes and DE-mRNAs (Kuleshov et al., 2016). The GO analysis included three categories: biological process (BP), cellular component (CC), and molecular function (MF). *P* < 0.05 was considered as statistically significant.

### Establishment and Analysis of Protein-Protein Interaction (PPI) Network

To better know the relationship among these screened genes, the PPI network was established using the STRING database (Szklarczyk et al., 2017). PPI node pairs with a combined score ≥0.4 were selected for further analysis. The hub genes in the PPI network were identified according to degree using Cytoscape software (version 3.6.1).

### Validation of Hub Gene Expression Levels

The expression levels of top 20 hub genes were further validated using the Gene Expression Profiling Interactive Analysis (GEPIA), which is a newly developed interactive web server for analyzing the RNA sequencing expression data of 9,736 tumors and 8,587 normal samples from the TCGA and the GTEx projects (Tang et al., 2017). Hub genes with |log_2_FC| > 2 and *P* < 0.05 were considered as statistically significant.

### Prognoscan Database Analysis

The prognostic roles of screened hub genes in GBM were analyzed using PrognoScan database (http://www.abren.net/PrognoScan/), which is a new database for meta-analysis of the prognostic value of genes (Mizuno et al., 2009). Cox *P* < 0.05 was considered as significant and corresponding information and analyses were listed in **Table 4**.

### Statistical Analysis

Most of the statistical analysis has been done by the bioinformatic tools mentioned above. When we conducted differential expression analysis, only genes or miRNAs with |log_2_FC| > 2 and *P* < 0.05 were considered as statistically significant. Cox *P* < 0.05 was considered as statistically significant for survival analysis.

## Results

### Identification of Candidate DE-miRNAs

**Figure 1:**
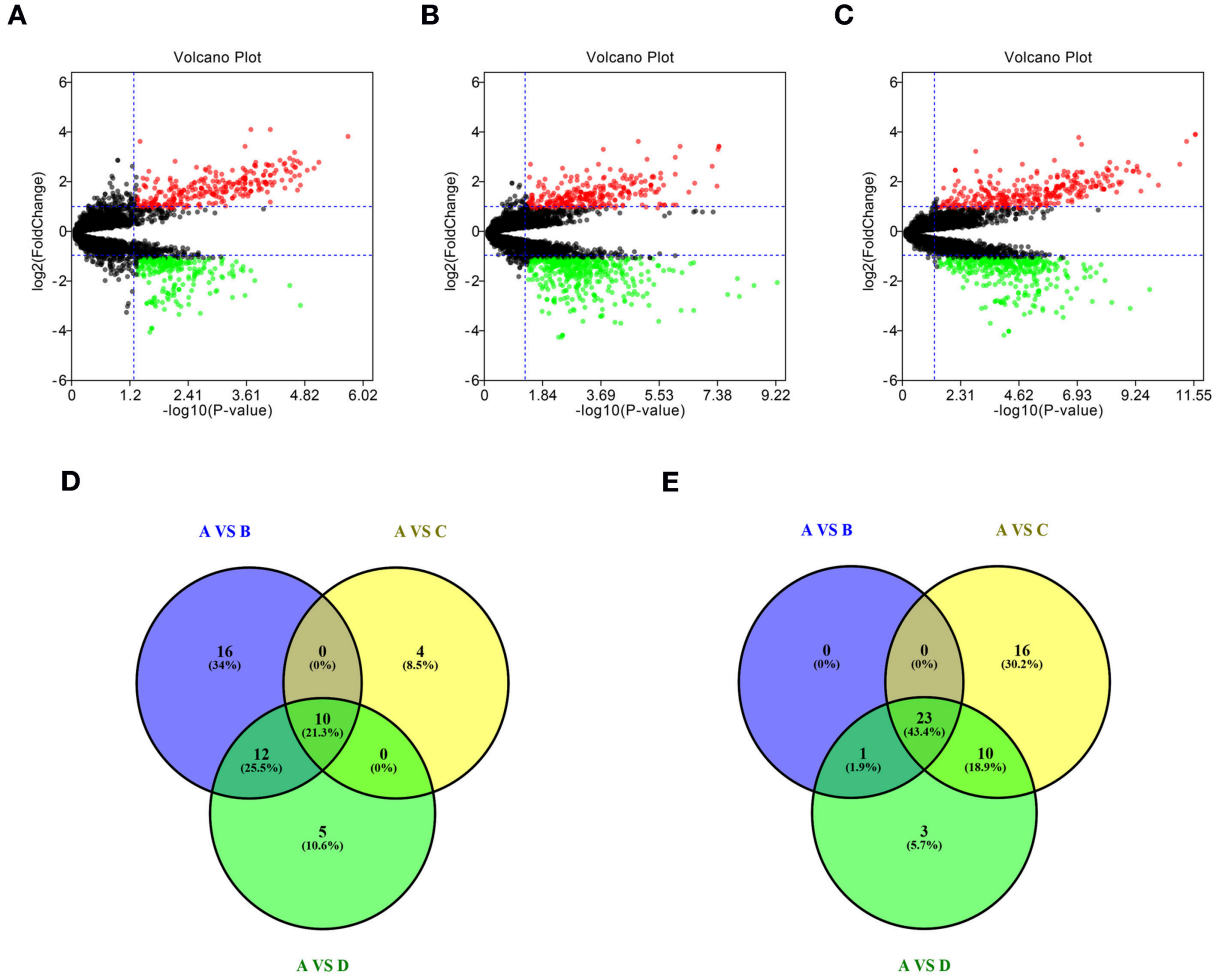
Identification of differentially expressed miRNAs (DE-miRNAs). **(A)** DE-miRNAs between group A and group B; **(B)** DE-miRNAs between group A and group C; **(C)** DE-miRNAs between group A and group D; **(D)** intersection of upregulated DE-miRNAs in three compared sets; **(E)** intersection of downregulated DE-miRNAs in three compared sets. Group A contained GBM tumor tissue samples (*n* = 16); group B contained adjacent normal brain tissue samples from GBM patients (*n* = 4); group C contained normal brain tissue samples from healthy volunteer (*n* = 3); group D contained normal samples from group B and C (*n* = 7). |log_2_FC| > 2 and adj *P* < 0.05 were set as the thresholds for identifying DE-miRNAs. Red dots and green dots represent the upregulated and downregulated miRNAs in GBM tumor samples, respectively; black dots represent miRNAs that are not differentially expressed between tumor samples and normal samples.

To identify potential miRNA-mRNA regulatory network in GBM, dataset GSE90603 was first selected to screen DE-miRNAs between GBM samples and normal samples. By various defined groups, DE-miRNAs were identified using GEO2R (Figures 1A–C). Only DE-miRNAs commonly appeared in three sets were chosen as candidate DE-miRNAs. As shown in Figures 1D,E, 10 upregulated DE-miRNAs (miR-21-5p, miR-25-3p, miR-106b-3p, miR-155-5p, miR-10b-5p, mir-92b, miR-92b-3p, miR-424-3p, miR-181a-2-3p, and miR-15b-5p) and 23 downregulated DE-miRNAs (mir-1225, miR-7-5p, miR-139-5p, miR-124-3p, miR-330-3p, mir-139, mir-124, miR-128-3p, miR-490-5p, miR-139-3p, miR-6782-5p, miR-128-2-5p, miR-770-5p, miR-338-3p, miR-873-3p, mir-137, miR-124-5p, miR-433-3p, miR-218-5p, miR-758-5p, and miR-138-2-3p) were finally identified.

### Prediction of Upstream Transcription Factors of DE-miRNAs

**Figure 2:**
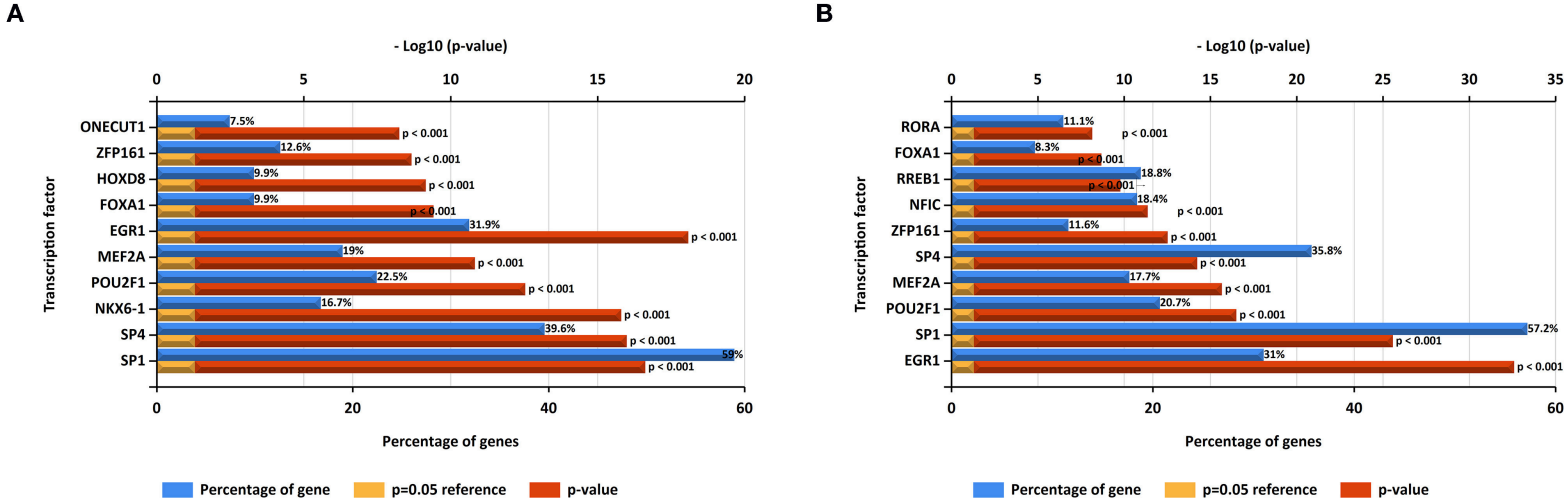
Predicted transcription factors of DE-miRNAs. **(A)** Transcription factors of upregulated DE-miRNAs; **(B)** transcription factors of downregulated DE-miRNAs.

In this study, upstream transcription factors of candidate upregulated and downregulated DE-miRNAs were predicted by using FunRich software. The top 10 transcription factors for upregulated and downregulated DE-miRNAs were presented in Figures 2A,B, respectively. For upregulated DE-miRNAs, the top 10 transcription factors were SP1, SP4, NKX6-1, POU2F1, MEF2A, EGR1, FOXA1, HOXD8, ZFP161, and ONECUT1. For downregulated DE-miRNAs, the top 10 transcription factors were EGR1, SP1, POU2F1, MEF2A, ZFP161, NFIC, RREB1, FOXA1, and RORA.

### Prediction of Downstream Target Genes of DE-miRNAs

**Figure 3:**
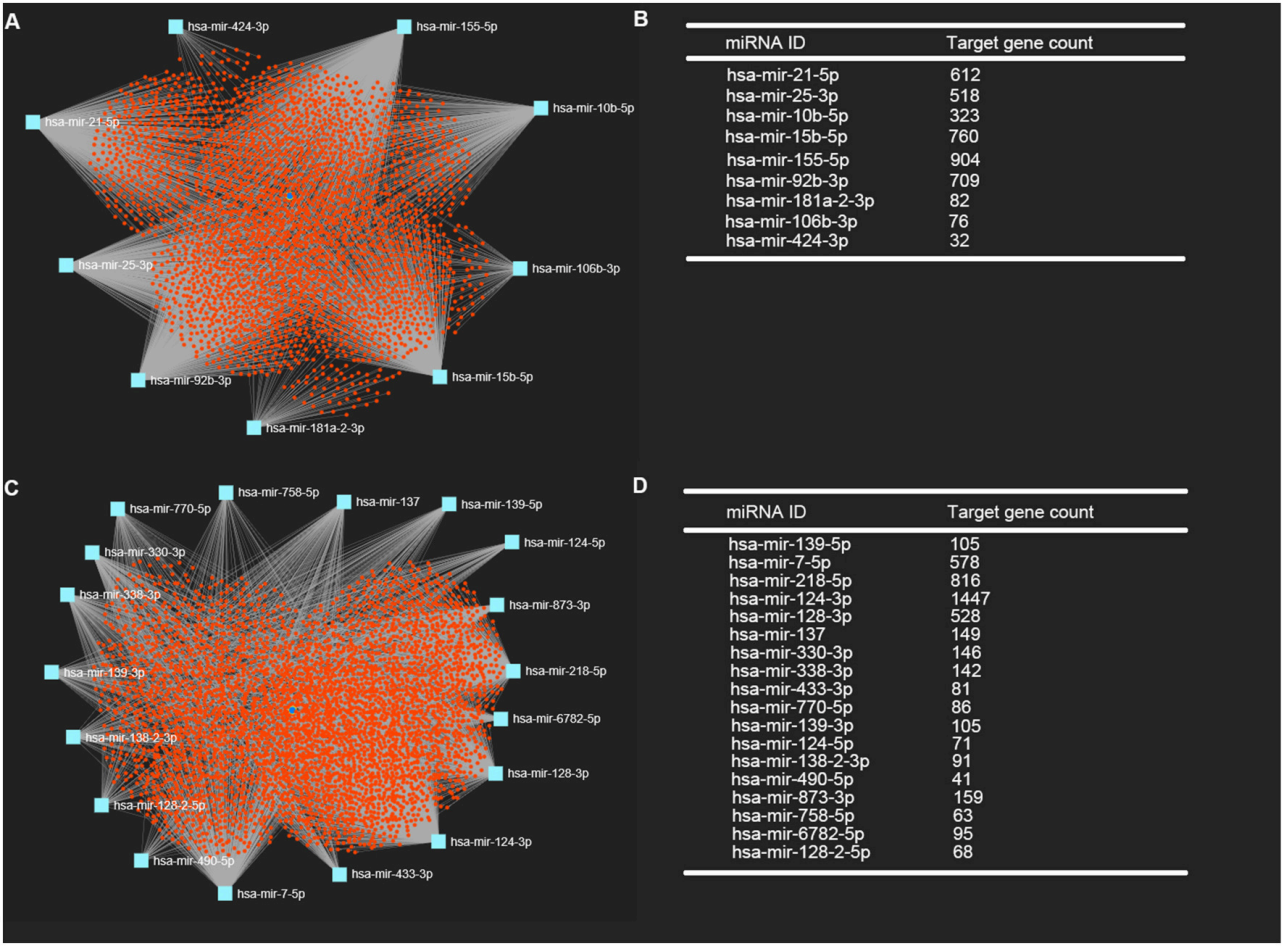
Potential target genes of DE-miRNAs predicted by miRNet database. **(A)** Upregulated DE-miRNAs-target genes network constructed using miRNet; **(B)** downregulated DE-miRNAs-target genes network constructed using miRNet; **(C)** target gene count for each upregulated DE-miRNA; **(D)** target gene count for each downregulated DE-miRNA.

It is widely acknowledged that miRNAs exert their biological effect mainly through directly targeting 3' untranslated region of messenger RNA. Thus, we predicted the downstream target genes of candidate DE-miRNA by miRNet database. 3,027 and 3,879 target genes were finally predicted for the upregulated DE-miRNAs and downregulated DE-miRNAs, respectively. These predicted target genes were listed in Table S1. Moreover, for better visualization, upregulated DE-miRNA-target gene network and downregulated DE-miRNA- target gene network were successively established and presented in Figures 3A,C, respectively. Additionally, the target gene count for each DE-miRNA were also listed in Figures 3B,D.

### Identification of Candidate Target Genes

**Figure 4:**
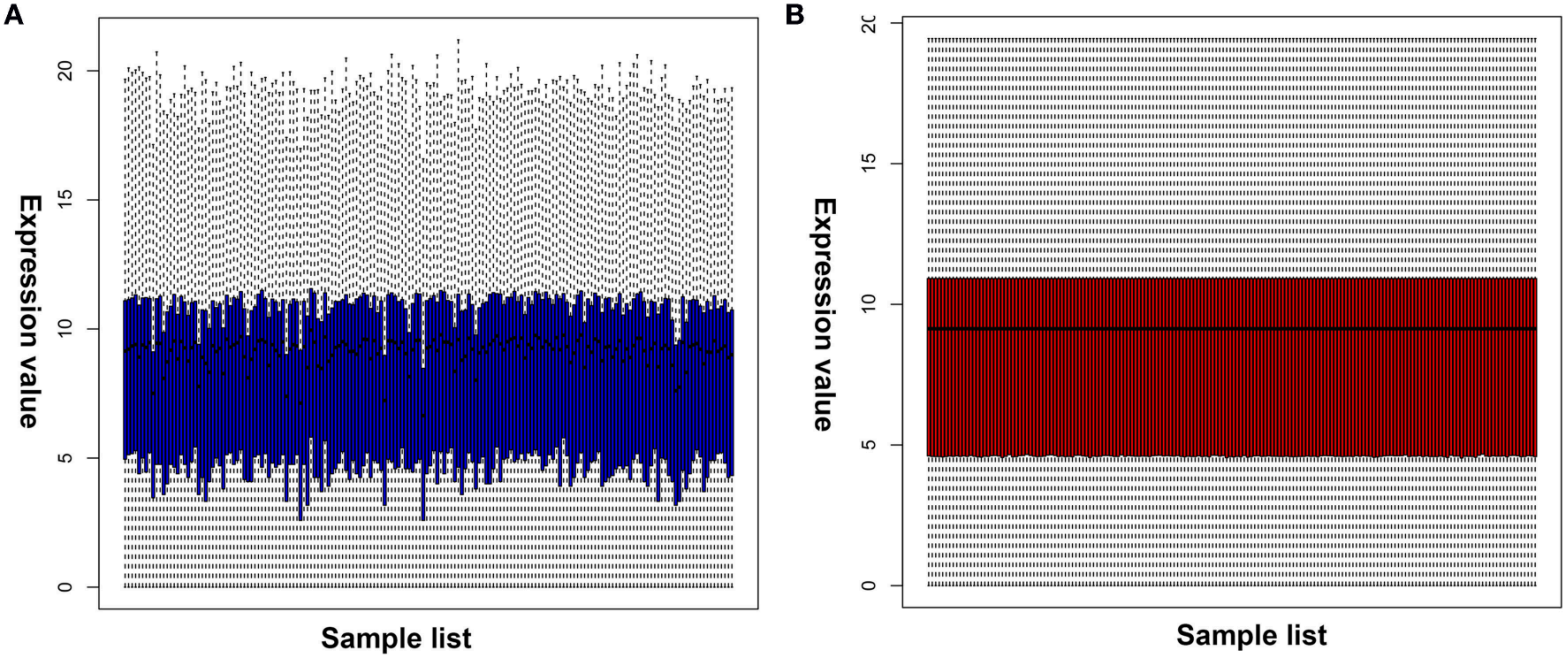
Normalization of TCGA data. **(A)** Data before normalization; **(B)** data after normalization.

**Figure 5:**
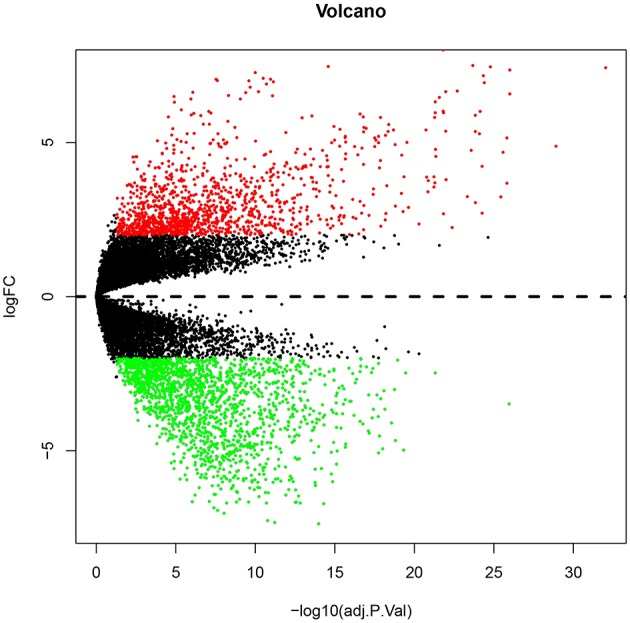
The differentially expressed genes (DEGs) between GBM samples and normal samples from TCGA database. The red and green dots represent significantly upregulated and downregulated DEGs, respectively. The black dots represent genes that are not differentially expressed tumor samples and normal samples.

**Figure 6:**
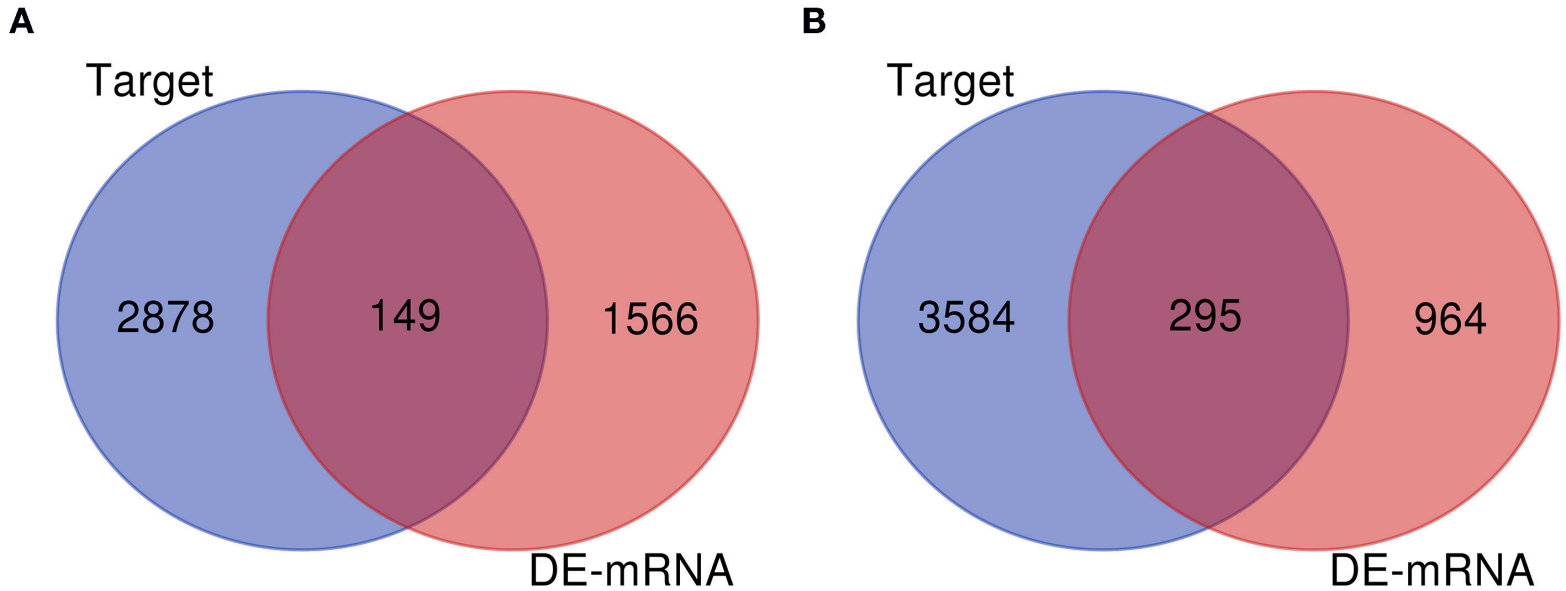
Screen of candidate genes. **(A)** The intersection of target genes of upregulated DE-miRNAs and downregulated DEGs; **(B)** the intersection of target genes of downregulated DE-miRNAs and upregulated DEGs.

Numerous evidences have supported an inverse relationship between miRNA and target gene. We first intended to identify DEGs between GBM samples and normal samples using TCGA mRNA data. Data downloaded from TCGA database were first normalized. Data before and after normalization were depicted in Figures 4A,B, respectively. Subsequently, 1,259 upregulated DE-mRNAs and 1,715 downregulated DE-mRNAs were identified as shown in Figure 5 and Table S2. After conducting a combined analysis of DE-mRNAs and target genes of DE-miRNAs, we further screened 149 and 295 candidate target genes for upregulated and downregulated DE-miRNAs, respectively (Figure 6 and Table S3).

### Functional Annotation and Pathway Enrichment Analysis

**Figure 7:**
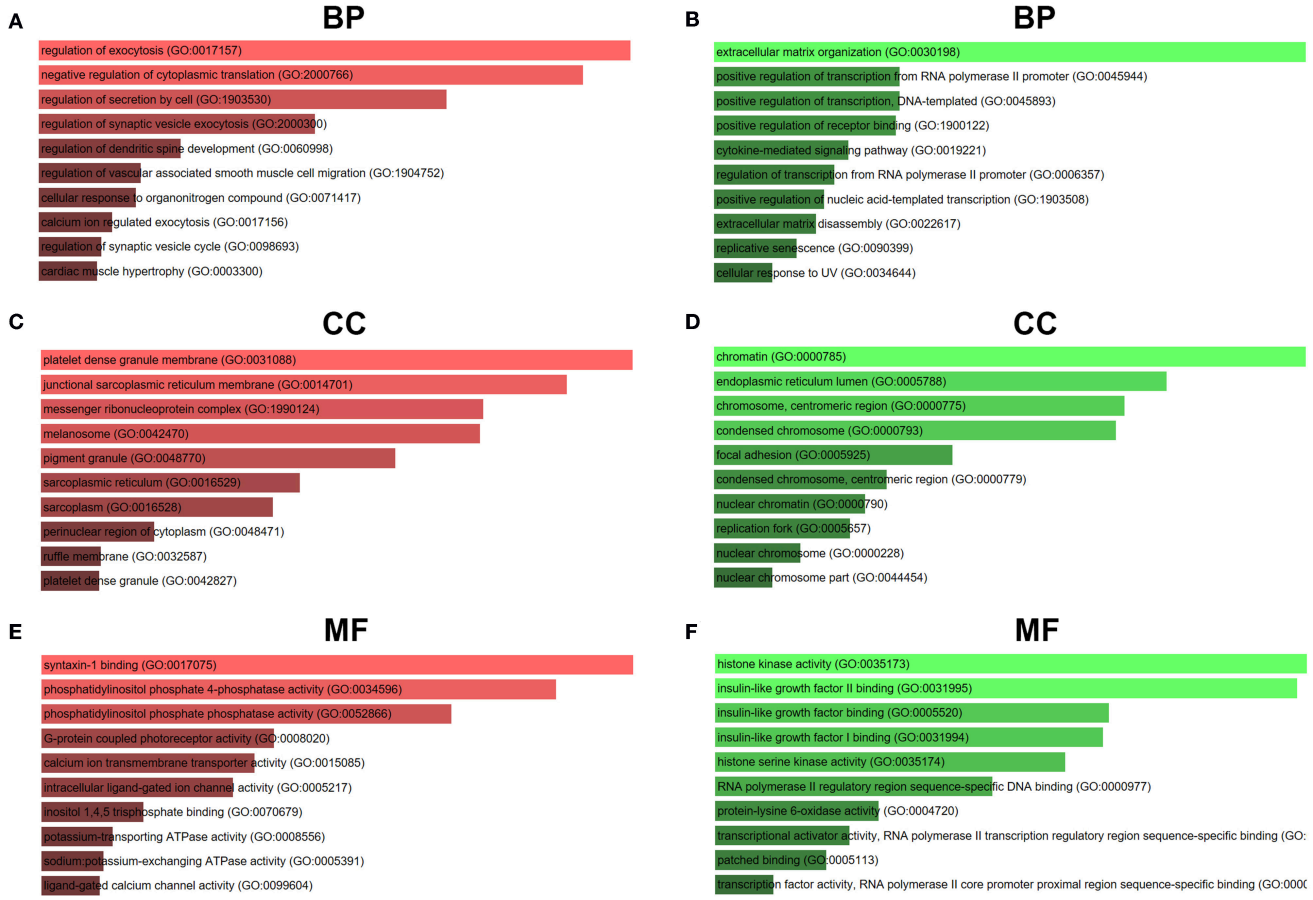
GO functional annotation of candidate genes. **(A)** The top 10 enriched BP items of downregulated candidate genes; **(B)** the top 10 enriched BP items of upregulated candidate genes; **(C)** the top 10 enriched CC items of downregulated candidate genes; **(D)** the top 10 enriched CC items of upregulated candidate genes; **(E)** the top 10 enriched MF items of downregulated candidate genes; **(F)** the top 10 enriched MF items of upregulated candidate genes.

For better understanding these screened candidate target genes, Enrichr database was utilized to perform GO function and KEGG pathway enrichment analysis. GO functional annotation include three categories, namely biological process (BP), cellular component (CC), and molecular function (MF). The top 10 enriched GO items were listed in Figure 7. GO BP analysis revealed that candidate target genes of upregulated DE-miRNAs were significantly enriched in regulation of exocytosis, negative regulation of cytoplasmic translation and regulation of secretion by cell (Figure 7A). For CC analysis, these genes were significantly enriched in platelet dense granule membrane, junctional sarcoplasmic reticulum membrane and messenger ribonucleoprotein complex (Figure 7C). The MF analysis for these genes included syntaxin-1 binding, phosphatidylinositol phosphate 4-phosphatase activity and phosphatidylinositol phosphate phosphatase activity (Figure 7E). GO BP analysis demonstrated that candidate target genes of downregulated DE-miRNAs were significantly enriched in extracellular matrix organization, positive regulation of transcription from RNA polymerase II promoter and positive regulation of transcription DNA-templated (Figure 7B). For CC analysis, these genes were significantly enriched in chromatin, endoplasmic reticulum lumen and chromosome centromeric region (Figure 7D). At last MF analysis for these genes revealed that they were significantly enriched in histone kinase activity, insulin-like growth factor II binding, and insulin-like growth factor binding (Figure 7F).

KEGG pathway enrichment analysis was further conducted for candidate target genes of upregulated and downregulated DE-miRNAs. As listed in Table 1, candidate target genes of upregulated DE-miRNAs were significantly enriched in cGMP-PKG signaling pathway, calcium signaling pathway, pancreatic secretion, thyroid hormone synthesis, oocyte meiosis, GABAergic synapse, salivary secretion, oxytocin signaling pathway, endocrine and other factor-regulated calcium reabsorption, and MAPK signaling pathway. The enriched pathways for candidate target genes of downregulated DE-miRNAs were pathways in cancer, cell cycle, bladder cancer, proteoglycans in cancer, small cell lung cancer, ECM-receptor interaction, PI3K-Akt signaling pathway, focal adhesion, pancreatic cancer and hepatitis B as shown in Table 2.

**Table 1:** The top 10 enriched KEGG pathways for the intersection of target genes of upregulated DE-miRNAs and downregulated DEGs.

**Term**	**Count**	***P*-value**
cGMP-PKG signaling pathway	7	0.000259
Calcium signaling pathway	7	0.000408
Pancreatic secretion	5	0.000761
Thyroid hormone synthesis	4	0.001952
Oocyte meiosis	5	0.002292
GABAergic synapse	4	0.004251
Salivary secretion	4	0.004425
Oxytocin signaling pathway	5	0.006642
Endocrine and other factor-regulated calcium reabsorption	3	0.00517
MAPK signaling pathway	6	0.01226

**Table 2:** The top 10 enriched KEGG pathways for the intersection of target genes of downregulated DE-miRNAs and upregulated DE-mRNAs.

**Term**	**Count**	***P*-value**
Pathways in cancer	24	5.98E-09
Cell cycle	13	3.82E-08
Bladder cancer	8	1.28E-07
Proteoglycans in cancer	15	3.67E-07
Small cell lung cancer	10	5.52E-07
ECM-receptor interaction	9	3.34E-06
PI3K-Akt signaling pathway	16	5.09E-05
Focal adhesion	12	4.86E-05
Pancreatic cancer	7	5.23E-05
Hepatitis B	10	6.34E-05

### Screen of Hub Genes

**Figure 8:**
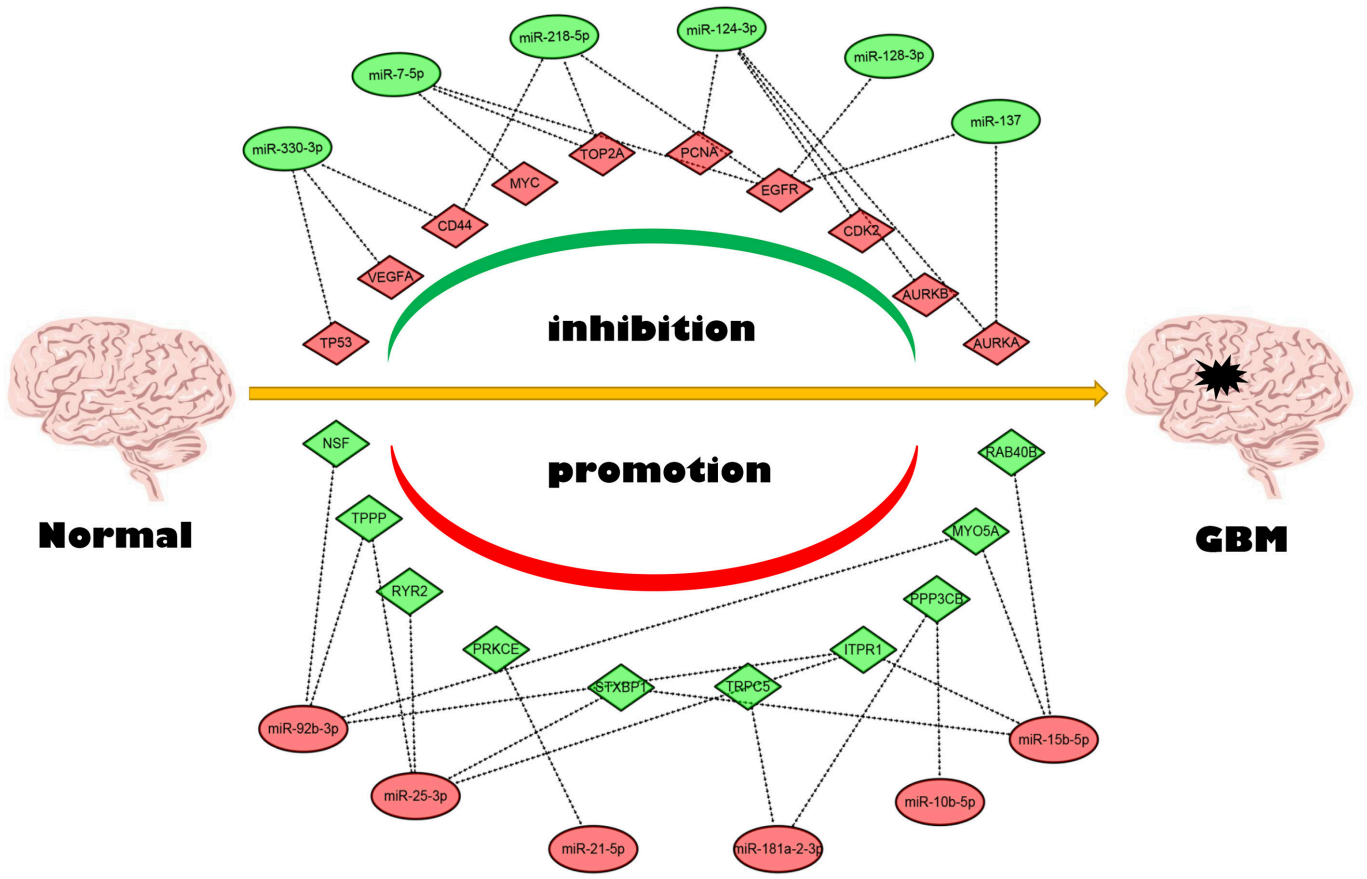
The candidate miRNA-hub gene regulatory network in GBM.

Next, we mapped these candidate target genes into the STRING database. Based on the information from this public database, we constructed PPI network of these genes. 69 and 1,313 node pairs for candidate target genes of upregulated DE-miRNAs and downregulated DE-miRNAs were finally obtained. Aim to access hub genes in the two PPI networks, these node pairs were input into Cytoscape software. The top 10 hub genes were listed in Table 3. For the target genes of upregulated DE-miRNAs, the hub genes were STXBP1, MYO5A, NSF, ITPR1, PPP3CB, PRKCE, RAB40B, TPPP, TRPC5, and RYR2. For the target genes of downregulated DE-miRNAs, the hub genes were TP53, TOP2A, MYC, EGFR, VEGFA, PCNA, CD44, AURKB, and AURKA. According to the predicted miRNA-mRNA pairs, the candidate miRNA-hub gene regulatory network associated with development of GBM were finally constructed as presented in Figure 8.

**Table 3:** Hub genes in the PPI networks.

**Downregulated candidate genes**		**Upregulated candidate genes**	
**Name**	**Node**	**Name**	**Node**
STXBP1	8	TP53	75
MYO5A	6	TOP2A	67
NSF	6	MYC	60
ITPR1	6	EGFR	55
PPP3CB	6	VEGFA	54
PRKCE	4	PCNA	54
RAB40B	3	CD44	47
TPPP	3	AURKB	46
TRPC5	3	AURKA	45
RYR2	3	CDK2	44

### Identification of Potential miRNA-mRNA Regulatory Pathways

**Figure 9:**
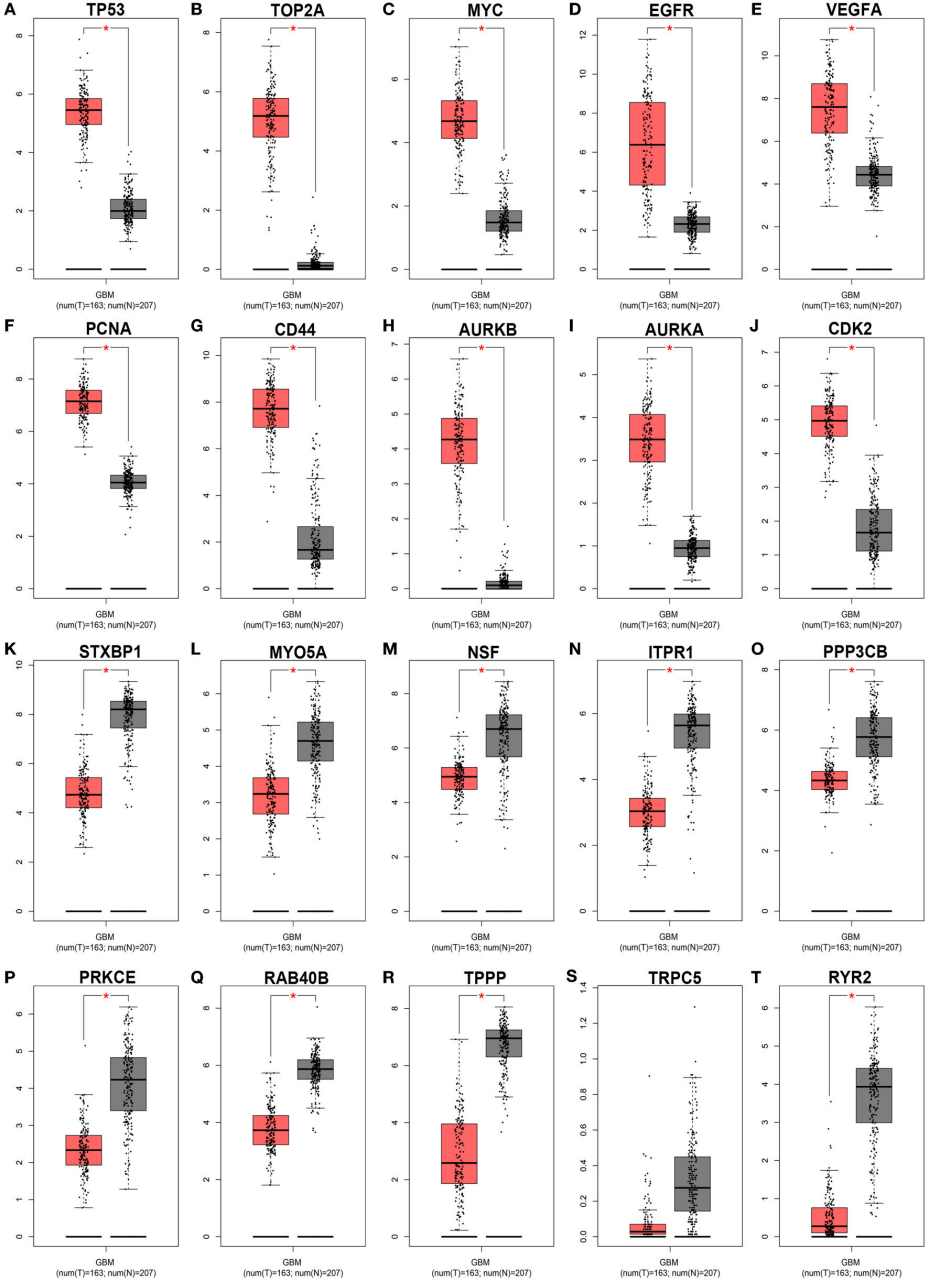
The expression levels of 20 hub genes from the GEPIA database. ^*^P < 0.05.

Subsequently, GEPIA database was used to detect the 20 hub genes expression levels. As shown in Figure 9, the expression levels of all the 10 hub genes of downregulated DE-miRNAs were significantly higher in GBM tissues than that in normal tissues. Nine of 10 hub genes of upregulated DE-miRNAs were markedly upregulated in GBM tissues when compared with normal tissues. The expression analysis of TRPC5 demonstrated no significant difference between GBM and normal samples.

For further identifying potential hub genes, the prognostic roles of these 20 hub genes in GBM were evaluated by PrognoScan database. As listed in Table 4, the higher expression of EGFR indicated a worse prognosis whereas the higher expression of PPP3CB and MYO5A showed a better prognosis in GBM.

**Table 4:** The association of hub gene expression and the survival in patients with GBM.

**Gene symbol**	**EGFR**	**EGFR**	**EGFR**	**PPP3CB**	**PPP3CB**	**MYO5A**
Dataset	GSE7696	GSE7696	GSE7696	GSE7696	GSE7696	GSE7696
Endpoint	Overall survival	Overall survival	Overall survival	Overall survival	Overall survival	Overall survival
Probe ID	201984_s_at	201983_s_at	211607_x_at	209817_at_	202432_at	204527_at
N	70	70	70	70	70	70
Cox *P*-value	0.015	0.003	0.012	0.035	0.031	0.000
HR	1.20	1.23	1.80	0.46	0.50	0.33
95% CI	1.04–1.39	1.07–1.41	1.14–2.84	0.22–0.95	0.27–0.94	0.18–0.61
Effect	Worse	Worse	Worse	Better	Better	Better

Conjoint analysis of the expression pattern and prognosis roles of hub genes demonstrated the oncogenic effect of EGFR and tumor suppressive effect of PPP3CB and MYO5A in GBM. These were in line with the previous prediction of them as potential target genes of tumor suppressive and oncogenic miRNAs. Based on these findings, a potential miRNA-mRNA regulatory network contributing to GBM onset and progression was established, including miR-10b-5p/miR-181a-2-3p-PPP3CB, miR-92b-3p/miR-15b-5p-MYO5A, and miR-7-5p/miR-218-5p/miR-128-3p/miR-137-EGFR regulatory pathways.

## Discussion

It is believed that there is a significant link between miRNA-mRNA regulatory network and brain (Adlakha and Saini, 2014; Petri et al., 2014). The dysregulation of miRNA-mRNA regulatory network in brain leads to a variety of human diseases, such as Alzheimer's disease (Bekris et al., 2013; Bekris and Leverenz, 2015; Hara et al., 2017), Parkinson's disease (Burgos et al., 2014), cerebral infarction (Liang et al., 2018) and ischemic neuronal injury (Feng et al., 2018). During the past few years, many studies have intensively suggested that alteration of miRNAs and their downstream target genes expression levels is closely associated with the development of brain tumors including GBM (Møller et al., 2013; Hui et al., 2018; Mao et al., 2018).

However, to our knowledge, up to now, a comprehensive miRNA-mRNA regulatory network in GBM has still not been created. In this present study, we conducted a differential expression analysis using miRNA and mRNA data from GEO database and TCGA database. Ten upregulated DE-miRNAs and 23 downregulated DE-miRNAs were finally identified. Previous studies have shown that most of expression of DE-miRNAs that we screened was identical with our analytic results. For example, miR-21-5p is overexpressed in GBM cell lines and tumor tissue (Yang et al., 2014; Shang et al., 2015); upregulation of miR-10b-5p functions as an oncogene in GBM (Guessous et al., 2013; Ma et al., 2017); miR-7-5p is found to be significantly downregulated in GBM (Kefas et al., 2008; Liu et al., 2014); miR-137 expression is lower in GBM than that in normal tissues (Bier et al., 2013).

Previous studies have suggested that miRNA expression can be modulated by transcription factors (Mullany et al., 2018; Si et al., 2018). Therefore, we predicted the transcription factors that can potentially regulating these DE-miRNAs. Specificity protein 1 (SP1), a zinc finger transcription factor that binds to GC-rich motifs of many promotes, was predicted as the transcription factor that could potentially regulate expression of a majority of screened DE-miRNAs. It has been reported to act as a key player in modulating miRNA expression and function. For instance, a recent investigation has demonstrated that SP1 is involved in miR-183-5p-IκB-α signaling pathway, thereby contributing to cerebral ischemia (Qin et al., 2018). Its regulatory roles in brain tumors has also been well documented (Dong et al., 2014). More experiments about the roles of these predicted transcription factors in GBM need to be further investigated in the future.

Then, through integrating DE-mRNAs and target genes of DE-miRNAs, multiple candidate genes were screened, consisting of 149 downregulated and 295 upregulated genes. Subsequent pathway enrichment analysis revealed that the downregulated genes were mainly enriched in cGMP-PKG signaling pathway and calcium signaling pathway, whereas the upregulated genes were primarily enriched in pathways in cancer and PI3K-Akt signaling pathway. A study performed by Charles et al. indicated that cGMP-PKG pathway promoted stem-like character of glioma (Charles et al., 2010). A plenty of studies have also suggested that calcium signaling pathway correlates with human cancers including GBM (Robil et al., 2015; Leclerc et al., 2016). The activation of PI3K-Akt signaling pathway has been verified to have an association with GBM progression and poor prognosis (Guan et al., 2018; Li et al., 2018). These reports further support our previous analytic results.

Next, PPI network was constructed and the top 20 hub genes, containing 10 upregulated and 10 downregulated genes, were identified. Furthermore, the expression analysis of these hub genes in GBM were further evaluated using GEPIA database, which includes more normal samples than TCGA database (Tang et al., 2017). Inspiringly, the 20 genes expression were generally in accordance with our analytic results of TCGA mRNA data. The majority of these genes has been identified to act as pivotal modulators in GBM. For example, inhibition of VEGFA/VEGFR2 expression suppresses GBM growth (Serrill et al., 2016); EGFR suppression hinders GBM growth (Shen et al., 2018); MYC activation in GBM drives glycolysis, thereby fueling pathogenesis of GBM (Tateishi et al., 2016); CD44 expression is overexpressed in GBM and the increased expression of CD44 promotes GBM aggressiveness including mediation of migration (Chetty et al., 2012; Mooney et al., 2016). Furthermore, analyses of hub genes' prognostic roles demonstrated significant oncogenic effect of EGFR and tumor suppressive effect of PPP3CB and MYO5A in GBM. Finally, based on these findings, we established a potential miRNA-mRNA regulatory network. Despite the expression and effect of these miRNAs and mRNAs in cancers have been previously confirmed as we discussed above, most of these miRNA-mRNA pairs potentially contributing to the pathogenesis of GBM listed in the network remain not be studied, which is of importance for exploring and developing novel mechanisms and therapeutic targets.

Although an integrated *in silico* analysis has been performed and a potential miRNA-mRNA regulatory network has been constructed in this present study, some limitations exist. The sample size of GEO dataset included in this study is not big enough. Lacking of experimental validation of the direct relationship of these miRNA-mRNA pairs in the constructed network is another limitation of the study. In addition, we do not conduct *in vitro* and *in vivo* function experiments of these miRNA-mRNA regulatory pathways in GBM. Corresponding experiments will be performed to verified in our future work, thus conversely testifying *in silico* analysis.

## Conclusions

In summary, we constructed a potential miRNA-mRNA regulatory network involving in the pathogenesis of GBM. This study indicates a relatively across-the-aboard potential mechanisms of miRNA-mRNA regulatory axes in pathogenesis of GBM and may assist in the treatment of GBM and improve prognosis of patients with GBM by targeting the established miRNA-mRNA regulatory network in the future.

## Data Availability

GSE90603 dataset can be found in the GEO database and GBM mRNA data can be downloaded from TCGA database.

## Author Contributions

WL and WF conceived and designed this study. WL wrote this manuscript. WF revised this manuscript. WL made these figures with the help of BD and LX.

### Conflict of Interest Statement

The authors declare that the research was conducted in the absence of any commercial or financial relationships that could be construed as a potential conflict of interest.

## Abbreviations

BP: Biological process
CC: Cellular component
GEO: Gene Expression Omnibus
GBM: Glioblastoma multiforme
GEPIA: Gene Expression Profiling Interactive Analysis
microRNA: miRNA
MF: Molecular function
OS: overall survival
PPI: Protein-protein interaction
SP1: Specificity protein 1
TCGA: The Cancer Genome Atlas.
