# Supplementary material for: Construction of Potential Glioblastoma Multiforme-Related miRNA-mRNA Regulatory Network
Source: Front Mol Neurosci. 2019 Mar 26;12:66. doi: 10.3389/fnmol.2019.00066 (PMC6444190; doi:10.3389/fnmol.2019.00066)
Supplement: Supplementary file 1 [file Table_1.DOCX]

Table S1. Target genes of upregulated and downregulated DE-miRNAs.

| Target genes of upregulated DE-miRNAs | Target genes of downregulated DE-miRNAs |
| --- | --- |
| ACAT1 | USP6NL |
| PARP1 | ARHGAP32 |
| JAG1 | MLEC |
| CRYBG1 | PSME3 |
| AKT2 | STAMBP |
| APAF1 | SUGT1 |
| APC | GABARAP |
| FAS | OIP5 |
| FASLG | ZHX2 |
| RHOB | CCT5 |
| ATP2B4 | FNBP4 |
| ATRX | SLC39A6 |
| BCAT1 | CLIC4 |
| BCL2 | FAM162A |
| BCL6 | TNRC6A |
| BCL7A | TMED7 |
| BMI1 | HOOK1 |
| BMPR2 | PEX5L |
| BNIP2 | YTHDF1 |
| BRCA1 | TMEM19 |
| KLF9 | RHOT1 |
| KLF5 | CAND1 |
| CALD1 | CTTNBP2NL |
| CASP8 | KCNQ5 |
| KRIT1 | C21orf59 |
| CCNG1 | DUSP22 |
| CD47 | CIAPIN1 |
| CDC25A | MKL2 |
| CDK6 | ZBTB26 |
| CEBPB | RAP2C |
| FOXN3 | ADGRL4 |
| CLCN5 | IPPK |
| CLU | TAF1D |
| CCR1 | WNT1 |
| CCR7 | BTG2 |
| COL4A1 | CXCR4 |
| COL5A2 | SGPL1 |
| SLC31A1 | GLP2R |
| ATF2 | ROCK2 |
| HAPLN1 | PDE4DIP |
| CSNK1A1 | LCOR |
| DAXX | TGS1 |
| DDX3X | PDZD8 |
| DLG1 | UHMK1 |
| DMD | DCBLD2 |
| DYNC1LI2 | ZNF480 |
| SLC26A2 | B3GALNT2 |
| DUSP8 | FAM84B |
| E2F1 | PAPD4 |
| E2F2 | ADAMTS17 |
| E2F3 | ZNF431 |
| MEGF9 | ZNF367 |
| EGFR | SPATA13 |
| EIF1AX | EPGN |
| EIF2S1 | KSR2 |
| EIF4A2 | TMPRSS12 |
| EIF4EBP2 | DCAF4L1 |
| EIF5 | NANOGNB |
| ELAVL4 | NCR3LG1 |
| EPHA4 | ZBTB34 |
| ERBB2 | BCL2L15 |
| FGF12 | PGAM4 |
| FKBP5 | PTGFRN |
| FOXO1 | RAP1B |
| FOXO3 | RPS23 |
| FMOD | RPS26 |
| FMR1 | RREB1 |
| GLG1 | SMARCA4 |
| GNAQ | SRPK1 |
| GOLGA4 | SYT5 |
| GP5 | TAF13 |
| GPD2 | TCF12 |
| MKNK2 | DERL1 |
| MSH6 | PAGR1 |
| GTF2A1 | KLHL36 |
| GTF2I | TMC7 |
| HIF1A | FBXL18 |
| HMGB1 | CHD9 |
| HMGB3 | SPRY4 |
| HNRNPH1 | ABL2 |
| HNRNPK | ACTC1 |
| HOXA9 | SLC25A6 |
| HPGD | BCL2 |
| FOXN2 | DDX6 |
| ICAM1 | FOS |
| IGF1R | NR5A2 |
| IL1B | HMGB2 |
| IL12A | HNRNPF |
| CXCL10 | HRAS |
| IPP | HSP90AA1 |
| IRAK1 | IGF1R |
| IREB2 | JUN |
| ITGB8 | KCND3 |
| KLK2 | KPNA2 |
| TNPO1 | TNPO1 |
| LAMP2 | MCL1 |
| LIFR | MET |
| LRP6 | MMP11 |
| CAPRIN1 | NFKB1 |
| MARCKS | NOTCH1 |
| SMAD7 | PDE4D |
| MBNL1 | PIK3CA |
| MDM4 | KLF10 |
| MEF2A | TPD52 |
| MEF2C | ACTN2 |
| MEIS1 | PARP1 |
| MAP3K1 | ALDH1A3 |
| MMP2 | ACVR1B |
| MMP9 | ADCY9 |
| MSH2 | ANXA11 |
| MTAP | XIAP |
| COX2 | ALDH3B2 |
| MUC1 | ALDH3A2 |
| MYC | EIF4E |
| MYD88 | EIF4EBP2 |
| MYO9A | ENSA |
| NEK1 | FGF1 |
| NFIA | FLNA |
| NFIB | GALNT2 |
| NFKB1 | GALNT3 |
| NKTR | GATA6 |
| NTF3 | GDNF |
| DDR2 | GFI1 |
| OLR1 | GFRA1 |
| TNFRSF11B | GGCX |
| ORC4 | GLO1 |
| OXTR | GLS |
| PBX1 | GNA12 |
| PCBP1 | GPR19 |
| PDHA2 | GPR20 |
| PFKFB2 | GRB2 |
| ABCB1 | GRIN2D |
| SERPINB5 | GRINA |
| SERPINI1 | PDIA3 |
| PIK3C2A | HDLBP |
| PIK3R1 | HELLS |
| PKD2 | SLC29A2 |
| PKNOX1 | HNRNPU |
| PLAT | HOXA3 |
| PLD1 | HOXB3 |
| PPARA | HOXB5 |
| PRKAB2 | HOXC12 |
| PRKCE | HPCAL1 |
| MAP2K3 | HRH2 |
| PSMD9 | IGSF3 |
| PTEN | IDE |
| PTGFR | IDH3A |
| PTK2 | IGFBP4 |
| PTPN3 | IGFBP5 |
| PTPN14 | RBPJ |
| PTX3 | IL12RB2 |
| PURA | FOXK2 |
| PURB | ILF2 |
| ABCD3 | ILF3 |
| RAB6A | IRS1 |
| RASA1 | JARID2 |
| RB1 | KCNH2 |
| ARID4A | KCNJ10 |
| REST | KCNJ14 |
| REV3L | KIF5B |
| RHO | KPNB1 |
| RNF6 | KRT7 |
| RP2 | LAMC2 |
| RPS6KA3 | LGALS3BP |
| RPS7 | LRP6 |
| SATB1 | MAP1B |
| CCL1 | MAZ |
| CCL20 | MEF2D |
| TRAPPC2 | ATXN3 |
| SET | ABCC1 |
| SGCB | MSH3 |
| ST6GAL1 | MYC |
| SKP2 | MYLK |
| SLC5A3 | NBL1 |
| SMARCA4 | NDUFA4 |
| SMN1 | NFYA |
| SOD3 | NRAS |
| SOX2 | ROR1 |
| SOX5 | OAS2 |
| SP1 | PA2G4 |
| SRPK2 | PRDX1 |
| SSFA2 | PAK1 |
| STAT3 | PAPPA |
| ELOVL4 | PAX6 |
| TAF1 | PFN2 |
| TAF5 | SERPINB5 |
| TAP1 | PIGH |
| TCF21 | PIK3CB |
| TGFB1 | APLP2 |
| TGFB2 | ARF4 |
| TGFBI | ARG1 |
| TGFBR2 | ATP1B3 |
| TGFBR3 | AUP1 |
| TGIF1 | BAX |
| TIAM1 | BCAT1 |
| TIMP3 | BMPR2 |
| TLR3 | CALM3 |
| TLR4 | CALU |
| TNFAIP3 | CAMK2D |
| TOP2A | CANX |
| TP53BP2 | CAPN2 |
| TPM1 | CAPZA1 |
| NR2C2 | CAPZB |
| TSNAX | CASP9 |
| UBE2N | CAV1 |
| UQCRB | CBS |
| UTRN | CCNE1 |
| VEGFA | CCNT2 |
| VHL | SCARB2 |
| CORO2A | ENTPD6 |
| WFS1 | CDC25B |
| NSD2 | CHRNA2 |
| WNT5A | ERCC8 |
| ZNF35 | CKS2 |
| PCGF2 | CLK3 |
| ZMYM2 | TPP1 |
| ZNF207 | CLN3 |
| ZNF217 | CNN3 |
| BTG2 | CRKL |
| ALMS1 | DCTD |
| RNF103 | DEFA5 |
| SLMAP | DHCR7 |
| USP7 | SLC26A3 |
| EPM2A | DSC2 |
| KAT6A | DTYMK |
| CDK2AP1 | E2F2 |
| ANP32A | PHC2 |
| GDF5 | EGFR |
| NCOA3 | EIF2S3 |
| SMC1A | MRAS |
| FZD6 | ATF5 |
| RECK | ZNF507 |
| PPFIA4 | ZNF365 |
| CBX4 | SIRT2 |
| PLPP1 | MYH15 |
| TP63 | SETD1B |
| SOCS1 | PPRC1 |
| B3GALNT1 | TBC1D2B |
| TNFRSF10B | TNRC6B |
| PER3 | RAD54L2 |
| PER2 | TTLL12 |
| FUBP1 | ZCCHC14 |
| BCL10 | ZC3H4 |
| PLOD3 | RPRD2 |
| BAZ1B | PPIP5K2 |
| SEMA5A | ICOSLG |
| LATS1 | ARHGEF12 |
| SCAF11 | SRGAP2 |
| LRRFIP1 | NUDCD3 |
| SRSF11 | EXOSC2 |
| SOCS6 | CBX7 |
| VPS26A | SNAPIN |
| CLOCK | CDC42EP4 |
| PREPL | RUSC1 |
| SOCS5 | FAM89B |
| SECISBP2L | KPNA6 |
| DOCK4 | HSPBP1 |
| USP34 | SH3BP4 |
| SLK | DCAF12 |
| PHACTR2 | TPGS2 |
| ZFYVE16 | ZNF385A |
| JADE3 | PNISR |
| CKAP5 | TSKU |
| SCRN1 | TMEM98 |
| EPM2AIP1 | CNTNAP2 |
| DDX46 | RAB11FIP5 |
| FAM20B | SZRD1 |
| LPGAT1 | PITPNC1 |
| HS3ST3B1 | LHX6 |
| DMTF1 | CNNM4 |
| PTBP3 | MYEOV |
| GNE | CHORDC1 |
| DNM1L | AP3M1 |
| EDIL3 | RNF11 |
| ACTR2 | LYPD3 |
| PPIF | CACNG5 |
| HIPK3 | EIF2AK1 |
| RASGRP1 | INTU |
| AKAP9 | VPS4A |
| ADGRG2 | SERP1 |
| TOPORS | LSM3 |
| MSLN | SULT1B1 |
| SPRY2 | CRCP |
| STUB1 | RBMS3 |
| SMNDC1 | MOCS3 |
| BTN3A3 | ANGPTL3 |
| PIAS3 | TMEM97 |
| BASP1 | GIT1 |
| TESK2 | DBNL |
| PGRMC2 | SSU72 |
| TMEM147 | UHRF1 |
| FAM3C | SERTAD3 |
| ECI2 | DNTTIP2 |
| TRIM38 | EHD3 |
| SLC9A6 | IL21R |
| IVNS1ABP | CRIM1 |
| EXOC5 | SHISA5 |
| NFAT5 | TMEM69 |
| YME1L1 | DNAJC27 |
| STAG2 | ZDHHC3 |
| PHTF1 | SLC25A37 |
| MAP3K2 | MS4A4A |
| ZNF460 | EIF3L |
| FRS2 | RWDD1 |
| FAXDC2 | UBE2D4 |
| MALT1 | SLC25A39 |
| SPIN1 | LUC7L2 |
| FERMT2 | ASB2 |
| WWP1 | C11orf24 |
| CNTRL | POLE3 |
| DUSP10 | DNAJC10 |
| SEC63 | ARMCX6 |
| HPS5 | TRMT13 |
| FILIP1L | MIER2 |
| MGAT4A | NDFIP2 |
| COBLL1 | ARL15 |
| RAB11FIP2 | MINDY2 |
| CPEB3 | TMEM106B |
| RUFY3 | CRLS1 |
| BTBD3 | TET2 |
| SACM1L | UBE2R2 |
| KIFAP3 | DUSP23 |
| SIRT2 | SLC35A5 |
| NT5C2 | RBM23 |
| CEP152 | TMEM38B |
| LIMCH1 | VPS13D |
| DAAM1 | GOLPH3L |
| PALLD | PHF10 |
| ZNF292 | C5orf22 |
| MON2 | LRRC59 |
| FNBP1 | YOD1 |
| SETD1B | KIF16B |
| ERP44 | PACS1 |
| MYCBP2 | NDC1 |
| CDK19 | DNAJC11 |
| TNRC6B | AGK |
| HIC2 | RNF114 |
| GPD1L | NXT2 |
| ATP11B | RCC2 |
| SYNE2 | C20orf24 |
| VPS13A | PDGFC |
| ANKRD28 | SERTAD4 |
| RPRD2 | MEPCE |
| DDHD2 | BCCIP |
| MGA | EIF5A2 |
| ATMIN | POLE4 |
| ICOSLG | KCNK13 |
| TRIM2 | JPH1 |
| SASH1 | UBQLN4 |
| WDR7 | PMEPA1 |
| DNAJC16 | PRDM8 |
| ARHGEF12 | PARP11 |
| NCSTN | NIPAL3 |
| ADNP | MIF4GD |
| DICER1 | REXO1 |
| RHOQ | KIAA1143 |
| ISCU | GATAD2B |
| SUZ12 | PLEKHH1 |
| MORC3 | USP31 |
| TTC33 | NCEH1 |
| PIGN | POGK |
| DDAH1 | GRAMD1A |
| RABGAP1 | CTDSP1 |
| TMEM2 | PLEKHB1 |
| SGK3 | CACNG7 |
| CADM1 | SAMSN1 |
| TMEM245 | DUS1L |
| RASGRP3 | RBSN |
| ATXN10 | NSD1 |
| FBXL2 | ZNF106 |
| HECTD1 | MPP5 |
| NIPBL | CRTC3 |
| C20orf194 | FNDC4 |
| MOXD1 | MARCKSL1 |
| SPATS2L | UBE2Z |
| OSBPL3 | TRIR |
| AUTS2 | ZNF655 |
| APPL1 | TSEN34 |
| RAI14 | ATG9A |
| EDRF1 | SLC25A23 |
| WSB1 | TMUB2 |
| GAPVD1 | EFHD2 |
| ZBTB20 | TMEM43 |
| FBXO3 | ZNF557 |
| SLC17A5 | FYCO1 |
| NBEA | SLC52A2 |
| AP3M1 | RNF128 |
| RNF11 | RHBDF2 |
| AGO2 | HECTD3 |
| SESN1 | ALG9 |
| PDCD4 | HPS6 |
| SETD2 | DOCK5 |
| DSE | NRSN2 |
| PURG | SEMA6D |
| ITSN2 | ATF7IP2 |
| DCAF8 | DCAF17 |
| MYEF2 | TMEM134 |
| FOXP3 | OPA3 |
| VPS36 | DNAJC5 |
| PHF20L1 | ZNF436 |
| RDH11 | SETD7 |
| NIN | COLEC12 |
| PHF20 | C1orf21 |
| UBR5 | TRIM8 |
| REV1 | TSC22D4 |
| LARS | GLT8D2 |
| VPS54 | BCL2L12 |
| ARMCX3 | C19orf12 |
| TRIM33 | ARMC10 |
| RAPGEF6 | TM2D2 |
| RSF1 | STK40 |
| FGFRL1 | CHCHD5 |
| ATAD2B | LLPH |
| MTMR12 | DDI2 |
| SGTB | MFSD14B |
| EGLN1 | MFSD9 |
| ZRANB1 | LMNB2 |
| RNF111 | RIOX2 |
| KLHL24 | ADO |
| AFTPH | FAM136A |
| TRPM7 | CORO6 |
| SNRK | MFSD5 |
| NSUN2 | RBM17 |
| NSD3 | KRTAP4-2 |
| PIGX | MICALL1 |
| PRPF39 | ZIC5 |
| PHIP | CCDC65 |
| USP47 | NAV1 |
| MED9 | RSPRY1 |
| RALGPS2 | ZNF625 |
| AGGF1 | TRIM47 |
| FIGN | PCED1B |
| CENPQ | RPS19BP1 |
| MRPS10 | SLFN11 |
| PBRM1 | SNX29 |
| KIAA1551 | TIMM50 |
| ZNF532 | FAM114A1 |
| FANCI | PRDM6 |
| SPTLC3 | IGSF8 |
| LIN7C | ZFAND4 |
| STRBP | TJAP1 |
| YOD1 | ERI2 |
| AP1AR | NACC1 |
| ETNK1 | TEX261 |
| FAM46A | OSBPL8 |
| DOCK10 | OSBPL11 |
| KANSL3 | MAS1L |
| POLR3B | RFFL |
| BTBD7 | SP7 |
| ATF7IP | ACOT4 |
| ENAH | CANT1 |
| DCP1A | LSM12 |
| PAG1 | MRPL10 |
| TMX4 | TANGO2 |
| SAR1A | EMID1 |
| JPH1 | TAF8 |
| UGGT1 | ENPP6 |
| TM9SF3 | MPLKIP |
| BDH2 | UBXN2B |
| KNL1 | RNF183 |
| PITHD1 | SPTY2D1 |
| RTN4 | KRT80 |
| PELI1 | ZNF555 |
| THOC2 | COMMD7 |
| RAB22A | DUSP18 |
| ESYT2 | BTLA |
| NUFIP2 | RNF38 |
| MIB1 | C8orf37 |
| KLHL42 | SLC35G1 |
| ARHGAP21 | SDE2 |
| SLAIN2 | SPTSSB |
| TSHZ3 | ASXL1 |
| GPAM | FAM9C |
| DDX55 | LDHD |
| PLEKHA1 | CASP16P |
| PLEKHA2 | ALG14 |
| GNB4 | SLC39A11 |
| LDAH | HTRA4 |
| GAS5 | CYB561A3 |
| ZNF667 | CPNE2 |
| FAM217B | UNC5CL |
| MOAP1 | ANKRD31 |
| RRAGC | BCL9L |
| NCAPG | CHAMP1 |
| HERPUD2 | C17orf78 |
| LMBR1 | FAM126B |
| MPP5 | C3orf38 |
| TNS3 | PRRT3 |
| CCDC14 | CAGE1 |
| RMND5A | POTED |
| FBXL17 | KRTAP11-1 |
| RAPH1 | IFNE |
| SOWAHC | CLEC4G |
| WNK1 | GLDN |
| WNK3 | KIF24 |
| MTMR9 | LCE1B |
| GID4 | LCE1E |
| DERL1 | ANKRD36 |
| DCAF10 | PTAR1 |
| CEP97 | PNPLA7 |
| ZYG11B | KMT5A |
| TBL1XR1 | TMEM81 |
| CLIP4 | ZNF805 |
| VASH2 | C10orf105 |
| CYBRD1 | TMEM236 |
| JADE1 | RHOXF2B |
| ELOVL7 | SIGLEC14 |
| DOCK5 | KRTAP4-7 |
| WWC2 | SMIM9 |
| ASRGL1 | AKR7A2 |
| FBXO11 | RDH16 |
| SPG11 | PLPP3 |
| PROSER1 | IRS2 |
| NAA50 | VAMP8 |
| NUBPL | GBF1 |
| PDGFD | GPAA1 |
| KLHL15 | FADD |
| TET1 | SOCS2 |
| DCAF11 | CFLAR |
| LNPK | ST3GAL5 |
| APOLD1 | SQSTM1 |
| NETO2 | AP1M1 |
| SPRY4 | TRPA1 |
| PARP9 | HAP1 |
| B3GNT5 | UNC119 |
| KBTBD7 | XPR1 |
| RAB6C | ZBTB22 |
| TRAF7 | KLF4 |
| PYM1 | NREP |
| PLEKHA8 | GTF3C5 |
| FAM136A | CNOT8 |
| ZNF587 | SNAP29 |
| SERAC1 | ZNF264 |
| ZCCHC3 | LITAF |
| DOCK7 | MPDU1 |
| NAV3 | VPS26A |
| KBTBD6 | TCL1B |
| RSPRY1 | SLC25A44 |
| LYRM7 | UBE3C |
| CCDC34 | TRAM2 |
| SESTD1 | SECISBP2L |
| RNF185 | SUSD6 |
| ACBD5 | JADE3 |
| BOC | MATR3 |
| FDXACB1 | SERTAD2 |
| ZBTB47 | DAZAP2 |
| SFXN1 | KEAP1 |
| MRAP2 | SPATA2 |
| GLCCI1 | TRIM14 |
| TUBGCP5 | GINS1 |
| OSBPL1A | AKT3 |
| COX20 | GJC1 |
| SLC16A10 | SCAMP2 |
| RFFL | PPIF |
| SPPL3 | WASF2 |
| SOCS4 | SLC25A15 |
| NAA30 | RNF41 |
| TMEM170A | ALG3 |
| TPRG1L | MFSD10 |
| TCEANC2 | DCAF7 |
| OSR1 | SIGMAR1 |
| UBR3 | BCKDK |
| AHSA2 | LANCL1 |
| PRRC1 | HMGN4 |
| JMY | CRTAP |
| GRPEL2 | FBLN5 |
| STXBP5 | IPO7 |
| PM20D2 | AGPAT1 |
| MTPN | TGOLN2 |
| PTPDC1 | PNMA2 |
| SREK1 | ZNF460 |
| TMEM56 | FRS2 |
| EXOC8 | ZNF275 |
| DTX3L | EHD1 |
| PPM1L | TMED2 |
| SNRNP48 | CKAP4 |
| ANKRD46 | TMED10 |
| RASEF | RAB32 |
| TOR1AIP2 | CDC37 |
| LONRF2 | WDR45 |
| PRICKLE2 | FICD |
| AGO4 | AKAP11 |
| ZNF367 | CHP1 |
| OTUD1 | LYPLA2 |
| FBXL13 | TUSC2 |
| TXLNGY | EXOC3 |
| PRR14L | MGLL |
| ZBTB38 | RRAS2 |
| CERS6 | SURF6 |
| LCORL | TAF1 |
| CASC2 | TAF4 |
| LRRC57 | TAGLN |
| PAN3 | TAPBP |
| GK5 | TCOF1 |
| ZADH2 | PPP1R11 |
| ZNF326 | TFPI |
| FAM126B | TFRC |
| CYP4V2 | TGM2 |
| TRIM59 | TLR4 |
| TICAM2 | TNFAIP2 |
| PTAR1 | TNP1 |
| C15orf52 | TOP2A |
| SAMD5 | TSN |
| PATE2 | UBE2A |
| SNX30 | NR1H2 |
| C1orf147 | VDAC1 |
| CCT6P1 | WFS1 |
| ZBTB8A | WNT8B |
| GXYLT2 | XRCC2 |
| C8orf17 | XRCC5 |
| NAT1 | YY1 |
| ACP1 | ZP3 |
| ACTC1 | ZXDA |
| ACTL6A | ZYX |
| AMD1 | LUZP1 |
| ANXA11 | DNALI1 |
| ARF1 | CUL5 |
| ASGR2 | PTP4A2 |
| ASS1 | SSPN |
| ATOX1 | FXR1 |
| ATP2A2 | GAN |
| BAK1 | SLC7A5 |
| BMPR1A | AKAP1 |
| CAD | GDF5 |
| CAPZB | OR1A1 |
| CCNB1 | RGS5 |
| CDC5L | PIK3R3 |
| CDH1 | YARS |
| CDK4 | PIK3CG |
| CDKN1C | PLAGL2 |
| CLIC1 | PLEC |
| CLTA | PMP2 |
| COL1A2 | POLR2E |
| CTSE | PPP2R1B |
| CYP2B6 | MAPK9 |
| CYP2C19 | MAP2K2 |
| DCTN1 | THAP12 |
| DHX9 | PTK2 |
| DHFR | PTK7 |
| ATN1 | PURB |
| DSC2 | RAB3A |
| DUSP5 | RAF1 |
| EEF1A1 | RALA |
| EIF4B | REL |
| EIF4EBP1 | RELA |
| EIF4G2 | RPL15 |
| EIF5A | RPS3A |
| ELF4 | RSU1 |
| EMD | RYK |
| EP300 | ATXN1 |
| CLN8 | CXCL5 |
| ETFA | SDHC |
| EZH2 | SRSF1 |
| BPTF | SKP2 |
| FECH | SLC5A3 |
| FGF2 | SLC6A9 |
| FKBP1A | SLIT3 |
| GAPDH | SLPI |
| GATA6 | SMARCD1 |
| GGCX | SNCA |
| GM2A | SOD2 |
| GOLGA3 | CAPN15 |
| RAPGEF1 | SRM |
| GTF2E1 | SSX1 |
| H3F3B | STK11 |
| HIVEP1 | SULT1C2 |
| HMBS | PIK3CD |
| HNRNPA1 | SUPT4H1 |
| HOXA13 | KCNMA1 |
| HOXC8 | KIT |
| HSPA1B | IPO5 |
| HSPA8 | LAMB3 |
| HSP90AA1 | LASP1 |
| HYAL1 | FADS1 |
| RBPJ | LMNB1 |
| IL6ST | LNPEP |
| INCENP | LOXL2 |
| INSIG1 | TM4SF1 |
| ITGB1 | MARCKS |
| ITPR1 | MAFG |
| KCNC4 | MDM2 |
| KIF5B | MEA1 |
| RPSA | MEIS2 |
| LETM1 | MAP3K5 |
| CD180 | MFAP3 |
| MAP1B | CD99 |
| MBD1 | MID1 |
| DNAJB9 | MITF |
| MDM2 | MKI67 |
| NF2 | MAP3K9 |
| NFYB | MAP3K11 |
| CNOT2 | MBNL1 |
| CNOT4 | MMP2 |
| NOTCH2 | MMP16 |
| NPM1 | MNT |
| NRAS | MTR |
| YBX1 | MYO1D |
| NVL | MYO5B |
| PAWR | MYO10 |
| PAX9 | NDUFA6 |
| PCDH7 | NDUFS1 |
| PDGFRL | NEDD9 |
| PDK2 | NFATC3 |
| PHKA1 | NFE2L1 |
| PIK3CD | KMT2A |
| PPIC | MTOR |
| PPP1R3D | FSHR |
| NPY4R | GAPDH |
| PRKAR1A | NR6A1 |
| PRPS1 | GFPT1 |
| PSMB4 | GJA1 |
| TWF1 | GLG1 |
| PEX5 | GLI2 |
| RAD51 | GMDS |
| RBMS2 | GNAI2 |
| REL | GNAS |
| RGS3 | GNB1 |
| RNF4 | GOLGA3 |
| ROBO1 | GOLGB1 |
| RPL7A | GPM6A |
| RPL9 | GSPT1 |
| RPL24 | H2AFX |
| RPL26 | HDGF |
| RPS3A | HLA-A |
| RPS14 | HLA-E |
| RPS28 | HLCS |
| RYR2 | HMGB1 |
| TSPAN31 | HMGA1 |
| MAP2K4 | HMOX1 |
| SLC12A4 | HNRNPA1 |
| SNRPD1 | HSPG2 |
| SNTB2 | FOXN2 |
| SOX4 | IFNAR2 |
| SOX11 | IGF2R |
| SREBF1 | IKBKB |
| SRPRA | IMPDH2 |
| SSRP1 | INHBA |
| STAT2 | EIF3E |
| AURKA | ITGAV |
| STXBP1 | ITGB1 |
| ELOA | ITPK1 |
| TEF | SNAI2 |
| TLN1 | SMARCB1 |
| TMF1 | SMO |
| TP53 | SNRPD3 |
| UGDH | SNTB1 |
| UCK2 | SP1 |
| UQCRFS1 | SP3 |
| EZR | SP100 |
| XPC | SREBF2 |
| ZNF24 | SSR1 |
| ZNF75A | SS18 |
| ZNF134 | STAU1 |
| ZNF157 | ADAM17 |
| PRRC2A | MLX |
| TUSC3 | TMBIM6 |
| HMGA2 | TERF2 |
| HIST2H2AA3 | TFDP2 |
| NSMAF | TFF1 |
| SMARCA5 | TGFBR3 |
| GEMIN2 | TLN1 |
| IFITM1 | TPM3 |
| COPS3 | TPR |
| CHST1 | HSP90B1 |
| RNGTT | TRPS1 |
| TNFSF10 | UBE2D3 |
| KAT2B | UBE2G2 |
| CDK5R1 | UBE2H |
| WASL | UBE2V1 |
| CCNE2 | UBP1 |
| OSMR | UCHL1 |
| SLC33A1 | UGP2 |
| GCNT3 | UCK2 |
| CYTH2 | KDM6A |
| KLF4 | VAV2 |
| TCEAL1 | SLC11A2 |
| RPL23 | NSF |
| DDX23 | ODC1 |
| ZNF264 | PAK2 |
| MED7 | PAM |
| ITM2B | PAWR |
| HAND2 | PCBP2 |
| KIF23 | PDE6D |
| SLC4A7 | PDGFRA |
| VPS4B | SERPINB6 |
| RAB3D | PIK3C2B |
| BZW1 | PI4KA |
| EDEM1 | PIP4K2A |
| TRAM2 | PKM |
| SNPH | PLS3 |
| ZSCAN12 | POLR2A |
| MLEC | POU2F2 |
| C2CD5 | PPARD |
| UBAP2L | PPARG |
| AP5Z1 | PPIA |
| SUPT7L | PPP1CC |
| GFPT2 | PPP2CB |
| TANK | PPP2R2A |
| BCL2L11 | PPP2R5E |
| SH2B3 | PPP6C |
| ABCF2 | PRKAG1 |
| ARPC3 | PRKAR1B |
| TSFM | PRKCA |
| TRAP1 | PRKDC |
| TOB1 | PRKG1 |
| CNIH1 | MAP2K3 |
| LHFPL2 | MAP2K7 |
| EIF1 | PRLR |
| CTDSPL | PSMB5 |
| AP3S2 | PTCH1 |
| DENND4A | PTPN11 |
| STAG1 | PTPN14 |
| KATNB1 | PTPRG |
| ZNF267 | PTPRK |
| CCL26 | NECTIN2 |
| TUBB4B | RARA |
| PRMT5 | RBM4 |
| SEMA4D | RET |
| DEAF1 | REV3L |
| RNASEH2A | RFC1 |
| PRPF8 | RFX5 |
| PAIP1 | RFXAP |
| CD226 | RIT1 |
| POLQ | RNF4 |
| WDR4 | ROBO1 |
| PGRMC1 | RORA |
| LILRA2 | RPL3 |
| PAPD7 | RPL8 |
| TPPP | RPL12 |
| TBC1D8 | RPL21 |
| ZNF277 | RPS6KA3 |
| SUPT16H | RPS6KB1 |
| AKAP10 | RPS13 |
| B4GALT7 | RPS18 |
| FKBP9 | RPS25 |
| PHB2 | SORT1 |
| RNF44 | S100A11 |
| SBNO2 | SATB1 |
| KLHDC10 | SFPQ |
| GOLGA8A | SFRP2 |
| PHLPP2 | SFSWAP |
| MCF2L2 | SH3GL1 |
| GRAMD4 | SHMT1 |
| CIC | SIAH2 |
| RCOR1 | ST6GAL1 |
| UBXN4 | ST3GAL2 |
| EXOC6B | SLC1A5 |
| DENND5A | SLC20A2 |
| PIP5K1C | ABL1 |
| SLC39A14 | ACACA |
| CABIN1 | ACTB |
| SRRM2 | ACTN1 |
| RBFOX2 | ADARB1 |
| ZNF346 | ADH5 |
| SLC7A11 | PARP4 |
| TMEM184B | AP2A2 |
| NDUFAF3 | ANK3 |
| ZDHHC5 | ANXA2 |
| SZRD1 | APC |
| TRAF3IP1 | BIRC5 |
| FBXW2 | SHROOM2 |
| NARF | RHOA |
| CNNM4 | RHOB |
| AGO1 | ARNT |
| LATS2 | ATM |
| RANBP6 | ATP1B1 |
| PELP1 | ATP2B4 |
| DKK4 | ATP5B |
| TUBGCP4 | ATP6AP1 |
| TOR1B | ATR |
| CCDC113 | BCL2L1 |
| C1GALT1C1 | BCL9 |
| MYLIP | BMI1 |
| ZDHHC8 | BMP1 |
| SERTAD3 | DST |
| BAZ2B | KLF9 |
| TNPO2 | VPS51 |
| HP1BP3 | RUNX2 |
| F11R | RUNX3 |
| MRPS16 | CBL |
| TVP23B | CCND3 |
| RLIM | CCNF |
| ERGIC2 | CD44 |
| NRN1 | CDH2 |
| GULP1 | CDK6 |
| MPP6 | CDKN1B |
| GALNT7 | CDKN2C |
| C11orf24 | CHD2 |
| PELO | CHKB |
| CHRAC1 | COL4A1 |
| RBM27 | COX15 |
| MRPS21 | CPOX |
| CCSER2 | CREBBP |
| ANKIB1 | VCAN |
| PLEKHA5 | CTNNA1 |
| DDIT4 | CTSB |
| DDX49 | CUX1 |
| RSBN1 | CYP24A1 |
| RRN3 | DAPK1 |
| NDE1 | CYB5R3 |
| ESRP1 | DLG1 |
| PGPEP1 | DNMT1 |
| TOR4A | DOK1 |
| UHRF1BP1 | DPYSL3 |
| NCAPG2 | EEF1D |
| MTMR10 | EEF1G |
| LAX1 | EFNA1 |
| SEMA4C | EFNB2 |
| DUS2 | EIF2B1 |
| CCDC186 | EIF4A2 |
| ARHGAP17 | EIF4G1 |
| RBM28 | ELAVL1 |
| TMEM33 | EPHB2 |
| SLC25A36 | ETS2 |
| SMU1 | EXT1 |
| FBXW7 | FAT1 |
| PARL | FBN2 |
| TRIM36 | FKBP1A |
| PLXNA3 | FOXO3 |
| CNOT11 | FLNB |
| MED29 | FN1 |
| G2E3 | PRRC2C |
| NHP2 | NUP210 |
| WDR11 | SIK2 |
| NSMCE3 | CAMTA1 |
| CTNNBL1 | CLEC16A |
| EIF5A2 | SATB2 |
| PNO1 | USP22 |
| PMEPA1 | DOCK9 |
| ARNTL2 | PSD3 |
| MFF | PUM2 |
| OTUD7B | DICER1 |
| TULP4 | RHOQ |
| PDXP | ICMT |
| ZNF695 | ZFYVE26 |
| CYP20A1 | CLCF1 |
| GATAD2B | TTC33 |
| SCAF4 | DNPEP |
| SLC12A5 | CHST5 |
| ARID1B | DDX58 |
| CASKIN1 | CORO1C |
| USP28 | SH3BP1 |
| KIAA1586 | SLC7A11 |
| ZNF317 | NPAP1 |
| GPBP1L1 | TFIP11 |
| AGBL5 | SUMF2 |
| GUF1 | POLR1A |
| SPCS3 | RCHY1 |
| SMAP1 | MTHFD1L |
| CIDEC | SIN3A |
| CLSPN | KBTBD2 |
| C17orf75 | REXO2 |
| CPEB1 | L3MBTL1 |
| NUCKS1 | LRIG1 |
| SMAP2 | GLCE |
| FNDC3B | SETBP1 |
| AEN | ZNF500 |
| CEP85 | AUTS2 |
| BCL11B | RAI14 |
| GPBP1 | PHF19 |
| UBE2Z | RSL1D1 |
| ZFYVE21 | INTS1 |
| KCTD15 | FBXW2 |
| PRRG4 | FBXL5 |
| NOL12 | FBXL4 |
| C1orf35 | BLOC1S6 |
| ZNF557 | SACS |
| OR2A4 | TIMM10B |
| NKAP | AGO1 |
| TXNDC15 | DAZAP1 |
| AGMAT | DNAJC2 |
| LONRF3 | NPTN |
| TRMT2B | GHITM |
| GPR157 | FOXP1 |
| PTGES2 | TAF5L |
| CTC1 | DKK2 |
| OPA3 | SALL3 |
| ORAI2 | PRELID1 |
| CPEB4 | PDCD4 |
| SLC25A32 | CSDC2 |
| FAM49A | RAB30 |
| ANP32E | PGAP2 |
| VMP1 | POLM |
| ARPC5L | C11orf54 |
| TMUB1 | TMEM14A |
| MRO | HIPK2 |
| PCBD2 | MRPL13 |
| ZNRF3 | BRD7 |
| KIAA1109 | STRN4 |
| YIPF4 | CPSF1 |
| DDI2 | SH3KBP1 |
| LCOR | ZNF354C |
| SLX4 | SCAPER |
| ACSS1 | PNPLA8 |
| PARD6B | DCAF8 |
| CARD6 | HP1BP3 |
| PRRC2B | SOST |
| ZNF607 | SLC35C2 |
| ZIC5 | NOSIP |
| TMEM41A | MRPS2 |
| ZNF598 | IER3IP1 |
| ZNF682 | CHCHD2 |
| PKDCC | SDF4 |
| ZC3HAV1L | LEF1 |
| DPH7 | PAIP2 |
| ADAMTSL1 | FAM13B |
| TMEM44 | FAM198B |
| FNIP1 | AIG1 |
| TIRAP | ANKFY1 |
| PCMTD1 | LARS |
| TNFRSF13C | OSER1 |
| FAM129A | SIRT6 |
| ZNF354B | GDE1 |
| PDZD8 | SRRT |
| FOPNL | ATP6V1H |
| WDR81 | GPRC5B |
| LOXHD1 | RSF1 |
| EID2B | SSH1 |
| IRGQ | PLEKHA5 |
| SHE | RSBN1 |
| TATDN3 | CNNM2 |
| TAF8 | AHI1 |
| SYNPR | FAM83E |
| C5orf24 | QRICH1 |
| SESN3 | MTMR10 |
| ZNF417 | RPP25 |
| ENTHD1 | PARP16 |
| SRFBP1 | C1orf159 |
| KIAA1958 | AURKAIP1 |
| ZNF383 | PHIP |
| DENND2C | USP47 |
| FUT11 | SUSD4 |
| ANKRD24 | ARHGAP17 |
| AAED1 | HEATR1 |
| ZFC3H1 | UBR7 |
| UBR1 | MTPAP |
| EME2 | RFWD3 |
| DAND5 | MRPS18A |
| ALG14 | SLC25A36 |
| TXLNA | SBNO1 |
| APOBEC3F | VPS53 |
| FLCN | UBE2W |
| TUBB | BRF2 |
| CCDC171 | AGPAT5 |
| VMA21 | SLC39A9 |
| MED19 | HJURP |
| ANKRD18A | LGR4 |
| ZDHHC24 | TMCO6 |
| MCOLN2 | DNAJA4 |
| CNEP1R1 | ETNK1 |
| CCDC96 | CDKN2AIP |
| SPRYD4 | ANKRD10 |
| CEP170B | DOCK10 |
| GOLGA8IP | THUMPD1 |
| PPP1R37 | ZNF407 |
| NLRP9 | BTBD2 |
| ZBTB8OS | NHP2 |
| TMEM173 | BCAS4 |
| ZDHHC21 | NPLOC4 |
| FMN1 | IWS1 |
| ZNF850 | FRMD4A |
| XKR7 | BTBD7 |
| NUP43 | COA1 |
| BMP8A | CNDP2 |
| NUGGC | UGGT2 |
| VHLL | UBAP2 |
| MUC21 | HDAC8 |
| ZNF772 | LANCL2 |
| CHCHD10 | SULF2 |
| ZBTB34 | FAM214A |
| H3F3C | PARD3 |
| GOLGA8B | DIABLO |
| HIST2H2BF | TRIM39 |
| PGAM4 | EMC7 |
| ARGFX | SPIRE1 |
| ZFP62 | SEMA3G |
| GOLGA8J | OTUD7B |
| ZBTB8B | KIF15 |
| TMEM239 | RARS2 |
| C15orf38-AP3S2 | CASS4 |
| MYZAP | SLC39A10 |
| ACLY | THOC2 |
| ACTG1 | KIAA1147 |
| ACVR2A | NHSL1 |
| ADRA1B | LYRM2 |
| APLNR | CBX8 |
| AHCY | NMRAL1 |
| AKT1 | BIRC6 |
| SLC25A5 | MYORG |
| ANXA7 | RIMKLB |
| XIAP | ZNF608 |
| APOC3 | NUFIP2 |
| ASCL2 | CFAP97 |
| ATIC | SLAIN2 |
| AXL | JCAD |
| CD3D | ZFAT |
| CDC6 | KIAA1522 |
| CDK2 | KIAA1549 |
| CDKN1A | CHD8 |
| CDKN2A | ZNF317 |
| CLPTM1 | TRAPPC1 |
| COL6A2 | PRUNE1 |
| CREB1 | HIVEP3 |
| CRK | GNB4 |
| CRY2 | GPBP1L1 |
| CSNK2A1 | FKBP10 |
| CTBP1 | MANBAL |
| DIAPH2 | BLOC1S5 |
| DLAT | FAM217B |
| DLX1 | ATL2 |
| DMWD | VEGFC |
| DVL3 | WARS |
| EXTL3 | CLIP2 |
| FASN | WNT5A |
| FHL2 | YWHAB |
| FUT1 | ZFX |
| FUT6 | ZNF3 |
| FZD2 | ZNF711 |
| GATA3 | ZNF41 |
| NIPSNAP2 | VEZF1 |
| GK | PTP4A1 |
| GP1BA | ALMS1 |
| GRK6 | REEP5 |
| GRINA | PRRC2A |
| GSR | BAG6 |
| GSS | NUP214 |
| HIVEP2 | CCDC6 |
| HK1 | NCOA4 |
| HLA-E | SHOC2 |
| HMGN2 | ADAM12 |
| HNRNPF | KMT2D |
| HOXB3 | AAAS |
| HOXD1 | ARID1A |
| HOXD10 | AXIN1 |
| HOXD11 | BAP1 |
| ID4 | PIP4K2B |
| IGSF1 | LTBP4 |
| INHBA | STK24 |
| STT3A | CUL3 |
| LAMC1 | CDC42BPA |
| LRP3 | RANBP3 |
| LSS | PKP4 |
| LYL1 | DGKZ |
| ND6 | COPS3 |
| MYF5 | EIF4G3 |
| NAP1L1 | SRSF9 |
| NDUFB5 | GALNT4 |
| NF1 | SNX4 |
| NOTCH1 | CTNNAL1 |
| NPTX1 | RNMT |
| SLC11A2 | PABPC4 |
| ORC1 | DCAF5 |
| PAFAH1B1 | DPM2 |
| PAX6 | NRP1 |
| PDK3 | KSR1 |
| PFAS | ASAP2 |
| PIK3CA | FUBP1 |
| POLR2A | MCM3AP |
| POU2F2 | EIF2B5 |
| PPP3CB | BUD31 |
| PSMD11 | MTMR3 |
| PSMD13 | ATP6V0E1 |
| RARG | SEMA5A |
| DPF2 | SPAG9 |
| RNF2 | MTMR4 |
| RORA | COX7A2L |
| RPS8 | LRRFIP1 |
| RPS9 | MSC |
| RPS15A | TSPOAP1 |
| SDC1 | PDLIM7 |
| SFRP1 | COPS2 |
| SRSF1 | EFTUD2 |
| TRA2B | SLIT2 |
| SLC2A3 | TGFBRAP1 |
| SLC5A5 | FADS2 |
| SON | KCNK6 |
| SRP19 | PMPCB |
| STAT6 | PDIA4 |
| SURF6 | SDC3 |
| ZEB1 | TTI1 |
| TCF15 | NUP93 |
| TCF19 | USP34 |
| TFAP2C | IST1 |
| TFPI | SCRN1 |
| TPM4 | GIT2 |
| UROD | ZBTB24 |
| ZNF708 | GAB2 |
| ZNF223 | POM121 |
| NR4A3 | KLHL21 |
| CHAF1B | SEC16A |
| USP9X | RNF10 |
| HIST1H2AL | MFN2 |
| PPFIBP1 | WDR1 |
| API5 | DMTF1 |
| MAPKAPK5 | SRA1 |
| DGAT1 | PDCD6 |
| SNX4 | HMGXB4 |
| NRP2 | ABCF2 |
| USP6 | ARPC1B |
| S1PR2 | ACTR2 |
| KL | PDIA6 |
| CRIPT | NAMPT |
| FXR2 | TOB1 |
| H6PD | MBNL2 |
| NCOR2 | RASA4 |
| RAPGEF2 | RBM6 |
| KIAA0100 | RBM5 |
| USP6NL | OLIG2 |
| SART3 | STAM2 |
| ATG13 | AKAP8 |
| PIEZO1 | BCAS2 |
| RGP1 | CAP1 |
| USP3 | VAT1 |
| CCS | SEMA4D |
| FEM1B | DDX17 |
| FAM13A | DEAF1 |
| MBNL2 | IPO8 |
| TUBA1B | PITRM1 |
| HTATIP2 | PRPF8 |
| ARFGEF1 | CDC42EP3 |
| PAICS | ERLIN1 |
| ERLIN1 | EBP |
| ZMYND11 | RBBP9 |
| RALBP1 | HBS1L |
| MSL3 | SRSF10 |
| EBNA1BP2 | ARPP19 |
| COPS5 | SEC24A |
| PTPRT | WASF3 |
| NUDT4 | BLCAP |
| SYNPO | RNPS1 |
| CARD8 | CBX1 |
| MAPRE1 | SERINC3 |
| SCMH1 | STIP1 |
| XPO7 | FERMT2 |
| NCOA6 | PAPD7 |
| SYT11 | ABHD2 |
| PRRC2C | GLMN |
| FAM208A | NUDT5 |
| ESYT1 | WDHD1 |
| SMCHD1 | SUPT16H |
| PPP1R13B | RPL35 |
| AHCYL2 | KLF12 |
| EXOSC2 | PUF60 |
| ATP1B4 | ZNF652 |
| PSD4 | SEC31A |
| DDX58 | MORC2 |
| LEMD3 | CLSTN1 |
| ZNF318 | ARSG |
| QPCT | SEPHS1 |
| METTL7A | ELL2 |
| POC1A | ACIN1 |
| IPCEF1 | CNOT1 |
| UBXN7 | PALLD |
| PABPC1 | KIF21B |
| ACAD8 | ZNF609 |
| MCTS1 | AVL9 |
| UHRF1 | ZC3H13 |
| TMED5 | SARM1 |
| GLOD4 | MRPS27 |
| RSRC1 | ZZEF1 |
| CRLF3 | MPRIP |
| SPOUT1 | LARP4B |
| CMPK1 | PSME4 |
| CDK12 | ARL6IP1 |
| PHAX | SYT11 |
| CHPF2 | TBC1D3C |
| PRMT7 | CCDC88C |
| ALKBH4 | NAMPTP1 |
| FAM118A | CDK11A |
| MSTO1 | SLC35E2B |
| KLHDC8A | ERVMER34-1 |
| CHDH | POC1B-GALNT4 |
| LRRC59 | IMMP2L |
| NCBP3 | FAM172A |
| FEM1A | KATNAL1 |
| KIF21A | TMEM222 |
| THUMPD1 | SLC10A7 |
| SLC48A1 | ANKRD27 |
| TMEM127 | ARL6 |
| URGCP | NLRC5 |
| TENM3 | RHBDD1 |
| HHAT | TMEM101 |
| CSGALNACT1 | CARD11 |
| MBNL3 | MAML2 |
| ACOT13 | MAP3K21 |
| GPCPD1 | ZC3H8 |
| C1GALT1 | SRRM4 |
| CHMP1B | MRPL43 |
| KIAA1143 | KIRREL3 |
| CNOT6 | TNRC18 |
| MAVS | DGAT2 |
| TXNDC16 | ZNF496 |
| CAMSAP3 | ATAD1 |
| XPNPEP3 | C9orf3 |
| MRPS14 | FAM104A |
| HHIP | ZNF566 |
| CSMD1 | HIST1H2BK |
| WDR13 | KIAA1644 |
| ZNF574 | TRIM5 |
| C6orf106 | SLC45A3 |
| LPIN3 | HPS4 |
| STK33 | TSPAN18 |
| TMEM109 | HS6ST2 |
| WDR77 | KLHL13 |
| ZNF329 | CCDC74A |
| FBXO31 | CREB3L1 |
| FOXRED2 | TMEM259 |
| CSRNP3 | BTF3L4 |
| C3orf36 | CCDC74B |
| TTYH3 | ELMSAN1 |
| SETD7 | RFT1 |
| NUAK2 | OXNAD1 |
| FAHD1 | RBM18 |
| CCDC8 | NAPRT |
| ZNF394 | ACTR8 |
| TMEM101 | FNIP1 |
| PPP1R15B | ZC3H18 |
| RBM17 | EARS2 |
| SAPCD2 | ZNF813 |
| LMF2 | EID2B |
| ZNF502 | IFFO2 |
| CENPL | PPP1R21 |
| TIMM50 | DIS3L2 |
| ARHGAP18 | MBOAT2 |
| GLB1L3 | ICA1L |
| BORCS5 | LYPD6B |
| OLFM3 | EMB |
| SLC24A4 | STARD4 |
| ZC3H18 | NANP |
| LIX1L | SREK1 |
| AASDH | HECTD2 |
| NEK7 | CACUL1 |
| ZNF280B | DNHD1 |
| KCTD11 | WDR66 |
| ARSK | HNRNPA1L2 |
| CNKSR3 | WIPF2 |
| RUNDC3B | RC3H1 |
| SLC25A43 | FBXO41 |
| RTKN2 | CCDC80 |
| C6orf89 | MB21D2 |
| TMED4 | TMPRSS3 |
| SLC25A30 | SMYD3 |
| PLA2G4F | FNDC3B |
| C17orf105 | BCL11B |
| TSPAN33 | SLC30A5 |
| MTX3 | CCDC71 |
| ZNF445 | TRAK2 |
| SFT2D2 | ELOVL6 |
| RAB15 | LBHD1 |
| RBM12B | ZSCAN5A |
| ONECUT3 | TNIP2 |
| PLA2G2C | CENPO |
| FRRS1 | THTPA |
| ANKRD33B | ZXDC |
| ZNF878 | RIPOR1 |
| MTRNR2L7 | SAP130 |
| MTRNR2L3 | RNF219 |
| MTRNR2L10 | PANK3 |
| MTRNR2L11 | C3orf52 |
| ABL2 | TTC21B |
| ACOX1 | METTL8 |
| ADORA3 | DCAKD |
| ADRA2B | KIAA0319L |
| AP2B1 | ERMP1 |
| ALDH3B1 | PHC3 |
| APP | GPR157 |
| APRT | SIKE1 |
| ABCC6 | NAA15 |
| ARCN1 | SLC35F5 |
| ARHGDIA | EDEM3 |
| ATP5G3 | EPC1 |
| BAX | TTYH3 |
| CCND1 | ZFP91 |
| BSG | SLC38A1 |
| CA8 | VOPP1 |
| CALU | MAGED4B |
| CANX | NIPA2 |
| CAPZA2 | ITM2C |
| RUNX1T1 | TSPAN14 |
| CBFA2T3 | CCNL2 |
| CCND2 | NUAK2 |
| CCND3 | RNF170 |
| CCNE1 | ABHD11 |
| CCNT1 | EIF2A |
| CCNT2 | TMEM64 |
| ENTPD1 | GLIS3 |
| ENTPD6 | ANO6 |
| CD44 | TRIM65 |
| CDK1 | DENND6A |
| CDC27 | UNC5B |
| CHEK1 | HNRNPA3 |
| AP2M1 | ZBTB9 |
| CLCN6 | PXDC1 |
| CPOX | FOXK1 |
| CREBL2 | CCZ1B |
| CRKL | ATXN7L1 |
| CSNK1E | RICTOR |
| CYBA | UBN2 |
| CYLD | AKNAD1 |
| CYP51A1 | TCP11L2 |
| DDX5 | ASPM |
| DECR1 | HARBI1 |
| DIAPH1 | CD163L1 |
| DMPK | ANKRD52 |
| DSG2 | ZNF776 |
| ECHS1 | FAM102B |
| EFNB2 | SNHG15 |
| EIF4A1 | XKR6 |
| ELK4 | MSL1 |
| EN2 | SPOPL |
| SLC29A1 | GALNT18 |
| EXT1 | TEX9 |
| EZH1 | SFT2D2 |
| FANCC | TMEM189 |
| FGF5 | TMEM189-UBE2V1 |
| FGFR4 | ZNF827 |
| FLOT2 | ABCA13 |
| FUT2 | RDH10 |
| GABPA | RASEF |
| GALNT1 | HFM1 |
| NR6A1 | DCP2 |
| B4GALT1 | C17orf102 |
| GNAL | KLHL31 |
| GNAT1 | ACADVL |
| GNB1 | ACAT2 |
| GPR27 | AARS |
| GRB2 | ACADL |
| GSK3B | ADCY3 |
| HDGF | ADCY6 |
| HMGA1 | ADPRH |
| HNRNPA2B1 | ADRB3 |
| HNRNPH3 | JAG1 |
| HOXA3 | AGTR1 |
| HOXA10 | AGTR2 |
| DNAJA1 | AHR |
| HSP90AB1 | CRYBG1 |
| IFNG | AK2 |
| IFNGR2 | AKT2 |
| INSR | ALDH9A1 |
| ITGA6 | ADD3 |
| IRF4 | ANXA5 |
| ITGA2 | ANXA6 |
| JAK1 | BIRC2 |
| JARID2 | AR |
| KDR | ARAF |
| KPNA1 | RHOC |
| KPNA3 | RHOG |
| KRT33B | ARHGAP1 |
| TM4SF1 | ARSD |
| SMAD2 | ALDOB |
| SMAD3 | ANPEP |
| MDH1 | HCN2 |
| AFDN | BDKRB1 |
| MLLT6 | BDNF |
| MPP1 | BMP6 |
| MTHFR | BMPR1A |
| MYO5A | ZFP36L1 |
| NAB1 | ZFP36L2 |
| HNRNPM | BTC |
| NFIC | C4BPB |
| OCRL | MRPL49 |
| FURIN | LDLRAD4 |
| PAFAH1B2 | PTTG1IP |
| PAK2 | CA12 |
| PCMT1 | S100G |
| CDK17 | CALR |
| PDCD1 | CAMP |
| PDE4D | CAPNS1 |
| PEX12 | CAST |
| PEX13 | SERPINH1 |
| PGD | CBR3 |
| PIK3C2B | CCND2 |
| PIM1 | CD1D |
| PLAG1 | CD86 |
| PLRG1 | CD59 |
| POLR2E | CD151 |
| POU2AF1 | CDC27 |
| POU2F1 | CDH11 |
| PPM1A | CDH12 |
| PPP1CB | CDK2 |
| PPP1CC | CDK4 |
| PPP2R5C | CDKN1A |
| PPP6C | AZF1 |
| PPT1 | BCL6 |
| PRKAA1 | DDX3X |
| PRKAR2A | DFFB |
| PRKCD | DHCR24 |
| MAPK1 | DKC1 |
| EIF2AK2 | DNAH5 |
| PSKH1 | DNM2 |
| PSMB5 | DNMT3B |
| PSMD7 | DPYD |
| PTBP1 | DRD4 |
| PTPRD | DRG2 |
| PTPRJ | RCAN1 |
| PXMP2 | DSG2 |
| QARS | TSC22D3 |
| RAB1A | SLC26A2 |
| MAP4K2 | DUSP2 |
| RAD23B | DVL2 |
| RARB | E2F4 |
| RBBP6 | E2F5 |
| RNH1 | E2F6 |
| RPL27A | ECE1 |
| RPLP0 | EDN1 |
| RPS3 | EFNB1 |
| RPS5 | EGR1 |
| RPS6 | EGR2 |
| RPS6KB1 | EPHA2 |
| RS1 | EIF4EBP1 |
| SALL1 | ELF3 |
| SKI | ELF4 |
| SLC1A5 | ELK3 |
| SLC9A1 | EML1 |
| SNCG | EMD |
| SNRPB2 | EMP1 |
| SRPK1 | SLC29A1 |
| STXBP3 | STOM |
| SYPL1 | STX2 |
| TAF13 | ERBB2 |
| MAP3K7 | EREG |
| TARBP2 | EYA4 |
| HNF1A | ERCC5 |
| TCF3 | ERF |
| TCP1 | EVI2A |
| PPP1R11 | EZH2 |
| TERF2 | F3 |
| TFAP2A | F13B |
| THRA | FECH |
| TLE4 | FGF5 |
| TLL1 | FGFR1 |
| TPD52 | FHL2 |
| TPM2 | FOXF2 |
| TPM3 | FLG |
| TTC1 | FLI1 |
| TUBB2A | FLII |
| UBE2H | FLOT2 |
| UBE2V1 | FMOD |
| UGT2B4 | FPGS |
| VAV2 | FXN |
| VCL | KDSR |
| VDAC2 | XRCC6 |
| WEE1 | GAS6 |
| YWHAH | GBP1 |
| ZNF91 | GCH1 |
| ZBTB16 | KAT2A |
| ZFAND5 | GDF10 |
| LUZP1 | B4GALT1 |
| CSDE1 | GNAI3 |
| MAFK | GNG10 |
| NUP214 | GNRHR |
| SHOC2 | GPC1 |
| KMT2D | GPM6B |
| SLC7A5 | GPX4 |
| COIL | CDKN2A |
| NRIP1 | CDO1 |
| LZTR1 | CEBPA |
| TKTL1 | CEBPE |
| AXIN2 | RCC1 |
| FZD9 | CLN5 |
| HIST2H2BE | CLTA |
| DYRK3 | CNP |
| CUL3 | COL1A1 |
| CUL2 | COL4A4 |
| SUPT3H | COL6A2 |
| OGT | COL8A2 |
| RAE1 | COL12A1 |
| PPM1D | COL13A1 |
| BHLHE40 | COL17A1 |
| DENR | KLF6 |
| CASK | SLC31A1 |
| SLC25A12 | SLC31A2 |
| PLPP3 | MAP3K8 |
| DDX3Y | CPM |
| RNMT | MAPK14 |
| TNFSF9 | CSPG4 |
| CDS2 | CSRP1 |
| FADD | CSTF3 |
| NAPG | CTGF |
| CREG1 | CTNS |
| SYNJ1 | CTNNB1 |
| EIF2B2 | CTNND1 |
| MTMR3 | CTSH |
| CPNE1 | CYB5A |
| MBD4 | CYP1B1 |
| BTRC | CD55 |
| ATP6V0E1 | DGKA |
| SOCS3 | DBT |
| BTAF1 | DCC |
| RPL14 | PLXNA2 |
| GPRC5A | PRRX1 |
| MTMR6 | PODXL |
| MTMR4 | POLA1 |
| LRRFIP2 | POLR2B |
| TBRG4 | POLR2J |
| MAPKAPK2 | POLR2L |
| GLP2R | PPARA |
| EFTUD2 | PRKCB |
| UBE4A | MAPK11 |
| PPIG | EIF2AK2 |
| KIF3B | PRPH |
| TM9SF2 | PRPS1 |
| LITAF | PSG3 |
| TBPL1 | PSG9 |
| GOSR1 | PSKH1 |
| BAG4 | PTBP1 |
| IER2 | PTGS1 |
| EIF5B | PTGS2 |
| N4BP1 | PTH2R |
| UBE3C | QSOX1 |
| RUBCN | PTPN9 |
| ARHGAP32 | PTPN12 |
| RASSF2 | PTPRB |
| RIMS3 | PTPRJ |
| MTSS1 | PTPRZ1 |
| PHYHIP | PXN |
| URB2 | ALDH18A1 |
| TSC22D2 | RAB2A |
| TRIM14 | RAB3IL1 |
| LRIG2 | RAB27A |
| TLK1 | RAC1 |
| TECPR2 | RAD51 |
| ZBTB5 | RAP2A |
| USP15 | RARG |
| THRAP3 | RBMS1 |
| HNRNPDL | DPF2 |
| AKT3 | REST |
| ZBTB33 | RFC3 |
| TSPAN3 | RFX1 |
| LRPPRC | RFX2 |
| PDIA6 | RGS4 |
| MPHOSPH9 | ROCK1 |
| PATJ | ROM1 |
| DSCR3 | RPL19 |
| LANCL1 | MRPS12 |
| LRRC41 | RPS15 |
| CARM1 | RRAS |
| HYOU1 | RYR3 |
| SPTLC1 | S100A2 |
| TGOLN2 | SC5D |
| TXNIP | SCN3A |
| DMRT2 | CCL2 |
| B3GNT2 | SDC4 |
| TBR1 | SECTM1 |
| NUP50 | SRSF3 |
| TOB2 | SRSF5 |
| NCKAP1 | SGK1 |
| SEC24A | SHC1 |
| ZNF275 | SHMT2 |
| PNPLA6 | SKIL |
| RNPS1 | SLC1A3 |
| PRDX3 | SLC1A4 |
| PRSS21 | SLC7A1 |
| RAB40B | SLC16A1 |
| YWHAQ | SLC22A3 |
| ABHD2 | SLC22A5 |
| PRDM4 | SMARCA2 |
| RCAN3 | SNAI1 |
| AP4S1 | SNRPB |
| BAZ2A | SNRPF |
| PLPBP | SNTA1 |
| AKAP11 | SNTB2 |
| SYNRG | SOS1 |
| STK38 | SOS2 |
| GABARAP | SOX9 |
| OIP5 | SPTA1 |
| HSPA4L | SRP9 |
| ATG14 | TROVE2 |
| MLXIP | SSFA2 |
| TRAK1 | STAT3 |
| DHX30 | STAT5A |
| AVL9 | SULT1E1 |
| TRIM35 | AURKA |
| TTLL5 | SURF4 |
| TAB2 | SYCP1 |
| GPATCH8 | SYPL1 |
| GGA3 | TARBP1 |
| ZCCHC14 | TBX2 |
| MESD | TBXA2R |
| KANK1 | TCF3 |
| GANAB | TCTA |
| NUP210 | TEAD1 |
| DNAJC9 | TFAP4 |
| PPIP5K2 | TGFB1I1 |
| CLUH | TGM1 |
| NUP160 | THPO |
| U2SURP | TIMP3 |
| SUN1 | ICAM5 |
| KIAA0895 | TLR3 |
| SIRT4 | TSPAN6 |
| TARDBP | TPD52L2 |
| SF3B3 | TPM1 |
| CBX6 | CCT3 |
| TRAM1 | TRPC1 |
| NNT | TRPC6 |
| TNPO3 | TUB |
| CD2AP | TYK2 |
| EDC4 | TYRP1 |
| TRIM29 | SLC35A2 |
| SH3BP4 | UMPS |
| GABARAPL1 | USH2A |
| PPIL2 | VDR |
| PISD | VIM |
| BAMBI | WIPF1 |
| ARIH1 | CORO2A |
| MOB4 | XBP1 |
| ANAPC13 | ZNF131 |
| RPL36 | TRIM25 |
| C1orf43 | EVI5 |
| PNISR | MOGS |
| HIGD1A | TUBA1A |
| LSM14A | MALL |
| WIPI2 | IFRD2 |
| SERBP1 | MAPKAPK3 |
| PHF19 | PLA2G7 |
| VPS33B | TFEB |
| HEYL | NR4A3 |
| TIMM13 | SLC25A16 |
| AFF4 | MFAP5 |
| VPS4A | USP5 |
| SLCO3A1 | PICALM |
| C16orf72 | EEA1 |
| SSU72 | RECK |
| CLEC2D | NSMAF |
| CD274 | TPST2 |
| RACGAP1 | TPST1 |
| CARD10 | YBX3 |
| HCFC2 | LGR5 |
| SNX11 | PIAS1 |
| SEC61A1 | CDC14B |
| PSAT1 | PLA2G4C |
| MINK1 | STC2 |
| ASCC1 | TP63 |
| ZNF691 | HSD17B6 |
| FCF1 | SSNA1 |
| SIDT2 | OASL |
| SCCPDH | DDX3Y |
| TFB1M | EIF3B |
| CRIM1 | STX10 |
| TMEM69 | PEA15 |
| ANKMY1 | ABCC3 |
| WAC | TNFRSF25 |
| AIG1 | ADAM15 |
| RNF138 | CD164 |
| TMEM138 | SNAP23 |
| PPIL1 | SUCLG2 |
| CHMP3 | IL18R1 |
| RAB23 | LIN7A |
| LUC7L3 | IQGAP1 |
| TM7SF3 | WISP2 |
| CHIC1 | AKAP4 |
| PANK1 | VNN2 |
| DNAJC10 | SPHK1 |
| USP53 | EIF2S2 |
| GNL3L | CPNE3 |
| GATAD2A | CCNA1 |
| BSPRY | PLOD3 |
| QRICH1 | RPS6KA4 |
| DPP8 | GPX7 |
| C2orf42 | GRIA1 |
| AURKAIP1 | GRIA2 |
| STX17 | GRIA3 |
| CDCA4 | NR3C1 |
| PLEKHB2 | GRM1 |
| UACA | CXCL1 |
| PNPO | GRSF1 |
| CEP55 | GSN |
| TBCCD1 | GTF2E1 |
| RIF1 | GUK1 |
| SBNO1 | HSD17B10 |
| TMEM100 | HADHA |
| PPP6R3 | HADHB |
| PI4K2B | HADH |
| RFK | NRG1 |
| AP5S1 | HMGB3 |
| AGPAT5 | NR4A1 |
| SLC39A9 | FOXA2 |
| TMEM63B | HNRNPA2B1 |
| STRADB | HOXC4 |
| CDV3 | HOXC9 |
| UBE2Q1 | HRH1 |
| OTUB1 | HSD17B2 |
| TASP1 | ID1 |
| PNRC2 | ID2 |
| CDC37L1 | ID3 |
| VPS35 | ID4 |
| LRIF1 | IFI16 |
| BEX1 | IFIT2 |
| ASH1L | IFIT3 |
| GNG12 | IGFBP1 |
| MTFR1L | IGFBP3 |
| FAM214A | IGFBP7 |
| C21orf62 | CYR61 |
| CYP26B1 | IL1R1 |
| POLE4 | IL6 |
| C15orf39 | IL6R |
| C5orf15 | IL7 |
| CDC42SE2 | CXCL8 |
| AKR1B10 | IL11 |
| PLSCR4 | ITGA3 |
| ENTPD7 | ITGB3 |
| RALGAPB | STT3A |
| SMURF1 | IVD |
| ACTR3B | JAG2 |
| REXO1 | JUP |
| USP31 | KCNC4 |
| ODF2L | KCNK1 |
| TBC1D14 | KCNK2 |
| TAOK1 | KCNS3 |
| KIAA1456 | KRT14 |
| RANBP10 | KRT33A |
| DHX37 | L1CAM |
| UVSSA | LAMA4 |
| RAP2C | LAMC1 |
| SRPRB | LAMP2 |
| TRAPPC1 | LDLR |
| RPRD1B | LMNA |
| KMT2C | LOXL1 |
| DLGAP3 | LPP |
| SNX16 | LTA4H |
| VSIR | CAPRIN1 |
| IFIH1 | SMAD5 |
| RFWD2 | MAGEA12 |
| ZMAT3 | MAN2A1 |
| C16orf58 | MCAM |
| IPPK | CHST6 |
| KLC2 | MCM7 |
| MRPS11 | MDK |
| MRPL40 | MECP2 |
| TMEM135 | MAP3K3 |
| GRAMD2B | MAP3K4 |
| ZBTB10 | RAB8A |
| ATG9A | MFAP4 |
| CHAC1 | MIA2 |
| TMEM43 | MGMT |
| HAUS3 | MKLN1 |
| PAGR1 | MLLT1 |
| ATP13A3 | MLLT3 |
| CHPF | AFDN |
| TANGO6 | NR3C2 |
| HMBOX1 | MMP19 |
| MCPH1 | MOBP |
| YRDC | MOV10 |
| SLC25A22 | MSN |
| ZFHX4 | MSRA |
| CPSF7 | MUC1 |
| TMC7 | TRIM37 |
| ATAD5 | MVD |
| L2HGDH | MVK |
| PHC3 | MYCN |
| NAA25 | MYH9 |
| FBXL18 | NASP |
| DCAF17 | NCF2 |
| EDC3 | NEDD4 |
| IFT74 | NFATC1 |
| DNAJC5 | NFIA |
| NDFIP1 | NFIB |
| VOPP1 | NFIC |
| C1orf21 | NFIX |
| CDADC1 | NID1 |
| RAB1B | NME4 |
| GSG1 | NNMT |
| RASSF5 | NONO |
| BCL2L12 | PNP |
| AMMECR1L | NPR1 |
| FAM103A1 | NRF1 |
| KATNAL1 | NRTN |
| FBXO30 | YBX1 |
| USP42 | NT5E |
| USP48 | NTF4 |
| POLDIP3 | NTHL1 |
| ZDHHC16 | OCA2 |
| MSANTD4 | TNFRSF11B |
| DCTN5 | ORC1 |
| FAXC | OSBP |
| CBX2 | SERPINE1 |
| AIFM2 | SERPINB2 |
| TBRG1 | PAK3 |
| ZNRF1 | PARN |
| RELT | PBX1 |
| FBXL20 | PCCB |
| SELENOI | PCNA |
| UBE3B | PDE3B |
| MCFD2 | SLC26A4 |
| ZNF622 | VIT |
| PIP4P1 | PGGT1B |
| JPT2 | PGM1 |
| CDKN2AIPNL | PGM5 |
| SMDT1 | PIK3C2A |
| YTHDC1 | PIM1 |
| CHMP7 | PKP1 |
| ZNF585B | PLA2G4A |
| UBE2Q2 | PLOD1 |
| TADA2B | PLP2 |
| ARHGAP12 | RAB31 |
| AHNAK2 | DDX52 |
| SCAMP4 | DUS4L |
| SFT2D1 | TOPBP1 |
| MCM3AP-AS1 | KATNA1 |
| RAB3IP | ZWINT |
| SLC25A29 | CAPN11 |
| ANKRD13B | KAT7 |
| OSCAR | BVES |
| TBC1D20 | FSTL1 |
| CHMP4B | RASSF1 |
| KLHL40 | CHEK2 |
| CPEB2 | IRAK3 |
| LSM11 | TREX2 |
| IRAK1BP1 | CA5B |
| AMER1 | DCTN3 |
| DOCK11 | ATXN2L |
| ZFP28 | STK38 |
| NOL4L | PDAP1 |
| DYNLL2 | CASC3 |
| CACUL1 | NID2 |
| ETFRF1 | NLGN4Y |
| E2F7 | CHSY1 |
| HNRNPA1L2 | DZIP1 |
| CMTM4 | BAHD1 |
| PDIK1L | DIS3 |
| SIK1 | RUFY3 |
| CCDC80 | SBNO2 |
| RNF38 | RAB3GAP1 |
| CREBRF | RPIA |
| TMEM161B | KDM2A |
| AMOT | LIMCH1 |
| AMOTL1 | STK38L |
| PEBP4 | SAMD4A |
| CAMSAP1 | ZHX3 |
| FAM122B | ENDOD1 |
| PPTC7 | GGA2 |
| GPR180 | SWAP70 |
| SPRED1 | KIF1B |
| RNF168 | GPATCH8 |
| ASXL1 | SNX13 |
| RHOV | ABRAXAS2 |
| SCAMP5 | MESD |
| ARID2 | KANK1 |
| TET3 | FAM120A |
| STT3B | PLEKHM2 |
| DENND6A | SULF1 |
| ZNF449 | FBXO28 |
| CYB561A3 | MTCL1 |
| CCDC83 | DNMBP |
| ARMC12 | MTUS2 |
| BRAT1 | IQCE |
| FOXK1 | BICD2 |
| ZNRF2 | KIAA0930 |
| ZNF620 | CUX2 |
| UBN2 | MAN2B2 |
| NEGR1 | ESYT1 |
| MKX | SYNE1 |
| RNF149 | LARP1 |
| FRYL | SIRT1 |
| TCAF2 | CRB1 |
| LURAP1L | GPR161 |
| YIPF6 | SLC44A1 |
| HIST2H3A | SEC11A |
| MSL1 | PES1 |
| ZNF284 | TGDS |
| ZNF391 | POFUT1 |
| MIGA1 | PSD4 |
| ANKRD36 | PDSS1 |
| TMEM189 | HEBP2 |
| TMEM189-UBE2V1 | CD2AP |
| LIN28B | BACE1 |
| XKR9 | TRIM29 |
| MED11 | PLXNB2 |
| C1orf226 | TMEFF2 |
| CCDC88C | RAB38 |
| ATXN7L3B | SHPK |
| FAM229B | SDF2L1 |
| ZNF704 | MTCH1 |
| PHLDB3 | FTSJ1 |
| SLC35E2B | NAT6 |
| POM121C | GCA |
| HSPE1-MOB4 | TMEM184B |
| ADAM10 | METTL7A |
| ADD3 | PLEKHG4 |
| ADH5 | CCDC28A |
| AP1G1 | HEATR5A |
| AGL | KANK2 |
| AGTR1 | CHMP2B |
| ALDH9A1 | NGDN |
| ALDH3A2 | SNED1 |
| ANPEP | LRP10 |
| ANXA2 | ACOT11 |
| RHOA | IPCEF1 |
| ASNS | ZNF451 |
| ASPH | DNM3 |
| ATP6V1C1 | ARHGEF26 |
| BACH1 | LDLRAP1 |
| CARS | TTLL3 |
| CASP3 | RIBC2 |
| CAT | KIF26A |
| RUNX2 | NUPR1 |
| CBFB | SLC17A5 |
| CBL | CHIC2 |
| CD36 | TIMM13 |
| CD68 | TIMM8B |
| CD81 | AATF |
| CDH2 | GREM1 |
| CDH6 | DIEXF |
| CDH13 | HSPB7 |
| CDK5 | STK36 |
| CDKN1B | AGO2 |
| CFL2 | DISC1 |
| CLTC | ARFIP1 |
| COL4A2 | TNFRSF21 |
| CPD | APEX2 |
| CPT1A | SGSM3 |
| CRAT | AAMDC |
| CS | NTMT1 |
| MAPK14 | COMMD5 |
| CSE1L | SLC43A3 |
| CSF1R | ZCCHC4 |
| CSNK1G2 | GTPBP8 |
| CSRP2 | BABAM1 |
| CTLA4 | PYCARD |
| CTNNA1 | MYLIP |
| CTNNB1 | PRSS50 |
| CUX1 | CD274 |
| CYP1A1 | PACSIN3 |
| DAG1 | ABT1 |
| DBN1 | NCAPH2 |
| ECI1 | SNX12 |
| DDB2 | ANAPC4 |
| DDX10 | CERS2 |
| TIMM8A | MDFIC |
| DHCR24 | SLC39A3 |
| DNMT1 | CXXC1 |
| DOCK1 | EHD2 |
| DR1 | TAX1BP3 |
| S1PR1 | ITSN2 |
| EDN1 | AK3 |
| PHC2 | F11R |
| EEF1A2 | RNF141 |
| EEF2 | CDON |
| EPB41L2 | TBX22 |
| EPRS | TMED5 |
| ESRRA | RRP15 |
| ETS1 | GOLT1B |
| F5 | FCF1 |
| FDFT1 | PLLP |
| FGF7 | ABHD5 |
| FKBP3 | RMDN1 |
| FOXE1 | GOLGA7 |
| FLI1 | ANGPTL4 |
| FLNA | CEP83 |
| FLNB | RNFT1 |
| FLT1 | TUBE1 |
| FOS | SS18L2 |
| GALC | ACP6 |
| GATM | GMPR2 |
| GLB1 | TNFRSF12A |
| GCLC | THEM6 |
| GNAS | DACT1 |
| GPM6B | POP5 |
| NR3C1 | SNX7 |
| HAL | NIP7 |
| HK2 | SNX9 |
| HLA-DPA1 | ANAPC5 |
| HMGCS1 | ANAPC7 |
| DNAJB1 | PRRX2 |
| IFNGR1 | PHF7 |
| IGF2R | DPH5 |
| CYR61 | NUB1 |
| JCHAIN | ACSL5 |
| IL2 | STK26 |
| IL6 | SIX4 |
| CXCL8 | GALNT7 |
| IL13RA1 | SHC3 |
| INPP5A | LIMD1 |
| INPP5D | TAF1A |
| EIF3E | CLIC3 |
| ITGB4 | SLC13A2 |
| ITGB5 | PAPSS2 |
| ITK | USP10 |
| JUN | MTMR6 |
| JUNB | SEC22C |
| JUP | INA |
| KCNN3 | SLC16A6 |
| KIF22 | ARHGEF1 |
| KPNA5 | SYNGR2 |
| KRAS | SCAF11 |
| KRT6B | LRRFIP2 |
| FADS1 | AURKB |
| LPL | CRLF1 |
| TACSTD2 | DHRS3 |
| SMAD1 | MAPKAPK2 |
| SMAD4 | TRIP12 |
| SMAD5 | TRIP11 |
| MCAM | B4GALT5 |
| MECP2 | VAMP3 |
| MEST | RPL23 |
| MIA2 | LHX2 |
| MGST2 | PEX16 |
| MITF | ARHGAP29 |
| MKLN1 | TJP2 |
| MLH1 | QKI |
| MAP3K10 | IL27RA |
| MMP16 | MED20 |
| MOV10 | CABP1 |
| MPP2 | ADAMTS1 |
| MUT | VPS4B |
| MXI1 | BAG5 |
| MYB | PTGES |
| MYBL1 | H6PD |
| MYLK | GABBR2 |
| MYO1D | CLOCK |
| MYO1E | BABAM2 |
| MYO6 | WTAP |
| MYO10 | RIN1 |
| NARS | GDA |
| NASP | IKBKE |
| NEU1 | SH3PXD2A |
| NEUROG1 | MICAL2 |
| NFYC | HS2ST1 |
| NKX3-1 | KIAA0355 |
| NOS3 | ECE2 |
| NOVA1 | SART3 |
| NT5E | LAPTM4A |
| OXCT1 | PHACTR2 |
| PEBP1 | RIPOR2 |
| PCCA | PCLAF |
| PCCB | RASSF2 |
| PCDH9 | SNX17 |
| PCNT | MAML1 |
| PDE3A | EFCAB14 |
| PDK1 | ARMCX2 |
| PFDN4 | SEC24D |
| PLAUR | FCHSD2 |
| PLK1 | G3BP2 |
| PLS1 | GFPT2 |
| UBL3 | AMMECR1 |
| PODXL | MVP |
| POLR2C | FGFBP1 |
| CTSA | PTBP3 |
| PPL | GNPDA1 |
| PPM1G | SH2B3 |
| PPP2R2A | TOM1L1 |
| PPP5C | SH2D3A |
| PKIA | AP1M2 |
| PRKCI | ACTR3 |
| PKN2 | CTDSP2 |
| MAPK13 | KIF20A |
| HTRA1 | PREB |
| PSEN1 | HIPK3 |
| PTMS | G3BP1 |
| PTN | FARP1 |
| RAD1 | LPCAT3 |
| PYGL | TSHZ1 |
| RAB2A | MPHOSPH9 |
| RAB3B | MPHOSPH10 |
| RAB27B | CALCRL |
| RAB5C | FLOT1 |
| RAC1 | CTDSPL |
| RAP1B | ANGPTL7 |
| RARS | PLIN3 |
| RCN2 | SPRY2 |
| RHEBP1 | ZMPSTE24 |
| RHEB | FSTL3 |
| RING1 | TRAIP |
| RPL39 | DLEU1 |
| RPS20 | TMEM5 |
| RREB1 | NPM3 |
| RRM2 | KLF2 |
| S100A11 | TUBB4A |
| SCD | CEPT1 |
| CCL2 | RACK1 |
| SDCBP | PEMT |
| SEL1L | RAPGEF3 |
| SELE | CD2BP2 |
| SRSF2 | PGRMC2 |
| SLC7A1 | ACAA2 |
| SMARCD2 | ECI2 |
| SMARCE1 | MAD2L2 |
| UAP1 | TRIM38 |
| SPI1 | EIF3M |
| TROVE2 | SSSCA1 |
| STAT1 | HTATIP2 |
| STIM1 | SLU7 |
| STRN | MXD4 |
| STX5 | RBCK1 |
| STXBP2 | POLR3G |
| SUPT5H | GAS2L1 |
| TBCA | RAD51AP1 |
| TCEA1 | YKT6 |
| TCF4 | B3GNT2 |
| GCFC2 | NUP50 |
| TCF12 | ZBTB6 |
| TMBIM6 | ARPP21 |
| TERF1 | NEK6 |
| TFAM | SDCCAG8 |
| TFCP2 | FAXDC2 |
| TGM2 | PPP1R13L |
| THBS1 | LYVE1 |
| THRB | CPSF4 |
| TJP1 | SMPDL3A |
| TNFAIP2 | SPIN1 |
| TPBG | BTG3 |
| TPP2 | TOMM34 |
| TRIO | IFI44L |
| TRPS1 | ASCC3 |
| TTF1 | METAP2 |
| TXNRD1 | KIF2C |
| TYRP1 | KDELR2 |
| UBE2D2 | TMED1 |
| UBE2D3 | RBPMS |
| UBE2G1 | ABCG8 |
| UGT8 | RHBDF1 |
| VBP1 | NOC3L |
| VCAM1 | NFKBIZ |
| LAT2 | HS1BP3 |
| WRB | NXN |
| XPNPEP1 | GMCL1 |
| XPO1 | GMCL1P1 |
| YWHAZ | INF2 |
| ZFP36 | MFSD14A |
| ZIC3 | MFSD1 |
| ZNF28 | CREB3L2 |
| ZKSCAN1 | MTHFSD |
| ZNF148 | MICAL1 |
| VEZF1 | NMNAT1 |
| ZNF236 | ELOVL1 |
| SLC30A1 | PORCN |
| DEK | ETNPPL |
| ALDH5A1 | FAM129B |
| ABHD16A | CDCP1 |
| PNPLA4 | MRPS11 |
| PICALM | KRI1 |
| STK24 | ZFP69B |
| CUL4B | C8orf33 |
| YBX3 | CAPRIN2 |
| DEGS1 | GRAMD2B |
| YARS | VKORC1 |
| AKR7A2 | DDX50 |
| PSMG1 | TMEM109 |
| AKR1C3 | CCDC86 |
| EIF3A | CARD14 |
| EIF3C | CHCHD7 |
| EIF3F | THOC6 |
| EIF3G | LRRC2 |
| EIF3J | NKAP |
| PEA15 | ADIPOR2 |
| TNFRSF10A | CCDC121 |
| TRIM24 | DYNC2H1 |
| GMPS | PPP1R3B |
| ALDH1A2 | ICE2 |
| SGPL1 | PLEKHF2 |
| EIF2B5 | NLRX1 |
| BUD31 | OGFOD2 |
| FUBP3 | ZDHHC14 |
| MPZL1 | COLGALT1 |
| MAP3K14 | MORC4 |
| UBA3 | LIN28A |
| DOK2 | NSUN7 |
| CLDN1 | ARHGAP28 |
| USP8 | CLMP |
| INA | CCDC102B |
| CNOT9 | ZBTB3 |
| AIFM1 | FAM57A |
| AURKB | CYBRD1 |
| XPR1 | ACTR5 |
| NOLC1 | NOL10 |
| AIMP1 | JADE1 |
| BCL7C | DENND2D |
| TRIP13 | TMEM156 |
| VAMP3 | FOXRED2 |
| SNAP29 | PANK2 |
| SLC9A3R2 | FRAS1 |
| CIAO1 | CTC1 |
| EIF4E2 | MYO19 |
| ADAMTS4 | HKDC1 |
| EEF1E1 | MED28 |
| SCAMP1 | CCDC68 |
| BAG5 | PUS1 |
| ZNF254 | TMEM121 |
| H2AFY | LIMD2 |
| CDC42BPB | WNT5B |
| NUP155 | SYNC |
| FEZ2 | RCC1L |
| IKBKE | LMAN2L |
| SH3PXD2A | CDADC1 |
| TTC37 | FAM83D |
| MARF1 | ANP32E |
| PHF14 | TRIM7 |
| CLINT1 | VANGL1 |
| MATR3 | ARPC5L |
| SERTAD2 | LAS1L |
| TOMM20 | PUS3 |
| NCAPD2 | AIF1L |
| KIF14 | THAP2 |
| MAFB | RASSF5 |
| CHAF1A | CCDC3 |
| UBA2 | FAM167A |
| NR1H3 | PARP9 |
| NAMPT | FRMD8 |
| HNRNPA3P1 | RAB34 |
| ABI2 | CDCA7 |
| PSME3 | SPATA9 |
| DCAF7 | SPRTN |
| ABCC4 | MAGT1 |
| BET1 | KIRREL2 |
| RTN3 | QRFPR |
| NSA2 | ATRIP |
| SEC24B | ASCC2 |
| HAX1 | LOXL4 |
| ZBTB18 | FAR1 |
| DDX17 | USP48 |
| IPO8 | LRRC8C |
| SSSCA1 | MED10 |
| P3H3 | DCUN1D5 |
| ARL6IP5 | TMEM79 |
| SLC35A1 | PHF6 |
| CCT2 | ZBED3 |
| DRAP1 | AKT1S1 |
| PDLIM5 | ADGRA1 |
| RRAGA | ZBTB37 |
| GNA13 | TUBB6 |
| NES | USP38 |
| ZNF273 | MYPN |
| MTHFD2 | CBX2 |
| CCR9 | SFT2D3 |
| CD3EAP | CCDC142 |
| FGL2 | C1orf198 |
| MAN1A2 | FBXO18 |
| PAPOLA | ATOH8 |
| EHD1 | TNS4 |
| TOMM34 | LSM10 |
| METAP2 | DISP1 |
| SLC27A2 | TMEM128 |
| GLIPR1 | ALG2 |
| NUPL2 | ITPRIP |
| KIF3A | FHDC1 |
| CDC37 | TRIM4 |
| FSTL1 | TSR2 |
| PSIP1 | TMEM41A |
| PRAF2 | TMEM263 |
| PDCD10 | SPOCD1 |
| PACSIN2 | JPT2 |
| ACOT7 | IGFN1 |
| PDAP1 | NEK9 |
| EXOC3 | CABLES1 |
| COG2 | CHRDL1 |
| ZNF652 | DSEL |
| AAK1 | MTDH |
| INPP5F | SLC38A5 |
| SHANK2 | NT5C1B |
| TRIM32 | TMEM44 |
| KDM1A | MVB12A |
| CLUAP1 | MAPK1IP1L |
| MRPS27 | TADA2B |
| PLXND1 | HTRA3 |
| RGL1 | TP53INP1 |
| CYFIP1 | CAVIN3 |
| FAM120A | GTF3C6 |
| PSME4 | SNX18 |
| ANKRD12 | CMTM7 |
| CAMTA1 | GLMP |
| EXOC7 | L3HYPDH |
| WWC1 | PHF21B |
| NEMP1 | AHNAK2 |
| DPY19L1 | SCAMP4 |
| UBR4 | CHST14 |
| UFL1 | SMIM12 |
| KIAA0368 | TMEM54 |
| SIRT1 | TUBA3D |
| SKIV2L2 | CYP2U1 |
| CARHSP1 | MBD6 |
| NUP62 | FMNL2 |
| LDOC1 | VASN |
| ARFIP2 | ATG4A |
| ZKSCAN5 | FBXO17 |
| IFIT5 | LRRC42 |
| INTS7 | MALSU1 |
| CNOT10 | FCHO2 |
| GEMIN5 | DHRS1 |
| CLIC4 | CTHRC1 |
| SAMHD1 | LRRC58 |
| SIN3A | LYSMD3 |
| KBTBD2 | GINM1 |
| KANK2 | FAM129A |
| RPAP1 | SH2D1B |
| UPF2 | DEFB118 |
| LTN1 | TWIST2 |
| ZNF500 | SLC15A4 |
| HERC4 | IKBIP |
| CHTOP | FRMD6 |
| ARL5A | TTC8 |
| PHGDH | SENP8 |
| SERGEF | CMTM3 |
| CNNM3 | IQCK |
| INTS6 | ANKS3 |
| HBP1 | INO80C |
| DNAJC2 | RAVER1 |
| CNPPD1 | ZNF440 |
| GHITM | MFSD12 |
| TAF5L | MISP |
| PALD1 | SHE |
| BRPF3 | C1orf122 |
| ARFIP1 | NEU4 |
| LSM3 | PQLC3 |
| RAB30 | LRRC15 |
| MAT2B | TPRA1 |
| HDHD5 | LSMEM2 |
| OSTM1 | AFAP1L1 |
| SPCS1 | RAET1E |
| NOB1 | MTPN |
| MRPL18 | UNC5D |
| PACSIN3 | CARNMT1 |
| TMOD3 | C9orf85 |
| GMPPA | RPP25L |
| DPP7 | SLITRK4 |
| STRN3 | FAM199X |
| UBQLN2 | GAB3 |
| UBQLN1 | SAMD10 |
| PNPLA8 | CCM2L |
| TPRKB | RIPOR3 |
| PAM16 | STK35 |
| GOLT1B | PIWIL4 |
| UBXN1 | PLEKHA7 |
| TXNDC12 | CPNE8 |
| NMD3 | CMTM4 |
| KLHL5 | ZNF548 |
| MECR | ZNF420 |
| IER3IP1 | ATP8B3 |
| KCTD3 | SAMD11 |
| CEP83 | SLC35F3 |
| HSD17B12 | GCSAML |
| C3orf18 | SLC30A7 |
| KRCC1 | NBPF12 |
| MEX3C | LCA5L |
| CDC40 | FAM109B |
| PCYOX1 | ASPRV1 |
| UBE2J1 | GLIPR2 |
| HSD17B7 | PAQR3 |
| ANKFY1 | CREBRF |
| RTFDC1 | CNKSR3 |
| RAB14 | AMOTL1 |
| CUTA | ZNF483 |
| ATP6V1H | ZXDB |
| LUC7L2 | FAM122B |
| FAM96B | TMTC2 |
| HSPB11 | TRPV3 |
| SARAF | CABP7 |
| SELENOT | FAM171B |
| CAB39 | SPIN3 |
| BTBD1 | RHOV |
| TM6SF1 | RILPL2 |
| POLE3 | ZNF626 |
| GAR1 | FAM76A |
| KCTD5 | TXLNA |
| TMX3 | ANKS6 |
| PUS7 | C3orf58 |
| ARL15 | ZCCHC24 |
| SLC35F2 | TPCN2 |
| LY6K | DDIAS |
| KLHL28 | CFAP53 |
| QPCTL | OTUD1 |
| WBP1L | OAF |
| RETSAT | FAM171A1 |
| TBC1D8B | ARL5B |
| DNAAF5 | C6orf89 |
| IMPAD1 | RBM24 |
| MRPL16 | LNX2 |
| C17orf80 | PRR14L |
| PDPR | SLC25A30 |
| DET1 | LCLAT1 |
| ARGLU1 | MSRB3 |
| INTS10 | RNF144B |
| RPRD1A | PCSK9 |
| ARL8B | BCL6B |
| DCUN1D2 | ZNF549 |
| ETNK2 | GK5 |
| OGFOD1 | FRMD3 |
| IL17RB | SGMS1 |
| SOX6 | SEC14L3 |
| TRMT1 | POC1B |
| LRRC40 | MKX |
| CHD7 | NEAT1 |
| INTS8 | P4HA3 |
| YEATS2 | ZNF740 |
| NSUN5 | B4GALNT3 |
| RBM22 | C12orf40 |
| CNDP2 | GXYLT1 |
| ZNF83 | SLC46A3 |
| EXOC2 | C14orf178 |
| RIOK2 | FAM177A1 |
| KDM3A | TSEN54 |
| ERBIN | CDRT4 |
| SLC25A40 | TMEM102 |
| CIAPIN1 | CAVIN1 |
| C16orf62 | LAMA1 |
| TWSG1 | SLC9A9 |
| AGTRAP | EOGT |
| ATP13A1 | DOK7 |
| CORO1B | FAM83H |
| SLC39A10 | FAM133A |
| ZNF248 | DDX51 |
| ERGIC1 | CHSY3 |
| MRS2 | KCNK18 |
| RPTOR | ANKRD42 |
| FAM135A | FAM19A2 |
| WDFY1 | KRT17P2 |
| HOMEZ | ADAMTSL5 |
| ZNF492 | ZNF678 |
| VPS18 | CCDC39 |
| CHD8 | HEPHL1 |
| ZSWIM6 | HNRNPCL1 |
| RBAK | EYS |
| SNX6 | TUBB2B |
| C12orf10 | AMIGO2 |
| SLC25A19 | GSTK1 |
| RNF123 | TMEM179B |
| GOLPH3 | KIF7 |
| ATL2 | FAM89A |
| ATG3 | NUDT19 |
| ATPAF1 | ZNF772 |
| CREB3L2 | NCMAP |
| GNPNAT1 | XKRX |
| DDRGK1 | CTXN1 |
| MRPS34 | FAM35BP |
| SECISBP2 | PPDPFL |
| KDELC1 | DNAJC25 |
| RBM42 | WASF4P |
| CDC73 | ANXA8 |
| CARS2 | PRB2 |
| NAA16 | HOTAIR |
| DYNC2H1 | DNAJC1 |
| DHX40 | TFB2M |
| FASTKD1 | ROBO3 |
| SAP30L | TOR3A |
| COLGALT1 | FAM206A |
| CCDC82 | TIPIN |
| ERMP1 | FKBP14 |
| VCPIP1 | NOL8 |
| ZNF703 | VPS37C |
| THOC7 | PDPR |
| MUS81 | TMEM248 |
| CHD9 | TMEM45A |
| EDEM3 | ANO1 |
| GRPEL1 | RBM28 |
| CDK5RAP3 | SRBD1 |
| WDR82 | WRAP53 |
| FIP1L1 | ARMC1 |
| CAB39L | CENPQ |
| TSPAN14 | APPL2 |
| DIAPH3 | ATAD3A |
| VANGL1 | RAVER2 |
| ZNF611 | LRRC1 |
| HSDL1 | SMU1 |
| MARVELD1 | ELP2 |
| LONP2 | PGM2 |
| RAB34 | PI4K2B |
| FCAMR | DDX19A |
| OBSCN | RSAD1 |
| ARMC2 | LIN7C |
| ZNF644 | DRAM1 |
| ANTXR1 | SYNJ2BP |
| POLR1B | IMPACT |
| FAR1 | GPALPP1 |
| PHF6 | CDCA7L |
| CMSS1 | CNOT11 |
| CARD11 | CDV3 |
| MCM8 | SMPD4 |
| KIAA1841 | LRRC40 |
| GPT2 | DEPDC1 |
| CBR4 | NECAP2 |
| NFATC2IP | ENAH |
| MASTL | CSGALNACT1 |
| TNKS1BP1 | PECR |
| SSH2 | BEX1 |
| PNPT1 | ZNF395 |
| MIDN | BCAP29 |
| OTULIN | SLC50A1 |
| ZNF468 | BARX1 |
| ZNF160 | KIAA1217 |
| ERI1 | KYAT3 |
| FMNL3 | PPAN |
| CHURC1 | MMP26 |
| CHRDL1 | CLDND1 |
| ZNF300 | SLC2A4RG |
| INTS4 | TM9SF3 |
| OXNAD1 | ANKS1B |
| UBTD2 | MAP3K7CL |
| SPECC1 | DHX33 |
| SLC38A5 | SMCO4 |
| ZNF561 | MEPE |
| PAXBP1 | POGLUT1 |
| TP53INP1 | TWSG1 |
| CEP41 | PLSCR3 |
| RDH13 | PLSCR4 |
| CYP2U1 | CPA6 |
| FMNL2 | AVEN |
| OSBPL9 | PNPLA2 |
| OSBPL10 | RNPEPL1 |
| TMEM123 | SLC44A2 |
| SSX2IP | ATP8B2 |
| UBE2J2 | CEMIP |
| ANAPC16 | TTC7A |
| IKBIP | GJD2 |
| CSNK1A1L | STX18 |
| TTC8 | MBD3 |
| MTFMT | S1PR5 |
| MSI2 | BRWD1 |
| OVCA2 | RIPK4 |
| SWSAP1 | CHRAC1 |
| FITM2 | WNT4 |
| DNAJC19 | SLC38A2 |
| CD109 | GAR1 |
| UBXN2B | RETREG1 |
| FAM199X | MIEF1 |
| ASB6 | FAM105A |
| BRI3BP | SMOX |
| KDELC2 | RBM47 |
| KRT80 | FAM35A |
| ZNF98 | DDIT4 |
| ZNF714 | GNL3L |
| SLC30A7 | UGT1A10 |
| WTH3DI | PARP14 |
| GLIPR2 | ZNHIT6 |
| TMEM167A | TMED9 |
| MBLAC2 | EPDR1 |
| FAM91A1 | FBLIM1 |
| MOSPD2 | KLHL24 |
| TMTC3 | HAUS6 |
| DENND1B | KLHL28 |
| ZNF431 | NDE1 |
| ZNF384 | ASPN |
| FAM76A | GPATCH4 |
| PDE12 | TMEM104 |
| TAPT1 | DCAF16 |
| C3orf58 | NSUN2 |
| TYSND1 | INO80D |
| TMEM136 | SPDL1 |
| HNRNPA3 | RASIP1 |
| ARL5B | TMEM161A |
| TDRD6 | RHBDL2 |
| LNX2 | KANSL2 |
| RICTOR | DSCAML1 |
| LCLAT1 | ESYT2 |
| MMS22L | AHRR |
| GCSAM | KLHL42 |
| FAM177A1 | CC2D2A |
| FAM98B | KLHL14 |
| KANSL1 | UNC79 |
| ZNF493 | TMEM181 |
| EOGT | RANBP10 |
| ARL10 | ZBTB2 |
| PAXX | LRRN1 |
| CCDC137 | MYH7B |
| ZNF260 | PLEKHA4 |
| ZNF678 | EPB41L5 |
| AMIGO2 | BEND3 |
| GEN1 | ANKRD36B |
| IRF2BP2 | TRIB3 |
| AGRN | CCAR2 |
| KMT5A | ZNF410 |
| CISD2 | SQOR |
| EIF3CL | RPRD1B |
| MTRNR2L1 | ARHGAP22 |
| MTRNR2L5 | PRDM13 |
| MTRNR2L9 | PLEKHA1 |
| ABCA3 | RXFP1 |
| ABR | ELOVL5 |
| ACAA1 | ABHD4 |
| ACTN4 | KIF13A |
| AP2A2 | SNX16 |
| AP1B1 | LRRC4 |
| ALDOA | TMBIM1 |
| SLC25A4 | DSC3 |
| RERE | ADORA2B |
| ATP7A | APEX1 |
| BCAT2 | ARF6 |
| CD69 | ARHGAP5 |
| LYST | ZFHX3 |
| COPA | FXYD2 |
| DBT | ATP5A1 |
| DLST | ATP6V1C1 |
| EFNB1 | AZGP1 |
| FBN2 | BPGM |
| FKBP4 | BRCA2 |
| G6PD | KLF5 |
| GAA | BYSL |
| GNAI2 | SERPING1 |
| GNB2 | CASP3 |
| GPX1 | CASQ2 |
| GSTM3 | RUNX1 |
| HAGH | CCNT1 |
| ZBTB48 | CDH5 |
| HMGCR | CDH6 |
| IARS | CETN1 |
| IFIT3 | CCR4 |
| IK | CSF1 |
| ILF3 | CSRP2 |
| ITGAV | CTSZ |
| LDLR | CYP2C9 |
| LTBP3 | DARS |
| SMAD6 | DCK |
| MAN2A1 | DCX |
| MCL1 | JAK1 |
| MDK | KCNJ6 |
| MEF2D | E2F3 |
| MSH3 | EIF5 |
| COX1 | EN2 |
| MTF1 | EPB41L1 |
| MYH10 | EPHB1 |
| NCL | ETF1 |
| NONO | FYN |
| NSF | GBA |
| PA2G4 | GCNT2 |
| CDK16 | GLRX |
| PFKFB4 | GNAQ |
| PFN1 | GPI |
| PGAM1 | MKNK2 |
| PIN4 | H3F3B |
| PITPNA | HAL |
| PRKCSH | HINT1 |
| PSMA7 | HLA-DRA |
| PSMB3 | HOXA10 |
| PSMD3 | HOXC6 |
| PSMD5 | NDST1 |
| PTGER4 | IGF1 |
| RAD21 | ILK |
| RAN | ISL1 |
| RBBP5 | ITPR1 |
| RBL2 | RELN |
| RPL3 | PTEN |
| RPL8 | RBBP4 |
| RPS4X | RBBP5 |
| ATXN1 | RFX3 |
| SFPQ | RPL28 |
| SLC15A1 | RPS24 |
| SMARCC2 | RXRA |
| SNRNP70 | CCL17 |
| CAPN15 | SH3BGRL |
| SRP68 | SOX2 |
| TACC1 | SOX11 |
| TAF12 | SPTAN1 |
| TCOF1 | SREBF1 |
| DYNLT3 | SRPRA |
| TESK1 | SSX2 |
| TPT1 | STK3 |
| TSC1 | TBX3 |
| TSPYL1 | NR2F2 |
| USF1 | TGFBR1 |
| UVRAG | CLDN5 |
| WARS | TNNI1 |
| XRCC1 | TSPYL1 |
| YWHAE | UBE2D1 |
| ZNF17 | UBE2N |
| ZNF224 | UGCG |
| EVI5 | WEE1 |
| BAG6 | WRN |
| SLC25A16 | YWHAZ |
| FXR1 | ZNF148 |
| PABPN1 | ZNF236 |
| GAN | SLC39A7 |
| SYMPK | TUSC3 |
| RBM10 | DGS2 |
| SNN | PDHX |
| HIST1H2AM | CLPP |
| HIST2H2AC | FZD9 |
| HIST1H2BF | TAGLN2 |
| ZNF282 | NCK2 |
| RRP1 | DYRK3 |
| EDF1 | IFITM1 |
| MTMR1 | GAS7 |
| SYNGAP1 | MAPKAPK5 |
| MBD2 | ALDH4A1 |
| GPR55 | CCNK |
| GRHPR | DDX18 |
| MRPL19 | FUBP3 |
| SPOCK2 | AP3D1 |
| GIT2 | WASL |
| KEAP1 | HIP1R |
| POM121 | RPS6KA5 |
| G3BP2 | KNG1 |
| DENND4B | LBR |
| JOSD1 | LGALS3 |
| RBM8A | LIFR |
| MPHOSPH10 | SMAD2 |
| AKAP8 | MBL2 |
| BCKDK | MPI |
| RBM14 | MPP2 |
| ARFGEF2 | MYO1F |
| BRD8 | MYO9A |
| TRAFD1 | NDUFC2 |
| OGFR | NEK2 |
| TMEM115 | NOP2 |
| CIT | NTRK3 |
| PWP1 | PDE3A |
| PARK7 | PDK1 |
| U2AF2 | PDPK1 |
| IKZF2 | PFKL |
| KDM2A | PIK3R1 |
| FBXO21 | PKD1 |
| MAST3 | PLA2G5 |
| SMG1 | PLAG1 |
| KIF1B | POU5F1 |
| KIAA0556 | PRKAA1 |
| OTUD3 | MAPK6 |
| KLHL18 | MAP2K1 |
| BICD2 | A2M |
| DOCK9 | ABCA1 |
| POFUT1 | ACADM |
| ADAT1 | ADD1 |
| FJX1 | PCTP |
| DSTYK | ABHD17C |
| SLC39A6 | KMT2C |
| NECAP1 | TLNRD1 |
| REXO2 | MRPS14 |
| IBTK | CLSPN |
| KIF1BP | POPDC2 |
| NPTN | C17orf75 |
| EIF2AK1 | ATPAF1 |
| DKK3 | CERK |
| SLCO4A1 | RMND5A |
| CCDC22 | USP46 |
| DEXI | MRPL44 |
| SNX10 | KCTD14 |
| PYCR2 | PRRG4 |
| KMT5B | RETREG2 |
| RNF181 | ARMT1 |
| DNAJC27 | AKIRIN1 |
| ARL6IP4 | ZNF329 |
| MRPS23 | ZYG11B |
| NLK | MCTP1 |
| RAB8B | ZFHX4 |
| MRPL39 | MOB3B |
| C21orf91 | ZNF552 |
| XRN1 | NANOG |
| PPP1R12C | ZNF614 |
| DNAJB12 | NAA50 |
| FKBP14 | WDCP |
| SETD5 | CPEB4 |
| TCP11L1 | COASY |
| PRR5 | SGPP1 |
| MFN1 | YIPF5 |
| IARS2 | NECTIN4 |
| ASF1B | PPP1R14C |
| CISD1 | ITCH |
| WRNIP1 | ROPN1L |
| THAP10 | KREMEN1 |
| BBX | ZNF394 |
| PHTF2 | TOMM40L |
| AMIGO1 | ARID5B |
| GRAMD1B | POLR1B |
| XPO5 | METTL25 |
| KLHL14 | SETD3 |
| PHRF1 | MCRIP2 |
| SCAF1 | AJUBA |
| PBLD | WNT3A |
| PARVG | ANKRD40 |
| MMS19 | BOC |
| PAPD5 | LONRF1 |
| IFT22 | G6PC3 |
| CASD1 | MOB1B |
| ZSCAN18 | TUBBP1 |
| METRN | IMP4 |
| NABP2 | SFXN1 |
| PHF23 | ARHGAP12 |
| PPCS | VPS26B |
| HPS6 | ZBED6CL |
| QSER1 | FOXP4 |
| PEAK1 | NUS1 |
| COQ8B | HRASLS5 |
| ZNF430 | FRA10AC1 |
| CPTP | SFXN2 |
| SGPP1 | SFXN4 |
| PLA2G12A | WDR81 |
| NIPA2 | ZNF573 |
| C6orf62 | MOB3A |
| COG3 | C19orf47 |
| SLC10A7 | FAM213B |
| MEX3B | LIN54 |
| SLC37A3 | JMY |
| C9orf64 | TMEM68 |
| DNAJC30 | PTCHD1 |
| BRMS1L | DYNLL2 |
| TUBB6 | C11orf84 |
| GLYR1 | TMEM120B |
| PIGO | E2F7 |
| FUT10 | PROSER3 |
| CCNQ | RNF187 |
| IGSF8 | CCDC117 |
| FCHO2 | KLHL23 |
| MRPL53 | SH3D19 |
| PIK3AP1 | TMEM167A |
| EARS2 | ESCO2 |
| NACC2 | TMTC3 |
| SOGA1 | ZFP1 |
| SPC24 | OR7D2 |
| DAB2IP | DNAJB8 |
| ERICH1 | TIGD2 |
| ZNF721 | RASSF6 |
| C2orf69 | MIER3 |
| UNC5B | DACT2 |
| ORAOV1 | ZNF800 |
| ANKRD52 | FAM9B |
| GXYLT1 | FLCN |
| RAB7B | TMEM192 |
| OR6V1 | CDY2B |
| MIA3 | RNF152 |
| HIST2H4B | RNF182 |
| ATP1A3 | GPATCH11 |
| CAMK4 | MMS22L |
| CYP1B1 | C2orf72 |
| LAIR1 | BLOC1S2 |
| MSX2 | PROX2 |
| MYO1C | RGPD4 |
| PGK1 | SUMF1 |
| DNAJC3 | COX18 |
| PSD | FRYL |
| ATXN2 | ARL10 |
| TEAD1 | TUSC5 |
| TK1 | KRTAP13-2 |
| TRPC5 | CCDC36 |
| WT1 | SLC35F4 |
| ZNF138 | MACC1 |
| ARID1A | RILPL1 |
| CDKL2 | BPIFB3 |
| ABCG2 | PTCHD3 |
| NOS1AP | C6orf120 |
| CHST4 | SLC6A17 |
| COG5 | H3F3C |
| ARPP19 | GOLGA8B |
| ZC3H7B | UNC13C |
| TNFRSF13B | TMEM91 |
| MTHFD1L | SSX2B |
| TIMM10B | TMEM170B |
| RABGEF1 | C15orf38-AP3S2 |
| THYN1 | NDUFC2-KCTD14 |
| REPIN1 | ZBTB20 |
| NSG2 | FBXO6 |
| SUFU | HSPB8 |
| MTPAP | TIMM10 |
| SEC61A2 | NBEA |
| TXLNG | BEX3 |
| ANKRD50 | TSPAN13 |
| PNMA8B | AFF4 |
| ZNF490 | LSM1 |
| MKL2 | RND1 |
| NOX5 | TOR2A |
| ZNF419 | CCDC59 |
| GTPBP3 | NXT1 |
| REPS1 | USP25 |
| KLHDC3 | YPEL1 |
| GJD3 | SNX11 |
| ZFAND2B | SEC61A1 |
| OCIAD2 | PDZRN4 |
| SMCR8 | NOP53 |
| TCF23 | EFEMP2 |
| MIER3 | KMT5B |
| MUC20 | DBR1 |
| RSBN1L | SUCO |
| FADS6 | HACD3 |
| ZNF852 | TRMT112 |
| KRTAP21-2 | FAM49B |
| ARL5C | DYNC2LI1 |
| IL17REL | RAB8B |
| FAM27E2 | KDM3B |
| BMP2 | NEURL1B |
| CACNA1E | SDK2 |
| CBS | MBTD1 |
| DPH1 | S1PR2 |
| ELF2 | SLC22A14 |
| KDSR | ADAMTS4 |
| GMPR | GDF15 |
| PRICKLE3 | BAG2 |
| NFE2L2 | CREB5 |
| RANBP2 | ABCG1 |
| RPL37A | RGS6 |
| SALL2 | GCC2 |
| SSB | PHF14 |
| STAT5B | SUPT7L |
| TKT | NCAPD2 |
| PRDM2 | KBTBD11 |
| PTP4A1 | TNFSF15 |
| BCL7B | NR1D2 |
| ADAMTS2 | SLC17A4 |
| KIAA0232 | AP3S2 |
| PLEKHM1 | STAG1 |
| URB1 | TUBB4B |
| NUP153 | ATP8A1 |
| UQCR11 | ZBTB18 |
| ADRM1 | APPBP2 |
| HNRNPUL1 | TXNIP |
| COPE | RGS14 |
| KDM4B | GNB5 |
| SMG5 | POP4 |
| PRELID1 | GPR83 |
| ZNF638 | PDIA5 |
| STMN3 | LILRB5 |
| CHST15 | SNRNP27 |
| LRP1B | MID2 |
| WRAP53 | TPPP |
| PHF10 | ADAMTS5 |
| ERO1B | CD300A |
| CABLES2 | CLCA4 |
| UTP4 | DNAJC8 |
| ZNF689 | R3HDM2 |
| TTL | KIN |
| RBM33 | RAB21 |
| ZBTB7C | WDTC1 |
| STAG3L2 | FNBP1 |
| ARHGAP1 | PEG10 |
| ENTPD5 | CDK19 |
| GABRB3 | PDS5A |
| LGALS3 | SMCHD1 |
| MAT2A | UBR4 |
| RGR | HEY2 |
| SLC5A6 | SUZ12 |
| BCLAF1 | DDAH1 |
| HUWE1 | RABGAP1 |
| YAP1 | PNKD |
| FBXO8 | PYGO1 |
| EEF2K | GPAM |
| MAP10 | ZNF562 |
| RCC2 | VPS13C |
| ALG1 | WDR55 |
| ZFP91 | SNRK |
| SFT2D3 | CMTM6 |
| EIF5AL1 | MOB1A |
| TRIM16L | PSPC1 |
| CASP16P | FBXW7 |
|  | PI4K2A |
|  | TRIM36 |
|  | INTS8 |
|  | TMEM30A |
|  | PRR11 |
|  | EMC3 |
|  | CISD1 |
|  | GNG12 |
|  | MUC13 |
|  | C8orf4 |
|  | STARD7 |
|  | AGTRAP |
|  | PITHD1 |
|  | KIAA1191 |
|  | RAB22A |
|  | CYP20A1 |
|  | TBC1D24 |
|  | ZNF490 |
|  | NLN |
|  | INTS2 |
|  | TAOK1 |
|  | SH3RF1 |
|  | TP53INP2 |
|  | SERPINA3 |
|  | ASGR2 |
|  | ATOX1 |
|  | BMP7 |
|  | BNIP3L |
|  | CDC42 |
|  | CHRM3 |
|  | CNN2 |
|  | CSE1L |
|  | CTBP1 |
|  | DBN1 |
|  | DR1 |
|  | ESRRA |
|  | HOXC8 |
|  | MCM4 |
|  | MSI1 |
|  | COX2 |
|  | MYO1C |
|  | NFYB |
|  | PAFAH1B2 |
|  | PAX9 |
|  | PLS1 |
|  | MED1 |
|  | Nr1i3 |
|  | KDM1A |
|  | GRAMD4 |
|  | UBXN4 |
|  | SLC39A14 |
|  | PPP1R14B |
|  | BTBD1 |
|  | RBM27 |
|  | MRPS21 |
|  | DUS2 |
|  | TRMT10C |
|  | TMA16 |
|  | CTPS2 |
|  | USP28 |
|  | SPCS3 |
|  | CIDEC |
|  | GIGYF1 |
|  | NBEAL1 |
|  | LONRF3 |
|  | PTGES2 |
|  | KLHL15 |
|  | KDM7A |
|  | CALN1 |
|  | SLX4 |
|  | TXNDC17 |
|  | ZNF804A |
|  | SFXN5 |
|  | PCMTD1 |
|  | COX20 |
|  | RFTN2 |
|  | LYPD6 |
|  | FUNDC1 |
|  | WFDC6 |
|  | ZDHHC15 |
|  | FUT11 |
|  | ADAMTS15 |
|  | AGO4 |
|  | VMA21 |
|  | YTHDF3 |
|  | PPP1R37 |
|  | ZNF326 |
|  | SREK1IP1 |
|  | XKR7 |
|  | NUP43 |
|  | SLCO4C1 |
|  | LMOD2 |
|  | ZFP62 |
|  | AP5Z1 |
|  | HNRNPDL |
|  | LHFPL2 |
|  | TRIM13 |
|  | EIF1 |
|  | COQ7 |
|  | ZNF267 |
|  | NCOA2 |
|  | PAIP1 |
|  | KDM5B |
|  | GLIPR1 |
|  | RASGRF1 |
|  | RPL9 |
|  | CXCL12 |
|  | SLC6A3 |
|  | SNRPE |
|  | TEF |
|  | TGFB2 |
|  | ZNF24 |
|  | NCOA3 |
|  | CUL4A |
|  | DCLK1 |
|  | KDM4A |
|  | ZC3H11A |
|  | PHF13 |
|  | METTL21A |
|  | SGO1 |
|  | SLC38A9 |
|  | IRX2 |
|  | SDR16C5 |
|  | TUBB |
|  | NEGR1 |
|  | TRIM24 |
|  | ZMYM3 |
|  | ONECUT2 |
|  | SPTLC2 |
|  | NUP153 |
|  | CASP8AP2 |
|  | CELF1 |
|  | MAP3K2 |
|  | SYNPO |
|  | SMG1 |
|  | CLUH |
|  | NCS1 |
|  | RIMBP2 |
|  | ATL3 |
|  | SLCO3A1 |
|  | GPR171 |
|  | HCFC2 |
|  | PIK3R4 |
|  | TMBIM4 |
|  | BSPRY |
|  | PLEKHB2 |
|  | PNRC2 |
|  | LRRC8A |
|  | PRMT8 |
|  | BBX |
|  | RNF150 |
|  | MTA3 |
|  | DHX35 |
|  | RTP4 |
|  | FBXL17 |
|  | SLC35E1 |
|  | SLC19A3 |
|  | URM1 |
|  | LZIC |
|  | PLEKHA8 |
|  | ZNF648 |
|  | FAM168B |
|  | CCBE1 |
|  | CLTB |
|  | COL19A1 |
|  | CLDN4 |
|  | E2F1 |
|  | EFNA3 |
|  | GLRA2 |
|  | GUCY1A2 |
|  | C5orf51 |
|  | ABCB5 |
|  | AGBL3 |
|  | ZC3H6 |
|  | CLEC17A |
|  | C16orf52 |
|  | KRTAP4-9 |
|  | PFKFB3 |
|  | PPIC |
|  | MAP2K5 |
|  | RPS2 |
|  | RPS4X |
|  | VPS52 |
|  | SFTPB |
|  | SH3GL2 |
|  | SLC4A2 |
|  | SP4 |
|  | TARBP2 |
|  | THRA |
|  | TP53 |
|  | VEGFA |
|  | YWHAH |
|  | CNBP |
|  | ZBTB16 |
|  | ZNF217 |
|  | USP9X |
|  | TRRAP |
|  | HIST1H2AC |
|  | HIST1H3B |
|  | BCAS1 |
|  | HRK |
|  | MLH1 |
|  | ORC5 |
|  | PCK1 |
|  | PCMT1 |
|  | PEX14 |
|  | KPNA1 |
|  | SSR3 |
|  | SOX4 |
|  | WNT9A |
|  | ZNF208 |
|  | TRADD |
|  | HIST1H2BJ |
|  | RNF8 |
|  | GPRC5A |
|  | SLC7A7 |
|  | EMC2 |
|  | TAZ |
|  | KIAA0513 |
|  | ZEB2 |
|  | LRIG2 |
|  | ZBTB39 |
|  | TOM1 |
|  | ARL4C |
|  | SCML2 |
|  | STK25 |
|  | MORF4L1 |
|  | KIAA0895 |
|  | FAM131B |
|  | PFAS |
|  | PKLR |
|  | RPL35A |
|  | RPL41 |
|  | SALL1 |
|  | MRPS23 |
|  | C9orf78 |
|  | MTMR12 |
|  | SGTB |
|  | SDHAF2 |
|  | TMEM74B |
|  | UBE2Q1 |
|  | TENM3 |
|  | CDC42SE1 |
|  | LRRC47 |
|  | KIAA1210 |
|  | SHROOM3 |
|  | MIER1 |
|  | HES4 |
|  | ARHGEF28 |
|  | CCDC14 |
|  | ATAT1 |
|  | PREX2 |
|  | ZKSCAN3 |
|  | TRAF7 |
|  | ZDHHC18 |
|  | MRPL45 |
|  | PHF3 |
|  | ACOT9 |
|  | FJX1 |
|  | ANAPC13 |
|  | GPR132 |
|  | ZBTB7B |
|  | RLIM |
|  | RSRC1 |
|  | SCARA3 |
|  | ACVR1 |
|  | ADCY7 |
|  | GRK2 |
|  | ALOX5AP |
|  | CCND1 |
|  | CAPN5 |
|  | C8A |
|  | FUT4 |
|  | GRIK3 |
|  | HIF1A |
|  | MNX1 |
|  | DNAJB2 |
|  | KIFC1 |
|  | MAK |
|  | MAP1A |
|  | MMP9 |
|  | NOVA1 |
|  | ORC4 |
|  | PCDH7 |
|  | BTBD9 |
|  | C1QTNF6 |
|  | GPR146 |
|  | SSX2IP |
|  | B3GAT2 |
|  | BEST3 |
|  | TMEM199 |
|  | ZNF582 |
|  | DAB2IP |
|  | RNF217 |
|  | TET3 |
|  | SLC25A34 |
|  | NLRP9 |
|  | MYLK4 |
|  | CATSPER4 |
|  | C15orf52 |
|  | SCIMP |
|  | C2orf71 |
|  | PLA2G2C |
|  | VHLL |
|  | POLR2J3 |
|  | ISPD |
|  | UPK3BL1 |
|  | PLA2G4B |
|  | SLFN12L |
|  | SERBP1 |
|  | FGF20 |
|  | PDLIM3 |
|  | AZIN1 |
|  | HAUS2 |
|  | GABPB1 |
|  | GBP2 |
|  | HIVEP1 |
|  | KRAS |
|  | MDM4 |
|  | NINJ1 |
|  | MAPK8 |
|  | RAB5C |
|  | RPL24 |
|  | SCNN1G |
|  | TCF20 |
|  | TFAP2A |
|  | TYMS |
|  | UGT2B4 |
|  | MAFK |
|  | DGKE |
|  | TM4SF5 |
|  | SLC28A1 |
|  | SCAMP1 |
|  | ENTPD4 |
|  | NSA2 |
|  | FAM3C |
|  | SPATS2L |
|  | ALDOA |
|  | BBS2 |
|  | CFTR |
|  | COX6B1 |
|  | COX8A |
|  | CREB1 |
|  | FGF9 |
|  | ERBIN |
|  | KLHL4 |
|  | WDR45B |
|  | HRH4 |
|  | MRPS25 |
|  | GPR135 |
|  | MTMR9 |
|  | NUBPL |
|  | ULBP2 |
|  | ITIH5 |
|  | TGIF2LX |
|  | LRIG3 |
|  | AFMID |
|  | TMEM229B |
|  | LONRF2 |
|  | ZNF584 |
|  | LEMD2 |
|  | SPRYD4 |
|  | SSC5D |
|  | ZNF780A |
|  | YIPF6 |
|  | POTEG |
|  | POTEM |
|  | FKBP1C |
|  | ACVR2B |
|  | ARCN1 |
|  | CDC25A |
|  | AES |
|  | ERCC2 |
|  | CS |
|  | RCOR3 |
|  | ZNF701 |
|  | ZNF83 |
|  | PLGRKT |
|  | ZNF286A |
|  | ZFAND3 |
|  | ZNF667 |
|  | GNPNAT1 |
|  | RAPH1 |
|  | ZNF426 |
|  | ZNF669 |
|  | MAP9 |
|  | REEP4 |
|  | DDHD1 |
|  | ZNF611 |
|  | RTL6 |
|  | HOOK3 |
|  | ZNF594 |
|  | ZNF468 |
|  | ZNF682 |
|  | TOP1MT |
|  | NUP35 |
|  | PPP1R1C |
|  | ZNF675 |
|  | SAMD7 |
|  | FAM229B |
|  | ZNF732 |
|  | ZNF286B |
|  | ADAM22 |
|  | ZRANB1 |
|  | DNAJC28 |
|  | C1orf27 |
|  | KCNJ12 |
|  | XBP1P1 |
|  | YWHAG |
|  | ZNF23 |
|  | ZNF90 |
|  | ZNF99 |
|  | ZNF138 |
|  | DEK |
|  | ANP32A |
|  | FZD6 |
|  | PPM1D |
|  | ZNF254 |
|  | VGLL4 |
|  | RIMS3 |
|  | MELK |
|  | NUAK1 |
|  | PAN2 |
|  | CCT7 |
|  | BTBD3 |
|  | MAPRE3 |
|  | HIC2 |
|  | ATP11A |
|  | KIAA0368 |
|  | ZNF544 |
|  | ZNF117 |
|  | MEG3 |
|  | ALOX15 |
|  | CHEK1 |
|  | B2M |
|  | EN1 |
|  | FOXC1 |
|  | GZMM |
|  | HNRNPC |
|  | MTHFD1 |
|  | PAX5 |
|  | CDK16 |
|  | PKIA |
|  | PVR |
|  | RPA1 |
|  | THY1 |
|  | UBE2G1 |
|  | UCP2 |
|  | RND2 |
|  | HIST1H2BN |
|  | SORBS2 |
|  | SLC5A6 |
|  | SYT7 |
|  | RAB11B |
|  | HAND2 |
|  | BCAR1 |
|  | RBM8A |
|  | LRPPRC |
|  | SLC43A2 |
|  | ZSWIM7 |
|  | ZNF570 |
|  | ZNF550 |
|  | CCDC71L |
|  | LRRC45 |
|  | LRTOMT |
|  | REEP3 |
|  | C14orf180 |
|  | GDF5OS |
|  | TEX22 |
|  | URGCP-MRPS24 |
|  | BARHL1 |
|  | YAE1D1 |
|  | TBX20 |
|  | NLGN4X |
|  | SINHCAF |
|  | FN3K |
|  | SUSD1 |
|  | WDR13 |
|  | VWA1 |
|  | MRPL9 |
|  | SMIM7 |
|  | ORAI2 |
|  | DNAL1 |
|  | ZMIZ2 |
|  | CNDP1 |
|  | HIST1H2AH |
|  | MCU |
|  | ZNF486 |
|  | ATG2A |
|  | SSBP2 |
|  | FAM184B |
|  | NOB1 |
|  | AMDHD2 |
|  | TUBD1 |
|  | ZBTB7A |
|  | ZNF589 |
|  | RASD1 |
|  | SLC38A4 |
|  | PNPO |
|  | SETD5 |
|  | ZNF585B |
|  | TMEM170A |
|  | ARF1 |
|  | CDH7 |
|  | ACHE |
|  | FOXG1 |
|  | CSTF2 |
|  | MAOB |
|  | SLC7A2 |
|  | NR2E1 |
|  | TXNRD1 |
|  | NELFE |
|  | FXR2 |
|  | UBE2E3 |
|  | CELF2 |
|  | RPH3A |
|  | ASTN2 |
|  | C16orf72 |
|  | PSAT1 |
|  | MRPL35 |
|  | ASB1 |
|  | RBM41 |
|  | VAC14 |
|  | KIAA1586 |
|  | NABP1 |
|  | CDC73 |
|  | SH3TC2 |
|  | SLC25A22 |
|  | RSPH3 |
|  | USP32 |
|  | MCFD2 |
|  | FOXQ1 |
|  | WDFY2 |
|  | BECN1 |
|  | TOR1AIP2 |
|  | THAP5 |
|  | C7orf33 |
|  | MPEG1 |
|  | SPATA21 |
|  | PSAPL1 |
|  | HYPK |
|  | RASGRP3 |
|  | NIN |
|  | TRPV2 |
|  | LSM8 |
|  | CCSER2 |
|  | THG1L |
|  | DHTKD1 |
|  | TMEM127 |
|  | ASH1L |
|  | CTNNBL1 |
|  | ARID1B |
|  | PHF12 |
|  | ZFP14 |
|  | SCOC |
|  | AZI2 |
|  | WDR59 |
|  | TRAPPC13 |
|  | VMP1 |
|  | SPPL2A |
|  | SEMA4F |
|  | POLR3A |
|  | RAB11FIP2 |
|  | VPS13A |
|  | CBX6 |
|  | RPL13A |
|  | ACAP2 |
|  | KIAA1549L |
|  | RPL7L1 |
|  | DPY19L4 |
|  | SLC35E4 |
|  | IRF2BP2 |
|  | SBK1 |
|  | LINC00598 |
|  | MT1E |
|  | RPL37 |
|  | STXBP2 |
|  | ZBTB25 |
|  | ESS2 |
|  | ABCB11 |
|  | ITGBL1 |
|  | CYTIP |
|  | NOS1AP |
|  | DDX46 |
|  | STX6 |
|  | RCAN2 |
|  | YAP1 |
|  | FAM83F |
|  | GJD3 |
|  | SPIN4 |
|  | PPM1L |
|  | ATP5G3 |
|  | BNIP2 |
|  | CRK |
|  | HIP1 |
|  | ONECUT1 |
|  | LRPAP1 |
|  | VAV3 |
|  | APP |
|  | EPHA4 |
|  | CEBPG |
|  | HTR7 |
|  | CX3CL1 |
|  | SLC2A3 |
|  | ZNF28 |
|  | HMGA2 |
|  | FRK |
|  | INTS7 |
|  | ZNF695 |
|  | RAB11FIP4 |
|  | FGD4 |
|  | FAM241A |
|  | RNF168 |
|  | PSORS1C2 |
|  | GPR137C |
|  | ZNF763 |
|  | FAM102A |
|  | HIST1H2BG |
|  | EIF3H |
|  | SLC16A3 |
|  | RASSF9 |
|  | SOCS5 |
|  | PPIP5K1 |
|  | HEPH |
|  | TRIM66 |
|  | SLC35E2 |
|  | ABCC5 |
|  | PCGF3 |
|  | COLEC10 |
|  | NUPL2 |
|  | DDX19B |
|  | OLR1 |
|  | NPY4R |
|  | PRKX |
|  | TMPRSS15 |
|  | RPL18A |
|  | MSMO1 |
|  | TRAPPC2 |
|  | TADA2A |
|  | TUFT1 |
|  | UGDH |
|  | UQCRFS1 |
|  | VHL |
|  | VSNL1 |
|  | ZNF157 |
|  | FZD5 |
|  | ZNF331 |
|  | CAMK2N1 |
|  | POLR3E |
|  | PCNP |
|  | PHTF2 |
|  | USP36 |
|  | KIAA1456 |
|  | SH2D4A |
|  | HERPUD2 |
|  | METRN |
|  | TMC5 |
|  | TRMT2B |
|  | CLPB |
|  | POFUT2 |
|  | CSTF2T |
|  | JADE2 |
|  | ATP6V0A2 |
|  | TMEM245 |
|  | GAPVD1 |
|  | FBXL3 |
|  | ARL5A |
|  | SIGLEC8 |
|  | HCAR1 |
|  | SESN1 |
|  | DCTN4 |
|  | ANGPT4 |
|  | GPR173 |
|  | BNC2 |
|  | PPTC7 |
|  | AAED1 |
|  | ARID2 |
|  | STK32A |
|  | SAMD9L |
|  | TMED4 |
|  | SCUBE3 |
|  | NKAPL |
|  | DEFB105A |
|  | C17orf105 |
|  | ZNF619 |
|  | C9orf47 |
|  | KLHL38 |
|  | SMTNL2 |
|  | ZNF850 |
|  | MTX3 |
|  | FOXL2NB |
|  | MXRA7 |
|  | ZNF716 |
|  | FBXO47 |
|  | DEFB105B |
|  | ZNF487 |
|  | STMP1 |
|  | LINC01556 |
|  | HTR5A-AS1 |
|  | KLLN |
|  | ZNF783 |
|  | ZNF417 |
|  | SPC24 |
|  | CKAP2L |
|  | CNTN4 |
|  | ALDH1B1 |
|  | CA6 |
|  | CD40LG |
|  | CHD3 |
|  | COL9A2 |
|  | CSTF1 |
|  | EGR3 |
|  | ERCC4 |
|  | F2RL2 |
|  | GABRB1 |
|  | CXCL10 |
|  | IRAK2 |
|  | KCNN3 |
|  | C9orf64 |
|  | FAXC |
|  | HES7 |
|  | MIDN |
|  | KNSTRN |
|  | TIMM29 |
|  | MYLK3 |
|  | SYAP1 |
|  | TSPEAR-AS2 |
|  | TTC39C |
|  | CHCHD4 |
|  | SLC2A14 |
|  | LYZ |
|  | NUCB1 |
|  | FOXA1 |
|  | PPP2CA |
|  | ZNF195 |
|  | FAM20B |
|  | TSPAN1 |
|  | UBE4B |
|  | SON |
|  | STRN |
|  | MORC3 |
|  | OLA1 |
|  | KLF3 |
|  | PPIL1 |
|  | SOBP |
|  | ZNF415 |
|  | CACNA2D3 |
|  | WSB2 |
|  | ZNF512B |
|  | TMEM67 |
|  | RAB3IP |
|  | SESN3 |
|  | VKORC1L1 |
|  | ZNF846 |
|  | ZDHHC22 |
|  | PIGW |
|  | ZNF268 |
|  | SMYD5 |
|  | MYL9 |
|  | PNPLA6 |
|  | RHOBTB3 |
|  | CAMTA2 |
|  | CTDNEP1 |
|  | SF3B3 |
|  | POLL |
|  | CECR2 |
|  | SLFN12 |
|  | OTUD5 |
|  | PNMA8B |
|  | SENP2 |
|  | CDK15 |
|  | YIPF2 |
|  | CORO7 |
|  | MYH14 |
|  | CYB5B |
|  | CDCA3 |
|  | GLIS2 |
|  | PPP1R9B |
|  | SESTD1 |
|  | ZNF845 |
|  | ZFP3 |
|  | CCSAP |
|  | C22orf39 |
|  | ZBTB46 |
|  | BRI3BP |
|  | TMEM86A |
|  | FAM117B |
|  | KANSL1L |
|  | RAB15 |
|  | ONECUT3 |
|  | ADSS |
|  | PHOX2A |
|  | BDH1 |
|  | AP2M1 |
|  | DMTN |
|  | GDI1 |
|  | OGDH |
|  | P4HB |
|  | PFN1 |
|  | RPL23A |
|  | RRAD |
|  | SLC2A4 |
|  | SP2 |
|  | SUMO1 |
|  | BRPF1 |
|  | DDX39B |
|  | PABPN1 |
|  | SMC1A |
|  | IP6K1 |
|  | TESPA1 |
|  | ARPC3 |
|  | HHLA3 |
|  | DPY19L1 |
|  | HAAO |
|  | SLC39A1 |
|  | TOR4A |
|  | NLRP2 |
|  | LIMS2 |
|  | PLEKHG5 |
|  | GRAMD1B |
|  | DOT1L |
|  | NKX6-2 |
|  | PIGO |
|  | SHANK3 |
|  | BICDL1 |
|  | WFIKKN2 |
|  | RPTN |
|  | FAM43A |
|  | SDK1 |
|  | INO80E |
|  | RTN4RL2 |
|  | ASCL5 |
|  | ACVRL1 |
|  | AP2S1 |
|  | MYH11 |
|  | NEUROD2 |
|  | NKX2-2 |
|  | PER1 |
|  | SKI |
|  | STX1A |
|  | STX4 |
|  | TFAP2B |
|  | KHSRP |
|  | RNF40 |
|  | NDRG1 |
|  | CARM1 |
|  | SEMA6B |
|  | LBX1 |
|  | RAI1 |
|  | GIPC1 |
|  | KDELR1 |
|  | HNRNPA0 |
