# Supplementary material for: Construction of Potential Glioblastoma Multiforme-Related miRNA-mRNA Regulatory Network
Source: Front Mol Neurosci. 2019 Mar 26;12:66. doi: 10.3389/fnmol.2019.00066 (PMC6444190; doi:10.3389/fnmol.2019.00066)
Supplement: Supplementary file 2 [file Table_2.DOCX]

Table S2. DE-mRNAs between GBM samples and normal samples from TCGA database.

| Gene symbol | logFC | AveExpr | t | P.Value | adj.P.Val | B |
| --- | --- | --- | --- | --- | --- | --- |
| SHOX2 | 8.005339 | 8.699806 | 12.2738 | 2.49E-25 | 1.55E-22 | 46.90805 |
| MYBL2 | 7.497967 | 10.35096 | 12.98292 | 2.28E-27 | 2.13E-24 | 51.5215 |
| HOXA5 | 7.467827 | 7.634577 | 9.421088 | 2.79E-17 | 2.57E-15 | 28.69565 |
| TOP2A | 7.451534 | 11.77919 | 13.48022 | 8.46E-29 | 1.66E-25 | 54.75744 |
| RRM2 | 7.423416 | 10.809 | 16.37986 | 4.70E-37 | 9.24E-33 | 73.40907 |
| UBE2C | 7.350585 | 10.10714 | 14.04644 | 2.00E-30 | 9.83E-27 | 58.43564 |
| HOXD10 | 7.270385 | 7.061466 | 7.444178 | 4.36E-12 | 1.02E-10 | 16.97991 |
| PBK | 7.165351 | 9.729366 | 13.28775 | 3.03E-28 | 4.58E-25 | 53.50538 |
| HOXA10 | 7.086218 | 7.282462 | 7.668081 | 1.19E-12 | 3.23E-11 | 18.24513 |
| HOXC10 | 7.056288 | 7.370636 | 6.284218 | 2.58E-09 | 3.00E-08 | 10.75964 |
| EN1 | 7.048953 | 7.386586 | 7.870316 | 3.66E-13 | 1.12E-11 | 19.40355 |
| HP | 7.008261 | 7.723787 | 6.32519 | 2.08E-09 | 2.47E-08 | 10.96873 |
| NKX2-5 | 7.005041 | 6.954712 | 7.264838 | 1.21E-11 | 2.52E-10 | 15.98034 |
| HOXA3 | 6.968866 | 7.017517 | 7.955628 | 2.21E-13 | 7.28E-12 | 19.89648 |
| DLGAP5 | 6.935196 | 8.654903 | 13.31761 | 2.48E-28 | 4.07E-25 | 53.69963 |
| HOXD9 | 6.89519 | 6.825984 | 7.758633 | 7.04E-13 | 2.00E-11 | 18.76204 |
| MMP9 | 6.795906 | 9.687014 | 7.302906 | 9.77E-12 | 2.07E-10 | 16.19145 |
| MELK | 6.671617 | 9.039068 | 12.62322 | 2.47E-26 | 2.02E-23 | 49.18052 |
| HOXD8 | 6.652101 | 6.859611 | 7.535685 | 2.57E-12 | 6.35E-11 | 17.49473 |
| BIRC5 | 6.644806 | 10.90128 | 12.35709 | 1.44E-25 | 1.05E-22 | 47.44945 |
| PLA2G2A | 6.633106 | 8.686917 | 5.538993 | 1.11E-07 | 8.69E-07 | 7.112689 |
| PITX1 | 6.622641 | 6.995329 | 7.16796 | 2.10E-11 | 4.10E-10 | 15.44573 |
| KIF20A | 6.576711 | 9.22058 | 14.05747 | 1.86E-30 | 9.83E-27 | 58.50719 |
| HOXB3 | 6.525339 | 8.253553 | 6.663544 | 3.41E-10 | 4.91E-09 | 12.72691 |
| ADAMDEC1 | 6.513062 | 6.445879 | 7.92995 | 2.57E-13 | 8.29E-12 | 19.74785 |
| SAA1 | 6.490536 | 8.055905 | 4.893408 | 2.25E-06 | 1.30E-05 | 4.216123 |
| AURKB | 6.468499 | 8.875741 | 12.1877 | 4.40E-25 | 2.62E-22 | 46.34857 |
| HOXB7 | 6.410521 | 6.939053 | 7.014337 | 4.96E-11 | 8.78E-10 | 14.60597 |
| HOXD13 | 6.410353 | 6.377113 | 5.478177 | 1.49E-07 | 1.14E-06 | 6.828889 |
| TROAP | 6.317478 | 8.940685 | 12.0725 | 9.43E-25 | 4.78E-22 | 45.60042 |
| LTF | 6.310759 | 12.01116 | 4.905938 | 2.13E-06 | 1.23E-05 | 4.269816 |
| POSTN | 6.063778 | 11.00829 | 5.153211 | 6.91E-07 | 4.49E-06 | 5.35036 |
| NCAPG | 6.014222 | 9.176492 | 12.27222 | 2.52E-25 | 1.55E-22 | 46.89778 |
| NDC80 | 6.011253 | 8.927573 | 13.18853 | 5.84E-28 | 7.18E-25 | 52.8597 |
| HOXA2 | 6.010289 | 5.96651 | 6.061706 | 8.17E-09 | 8.35E-08 | 9.639197 |
| E2F8 | 5.963276 | 6.944678 | 12.0716 | 9.48E-25 | 4.78E-22 | 45.59454 |
| HJURP | 5.963095 | 8.960125 | 12.28939 | 2.25E-25 | 1.48E-22 | 47.00942 |
| HOXD11 | 5.956246 | 6.031159 | 6.128043 | 5.81E-09 | 6.18E-08 | 9.970536 |
| HOXA4 | 5.942782 | 6.033513 | 5.57991 | 9.10E-08 | 7.25E-07 | 7.304862 |
| PIMREG | 5.928224 | 9.789551 | 10.22409 | 1.67E-19 | 2.59E-17 | 33.7223 |
| HOXB4 | 5.894506 | 6.084431 | 5.495956 | 1.37E-07 | 1.05E-06 | 6.91163 |
| ASF1B | 5.884955 | 9.569888 | 13.04752 | 1.49E-27 | 1.47E-24 | 51.94197 |
| HOXA7 | 5.873996 | 5.856168 | 5.802499 | 3.04E-08 | 2.70E-07 | 8.367211 |
| IGF2BP3 | 5.863705 | 8.414695 | 8.994421 | 4.03E-16 | 2.78E-14 | 26.07642 |
| MKI67 | 5.835913 | 10.41637 | 10.30188 | 1.01E-19 | 1.67E-17 | 34.2149 |
| PI3 | 5.83471 | 7.021187 | 4.681767 | 5.71E-06 | 2.97E-05 | 3.325012 |
| CENPA | 5.814472 | 7.88866 | 12.04813 | 1.11E-24 | 5.31E-22 | 45.44224 |
| FAM111B | 5.808088 | 8.230962 | 10.74681 | 5.67E-21 | 1.36E-18 | 37.04778 |
| GSC | 5.80464 | 6.176553 | 8.734111 | 2.01E-15 | 1.17E-13 | 24.49957 |
| DPEP1 | 5.690431 | 7.476499 | 6.646505 | 3.74E-10 | 5.34E-09 | 12.63706 |
| OTP | 5.598759 | 6.537633 | 6.074041 | 7.67E-09 | 7.90E-08 | 9.700632 |
| HOXB2 | 5.597717 | 7.435204 | 5.886829 | 1.99E-08 | 1.85E-07 | 8.777005 |
| CDCA2 | 5.588668 | 7.742441 | 10.95774 | 1.43E-21 | 4.15E-19 | 38.39896 |
| IGFBP2 | 5.526522 | 13.93027 | 9.566862 | 1.11E-17 | 1.13E-15 | 29.59944 |
| HOXC6 | 5.494011 | 6.050112 | 6.855204 | 1.20E-10 | 1.93E-09 | 13.74674 |
| CDC45 | 5.490453 | 8.586067 | 10.32991 | 8.45E-20 | 1.47E-17 | 34.39259 |
| KIFC1 | 5.459421 | 9.694942 | 10.38475 | 5.93E-20 | 1.06E-17 | 34.74061 |
| HOXC11 | 5.450944 | 5.294307 | 4.842048 | 2.83E-06 | 1.59E-05 | 3.997111 |
| E2F2 | 5.429252 | 8.071822 | 9.718372 | 4.26E-18 | 4.78E-16 | 30.54319 |
| CCNB2 | 5.411353 | 9.613972 | 11.07153 | 6.82E-22 | 2.03E-19 | 39.1297 |
| NUSAP1 | 5.406398 | 10.8068 | 11.83493 | 4.52E-24 | 1.89E-21 | 44.05923 |
| BUB1 | 5.368661 | 9.559741 | 12.34629 | 1.54E-25 | 1.08E-22 | 47.37925 |
| CEP55 | 5.356346 | 8.265404 | 13.04678 | 1.49E-27 | 1.47E-24 | 51.93719 |
| KIF14 | 5.352127 | 7.970769 | 10.9429 | 1.58E-21 | 4.50E-19 | 38.30375 |
| PCLAF | 5.350566 | 9.136659 | 10.31661 | 9.21E-20 | 1.56E-17 | 34.30826 |
| HOXA1 | 5.328213 | 5.701068 | 8.299356 | 2.84E-14 | 1.18E-12 | 21.90654 |
| MEOX2 | 5.289715 | 9.340999 | 5.58859 | 8.73E-08 | 6.99E-07 | 7.345753 |
| AC010422.3 | 5.287348 | 7.046792 | 13.16053 | 7.03E-28 | 8.13E-25 | 52.6775 |
| COL3A1 | 5.285013 | 12.96519 | 6.346032 | 1.86E-09 | 2.24E-08 | 11.07541 |
| HOXC9 | 5.279757 | 5.358416 | 5.355832 | 2.67E-07 | 1.93E-06 | 6.264655 |
| HAND2 | 5.241971 | 5.46009 | 4.807617 | 3.30E-06 | 1.82E-05 | 3.851274 |
| CENPU | 5.228988 | 9.516512 | 10.87504 | 2.46E-21 | 6.62E-19 | 37.86865 |
| GTSE1 | 5.203572 | 8.945411 | 10.69953 | 7.71E-21 | 1.76E-18 | 36.74563 |
| GPR82 | 5.196822 | 6.862647 | 9.859668 | 1.73E-18 | 2.19E-16 | 31.42712 |
| SGO1 | 5.163641 | 7.540804 | 9.855573 | 1.78E-18 | 2.23E-16 | 31.40145 |
| METTL7B | 5.162162 | 11.6092 | 6.911806 | 8.76E-11 | 1.46E-09 | 14.05109 |
| MAGED4B | 5.154304 | 6.284801 | 13.89794 | 5.34E-30 | 1.53E-26 | 57.47193 |
| KNL1 | 5.146387 | 8.578347 | 10.59144 | 1.56E-20 | 3.19E-18 | 36.05572 |
| IBSP | 5.094864 | 8.189024 | 4.99973 | 1.40E-06 | 8.44E-06 | 4.675019 |
| SPOCD1 | 5.086032 | 11.03581 | 6.511256 | 7.74E-10 | 1.02E-08 | 11.92873 |
| TREM1 | 5.083927 | 8.464039 | 6.196578 | 4.07E-09 | 4.52E-08 | 10.31528 |
| TTK | 5.079615 | 8.525219 | 9.578747 | 1.03E-17 | 1.06E-15 | 29.67332 |
| BUB1B | 5.078021 | 8.928132 | 10.62456 | 1.25E-20 | 2.63E-18 | 36.267 |
| HOXC13 | 5.057097 | 5.040709 | 4.265388 | 3.27E-05 | 0.000144 | 1.662229 |
| SRPX2 | 5.043614 | 9.792814 | 7.455243 | 4.09E-12 | 9.62E-11 | 17.04199 |
| HOXA11 | 5.032685 | 4.888068 | 5.139283 | 7.37E-07 | 4.75E-06 | 5.288454 |
| SKA1 | 5.011914 | 7.96572 | 9.868612 | 1.63E-18 | 2.11E-16 | 31.48319 |
| CHI3L1 | 5.006175 | 16.03037 | 5.315903 | 3.23E-07 | 2.28E-06 | 6.082471 |
| ABCC3 | 5.005872 | 10.73594 | 6.614842 | 4.44E-10 | 6.20E-09 | 12.47046 |
| KIF4A | 5.00503 | 9.685089 | 11.40514 | 7.65E-23 | 2.89E-20 | 41.27878 |
| HOXC4 | 4.992137 | 7.837331 | 10.69305 | 8.04E-21 | 1.78E-18 | 36.7042 |
| ESCO2 | 4.991913 | 8.01542 | 9.504376 | 1.65E-17 | 1.59E-15 | 29.21151 |
| MMP7 | 4.983557 | 6.585676 | 4.583685 | 8.71E-06 | 4.37E-05 | 2.922365 |
| PRAME | 4.969145 | 4.955284 | 6.12334 | 5.95E-09 | 6.31E-08 | 9.946968 |
| FOXD3 | 4.965257 | 5.332854 | 6.096054 | 6.85E-09 | 7.16E-08 | 9.810469 |
| CFAP77 | 4.964403 | 6.202503 | 6.549821 | 6.30E-10 | 8.45E-09 | 12.12982 |
| HOXA6 | 4.963197 | 4.820577 | 6.353666 | 1.79E-09 | 2.17E-08 | 11.11454 |
| CD163 | 4.961534 | 12.13756 | 6.583292 | 5.26E-10 | 7.19E-09 | 12.30492 |
| CDK1 | 4.930273 | 10.00645 | 11.11549 | 5.11E-22 | 1.60E-19 | 39.41237 |
| SCNN1B | 4.909808 | 6.241775 | 4.883881 | 2.35E-06 | 1.35E-05 | 4.175365 |
| DTL | 4.885379 | 9.136116 | 9.71389 | 4.38E-18 | 4.89E-16 | 30.51521 |
| NKX3-2 | 4.880605 | 5.102737 | 6.387224 | 1.50E-09 | 1.84E-08 | 11.28689 |
| POTEF | 4.880294 | 4.86003 | 15.16604 | 1.26E-33 | 1.24E-29 | 65.66704 |
| E2F7 | 4.871514 | 8.190978 | 10.08955 | 3.97E-19 | 5.74E-17 | 32.87251 |
| KIF2C | 4.870406 | 9.769343 | 10.59455 | 1.52E-20 | 3.16E-18 | 36.07559 |
| IRX5 | 4.854532 | 6.0295 | 6.447205 | 1.09E-09 | 1.39E-08 | 11.59634 |
| NNMT | 4.819665 | 11.15246 | 5.551432 | 1.05E-07 | 8.22E-07 | 7.171009 |
| FOXM1 | 4.81364 | 10.71966 | 10.27722 | 1.19E-19 | 1.93E-17 | 34.05865 |
| IL2RA | 4.809393 | 6.311856 | 4.622188 | 7.38E-06 | 3.75E-05 | 3.079638 |
| ASPM | 4.792379 | 8.877631 | 8.953922 | 5.18E-16 | 3.43E-14 | 25.82998 |
| APOBEC3B | 4.786499 | 6.926763 | 8.610269 | 4.30E-15 | 2.26E-13 | 23.75555 |
| RAB42 | 4.77803 | 8.350618 | 12.08898 | 8.45E-25 | 4.62E-22 | 45.70742 |
| CA3 | 4.770138 | 9.244247 | 4.890175 | 2.28E-06 | 1.32E-05 | 4.202284 |
| COL4A1 | 4.750356 | 13.89945 | 9.268122 | 7.31E-17 | 5.99E-15 | 27.75198 |
| F2R | 4.749245 | 11.78517 | 12.94157 | 3.00E-27 | 2.68E-24 | 51.25234 |
| GPX8 | 4.704675 | 9.239757 | 7.875975 | 3.54E-13 | 1.09E-11 | 19.43618 |
| HOXC8 | 4.685059 | 4.820888 | 5.162009 | 6.64E-07 | 4.34E-06 | 5.389525 |
| POTEE | 4.680434 | 5.169686 | 13.79095 | 1.08E-29 | 2.66E-26 | 56.77714 |
| MCM10 | 4.670515 | 7.869299 | 8.5667 | 5.61E-15 | 2.86E-13 | 23.49479 |
| HOXA13 | 4.655526 | 5.345373 | 4.448454 | 1.54E-05 | 7.30E-05 | 2.378189 |
| CA9 | 4.651188 | 8.43455 | 4.879147 | 2.40E-06 | 1.37E-05 | 4.155133 |
| CENPK | 4.628912 | 8.47621 | 9.876441 | 1.55E-18 | 2.04E-16 | 31.53228 |
| HOXA9 | 4.627713 | 4.740801 | 4.459788 | 1.47E-05 | 7.00E-05 | 2.423302 |
| CSTA | 4.625171 | 6.822235 | 7.737429 | 7.97E-13 | 2.24E-11 | 18.64074 |
| CDC25C | 4.622525 | 7.536527 | 8.813802 | 1.23E-15 | 7.50E-14 | 24.98049 |
| KIF18A | 4.600862 | 7.802458 | 10.17167 | 2.34E-19 | 3.52E-17 | 33.3909 |
| APOL4 | 4.599157 | 10.20965 | 6.084821 | 7.26E-09 | 7.53E-08 | 9.754387 |
| EZH2 | 4.589914 | 10.35551 | 11.27301 | 1.82E-22 | 6.28E-20 | 40.42654 |
| TGFBI | 4.588183 | 12.77033 | 7.696015 | 1.02E-12 | 2.79E-11 | 18.40428 |
| CDC20 | 4.570351 | 10.15649 | 9.377365 | 3.68E-17 | 3.23E-15 | 28.42541 |
| MAGED4 | 4.552424 | 6.210111 | 12.09841 | 7.94E-25 | 4.46E-22 | 45.76862 |
| C6orf15 | 4.540165 | 4.409701 | 3.317139 | 0.001108 | 0.003308 | -1.64131 |
| TBX5 | 4.538258 | 4.786638 | 3.360107 | 0.000958 | 0.002906 | -1.50701 |
| IRX1 | 4.530791 | 6.480564 | 3.181477 | 0.001736 | 0.004932 | -2.05528 |
| PLEKHS1 | 4.48693 | 5.200941 | 5.409545 | 2.07E-07 | 1.53E-06 | 6.511255 |
| OSR2 | 4.486104 | 5.710951 | 6.639088 | 3.89E-10 | 5.53E-09 | 12.59799 |
| NUF2 | 4.466701 | 8.898594 | 9.038645 | 3.06E-16 | 2.17E-14 | 26.34598 |
| CYP19A1 | 4.465775 | 6.97135 | 7.149828 | 2.32E-11 | 4.49E-10 | 15.3461 |
| NR5A2 | 4.463166 | 6.510724 | 9.858903 | 1.74E-18 | 2.19E-16 | 31.42232 |
| SPINK8 | 4.431613 | 6.753561 | 6.905704 | 9.06E-11 | 1.51E-09 | 14.01821 |
| ESPL1 | 4.423072 | 8.735163 | 9.394772 | 3.30E-17 | 2.95E-15 | 28.53295 |
| IQGAP2 | 4.410589 | 9.87619 | 10.74459 | 5.75E-21 | 1.36E-18 | 37.03362 |
| LHX9 | 4.396753 | 6.714605 | 4.695707 | 5.38E-06 | 2.81E-05 | 3.382775 |
| GATA4 | 4.371506 | 4.387414 | 3.96663 | 0.000106 | 0.000412 | 0.546544 |
| SAA2 | 4.367568 | 5.939403 | 3.222199 | 0.001519 | 0.004386 | -1.93263 |
| UHRF1 | 4.350322 | 10.29995 | 8.31415 | 2.59E-14 | 1.09E-12 | 21.99388 |
| DMRTA2 | 4.326916 | 8.213338 | 4.458077 | 1.48E-05 | 7.04E-05 | 2.416487 |
| NEIL3 | 4.325525 | 7.209807 | 8.324183 | 2.44E-14 | 1.04E-12 | 22.05315 |
| MFAP2 | 4.322525 | 7.742516 | 5.919483 | 1.69E-08 | 1.59E-07 | 8.936737 |
| MS4A6A | 4.301803 | 11.46283 | 7.974944 | 1.97E-13 | 6.59E-12 | 20.00842 |
| GALNT5 | 4.298342 | 6.422272 | 4.84537 | 2.79E-06 | 1.57E-05 | 4.011224 |
| CDCA7 | 4.295706 | 9.727703 | 10.31265 | 9.45E-20 | 1.59E-17 | 34.28315 |
| CXCL10 | 4.273884 | 8.790975 | 5.347449 | 2.78E-07 | 2.00E-06 | 6.226323 |
| NOX4 | 4.265131 | 8.030129 | 8.970143 | 4.69E-16 | 3.14E-14 | 25.92864 |
| TMSB15A | 4.25407 | 7.434631 | 3.908764 | 0.000133 | 0.000503 | 0.338202 |
| IL1R2 | 4.248604 | 5.998611 | 5.176649 | 6.20E-07 | 4.07E-06 | 5.454808 |
| OIP5 | 4.246426 | 7.105678 | 8.826719 | 1.14E-15 | 6.99E-14 | 25.05859 |
| HIST1H2BH | 4.239877 | 4.379542 | 6.717698 | 2.54E-10 | 3.77E-09 | 13.01336 |
| SPC24 | 4.238017 | 9.154223 | 8.270705 | 3.37E-14 | 1.38E-12 | 21.73758 |
| WEE1 | 4.233189 | 10.99097 | 13.24134 | 4.12E-28 | 5.49E-25 | 53.20336 |
| COL1A1 | 4.230958 | 13.01163 | 4.699957 | 5.28E-06 | 2.76E-05 | 3.400411 |
| HAS2 | 4.225831 | 9.322432 | 8.769698 | 1.62E-15 | 9.55E-14 | 24.71412 |
| KIF23 | 4.222092 | 9.090429 | 9.415739 | 2.89E-17 | 2.63E-15 | 28.66257 |
| LEFTY2 | 4.220656 | 7.539486 | 5.619281 | 7.51E-08 | 6.09E-07 | 7.490693 |
| CDCA8 | 4.218292 | 9.349617 | 9.207231 | 1.07E-16 | 8.47E-15 | 27.37774 |
| KIF18B | 4.216539 | 9.202812 | 7.413256 | 5.20E-12 | 1.18E-10 | 16.80666 |
| ORC1 | 4.20695 | 7.915967 | 9.384812 | 3.51E-17 | 3.11E-15 | 28.47141 |
| CKAP2L | 4.204466 | 8.794391 | 8.835713 | 1.08E-15 | 6.68E-14 | 25.11299 |
| TNC | 4.198693 | 14.41234 | 8.572098 | 5.43E-15 | 2.79E-13 | 23.52707 |
| EGFR | 4.192645 | 14.65756 | 4.304114 | 2.80E-05 | 0.000125 | 1.811672 |
| NCAPH | 4.190303 | 9.009372 | 8.759641 | 1.72E-15 | 1.01E-13 | 24.65345 |
| UBD | 4.185085 | 5.048341 | 5.169893 | 6.40E-07 | 4.20E-06 | 5.424667 |
| HLA-DQA2 | 4.179617 | 7.899607 | 4.184707 | 4.53E-05 | 0.000192 | 1.354407 |
| CPXM1 | 4.178348 | 11.25567 | 7.942638 | 2.39E-13 | 7.77E-12 | 19.82126 |
| FCGBP | 4.177733 | 13.68085 | 6.55621 | 6.08E-10 | 8.20E-09 | 12.1632 |
| IDO1 | 4.174225 | 4.768291 | 4.341416 | 2.40E-05 | 0.000109 | 1.956643 |
| TUBA1C | 4.173732 | 11.99642 | 10.72275 | 6.63E-21 | 1.53E-18 | 36.894 |
| CLEC4E | 4.172768 | 6.412135 | 5.903591 | 1.83E-08 | 1.71E-07 | 8.858925 |
| ANGPT2 | 4.151516 | 10.69279 | 8.733655 | 2.02E-15 | 1.17E-13 | 24.49682 |
| GSG2 | 4.14266 | 6.438406 | 8.415294 | 1.41E-14 | 6.31E-13 | 22.59276 |
| HOXB13 | 4.136801 | 4.847347 | 3.394892 | 0.000851 | 0.002608 | -1.39719 |
| HMMR | 4.132509 | 8.440893 | 8.62914 | 3.83E-15 | 2.03E-13 | 23.86866 |
| CCNA2 | 4.124214 | 9.621605 | 9.764353 | 3.18E-18 | 3.72E-16 | 30.83045 |
| TFPI | 4.12205 | 10.26336 | 7.874269 | 3.57E-13 | 1.10E-11 | 19.42634 |
| IRX3 | 4.117662 | 6.551702 | 5.494084 | 1.38E-07 | 1.06E-06 | 6.902905 |
| PTX3 | 4.116977 | 9.791134 | 5.140419 | 7.33E-07 | 4.74E-06 | 5.293497 |
| TGM5 | 4.104883 | 5.610443 | 4.97991 | 1.53E-06 | 9.15E-06 | 4.58891 |
| C21orf62 | 4.078537 | 10.19719 | 6.011215 | 1.06E-08 | 1.06E-07 | 9.38857 |
| CXCL9 | 4.070341 | 6.917277 | 5.308979 | 3.34E-07 | 2.35E-06 | 6.05098 |
| FCGR2B | 4.065957 | 8.204291 | 5.193329 | 5.74E-07 | 3.80E-06 | 5.529356 |
| SKA3 | 4.056714 | 8.425454 | 7.875636 | 3.54E-13 | 1.09E-11 | 19.43422 |
| ABCA13 | 4.047177 | 5.990529 | 4.615511 | 7.60E-06 | 3.85E-05 | 3.052289 |
| GPR65 | 4.032883 | 8.616356 | 8.079864 | 1.06E-13 | 3.79E-12 | 20.61863 |
| TNFRSF12A | 4.026971 | 11.29697 | 7.767059 | 6.70E-13 | 1.92E-11 | 18.81029 |
| CENPM | 4.026362 | 8.321139 | 8.238571 | 4.09E-14 | 1.65E-12 | 21.54838 |
| CLEC5A | 3.996001 | 8.904486 | 5.862481 | 2.25E-08 | 2.06E-07 | 8.658286 |
| ATP6V1B1 | 3.991187 | 6.659525 | 6.879217 | 1.05E-10 | 1.72E-09 | 13.87569 |
| PAX3 | 3.984801 | 5.40009 | 3.70386 | 0.000285 | 0.000993 | -0.37878 |
| TIMP4 | 3.982204 | 11.44695 | 6.542059 | 6.56E-10 | 8.78E-09 | 12.08929 |
| HOXD4 | 3.980074 | 4.016669 | 4.794723 | 3.49E-06 | 1.91E-05 | 3.796867 |
| DEPDC1 | 3.96467 | 8.359668 | 7.956132 | 2.20E-13 | 7.28E-12 | 19.8994 |
| FRRS1 | 3.951853 | 6.134633 | 8.382248 | 1.72E-14 | 7.56E-13 | 22.39676 |
| PDPN | 3.951553 | 13.12775 | 7.020447 | 4.79E-11 | 8.55E-10 | 14.63918 |
| PRR32 | 3.931876 | 4.34176 | 4.744041 | 4.36E-06 | 2.33E-05 | 3.58408 |
| SIX1 | 3.930168 | 7.766023 | 5.866927 | 2.20E-08 | 2.02E-07 | 8.67994 |
| MS4A4A | 3.928455 | 9.858822 | 6.711069 | 2.63E-10 | 3.89E-09 | 12.97823 |
| MDK | 3.926085 | 12.51607 | 9.418594 | 2.84E-17 | 2.60E-15 | 28.68023 |
| FPR3 | 3.912549 | 8.723153 | 6.571108 | 5.62E-10 | 7.64E-09 | 12.24112 |
| TFAP2B | 3.904367 | 3.912147 | 3.570712 | 0.000461 | 0.001515 | -0.82703 |
| HOXB6 | 3.90057 | 4.185519 | 4.153065 | 5.14E-05 | 0.000215 | 1.234989 |
| PLP2 | 3.896495 | 11.44468 | 7.899704 | 3.07E-13 | 9.66E-12 | 19.57308 |
| ANXA1 | 3.893492 | 13.35924 | 7.008064 | 5.13E-11 | 9.02E-10 | 14.57189 |
| POTEI | 3.89259 | 4.275814 | 12.02736 | 1.27E-24 | 5.95E-22 | 45.30741 |
| POTEJ | 3.89177 | 4.416544 | 11.33524 | 1.21E-22 | 4.41E-20 | 40.82773 |
| HOXB9 | 3.885599 | 4.00432 | 4.815846 | 3.18E-06 | 1.76E-05 | 3.886057 |
| GSX1 | 3.882112 | 6.438233 | 3.577946 | 0.000449 | 0.001482 | -0.80304 |
| LYZ | 3.880621 | 10.15517 | 5.702984 | 4.98E-08 | 4.20E-07 | 7.888756 |
| EIF5AL1 | 3.879669 | 7.682724 | 11.85602 | 3.94E-24 | 1.68E-21 | 44.19595 |
| TMEM45A | 3.879648 | 9.870135 | 7.876383 | 3.53E-13 | 1.09E-11 | 19.43853 |
| ANXA2 | 3.875998 | 13.84368 | 8.085678 | 1.02E-13 | 3.70E-12 | 20.65255 |
| CHI3L2 | 3.871713 | 12.63975 | 4.609967 | 7.78E-06 | 3.94E-05 | 3.029608 |
| CKS2 | 3.863605 | 10.07124 | 8.977742 | 4.47E-16 | 3.03E-14 | 25.97488 |
| SPC25 | 3.861397 | 8.375563 | 7.634094 | 1.46E-12 | 3.83E-11 | 18.05188 |
| FAM60A | 3.856259 | 9.820648 | 9.005596 | 3.76E-16 | 2.61E-14 | 26.14449 |
| STEAP3 | 3.849842 | 11.55475 | 7.348666 | 7.53E-12 | 1.65E-10 | 16.44598 |
| PLAU | 3.845964 | 10.47298 | 6.173641 | 4.59E-09 | 5.02E-08 | 10.19963 |
| MDFI | 3.845498 | 10.47199 | 6.205104 | 3.89E-09 | 4.34E-08 | 10.35833 |
| RAD51 | 3.842717 | 8.027214 | 8.492788 | 8.80E-15 | 4.21E-13 | 23.05363 |
| SERPINE1 | 3.829587 | 12.42493 | 5.156737 | 6.80E-07 | 4.43E-06 | 5.36605 |
| TPX2 | 3.822674 | 11.04057 | 8.709914 | 2.33E-15 | 1.33E-13 | 24.35388 |
| ST14 | 3.822151 | 9.257606 | 7.123714 | 2.69E-11 | 5.10E-10 | 15.20285 |
| GJA3 | 3.814651 | 5.106793 | 4.676102 | 5.85E-06 | 3.03E-05 | 3.301576 |
| MNX1 | 3.805271 | 3.84689 | 3.74265 | 0.000247 | 0.000877 | -0.24556 |
| S100A3 | 3.803364 | 7.138864 | 6.460327 | 1.02E-09 | 1.30E-08 | 11.66428 |
| HIST1H3G | 3.802449 | 3.693183 | 5.582275 | 9.00E-08 | 7.18E-07 | 7.315999 |
| FOXJ1 | 3.800578 | 9.770709 | 4.791776 | 3.53E-06 | 1.93E-05 | 3.784446 |
| PDCD1LG2 | 3.79937 | 7.699269 | 6.597111 | 4.88E-10 | 6.74E-09 | 12.37737 |
| VIM | 3.790599 | 16.73379 | 12.3646 | 1.37E-25 | 1.03E-22 | 47.49829 |
| ISL2 | 3.788529 | 5.598641 | 6.133383 | 5.65E-09 | 6.02E-08 | 9.997308 |
| TNFRSF19 | 3.787726 | 11.0165 | 9.531995 | 1.39E-17 | 1.36E-15 | 29.38289 |
| 14-Sep | 3.785492 | 4.414766 | 3.63377 | 0.000368 | 0.001243 | -0.6165 |
| GBX2 | 3.781711 | 5.99464 | 4.12281 | 5.79E-05 | 0.000238 | 1.121501 |
| LOX | 3.775987 | 10.42891 | 5.240045 | 4.62E-07 | 3.13E-06 | 5.739058 |
| VNN1 | 3.766701 | 5.588865 | 7.038681 | 4.33E-11 | 7.84E-10 | 14.73837 |
| NACA2 | 3.763272 | 5.47323 | 11.89689 | 3.01E-24 | 1.34E-21 | 44.46094 |
| ERCC6L | 3.761951 | 6.659686 | 7.679394 | 1.12E-12 | 3.05E-11 | 18.30955 |
| CAPN6 | 3.760251 | 4.133877 | 3.723669 | 0.000265 | 0.00093 | -0.3109 |
| HPD | 3.760202 | 6.508159 | 5.075327 | 9.89E-07 | 6.19E-06 | 5.00577 |
| EXO1 | 3.760098 | 8.108606 | 7.863162 | 3.81E-13 | 1.16E-11 | 19.36233 |
| COL4A2 | 3.751032 | 13.90087 | 7.503447 | 3.10E-12 | 7.48E-11 | 17.31299 |
| GDF15 | 3.749236 | 8.606873 | 5.163591 | 6.59E-07 | 4.31E-06 | 5.396576 |
| CLEC12A | 3.745881 | 5.514308 | 5.169282 | 6.42E-07 | 4.21E-06 | 5.421941 |
| SERPINH1 | 3.74051 | 12.63809 | 9.81216 | 2.34E-18 | 2.81E-16 | 31.12952 |
| SIGLEC7 | 3.729969 | 7.192395 | 7.189671 | 1.86E-11 | 3.69E-10 | 15.56521 |
| MEX3A | 3.718978 | 10.26648 | 6.236807 | 3.30E-09 | 3.74E-08 | 10.51876 |
| PTTG1 | 3.717354 | 10.3144 | 9.020015 | 3.44E-16 | 2.41E-14 | 26.23237 |
| MSR1 | 3.713701 | 11.38782 | 7.316044 | 9.07E-12 | 1.94E-10 | 16.26444 |
| S100A4 | 3.707711 | 10.00684 | 6.262867 | 2.88E-09 | 3.30E-08 | 10.65103 |
| DDIAS | 3.706227 | 8.123932 | 9.113564 | 1.92E-16 | 1.42E-14 | 26.80368 |
| EIF4EBP1 | 3.689472 | 10.61959 | 12.05746 | 1.04E-24 | 5.12E-22 | 45.50279 |
| TLX1 | 3.687423 | 3.852402 | 3.251196 | 0.001381 | 0.004033 | -1.84444 |
| IGFBP3 | 3.681848 | 12.96188 | 4.872948 | 2.47E-06 | 1.41E-05 | 4.128666 |
| CDK2 | 3.678344 | 10.51602 | 13.89495 | 5.44E-30 | 1.53E-26 | 57.45248 |
| SERINC2 | 3.666519 | 8.889507 | 6.345948 | 1.86E-09 | 2.24E-08 | 11.07498 |
| COL8A1 | 3.653605 | 9.535747 | 4.177072 | 4.67E-05 | 0.000197 | 1.325525 |
| TERT | 3.651711 | 4.491128 | 4.22154 | 3.91E-05 | 0.000168 | 1.494343 |
| CCL18 | 3.640274 | 4.97197 | 2.991643 | 0.003179 | 0.008486 | -2.60867 |
| AQP1 | 3.637353 | 14.60106 | 4.665872 | 6.12E-06 | 3.16E-05 | 3.25931 |
| TBX15 | 3.636579 | 8.519243 | 7.142764 | 2.42E-11 | 4.65E-10 | 15.30732 |
| ADAMTS12 | 3.629091 | 7.525522 | 5.615333 | 7.66E-08 | 6.20E-07 | 7.472018 |
| HOXB8 | 3.624572 | 3.649349 | 3.063501 | 0.002537 | 0.006933 | -2.40278 |
| ENPEP | 3.624569 | 9.088946 | 8.217637 | 4.64E-14 | 1.83E-12 | 21.42529 |
| MACC1 | 3.620513 | 5.437171 | 7.926607 | 2.62E-13 | 8.44E-12 | 19.72853 |
| CXCL13 | 3.619468 | 4.61415 | 3.066601 | 0.002512 | 0.006875 | -2.3938 |
| GAS2L3 | 3.616359 | 9.100316 | 8.792082 | 1.41E-15 | 8.40E-14 | 24.84924 |
| SIX6 | 3.610461 | 3.626686 | 2.876752 | 0.004522 | 0.011539 | -2.92869 |
| OR51E1 | 3.601572 | 5.712459 | 4.547039 | 1.02E-05 | 5.04E-05 | 2.773634 |
| LOXL2 | 3.600482 | 10.88405 | 6.281511 | 2.61E-09 | 3.03E-08 | 10.74586 |
| TGFB1I1 | 3.59941 | 10.53838 | 10.914 | 1.91E-21 | 5.36E-19 | 38.11834 |
| CYTL1 | 3.598914 | 9.075954 | 6.358358 | 1.74E-09 | 2.11E-08 | 11.1386 |
| RAD51AP1 | 3.598775 | 8.614462 | 9.464482 | 2.13E-17 | 1.98E-15 | 28.96424 |
| HOXB5 | 3.58664 | 3.713953 | 3.384445 | 0.000882 | 0.002693 | -1.43027 |
| SLAMF9 | 3.585899 | 4.794846 | 4.187547 | 4.48E-05 | 0.00019 | 1.36516 |
| MMP14 | 3.58122 | 13.0193 | 7.57569 | 2.04E-12 | 5.19E-11 | 17.72079 |
| CD48 | 3.577846 | 7.816376 | 5.966641 | 1.33E-08 | 1.29E-07 | 9.168448 |
| FPR2 | 3.577639 | 5.823235 | 4.344267 | 2.37E-05 | 0.000108 | 1.967763 |
| SERPINA5 | 3.575588 | 8.139711 | 5.231148 | 4.81E-07 | 3.24E-06 | 5.699016 |
| CD44 | 3.571957 | 14.35274 | 7.186262 | 1.89E-11 | 3.75E-10 | 15.54644 |
| FN1 | 3.571285 | 15.59407 | 7.451305 | 4.18E-12 | 9.82E-11 | 17.01989 |
| TMEM150B | 3.570822 | 5.039551 | 6.200594 | 3.99E-09 | 4.44E-08 | 10.33555 |
| C1orf158 | 3.569809 | 5.084597 | 3.148728 | 0.001931 | 0.005437 | -2.15292 |
| RNASE2 | 3.566589 | 8.145816 | 5.312785 | 3.28E-07 | 2.31E-06 | 6.068285 |
| MMP2 | 3.558009 | 12.2669 | 7.240601 | 1.39E-11 | 2.86E-10 | 15.84623 |
| SFRP4 | 3.556613 | 10.22272 | 5.916726 | 1.71E-08 | 1.61E-07 | 8.92323 |
| LRRC15 | 3.555416 | 4.784124 | 3.635849 | 0.000365 | 0.001235 | -0.6095 |
| H3F3C | 3.551939 | 6.187979 | 12.02241 | 1.31E-24 | 6.00E-22 | 45.27528 |
| HOXD3 | 3.551579 | 3.88954 | 4.954185 | 1.71E-06 | 1.02E-05 | 4.477537 |
| SOCS1 | 3.54906 | 7.15607 | 6.522392 | 7.29E-10 | 9.64E-09 | 11.98672 |
| SLPI | 3.544245 | 8.596399 | 3.746238 | 0.000244 | 0.000867 | -0.23317 |
| FAM183A | 3.544023 | 5.057791 | 3.560595 | 0.000478 | 0.001563 | -0.86052 |
| NEK2 | 3.532597 | 8.480642 | 6.727866 | 2.40E-10 | 3.59E-09 | 13.0673 |
| CNGA3 | 3.52803 | 9.597115 | 4.489803 | 1.30E-05 | 6.26E-05 | 2.543213 |
| MS4A4E | 3.527826 | 4.537314 | 6.268532 | 2.80E-09 | 3.22E-08 | 10.67982 |
| FGFBP2 | 3.526283 | 6.686588 | 4.012016 | 8.94E-05 | 0.000352 | 0.711731 |
| NES | 3.525281 | 14.70056 | 7.559623 | 2.24E-12 | 5.61E-11 | 17.62992 |
| C7orf57 | 3.519014 | 6.088102 | 3.567415 | 0.000466 | 0.00153 | -0.83795 |
| CARD16 | 3.512992 | 8.031817 | 6.379258 | 1.56E-09 | 1.91E-08 | 11.24593 |
| BARHL1 | 3.498382 | 3.53938 | 4.612464 | 7.70E-06 | 3.90E-05 | 3.03982 |
| MTFR2 | 3.497337 | 6.527044 | 9.040863 | 3.02E-16 | 2.15E-14 | 26.35951 |
| IGF2BP2 | 3.49603 | 8.721075 | 4.391362 | 1.95E-05 | 9.05E-05 | 2.152325 |
| FAM156B | 3.492606 | 3.930006 | 10.56286 | 1.87E-20 | 3.72E-18 | 35.87354 |
| AC068775.1 | 3.479912 | 5.79281 | 9.101782 | 2.07E-16 | 1.52E-14 | 26.73161 |
| WDR38 | 3.47556 | 5.378595 | 3.618889 | 0.000388 | 0.001307 | -0.66647 |
| EVX2 | 3.470528 | 3.499731 | 4.528381 | 1.10E-05 | 5.42E-05 | 2.698268 |
| LMNB1 | 3.458054 | 10.93004 | 8.081817 | 1.04E-13 | 3.77E-12 | 20.63003 |
| ASIP | 3.457444 | 5.340179 | 6.907211 | 8.98E-11 | 1.50E-09 | 14.02633 |
| GAPT | 3.455776 | 7.262334 | 6.575163 | 5.49E-10 | 7.49E-09 | 12.26235 |
| SOX11 | 3.452118 | 10.86592 | 5.309744 | 3.33E-07 | 2.34E-06 | 6.054458 |
| VGLL2 | 3.449569 | 4.022627 | 3.386222 | 0.000877 | 0.002678 | -1.42465 |
| PLEK2 | 3.440653 | 6.84162 | 7.044283 | 4.20E-11 | 7.63E-10 | 14.76888 |
| COL1A2 | 3.438394 | 13.41721 | 4.489051 | 1.30E-05 | 6.27E-05 | 2.540202 |
| TMEM71 | 3.43547 | 7.585188 | 6.001316 | 1.11E-08 | 1.10E-07 | 9.33959 |
| TRPM8 | 3.431858 | 7.177185 | 4.848101 | 2.75E-06 | 1.55E-05 | 4.022833 |
| C10orf55 | 3.426992 | 4.662602 | 5.90798 | 1.79E-08 | 1.68E-07 | 8.880401 |
| IQGAP3 | 3.425652 | 9.299985 | 7.430351 | 4.72E-12 | 1.09E-10 | 16.90239 |
| PRPH | 3.423572 | 7.667218 | 6.455643 | 1.04E-09 | 1.33E-08 | 11.64002 |
| NMB | 3.420701 | 11.35417 | 6.142659 | 5.38E-09 | 5.78E-08 | 10.04385 |
| HIST2H4A | 3.418821 | 3.440553 | 8.052494 | 1.24E-13 | 4.38E-12 | 20.4591 |
| TCF19 | 3.418064 | 10.01601 | 9.184768 | 1.23E-16 | 9.50E-15 | 27.23989 |
| BPIFB2 | 3.417631 | 3.857983 | 3.750085 | 0.000241 | 0.000855 | -0.21989 |
| NRM | 3.403472 | 9.940686 | 11.88759 | 3.20E-24 | 1.40E-21 | 44.4006 |
| ALOX15B | 3.400918 | 6.770108 | 4.001649 | 9.30E-05 | 0.000365 | 0.673861 |
| ETV4 | 3.398506 | 9.181019 | 4.811858 | 3.23E-06 | 1.78E-05 | 3.869195 |
| MXRA5 | 3.396836 | 9.685114 | 4.426842 | 1.69E-05 | 7.93E-05 | 2.292416 |
| SRPX | 3.395041 | 11.50378 | 5.414618 | 2.02E-07 | 1.50E-06 | 6.534638 |
| RPE65 | 3.393609 | 8.475151 | 4.914856 | 2.05E-06 | 1.19E-05 | 4.308095 |
| ADGRG7 | 3.393165 | 3.29566 | 3.06458 | 0.002528 | 0.006913 | -2.39966 |
| LRRN4CL | 3.39306 | 8.490617 | 5.16737 | 6.47E-07 | 4.24E-06 | 5.413417 |
| FMOD | 3.391014 | 10.99314 | 4.262663 | 3.31E-05 | 0.000145 | 1.651756 |
| EMP3 | 3.387849 | 11.8614 | 6.662825 | 3.42E-10 | 4.92E-09 | 12.72311 |
| ITPRIPL1 | 3.386493 | 8.766539 | 9.456489 | 2.24E-17 | 2.07E-15 | 28.91474 |
| APOBEC3C | 3.380794 | 10.49659 | 7.911393 | 2.87E-13 | 9.10E-12 | 19.64059 |
| DEPDC1B | 3.37486 | 8.070809 | 6.732646 | 2.34E-10 | 3.52E-09 | 13.09267 |
| BRIP1 | 3.368037 | 7.806605 | 7.004469 | 5.24E-11 | 9.20E-10 | 14.55236 |
| CENPF | 3.367729 | 10.65466 | 6.125186 | 5.89E-09 | 6.26E-08 | 9.956221 |
| CRISPLD1 | 3.363247 | 12.09536 | 7.45546 | 4.09E-12 | 9.62E-11 | 17.0432 |
| DMBX1 | 3.35759 | 4.084734 | 3.94163 | 0.000117 | 0.000449 | 0.45622 |
| KIAA0040 | 3.351893 | 10.77737 | 7.351431 | 7.41E-12 | 1.63E-10 | 16.46139 |
| CHRNA1 | 3.350344 | 7.028435 | 3.218435 | 0.001538 | 0.004434 | -1.94402 |
| CA12 | 3.347403 | 11.34845 | 4.822064 | 3.09E-06 | 1.72E-05 | 3.912373 |
| SPARC | 3.344128 | 17.112 | 11.26363 | 1.94E-22 | 6.57E-20 | 40.3661 |
| FOXD1 | 3.337201 | 8.237841 | 6.279231 | 2.64E-09 | 3.06E-08 | 10.73425 |
| CHRNA9 | 3.334334 | 7.609786 | 3.578539 | 0.000448 | 0.00148 | -0.80108 |
| APOC1 | 3.332416 | 12.38986 | 6.352471 | 1.80E-09 | 2.17E-08 | 11.10841 |
| KIF15 | 3.328765 | 9.132592 | 7.011847 | 5.03E-11 | 8.88E-10 | 14.59244 |
| ITGB3 | 3.325819 | 6.876382 | 6.164534 | 4.81E-09 | 5.22E-08 | 10.15378 |
| EN2 | 3.323829 | 7.127098 | 4.75793 | 4.10E-06 | 2.20E-05 | 3.642222 |
| HSPA6 | 3.320653 | 8.970759 | 5.650703 | 6.44E-08 | 5.30E-07 | 7.639654 |
| EIF3CL | 3.320526 | 6.585217 | 10.55242 | 2.00E-20 | 3.90E-18 | 35.80701 |
| STON1 | 3.31988 | 9.47391 | 7.203818 | 1.71E-11 | 3.45E-10 | 15.64316 |
| AURKA | 3.319615 | 9.155186 | 8.694656 | 2.56E-15 | 1.44E-13 | 24.26209 |
| POLQ | 3.31678 | 7.186455 | 7.415095 | 5.15E-12 | 1.17E-10 | 16.81695 |
| TFAP2A | 3.315127 | 7.388234 | 3.899458 | 0.000138 | 0.000519 | 0.304935 |
| MEST | 3.312685 | 12.82059 | 5.865994 | 2.21E-08 | 2.03E-07 | 8.675392 |
| ID3 | 3.309 | 13.35131 | 8.031849 | 1.41E-13 | 4.88E-12 | 20.33894 |
| S100A8 | 3.305813 | 8.907481 | 4.077871 | 6.91E-05 | 0.00028 | 0.954186 |
| HNF4G | 3.304889 | 7.458032 | 6.921585 | 8.30E-11 | 1.40E-09 | 14.10382 |
| CCDC80 | 3.294618 | 12.66716 | 8.613454 | 4.22E-15 | 2.22E-13 | 23.77463 |
| TK1 | 3.290606 | 9.603279 | 7.915849 | 2.79E-13 | 8.88E-12 | 19.66634 |
| FBXO43 | 3.287886 | 5.396799 | 6.062546 | 8.13E-09 | 8.32E-08 | 9.64338 |
| FBXO39 | 3.287716 | 5.079988 | 5.02797 | 1.23E-06 | 7.51E-06 | 4.798144 |
| CXCR2 | 3.286759 | 6.042507 | 5.863368 | 2.24E-08 | 2.05E-07 | 8.662602 |
| KLHDC8A | 3.283531 | 12.18049 | 5.573258 | 9.40E-08 | 7.46E-07 | 7.273552 |
| VSIG4 | 3.282326 | 12.32552 | 5.484136 | 1.45E-07 | 1.11E-06 | 6.856597 |
| F2RL2 | 3.276778 | 6.614668 | 4.981402 | 1.52E-06 | 9.09E-06 | 4.595383 |
| ATP5EP2 | 3.273696 | 6.180745 | 9.615159 | 8.20E-18 | 8.48E-16 | 29.89981 |
| CELSR1 | 3.261828 | 8.576518 | 4.516915 | 1.16E-05 | 5.65E-05 | 2.652074 |
| ATP6V0D2 | 3.256506 | 3.845029 | 4.338575 | 2.43E-05 | 0.00011 | 1.945566 |
| LBX1 | 3.256056 | 3.432948 | 3.386677 | 0.000875 | 0.002675 | -1.42321 |
| NID1 | 3.252423 | 12.13491 | 7.632295 | 1.47E-12 | 3.86E-11 | 18.04166 |
| S100A9 | 3.247621 | 10.16873 | 4.177053 | 4.67E-05 | 0.000197 | 1.325451 |
| LIF | 3.246899 | 9.016505 | 3.800557 | 0.0002 | 0.000725 | -0.04449 |
| CDKN2C | 3.244492 | 11.72267 | 6.440446 | 1.13E-09 | 1.43E-08 | 11.56138 |
| CLIC1 | 3.244279 | 13.1285 | 9.107045 | 2.00E-16 | 1.47E-14 | 26.7638 |
| PLEKHA4 | 3.24367 | 11.42244 | 7.148319 | 2.34E-11 | 4.52E-10 | 15.33782 |
| PLK1 | 3.243566 | 9.987881 | 6.974564 | 6.19E-11 | 1.07E-09 | 14.39018 |
| RPLP0 | 3.243413 | 15.89414 | 12.85131 | 5.45E-27 | 4.66E-24 | 50.66485 |
| EME1 | 3.236713 | 7.841929 | 7.599162 | 1.78E-12 | 4.57E-11 | 17.8537 |
| CMTM3 | 3.236642 | 11.72621 | 13.72338 | 1.69E-29 | 3.70E-26 | 56.33816 |
| PIF1 | 3.230465 | 7.720591 | 6.789886 | 1.71E-10 | 2.67E-09 | 13.39731 |
| TIMP1 | 3.227718 | 13.70885 | 5.40445 | 2.12E-07 | 1.57E-06 | 6.48779 |
| GBP1 | 3.219631 | 11.49138 | 6.031146 | 9.55E-09 | 9.61E-08 | 9.487338 |
| GINS2 | 3.217829 | 9.346624 | 7.388492 | 6.00E-12 | 1.34E-10 | 16.66818 |
| ADGRE1 | 3.214986 | 6.708467 | 4.872767 | 2.47E-06 | 1.41E-05 | 4.127892 |
| CD248 | 3.212567 | 10.41256 | 6.392902 | 1.45E-09 | 1.79E-08 | 11.31611 |
| HMGA2 | 3.211128 | 5.74824 | 3.075276 | 0.002444 | 0.006708 | -2.36863 |
| HS3ST3B1 | 3.209459 | 8.302624 | 4.243099 | 3.58E-05 | 0.000156 | 1.576712 |
| ARHGAP11A | 3.208643 | 9.367937 | 8.535064 | 6.80E-15 | 3.41E-13 | 23.30577 |
| GBP5 | 3.207628 | 7.456239 | 5.200528 | 5.55E-07 | 3.68E-06 | 5.561581 |
| SEC61G | 3.205191 | 13.02038 | 3.523764 | 0.000544 | 0.001754 | -0.98172 |
| F2RL3 | 3.197611 | 5.411963 | 5.282383 | 3.78E-07 | 2.62E-06 | 5.930286 |
| SAA2-SAA4 | 3.192405 | 3.346738 | 2.295516 | 0.0229 | 0.047933 | -4.37033 |
| PITX2 | 3.190562 | 3.391371 | 2.638149 | 0.009094 | 0.021358 | -3.55672 |
| SLC47A2 | 3.189736 | 8.674697 | 4.437094 | 1.62E-05 | 7.63E-05 | 2.333063 |
| CD99 | 3.188313 | 14.49173 | 8.473749 | 9.88E-15 | 4.66E-13 | 22.94024 |
| SLAMF8 | 3.187346 | 8.386194 | 5.403665 | 2.13E-07 | 1.57E-06 | 6.484177 |
| TEAD2 | 3.171745 | 10.01229 | 6.530804 | 6.97E-10 | 9.26E-09 | 12.03057 |
| ANXA2R | 3.171084 | 7.72985 | 6.481418 | 9.08E-10 | 1.18E-08 | 11.77364 |
| C15orf48 | 3.166613 | 6.11771 | 4.471314 | 1.40E-05 | 6.70E-05 | 2.469275 |
| LAMB4 | 3.15923 | 4.725534 | 7.340281 | 7.90E-12 | 1.73E-10 | 16.39928 |
| CDCA7L | 3.157399 | 10.37123 | 7.365065 | 6.86E-12 | 1.52E-10 | 16.5374 |
| ANG | 3.15666 | 7.452679 | 7.156849 | 2.23E-11 | 4.33E-10 | 15.38466 |
| LPL | 3.15097 | 12.18336 | 5.429856 | 1.88E-07 | 1.40E-06 | 6.604964 |
| GPX7 | 3.147943 | 9.986861 | 10.04167 | 5.40E-19 | 7.53E-17 | 32.57074 |
| AJUBA | 3.143998 | 8.68459 | 8.073752 | 1.10E-13 | 3.90E-12 | 20.58299 |
| TAGLN2 | 3.13549 | 14.05451 | 7.665434 | 1.21E-12 | 3.26E-11 | 18.23007 |
| SLC16A4 | 3.125343 | 9.947176 | 6.640719 | 3.86E-10 | 5.49E-09 | 12.60658 |
| CSRP2 | 3.123647 | 12.12742 | 7.14454 | 2.39E-11 | 4.61E-10 | 15.31707 |
| ITGA4 | 3.118994 | 8.48955 | 6.094215 | 6.91E-09 | 7.21E-08 | 9.801285 |
| CPVL | 3.115156 | 11.26551 | 6.219701 | 3.61E-09 | 4.06E-08 | 10.43214 |
| VNN2 | 3.114154 | 6.888251 | 5.547024 | 1.07E-07 | 8.38E-07 | 7.150334 |
| CD207 | 3.112752 | 3.738235 | 4.694834 | 5.40E-06 | 2.82E-05 | 3.379153 |
| AGMO | 3.11144 | 7.489163 | 4.314112 | 2.68E-05 | 0.00012 | 1.850429 |
| FOLR1 | 3.110389 | 6.939655 | 4.165124 | 4.90E-05 | 0.000206 | 1.280414 |
| SLC34A2 | 3.108441 | 6.078814 | 4.11486 | 5.98E-05 | 0.000245 | 1.09179 |
| LUM | 3.105651 | 9.59462 | 3.84196 | 0.000171 | 0.00063 | 0.100872 |
| FAM46A | 3.103678 | 10.20286 | 8.043239 | 1.31E-13 | 4.61E-12 | 20.40522 |
| CFI | 3.09932 | 10.78821 | 5.35714 | 2.66E-07 | 1.92E-06 | 6.27064 |
| TACC3 | 3.095758 | 10.72317 | 7.449754 | 4.22E-12 | 9.89E-11 | 17.01118 |
| IKBIP | 3.090588 | 9.864649 | 10.22128 | 1.70E-19 | 2.60E-17 | 33.70452 |
| CDK4 | 3.084824 | 12.74019 | 4.108459 | 6.13E-05 | 0.000251 | 1.067907 |
| MAP3K7CL | 3.081513 | 8.999183 | 5.570666 | 9.52E-08 | 7.54E-07 | 7.261362 |
| AC113404.3 | 3.077962 | 5.773596 | 8.322789 | 2.46E-14 | 1.04E-12 | 22.04491 |
| HLA-DRA | 3.07778 | 14.77236 | 6.0306 | 9.58E-09 | 9.63E-08 | 9.484633 |
| TYMS | 3.077314 | 10.85492 | 7.276503 | 1.14E-11 | 2.37E-10 | 16.04497 |
| CBX2 | 3.07612 | 8.803613 | 5.755097 | 3.84E-08 | 3.34E-07 | 8.138613 |
| C1orf226 | 3.069284 | 10.22558 | 8.135353 | 7.59E-14 | 2.82E-12 | 20.94279 |
| IGFBP7 | 3.068722 | 14.71141 | 7.732255 | 8.22E-13 | 2.30E-11 | 18.61116 |
| CLCF1 | 3.066603 | 8.110114 | 5.11467 | 8.26E-07 | 5.26E-06 | 5.179357 |
| CLEC18C | 3.064649 | 4.29155 | 5.195778 | 5.67E-07 | 3.76E-06 | 5.540315 |
| CXCL11 | 3.063365 | 6.580562 | 3.592415 | 0.000426 | 0.001419 | -0.75493 |
| EEF1AKMT3 | 3.059691 | 9.529769 | 4.728939 | 4.65E-06 | 2.47E-05 | 3.521015 |
| TMEM37 | 3.057971 | 8.200402 | 6.427736 | 1.21E-09 | 1.52E-08 | 11.49571 |
| PYGL | 3.057448 | 11.49682 | 8.259162 | 3.61E-14 | 1.47E-12 | 21.66958 |
| HIST1H3H | 3.054326 | 5.744335 | 5.459677 | 1.63E-07 | 1.23E-06 | 6.742992 |
| ESM1 | 3.05259 | 8.267693 | 3.736084 | 0.000253 | 0.000895 | -0.26819 |
| VEPH1 | 3.050253 | 6.535575 | 3.689324 | 0.000301 | 0.001039 | -0.4284 |
| HNRNPCL1 | 3.04797 | 3.460647 | 8.524645 | 7.25E-15 | 3.60E-13 | 23.24359 |
| S100A11 | 3.046891 | 12.37928 | 6.795796 | 1.66E-10 | 2.59E-09 | 13.42885 |
| H3F3A | 3.046442 | 11.32586 | 13.06396 | 1.33E-27 | 1.46E-24 | 52.04896 |
| CENPI | 3.045859 | 7.394883 | 7.157344 | 2.23E-11 | 4.33E-10 | 15.38739 |
| S100A10 | 3.044402 | 12.66437 | 5.179748 | 6.11E-07 | 4.03E-06 | 5.468648 |
| MTHFD2 | 3.040476 | 11.10678 | 8.122651 | 8.18E-14 | 3.01E-12 | 20.86851 |
| LAMP3 | 3.039742 | 5.923625 | 6.588409 | 5.12E-10 | 7.03E-09 | 12.33174 |
| NPIPB13 | 3.038493 | 5.288391 | 6.845738 | 1.26E-10 | 2.02E-09 | 13.69599 |
| CD93 | 3.034626 | 11.3682 | 6.623687 | 4.23E-10 | 5.93E-09 | 12.51695 |
| CD70 | 3.031419 | 4.682561 | 3.154463 | 0.001895 | 0.005343 | -2.13589 |
| FBLIM1 | 3.026317 | 9.907621 | 5.177185 | 6.19E-07 | 4.07E-06 | 5.457203 |
| EBF2 | 3.026238 | 5.621456 | 4.559869 | 9.64E-06 | 4.79E-05 | 2.825597 |
| CDH15 | 3.021569 | 5.170132 | 3.510651 | 0.00057 | 0.001827 | -1.02461 |
| F13A1 | 3.021296 | 10.73246 | 2.92631 | 0.003889 | 0.010133 | -2.79204 |
| CASP4 | 3.019734 | 9.986572 | 7.194331 | 1.81E-11 | 3.61E-10 | 15.59088 |
| CYP27B1 | 3.018036 | 6.779804 | 3.678343 | 0.000313 | 0.001076 | -0.46577 |
| WDR76 | 3.014507 | 9.38896 | 8.263417 | 3.52E-14 | 1.43E-12 | 21.69464 |
| MMP13 | 3.012697 | 3.402571 | 2.589094 | 0.01044 | 0.024098 | -3.67964 |
| HS3ST3A1 | 3.004528 | 5.489039 | 3.439834 | 0.000729 | 0.002276 | -1.25383 |
| EDA2R | 3.004427 | 8.25395 | 4.698973 | 5.30E-06 | 2.77E-05 | 3.396328 |
| SLAMF7 | 2.999711 | 5.615038 | 5.079537 | 9.70E-07 | 6.08E-06 | 5.024299 |
| ERP27 | 2.999155 | 5.663282 | 5.089208 | 9.28E-07 | 5.84E-06 | 5.066904 |
| IGF2BP1 | 2.997079 | 3.323421 | 3.657537 | 0.000337 | 0.001151 | -0.53632 |
| MAP1LC3C | 2.982278 | 6.36988 | 4.588088 | 8.55E-06 | 4.29E-05 | 2.940297 |
| HIST1H2AG | 2.981942 | 6.112545 | 5.943216 | 1.50E-08 | 1.43E-07 | 9.053196 |
| AC112229.3 | 2.980709 | 4.476032 | 3.889078 | 0.000143 | 0.000538 | 0.267908 |
| TLR8 | 2.979507 | 6.889228 | 4.541734 | 1.04E-05 | 5.14E-05 | 2.752182 |
| ANKRD22 | 2.974792 | 7.41795 | 4.777667 | 3.76E-06 | 2.04E-05 | 3.725066 |
| S100A2 | 2.971903 | 7.039072 | 5.812278 | 2.89E-08 | 2.59E-07 | 8.414529 |
| ACTG2 | 2.970139 | 7.80397 | 4.187639 | 4.48E-05 | 0.00019 | 1.36551 |
| GBP2 | 2.969143 | 11.19932 | 6.206827 | 3.86E-09 | 4.31E-08 | 10.36704 |
| ZNF90 | 2.967959 | 7.101014 | 7.075888 | 3.52E-11 | 6.50E-10 | 14.94123 |
| GPR141 | 2.96133 | 4.430596 | 4.820368 | 3.11E-06 | 1.73E-05 | 3.905192 |
| EGFLAM | 2.960295 | 7.960327 | 5.271758 | 3.98E-07 | 2.74E-06 | 5.882195 |
| TNFSF13B | 2.956736 | 9.453749 | 5.995797 | 1.14E-08 | 1.13E-07 | 9.312307 |
| EFEMP1 | 2.955922 | 13.34959 | 4.83464 | 2.92E-06 | 1.64E-05 | 3.965669 |
| AFAP1L1 | 2.951958 | 9.821604 | 6.693657 | 2.90E-10 | 4.23E-09 | 12.88603 |
| PRR11 | 2.951597 | 9.463561 | 10.3253 | 8.71E-20 | 1.50E-17 | 34.36339 |
| CLEC18B | 2.950411 | 7.965888 | 5.24425 | 4.53E-07 | 3.07E-06 | 5.758002 |
| ASPN | 2.949361 | 7.020076 | 4.4047 | 1.85E-05 | 8.60E-05 | 2.204884 |
| TNFRSF10C | 2.940041 | 6.596477 | 6.152969 | 5.10E-09 | 5.51E-08 | 10.09564 |
| ANXA5 | 2.938155 | 14.63064 | 9.517179 | 1.52E-17 | 1.48E-15 | 29.29093 |
| PPP1R14B | 2.935754 | 11.66051 | 11.12252 | 4.88E-22 | 1.55E-19 | 39.45755 |
| XKR5 | 2.935659 | 4.248653 | 4.237005 | 3.67E-05 | 0.000159 | 1.553396 |
| PPIC | 2.933391 | 9.6032 | 7.837083 | 4.45E-13 | 1.33E-11 | 19.21221 |
| CENPH | 2.929976 | 9.004042 | 7.459607 | 3.99E-12 | 9.42E-11 | 17.06649 |
| MIA | 2.929395 | 3.679339 | 3.682736 | 0.000308 | 0.001062 | -0.45083 |
| PLAC8 | 2.929248 | 6.653721 | 4.123539 | 5.78E-05 | 0.000238 | 1.124227 |
| LBP | 2.929164 | 4.018113 | 2.716509 | 0.007266 | 0.017597 | -3.35595 |
| FANCD2 | 2.928203 | 9.389569 | 7.809889 | 5.22E-13 | 1.53E-11 | 19.05592 |
| BCL2L12 | 2.927494 | 8.475125 | 7.911102 | 2.87E-13 | 9.10E-12 | 19.63891 |
| GATA3 | 2.922762 | 4.752369 | 4.000956 | 9.33E-05 | 0.000366 | 0.671334 |
| CERKL | 2.920271 | 8.359619 | 6.308243 | 2.27E-09 | 2.68E-08 | 10.88214 |
| TRIM5 | 2.917729 | 9.775417 | 8.87389 | 8.51E-16 | 5.38E-14 | 25.34418 |
| MGP | 2.917411 | 11.69582 | 4.300433 | 2.84E-05 | 0.000126 | 1.797418 |
| STC2 | 2.916941 | 9.160853 | 4.562756 | 9.52E-06 | 4.74E-05 | 2.837308 |
| SHCBP1 | 2.915802 | 8.913115 | 8.133531 | 7.67E-14 | 2.85E-12 | 20.93214 |
| HIST1H3B | 2.910121 | 2.826496 | 3.891033 | 0.000142 | 0.000535 | 0.274875 |
| FCGR3B | 2.909618 | 6.279233 | 4.344104 | 2.37E-05 | 0.000108 | 1.967129 |
| CASP1 | 2.907883 | 9.783153 | 6.451789 | 1.06E-09 | 1.35E-08 | 11.62007 |
| C4orf47 | 2.904624 | 7.280617 | 6.071112 | 7.78E-09 | 7.98E-08 | 9.686038 |
| SPSB4 | 2.903464 | 8.982845 | 5.325018 | 3.10E-07 | 2.20E-06 | 6.123974 |
| POLE2 | 2.897221 | 7.594194 | 6.774536 | 1.86E-10 | 2.87E-09 | 13.31547 |
| TGIF1 | 2.896259 | 10.92175 | 11.43809 | 6.16E-23 | 2.38E-20 | 41.49154 |
| FCMR | 2.896084 | 6.812075 | 6.639819 | 3.88E-10 | 5.51E-09 | 12.60184 |
| CD58 | 2.889786 | 9.413744 | 7.181688 | 1.94E-11 | 3.82E-10 | 15.52125 |
| NLRP11 | 2.88779 | 3.075265 | 3.274841 | 0.001276 | 0.003753 | -1.77202 |
| NODAL | 2.887553 | 6.207619 | 3.898247 | 0.000138 | 0.000521 | 0.300613 |
| SMIM3 | 2.885088 | 11.36738 | 5.771802 | 3.54E-08 | 3.09E-07 | 8.219027 |
| FKBP10 | 2.883748 | 13.00424 | 9.762027 | 3.22E-18 | 3.75E-16 | 30.81591 |
| SLC43A3 | 2.883422 | 10.38754 | 8.711136 | 2.32E-15 | 1.32E-13 | 24.36123 |
| EVA1A | 2.882998 | 6.037745 | 3.454074 | 0.000694 | 0.002181 | -1.20806 |
| PCOLCE | 2.877714 | 10.83884 | 4.413321 | 1.78E-05 | 8.32E-05 | 2.238922 |
| TSPAN6 | 2.87521 | 11.7872 | 9.554804 | 1.20E-17 | 1.20E-15 | 29.52453 |
| RBM47 | 2.873694 | 9.091628 | 6.25474 | 3.01E-09 | 3.43E-08 | 10.60974 |
| DENND2D | 2.873128 | 8.190275 | 6.493054 | 8.53E-10 | 1.11E-08 | 11.83407 |
| IGFBP1 | 2.870258 | 6.092305 | 4.488607 | 1.30E-05 | 6.28E-05 | 2.538425 |
| SMO | 2.865962 | 11.31286 | 8.497133 | 8.57E-15 | 4.15E-13 | 23.07952 |
| SPP1 | 2.865799 | 16.6074 | 4.382641 | 2.03E-05 | 9.36E-05 | 2.118029 |
| BEST3 | 2.865662 | 8.856781 | 4.424947 | 1.70E-05 | 7.98E-05 | 2.284909 |
| HIST2H2AA3 | 2.864263 | 2.781956 | 5.253712 | 4.33E-07 | 2.95E-06 | 5.800666 |
| FAM129A | 2.861113 | 10.66577 | 5.921077 | 1.67E-08 | 1.58E-07 | 8.944551 |
| HLA-DQA1 | 2.861004 | 11.21086 | 4.867684 | 2.53E-06 | 1.44E-05 | 4.106209 |
| RDH10 | 2.859914 | 11.16035 | 5.410503 | 2.06E-07 | 1.53E-06 | 6.515672 |
| CLDN7 | 2.856823 | 5.891026 | 7.324379 | 8.65E-12 | 1.86E-10 | 16.31079 |
| MARVELD3 | 2.855978 | 6.360172 | 3.694677 | 0.000295 | 0.001022 | -0.41015 |
| SIT1 | 2.853975 | 4.253064 | 4.815664 | 3.18E-06 | 1.76E-05 | 3.88529 |
| CXCR4 | 2.847231 | 11.34372 | 6.867235 | 1.12E-10 | 1.82E-09 | 13.81132 |
| RUNX3 | 2.843285 | 7.953187 | 7.409396 | 5.32E-12 | 1.20E-10 | 16.78506 |
| MCOLN2 | 2.83861 | 5.354464 | 4.796434 | 3.46E-06 | 1.90E-05 | 3.804078 |
| CEACAM4 | 2.835888 | 4.23125 | 4.37738 | 2.07E-05 | 9.54E-05 | 2.097364 |
| PROS1 | 2.835574 | 11.17711 | 6.080604 | 7.41E-09 | 7.67E-08 | 9.733351 |
| JCHAIN | 2.835403 | 5.8608 | 2.456458 | 0.015016 | 0.033152 | -4.00128 |
| PTPN7 | 2.832385 | 7.980374 | 6.717395 | 2.54E-10 | 3.78E-09 | 13.01176 |
| HIST2H2AA4 | 2.831686 | 2.750316 | 5.726723 | 4.43E-08 | 3.78E-07 | 8.002384 |
| FCGR2A | 2.83044 | 11.31413 | 5.82798 | 2.67E-08 | 2.41E-07 | 8.490617 |
| LBX2 | 2.829376 | 4.429274 | 4.740306 | 4.43E-06 | 2.36E-05 | 3.568469 |
| AC009336.2 | 2.828439 | 2.747162 | 3.602036 | 0.000412 | 0.001378 | -0.72285 |
| FANCI | 2.827059 | 10.3625 | 7.130386 | 2.59E-11 | 4.95E-10 | 15.23942 |
| KIF11 | 2.825564 | 9.413101 | 6.73424 | 2.32E-10 | 3.50E-09 | 13.10114 |
| ADAM12 | 2.823732 | 10.08028 | 4.739443 | 4.44E-06 | 2.37E-05 | 3.564865 |
| IL32 | 2.817485 | 9.359191 | 5.080591 | 9.66E-07 | 6.06E-06 | 5.028938 |
| EMP1 | 2.817123 | 13.31797 | 5.752677 | 3.89E-08 | 3.38E-07 | 8.126974 |
| LRR1 | 2.815985 | 8.568625 | 8.852893 | 9.68E-16 | 6.05E-14 | 25.21698 |
| ARHGAP11B | 2.81399 | 7.521736 | 6.433942 | 1.17E-09 | 1.47E-08 | 11.52776 |
| AR | 2.810557 | 9.595854 | 5.513703 | 1.26E-07 | 9.73E-07 | 6.994406 |
| CCNB1 | 2.809819 | 10.8165 | 6.672022 | 3.26E-10 | 4.71E-09 | 12.77166 |
| 1-Dec | 2.808233 | 4.52727 | 5.462929 | 1.60E-07 | 1.21E-06 | 6.758075 |
| FAM180A | 2.806526 | 6.124228 | 3.039158 | 0.00274 | 0.007423 | -2.47302 |
| FSTL1 | 2.80088 | 13.37309 | 8.180057 | 5.81E-14 | 2.23E-12 | 21.20467 |
| NAT1 | 2.800845 | 6.810115 | 7.107265 | 2.95E-11 | 5.55E-10 | 15.11276 |
| VAX2 | 2.800717 | 7.337424 | 4.844409 | 2.80E-06 | 1.58E-05 | 4.007141 |
| RPS3A | 2.800168 | 14.68252 | 8.413838 | 1.42E-14 | 6.33E-13 | 22.58412 |
| HTRA4 | 2.799209 | 3.335408 | 4.197296 | 4.31E-05 | 0.000184 | 1.402124 |
| ITGA5 | 2.798637 | 11.57511 | 6.497666 | 8.32E-10 | 1.09E-08 | 11.85804 |
| ECSCR | 2.797527 | 7.26114 | 6.240763 | 3.23E-09 | 3.67E-08 | 10.53882 |
| PLEKHG2 | 2.796244 | 11.11708 | 8.408997 | 1.46E-14 | 6.51E-13 | 22.55539 |
| FCER1G | 2.785437 | 11.40371 | 6.001625 | 1.11E-08 | 1.10E-07 | 9.341121 |
| BRCA2 | 2.78454 | 7.682882 | 7.346408 | 7.63E-12 | 1.67E-10 | 16.43341 |
| CENPE | 2.782911 | 8.471473 | 6.101847 | 6.65E-09 | 6.97E-08 | 9.839414 |
| CDCP1 | 2.775675 | 8.540159 | 4.811122 | 3.24E-06 | 1.79E-05 | 3.866083 |
| PARPBP | 2.775304 | 7.94656 | 7.51097 | 2.97E-12 | 7.18E-11 | 17.35537 |
| ADAMTS6 | 2.775131 | 7.824958 | 6.491598 | 8.60E-10 | 1.12E-08 | 11.82651 |
| MMP25 | 2.773626 | 7.586183 | 5.437471 | 1.81E-07 | 1.36E-06 | 6.640159 |
| ZIC1 | 2.771123 | 11.0162 | 6.531506 | 6.95E-10 | 9.23E-09 | 12.03423 |
| MYBPH | 2.766627 | 6.868755 | 3.189326 | 0.001692 | 0.004826 | -2.03175 |
| CHEK2 | 2.764672 | 8.350597 | 8.672416 | 2.94E-15 | 1.61E-13 | 24.1284 |
| FAM177B | 2.759817 | 6.102936 | 4.930611 | 1.91E-06 | 1.12E-05 | 4.375849 |
| TRPV4 | 2.759462 | 6.050874 | 5.899502 | 1.87E-08 | 1.75E-07 | 8.838929 |
| PRDX4 | 2.756413 | 12.2091 | 8.162411 | 6.45E-14 | 2.45E-12 | 21.10123 |
| ARHGEF39 | 2.754975 | 7.205069 | 6.599232 | 4.83E-10 | 6.68E-09 | 12.38849 |
| ACTL6A | 2.754538 | 11.04867 | 11.30279 | 1.50E-22 | 5.26E-20 | 40.61847 |
| CD1D | 2.754284 | 6.312411 | 5.50297 | 1.32E-07 | 1.02E-06 | 6.94432 |
| POTEG | 2.75425 | 3.215089 | 8.14823 | 7.02E-14 | 2.65E-12 | 21.01816 |
| CDCA4 | 2.753749 | 9.012911 | 7.543594 | 2.46E-12 | 6.11E-11 | 17.53937 |
| C8orf22 | 2.753441 | 3.851497 | 2.622136 | 0.009515 | 0.022233 | -3.59707 |
| C1R | 2.752288 | 13.16314 | 4.988658 | 1.47E-06 | 8.83E-06 | 4.626884 |
| LAMC1 | 2.749246 | 12.62749 | 7.285202 | 1.08E-11 | 2.27E-10 | 16.0932 |
| FAM83D | 2.748898 | 8.392434 | 5.049926 | 1.11E-06 | 6.87E-06 | 4.894226 |
| PRRX1 | 2.744594 | 11.95628 | 7.94611 | 2.34E-13 | 7.65E-12 | 19.84136 |
| IFI30 | 2.738364 | 5.863577 | 5.988238 | 1.19E-08 | 1.17E-07 | 9.274967 |
| MRC2 | 2.737997 | 12.75622 | 5.684539 | 5.45E-08 | 4.56E-07 | 7.800693 |
| BTN3A2 | 2.73532 | 11.21368 | 6.651749 | 3.63E-10 | 5.21E-09 | 12.6647 |
| RASEF | 2.73319 | 6.339449 | 3.290308 | 0.001212 | 0.003583 | -1.72439 |
| PSMB9 | 2.731878 | 10.77454 | 6.284778 | 2.57E-09 | 2.99E-08 | 10.7625 |
| HOXD12 | 2.731836 | 2.804301 | 3.618497 | 0.000388 | 0.001308 | -0.66778 |
| CAMP | 2.731295 | 2.65281 | 2.875238 | 0.004543 | 0.011586 | -2.93283 |
| SLAMF6 | 2.730556 | 5.087001 | 4.490549 | 1.29E-05 | 6.25E-05 | 2.546204 |
| SLC11A1 | 2.73009 | 11.03049 | 4.943661 | 1.80E-06 | 1.06E-05 | 4.432093 |
| SOX4 | 2.72979 | 12.23726 | 4.779674 | 3.73E-06 | 2.03E-05 | 3.733504 |
| COL22A1 | 2.72902 | 9.323981 | 3.760488 | 0.000232 | 0.000826 | -0.1839 |
| PTPN22 | 2.72604 | 6.190616 | 5.643797 | 6.66E-08 | 5.46E-07 | 7.606864 |
| B2M | 2.720371 | 16.49646 | 8.505672 | 8.13E-15 | 4.01E-13 | 23.13042 |
| TUBB6 | 2.718225 | 11.24289 | 5.403875 | 2.13E-07 | 1.57E-06 | 6.485143 |
| S1PR3 | 2.714799 | 11.53888 | 6.589349 | 5.09E-10 | 7.00E-09 | 12.33666 |
| AEBP1 | 2.713546 | 13.87231 | 4.368508 | 2.15E-05 | 9.84E-05 | 2.062565 |
| LY96 | 2.711163 | 8.556849 | 4.831836 | 2.96E-06 | 1.66E-05 | 3.953777 |
| HLA-DMB | 2.707011 | 11.41704 | 5.55183 | 1.04E-07 | 8.21E-07 | 7.172878 |
| RCC1 | 2.705294 | 10.40814 | 10.26821 | 1.26E-19 | 2.03E-17 | 34.00158 |
| RPS2 | 2.703714 | 15.00511 | 13.23864 | 4.19E-28 | 5.49E-25 | 53.18585 |
| LILRB2 | 2.703692 | 7.864638 | 4.846503 | 2.77E-06 | 1.56E-05 | 4.016041 |
| EDARADD | 2.701422 | 5.229014 | 6.166336 | 4.76E-09 | 5.18E-08 | 10.16285 |
| SYDE1 | 2.698241 | 10.77389 | 10.88773 | 2.26E-21 | 6.18E-19 | 37.94997 |
| CTHRC1 | 2.692575 | 8.518005 | 3.476038 | 0.000643 | 0.002037 | -1.13715 |
| PGF | 2.691423 | 9.764103 | 5.222343 | 5.01E-07 | 3.36E-06 | 5.659435 |
| RDH5 | 2.690709 | 6.956019 | 5.287223 | 3.70E-07 | 2.57E-06 | 5.95222 |
| LRRC17 | 2.688963 | 9.45873 | 4.358729 | 2.24E-05 | 0.000102 | 2.024271 |
| YBX1 | 2.688408 | 14.54647 | 12.28909 | 2.25E-25 | 1.48E-22 | 47.00746 |
| PSMB8 | 2.682706 | 11.67232 | 7.356249 | 7.21E-12 | 1.59E-10 | 16.48824 |
| SCIMP | 2.68064 | 8.071474 | 6.125599 | 5.88E-09 | 6.25E-08 | 9.958288 |
| LRRC25 | 2.680476 | 8.872768 | 5.709771 | 4.81E-08 | 4.07E-07 | 7.92121 |
| TSPAN12 | 2.680123 | 10.35136 | 4.983369 | 1.50E-06 | 9.02E-06 | 4.603921 |
| VEGFA | 2.67781 | 13.63452 | 3.811387 | 0.000192 | 0.0007 | -0.0066 |
| HLA-DPA1 | 2.675944 | 13.42919 | 5.417116 | 2.00E-07 | 1.49E-06 | 6.546159 |
| COL6A2 | 2.674867 | 12.71178 | 3.40915 | 0.00081 | 0.002496 | -1.35188 |
| COL5A1 | 2.673694 | 10.90962 | 3.461508 | 0.000676 | 0.00213 | -1.18411 |
| VCAM1 | 2.671324 | 11.01536 | 4.456748 | 1.49E-05 | 7.08E-05 | 2.411193 |
| TP73 | 2.669192 | 7.808402 | 3.601137 | 0.000413 | 0.001381 | -0.72585 |
| SLC18A3 | 2.668824 | 3.04606 | 2.284582 | 0.023548 | 0.049065 | -4.39455 |
| ELN | 2.667773 | 12.62058 | 5.581076 | 9.05E-08 | 7.22E-07 | 7.31035 |
| MYO1G | 2.665363 | 8.174114 | 5.028102 | 1.23E-06 | 7.50E-06 | 4.798721 |
| XPNPEP2 | 2.664825 | 5.389036 | 4.422818 | 1.71E-05 | 8.04E-05 | 2.27648 |
| NT5DC2 | 2.662451 | 12.06993 | 7.572148 | 2.08E-12 | 5.27E-11 | 17.70075 |
| TNFRSF9 | 2.661386 | 4.315252 | 4.367769 | 2.15E-05 | 9.86E-05 | 2.059666 |
| GBP3 | 2.658733 | 10.03923 | 3.607321 | 0.000404 | 0.001354 | -0.70519 |
| NPIPA3 | 2.657829 | 5.154601 | 5.18888 | 5.86E-07 | 3.88E-06 | 5.509453 |
| FLNA | 2.657808 | 14.85479 | 6.499657 | 8.24E-10 | 1.08E-08 | 11.86839 |
| S100A6 | 2.656507 | 14.12211 | 6.054855 | 8.46E-09 | 8.62E-08 | 9.60511 |
| STAC | 2.656279 | 7.929134 | 2.990938 | 0.003186 | 0.008498 | -2.61067 |
| HLA-A | 2.654592 | 15.29353 | 7.260285 | 1.24E-11 | 2.58E-10 | 15.95513 |
| C8orf88 | 2.653811 | 6.903622 | 6.022419 | 9.99E-09 | 1.00E-07 | 9.444066 |
| NAMPT | 2.651531 | 13.60683 | 4.432508 | 1.65E-05 | 7.76E-05 | 2.314869 |
| ZBTB42 | 2.647821 | 8.492767 | 6.582779 | 5.27E-10 | 7.21E-09 | 12.30223 |
| CDC25A | 2.643443 | 8.51607 | 5.682859 | 5.50E-08 | 4.60E-07 | 7.792684 |
| TPRG1 | 2.641739 | 5.937106 | 6.069728 | 7.84E-09 | 8.03E-08 | 9.679139 |
| SPATA12 | 2.641149 | 3.778696 | 5.932672 | 1.58E-08 | 1.50E-07 | 9.001419 |
| LIMA1 | 2.640414 | 12.43677 | 7.368074 | 6.74E-12 | 1.50E-10 | 16.55419 |
| PDIA4 | 2.640267 | 13.21864 | 9.164542 | 1.40E-16 | 1.05E-14 | 27.11586 |
| GNG5 | 2.639033 | 11.94696 | 10.45428 | 3.78E-20 | 7.02E-18 | 35.1824 |
| VAMP8 | 2.638204 | 10.44109 | 5.246406 | 4.48E-07 | 3.04E-06 | 5.767719 |
| OR2B6 | 2.637962 | 2.928201 | 4.490425 | 1.29E-05 | 6.25E-05 | 2.545706 |
| DYNLT1 | 2.635807 | 12.02701 | 9.726816 | 4.03E-18 | 4.56E-16 | 30.59592 |
| NMI | 2.633957 | 9.435918 | 7.304353 | 9.69E-12 | 2.06E-10 | 16.19949 |
| RNF122 | 2.633118 | 9.377504 | 6.526014 | 7.15E-10 | 9.48E-09 | 12.0056 |
| SLC28A1 | 2.632379 | 4.550078 | 4.097185 | 6.41E-05 | 0.000261 | 1.025908 |
| CD276 | 2.628685 | 12.41842 | 9.825343 | 2.15E-18 | 2.65E-16 | 31.21206 |
| ARL11 | 2.625258 | 7.198221 | 5.56943 | 9.58E-08 | 7.58E-07 | 7.255548 |
| MMP19 | 2.623429 | 8.288982 | 3.315462 | 0.001114 | 0.003323 | -1.64652 |
| S100A12 | 2.622199 | 3.875654 | 3.460472 | 0.000679 | 0.002138 | -1.18745 |
| COL28A1 | 2.620476 | 8.796767 | 3.060328 | 0.002562 | 0.006993 | -2.41197 |
| MUC1 | 2.619282 | 8.924533 | 5.865612 | 2.21E-08 | 2.03E-07 | 8.673534 |
| GPR1 | 2.619137 | 5.20056 | 3.065522 | 0.002521 | 0.006895 | -2.39693 |
| ADM2 | 2.618359 | 5.184798 | 3.693433 | 0.000296 | 0.001026 | -0.41439 |
| PTCRA | 2.616915 | 4.616487 | 4.07631 | 6.96E-05 | 0.000281 | 0.948402 |
| GZMA | 2.613886 | 5.53853 | 3.726217 | 0.000263 | 0.000923 | -0.30214 |
| DLEU1 | 2.612407 | 8.202721 | 7.557749 | 2.27E-12 | 5.66E-11 | 17.61933 |
| ADAMTS7 | 2.610622 | 8.274435 | 4.843066 | 2.82E-06 | 1.58E-05 | 4.001438 |
| ANTXR2 | 2.602902 | 10.03846 | 7.072341 | 3.59E-11 | 6.61E-10 | 14.92187 |
| C6orf118 | 2.600941 | 6.18467 | 4.105594 | 6.20E-05 | 0.000254 | 1.057224 |
| TNFSF14 | 2.60094 | 5.902522 | 3.419822 | 0.000781 | 0.002418 | -1.31787 |
| PMEL | 2.59959 | 5.264811 | 6.034061 | 9.41E-09 | 9.49E-08 | 9.501806 |
| GIMAP2 | 2.596765 | 9.045223 | 6.505793 | 7.97E-10 | 1.05E-08 | 11.90031 |
| HLA-B | 2.595651 | 15.32817 | 6.48163 | 9.07E-10 | 1.18E-08 | 11.77474 |
| TP53I3 | 2.594058 | 9.725275 | 8.887649 | 7.81E-16 | 5.00E-14 | 25.42758 |
| SIGLEC9 | 2.593792 | 8.540371 | 5.588222 | 8.74E-08 | 7.00E-07 | 7.34402 |
| ADGRE3 | 2.593462 | 3.176059 | 3.653191 | 0.000343 | 0.001166 | -0.55102 |
| WWTR1 | 2.591179 | 12.47168 | 6.104281 | 6.56E-09 | 6.90E-08 | 9.851583 |
| INMT | 2.590872 | 6.546159 | 3.467656 | 0.000662 | 0.002091 | -1.16426 |
| RAB13 | 2.588427 | 12.0765 | 9.692929 | 5.00E-18 | 5.53E-16 | 30.38441 |
| DBF4 | 2.586976 | 9.57204 | 7.719482 | 8.85E-13 | 2.47E-11 | 18.53819 |
| BST1 | 2.586942 | 7.706795 | 6.54137 | 6.59E-10 | 8.81E-09 | 12.08569 |
| SIGLEC1 | 2.585253 | 9.049697 | 3.720896 | 0.000268 | 0.000938 | -0.32042 |
| FZD7 | 2.584693 | 10.72531 | 4.976564 | 1.55E-06 | 9.26E-06 | 4.574401 |
| NSUN7 | 2.584586 | 7.14285 | 4.323964 | 2.58E-05 | 0.000116 | 1.88869 |
| RAB38 | 2.584135 | 5.546835 | 5.00117 | 1.39E-06 | 8.40E-06 | 4.681285 |
| KCNMB1 | 2.583695 | 8.662974 | 5.608074 | 7.93E-08 | 6.39E-07 | 7.437704 |
| GINS1 | 2.583563 | 9.888479 | 6.732625 | 2.34E-10 | 3.52E-09 | 13.09256 |
| FLVCR2 | 2.581301 | 8.840515 | 7.908649 | 2.92E-13 | 9.22E-12 | 19.62474 |
| BCL2A1 | 2.58086 | 7.687388 | 3.897612 | 0.000139 | 0.000522 | 0.298343 |
| FKBP7 | 2.580779 | 8.680359 | 7.423329 | 4.91E-12 | 1.13E-10 | 16.86305 |
| MMP11 | 2.578743 | 7.61701 | 4.460646 | 1.46E-05 | 6.98E-05 | 2.42672 |
| TNFAIP6 | 2.571069 | 8.945349 | 4.028471 | 8.38E-05 | 0.000333 | 0.772008 |
| RBBP8 | 2.568801 | 10.61786 | 10.04984 | 5.13E-19 | 7.25E-17 | 32.62222 |
| CNN3 | 2.568504 | 15.00671 | 9.81469 | 2.31E-18 | 2.78E-16 | 31.14536 |
| C1QB | 2.567062 | 13.90971 | 5.194745 | 5.70E-07 | 3.78E-06 | 5.535691 |
| PRDM13 | 2.566093 | 2.858398 | 2.327088 | 0.021115 | 0.044653 | -4.29978 |
| CD180 | 2.565204 | 8.107313 | 5.511693 | 1.27E-07 | 9.81E-07 | 6.985022 |
| IL7 | 2.564587 | 6.067076 | 5.023052 | 1.26E-06 | 7.67E-06 | 4.776663 |
| PABPC4L | 2.564254 | 5.705293 | 5.333677 | 2.97E-07 | 2.12E-06 | 6.163445 |
| COL5A2 | 2.563133 | 11.99707 | 4.906965 | 2.12E-06 | 1.23E-05 | 4.274224 |
| PLBD1 | 2.562879 | 8.647988 | 5.379405 | 2.39E-07 | 1.75E-06 | 6.372665 |
| STC1 | 2.561288 | 8.766088 | 3.52845 | 0.000535 | 0.001729 | -0.96636 |
| SERPINA1 | 2.556345 | 11.34371 | 4.355333 | 2.27E-05 | 0.000103 | 2.010988 |
| C1QTNF1 | 2.556025 | 11.18377 | 4.587061 | 8.58E-06 | 4.31E-05 | 2.936113 |
| RAB32 | 2.555544 | 9.955186 | 5.670905 | 5.83E-08 | 4.85E-07 | 7.735724 |
| PDIA5 | 2.553095 | 9.081644 | 6.512569 | 7.69E-10 | 1.01E-08 | 11.93557 |
| NID2 | 2.552464 | 9.145025 | 5.044486 | 1.14E-06 | 7.02E-06 | 4.870389 |
| TP53 | 2.551812 | 11.69376 | 7.852743 | 4.05E-13 | 1.23E-11 | 19.30233 |
| CISH | 2.551394 | 7.976118 | 5.284826 | 3.74E-07 | 2.59E-06 | 5.941356 |
| MFAP4 | 2.551373 | 10.60371 | 4.154395 | 5.11E-05 | 0.000214 | 1.239993 |
| ZC3H11B | 2.548465 | 3.753561 | 6.310662 | 2.24E-09 | 2.65E-08 | 10.89449 |
| IL24 | 2.547204 | 4.572926 | 4.416605 | 1.76E-05 | 8.22E-05 | 2.251904 |
| CHEK1 | 2.543576 | 9.587094 | 8.487254 | 9.10E-15 | 4.32E-13 | 23.02066 |
| BARX1 | 2.543312 | 3.928408 | 2.640867 | 0.009024 | 0.021227 | -3.54984 |
| P2RY8 | 2.542614 | 6.494457 | 4.746331 | 4.31E-06 | 2.31E-05 | 3.593657 |
| FAM72D | 2.541967 | 4.640954 | 6.980142 | 6.00E-11 | 1.04E-09 | 14.4204 |
| FBXO5 | 2.540712 | 9.318017 | 7.535037 | 2.58E-12 | 6.37E-11 | 17.49107 |
| AGBL2 | 2.540231 | 6.033033 | 4.877216 | 2.42E-06 | 1.38E-05 | 4.146885 |
| TICRR | 2.540105 | 7.822693 | 5.340881 | 2.87E-07 | 2.06E-06 | 6.196324 |
| CASP6 | 2.536704 | 8.931995 | 9.493592 | 1.77E-17 | 1.67E-15 | 29.14464 |
| DKKL1 | 2.535993 | 4.183751 | 3.980605 | 0.000101 | 0.000393 | 0.597242 |
| ALOX5AP | 2.534398 | 11.34657 | 4.649112 | 6.58E-06 | 3.37E-05 | 3.190221 |
| C19orf35 | 2.533143 | 6.360384 | 6.039903 | 9.14E-09 | 9.24E-08 | 9.530806 |
| CHST14 | 2.530901 | 10.59681 | 10.11886 | 3.29E-19 | 4.83E-17 | 33.05743 |
| CD74 | 2.527093 | 16.09931 | 5.729184 | 4.37E-08 | 3.74E-07 | 8.014181 |
| SLC7A7 | 2.525893 | 10.22264 | 6.3026 | 2.34E-09 | 2.75E-08 | 10.85334 |
| LAG3 | 2.520731 | 5.95514 | 5.72066 | 4.56E-08 | 3.88E-07 | 7.973334 |
| CKLF | 2.520152 | 8.727609 | 7.999782 | 1.70E-13 | 5.79E-12 | 20.15255 |
| WISP1 | 2.520092 | 8.164278 | 2.948967 | 0.003628 | 0.009532 | -2.72886 |
| CD2 | 2.51989 | 6.08775 | 3.682926 | 0.000308 | 0.001061 | -0.45019 |
| PPP1R3B | 2.514372 | 9.802276 | 6.468455 | 9.73E-10 | 1.25E-08 | 11.7064 |
| PLVAP | 2.513126 | 10.54886 | 4.844147 | 2.80E-06 | 1.58E-05 | 4.00603 |
| PCNA | 2.512657 | 12.38345 | 9.206849 | 1.07E-16 | 8.47E-15 | 27.3754 |
| NXT1 | 2.512353 | 10.10876 | 8.137467 | 7.49E-14 | 2.80E-12 | 20.95517 |
| CD68 | 2.510796 | 6.637976 | 5.613046 | 7.74E-08 | 6.25E-07 | 7.461207 |
| UQCRHL | 2.509366 | 8.755105 | 8.868958 | 8.77E-16 | 5.51E-14 | 25.31429 |
| CASP5 | 2.50875 | 4.390689 | 4.827666 | 3.02E-06 | 1.68E-05 | 3.936101 |
| CNPY1 | 2.508309 | 5.862277 | 2.5725 | 0.010934 | 0.025095 | -3.72073 |
| SPINK1 | 2.504029 | 3.308664 | 2.949947 | 0.003617 | 0.009506 | -2.72612 |
| MSTN | 2.503323 | 7.914189 | 2.704947 | 0.007513 | 0.018122 | -3.38592 |
| KYNU | 2.50318 | 7.463187 | 5.274388 | 3.93E-07 | 2.71E-06 | 5.894093 |
| ADAMTS15 | 2.502487 | 8.68419 | 4.095863 | 6.44E-05 | 0.000262 | 1.020993 |
| ECM2 | 2.502225 | 10.04032 | 5.26132 | 4.18E-07 | 2.86E-06 | 5.835014 |
| FAM111A | 2.499765 | 10.64794 | 8.501389 | 8.35E-15 | 4.08E-13 | 23.10489 |
| CP | 2.499174 | 10.53777 | 3.40167 | 0.000831 | 0.002554 | -1.37567 |
| LILRA5 | 2.49912 | 5.451754 | 3.534252 | 0.000524 | 0.001698 | -0.94732 |
| HOXC5 | 2.498856 | 2.42705 | 3.52217 | 0.000547 | 0.001763 | -0.98694 |
| CD300LF | 2.4981 | 7.211457 | 4.948578 | 1.76E-06 | 1.04E-05 | 4.453317 |
| NTN1 | 2.497918 | 11.82268 | 5.907347 | 1.79E-08 | 1.68E-07 | 8.877302 |
| PHEX | 2.495834 | 8.240756 | 6.478495 | 9.22E-10 | 1.19E-08 | 11.75847 |
| RP2 | 2.495644 | 10.29933 | 10.87107 | 2.52E-21 | 6.71E-19 | 37.84321 |
| IDH1 | 2.493668 | 12.44757 | 9.37789 | 3.67E-17 | 3.23E-15 | 28.42865 |
| PROCR | 2.493408 | 8.862756 | 5.898649 | 1.87E-08 | 1.75E-07 | 8.834755 |
| CAV1 | 2.493366 | 11.64469 | 4.09336 | 6.51E-05 | 0.000265 | 1.011683 |
| SH2D4A | 2.492456 | 8.10634 | 4.98226 | 1.51E-06 | 9.06E-06 | 4.59911 |
| CDKN3 | 2.490866 | 8.695583 | 5.481703 | 1.47E-07 | 1.12E-06 | 6.845283 |
| PKMYT1 | 2.487322 | 8.865298 | 5.381527 | 2.37E-07 | 1.73E-06 | 6.382405 |
| ANKUB1 | 2.486206 | 4.217902 | 3.311227 | 0.00113 | 0.003364 | -1.65966 |
| EEF1A1 | 2.483284 | 17.85627 | 8.215387 | 4.70E-14 | 1.85E-12 | 21.41207 |
| TBX2 | 2.482593 | 10.12593 | 5.950306 | 1.44E-08 | 1.39E-07 | 9.088049 |
| CETP | 2.480941 | 6.781047 | 5.335841 | 2.94E-07 | 2.10E-06 | 6.173321 |
| GPBAR1 | 2.478496 | 4.739252 | 6.003098 | 1.10E-08 | 1.09E-07 | 9.348404 |
| DNAH11 | 2.47802 | 6.59869 | 3.886398 | 0.000145 | 0.000543 | 0.258363 |
| COL4A6 | 2.477326 | 7.442629 | 3.508534 | 0.000574 | 0.001838 | -1.03152 |
| PRIM2 | 2.475759 | 8.967747 | 9.696018 | 4.91E-18 | 5.45E-16 | 30.40368 |
| MMP1 | 2.475615 | 4.898281 | 2.497239 | 0.013448 | 0.03011 | -3.90405 |
| HIST2H4B | 2.473821 | 2.402734 | 5.106458 | 8.57E-07 | 5.44E-06 | 5.143042 |
| TSHR | 2.473293 | 5.344963 | 2.592418 | 0.010343 | 0.023909 | -3.67137 |
| GSX2 | 2.472084 | 6.045458 | 2.613384 | 0.009752 | 0.022707 | -3.61904 |
| HIST1H4J | 2.470978 | 3.186025 | 4.215222 | 4.01E-05 | 0.000172 | 1.470271 |
| SMC4 | 2.470818 | 10.32609 | 6.943456 | 7.35E-11 | 1.26E-09 | 14.22189 |
| SLC40A1 | 2.470385 | 11.48149 | 5.41703 | 2.00E-07 | 1.49E-06 | 6.545759 |
| KCNE3 | 2.469451 | 8.724462 | 6.039701 | 9.15E-09 | 9.24E-08 | 9.529798 |
| LFNG | 2.469106 | 11.92726 | 5.158436 | 6.75E-07 | 4.40E-06 | 5.373612 |
| MSN | 2.468741 | 14.41585 | 7.646855 | 1.35E-12 | 3.60E-11 | 18.12439 |
| LGALS3 | 2.465402 | 12.58819 | 3.883237 | 0.000146 | 0.000548 | 0.247109 |
| HIST2H2AC | 2.463504 | 4.703372 | 4.419068 | 1.74E-05 | 8.14E-05 | 2.261645 |
| TMEM154 | 2.462789 | 8.064057 | 5.488195 | 1.42E-07 | 1.09E-06 | 6.875485 |
| SLFN12 | 2.462412 | 7.183267 | 5.593623 | 8.52E-08 | 6.83E-07 | 7.369486 |
| BMF | 2.458778 | 8.977064 | 6.468143 | 9.74E-10 | 1.25E-08 | 11.70478 |
| KDELC1 | 2.458646 | 8.568947 | 7.23154 | 1.46E-11 | 2.99E-10 | 15.79616 |
| HLA-DMA | 2.455419 | 11.5588 | 5.582611 | 8.99E-08 | 7.17E-07 | 7.317581 |
| IL10 | 2.454508 | 5.55445 | 3.720143 | 0.000269 | 0.000941 | -0.323 |
| HIST1H2BO | 2.454018 | 3.363958 | 3.937009 | 0.000119 | 0.000457 | 0.439576 |
| C1QA | 2.451567 | 13.1652 | 4.90534 | 2.13E-06 | 1.24E-05 | 4.267249 |
| ZNF474 | 2.447888 | 5.081961 | 3.776252 | 0.000218 | 0.000785 | -0.1292 |
| EFNA4 | 2.447547 | 7.362377 | 6.358513 | 1.74E-09 | 2.11E-08 | 11.1394 |
| F8A3 | 2.447455 | 3.395355 | 6.73372 | 2.33E-10 | 3.51E-09 | 13.09837 |
| C5AR1 | 2.444103 | 10.05751 | 4.417688 | 1.75E-05 | 8.19E-05 | 2.256186 |
| FNDC11 | 2.443695 | 4.487758 | 3.799121 | 0.000201 | 0.000728 | -0.04951 |
| C1QC | 2.442265 | 13.66965 | 5.202107 | 5.51E-07 | 3.66E-06 | 5.568657 |
| HLA-DPB1 | 2.440927 | 12.8953 | 5.310673 | 3.31E-07 | 2.33E-06 | 6.058683 |
| XRCC2 | 2.440857 | 7.821251 | 5.520709 | 1.21E-07 | 9.44E-07 | 7.027136 |
| CLEC4F | 2.440337 | 3.730509 | 3.288282 | 0.00122 | 0.003606 | -1.73064 |
| CD80 | 2.439834 | 3.943161 | 4.82792 | 3.01E-06 | 1.68E-05 | 3.937177 |
| NOD2 | 2.439209 | 7.038068 | 5.141396 | 7.30E-07 | 4.72E-06 | 5.297836 |
| FAM72B | 2.439148 | 5.749451 | 6.673896 | 3.22E-10 | 4.67E-09 | 12.78156 |
| MS4A7 | 2.438333 | 10.60504 | 4.849082 | 2.74E-06 | 1.55E-05 | 4.027005 |
| BARD1 | 2.437993 | 9.632812 | 8.973998 | 4.58E-16 | 3.08E-14 | 25.9521 |
| TNNI2 | 2.433282 | 4.796631 | 5.324659 | 3.10E-07 | 2.20E-06 | 6.122339 |
| RHOH | 2.43186 | 6.772006 | 5.068281 | 1.02E-06 | 6.37E-06 | 4.974786 |
| FCGR3A | 2.431706 | 12.68259 | 4.641721 | 6.79E-06 | 3.47E-05 | 3.159814 |
| RPL39 | 2.431298 | 12.18447 | 6.867414 | 1.12E-10 | 1.82E-09 | 13.81228 |
| GINS4 | 2.430501 | 8.013421 | 6.754106 | 2.08E-10 | 3.18E-09 | 13.20671 |
| APOBEC3D | 2.429822 | 6.164122 | 5.421383 | 1.96E-07 | 1.46E-06 | 6.565843 |
| EMILIN2 | 2.429408 | 9.773467 | 4.749464 | 4.25E-06 | 2.28E-05 | 3.606767 |
| HELLS | 2.426944 | 8.482447 | 5.561749 | 9.95E-08 | 7.85E-07 | 7.219446 |
| DOK2 | 2.426431 | 6.722061 | 4.218683 | 3.95E-05 | 0.00017 | 1.483452 |
| EVA1B | 2.425807 | 9.005782 | 6.284136 | 2.58E-09 | 3.00E-08 | 10.75923 |
| SGO2 | 2.425787 | 8.927055 | 7.323704 | 8.68E-12 | 1.87E-10 | 16.30703 |
| SPRY1 | 2.42535 | 10.67333 | 4.8881 | 2.31E-06 | 1.33E-05 | 4.193406 |
| CRIP1 | 2.424594 | 5.094642 | 5.921478 | 1.67E-08 | 1.58E-07 | 8.946515 |
| EVC2 | 2.422078 | 7.31947 | 4.30818 | 2.75E-05 | 0.000123 | 1.827425 |
| HAUS1 | 2.421232 | 10.00993 | 7.624735 | 1.54E-12 | 4.01E-11 | 17.99874 |
| LILRA6 | 2.421038 | 5.792941 | 4.452704 | 1.51E-05 | 7.19E-05 | 2.395094 |
| TIMELESS | 2.419293 | 10.673 | 7.188898 | 1.86E-11 | 3.71E-10 | 15.56095 |
| CLEC2B | 2.418702 | 8.276999 | 4.148124 | 5.24E-05 | 0.000218 | 1.216407 |
| ZWINT | 2.416395 | 9.563021 | 6.048233 | 8.75E-09 | 8.90E-08 | 9.572188 |
| LOXL1 | 2.416264 | 8.973332 | 3.642551 | 0.000356 | 0.001208 | -0.58693 |
| PTPRZ1 | 2.416261 | 15.8251 | 4.363144 | 2.20E-05 | 0.0001 | 2.041552 |
| GAS1 | 2.414317 | 10.29301 | 4.048546 | 7.75E-05 | 0.00031 | 0.845819 |
| SLC37A2 | 2.413801 | 9.308782 | 5.71614 | 4.66E-08 | 3.96E-07 | 7.951689 |
| XIRP2 | 2.412485 | 2.843064 | 3.786275 | 0.00021 | 0.000759 | -0.09432 |
| RNASEH2A | 2.411024 | 10.68113 | 7.267648 | 1.19E-11 | 2.48E-10 | 15.9959 |
| PDLIM1 | 2.408607 | 10.25387 | 5.179219 | 6.13E-07 | 4.03E-06 | 5.466284 |
| AKNAD1 | 2.407997 | 6.435699 | 4.339045 | 2.42E-05 | 0.00011 | 1.947399 |
| F2RL1 | 2.407065 | 8.084585 | 3.73105 | 0.000258 | 0.000909 | -0.28552 |
| OSMR | 2.404492 | 11.32148 | 4.369479 | 2.14E-05 | 9.80E-05 | 2.06637 |
| MCM2 | 2.403852 | 11.52513 | 6.220185 | 3.60E-09 | 4.05E-08 | 10.43459 |
| TREM2 | 2.403666 | 11.63136 | 4.964118 | 1.64E-06 | 9.74E-06 | 4.520489 |
| IL18RAP | 2.403539 | 3.940669 | 3.888314 | 0.000144 | 0.00054 | 0.265187 |
| CLECL1 | 2.401599 | 4.73208 | 4.063281 | 7.32E-05 | 0.000295 | 0.900189 |
| CST7 | 2.401027 | 5.810927 | 4.677993 | 5.81E-06 | 3.01E-05 | 3.309398 |
| CKS1B | 2.400864 | 9.622043 | 6.841587 | 1.29E-10 | 2.06E-09 | 13.67374 |
| MMP8 | 2.399489 | 2.330538 | 2.645469 | 0.008907 | 0.020982 | -3.53819 |
| C1orf94 | 2.39901 | 7.075215 | 3.958349 | 0.00011 | 0.000424 | 0.516571 |
| PTK7 | 2.396802 | 11.2291 | 7.846981 | 4.19E-13 | 1.26E-11 | 19.26916 |
| SLC1A5 | 2.396073 | 10.13342 | 6.255793 | 2.99E-09 | 3.42E-08 | 10.61509 |
| RBP1 | 2.396023 | 12.00607 | 3.917728 | 0.000128 | 0.000488 | 0.370311 |
| SOCS2 | 2.395865 | 10.5548 | 3.451465 | 0.0007 | 0.002198 | -1.21646 |
| NPC2 | 2.39585 | 12.97332 | 6.158705 | 4.96E-09 | 5.37E-08 | 10.12447 |
| CTSC | 2.395651 | 11.67981 | 5.261653 | 4.17E-07 | 2.85E-06 | 5.836518 |
| ZNF300 | 2.395515 | 9.529364 | 5.342794 | 2.85E-07 | 2.04E-06 | 6.205059 |
| NEXN | 2.390516 | 7.868539 | 5.338556 | 2.90E-07 | 2.08E-06 | 6.185708 |
| CDT1 | 2.389983 | 9.287164 | 5.410258 | 2.06E-07 | 1.53E-06 | 6.514542 |
| ABCB4 | 2.389071 | 7.311972 | 6.602445 | 4.74E-10 | 6.57E-09 | 12.40536 |
| SNX20 | 2.38904 | 7.704254 | 4.770935 | 3.87E-06 | 2.09E-05 | 3.696776 |
| MXD3 | 2.385844 | 9.07757 | 5.260183 | 4.20E-07 | 2.87E-06 | 5.82988 |
| HAPLN3 | 2.385672 | 8.301209 | 6.30929 | 2.26E-09 | 2.67E-08 | 10.88749 |
| SIGLEC12 | 2.384996 | 3.403516 | 2.71227 | 0.007355 | 0.017782 | -3.36695 |
| SAPCD1 | 2.383769 | 5.370403 | 5.009379 | 1.34E-06 | 8.11E-06 | 4.71703 |
| IL2RG | 2.382384 | 7.427931 | 5.117324 | 8.16E-07 | 5.20E-06 | 5.191104 |
| DIAPH3 | 2.381676 | 7.926054 | 6.320691 | 2.13E-09 | 2.52E-08 | 10.94573 |
| CCL5 | 2.378777 | 7.778786 | 4.21859 | 3.95E-05 | 0.00017 | 1.4831 |
| AIM2 | 2.378564 | 5.327115 | 3.757457 | 0.000234 | 0.000834 | -0.19439 |
| JAG1 | 2.378129 | 12.0898 | 5.814641 | 2.86E-08 | 2.56E-07 | 8.42597 |
| ACP5 | 2.377462 | 7.824451 | 3.863076 | 0.000158 | 0.000587 | 0.175518 |
| BATF | 2.376789 | 5.75301 | 4.307575 | 2.76E-05 | 0.000123 | 1.82508 |
| RPSA | 2.373963 | 15.00984 | 8.677934 | 2.84E-15 | 1.56E-13 | 24.16156 |
| PINLYP | 2.373106 | 8.90806 | 5.256442 | 4.27E-07 | 2.91E-06 | 5.812989 |
| CD36 | 2.371612 | 7.875841 | 3.401213 | 0.000833 | 0.002557 | -1.37712 |
| LILRB3 | 2.370548 | 6.385665 | 4.782724 | 3.68E-06 | 2.00E-05 | 3.746331 |
| HIST1H1B | 2.369 | 2.808646 | 3.250277 | 0.001385 | 0.004043 | -1.84725 |
| GAL3ST4 | 2.368899 | 11.33744 | 7.990446 | 1.80E-13 | 6.06E-12 | 20.09835 |
| PRF1 | 2.368453 | 7.352694 | 3.492984 | 0.000606 | 0.001932 | -1.08217 |
| FBN2 | 2.367986 | 7.752091 | 2.413528 | 0.016839 | 0.036607 | -4.10201 |
| ANPEP | 2.366769 | 8.646733 | 3.783857 | 0.000212 | 0.000766 | -0.10275 |
| HMOX1 | 2.366391 | 12.34321 | 4.413831 | 1.78E-05 | 8.31E-05 | 2.240937 |
| RHOC | 2.366166 | 13.57173 | 9.760539 | 3.26E-18 | 3.77E-16 | 30.80661 |
| TEKT1 | 2.365983 | 6.483099 | 2.499095 | 0.01338 | 0.029972 | -3.8996 |
| PDE6G | 2.365096 | 5.04725 | 5.909405 | 1.77E-08 | 1.67E-07 | 8.887377 |
| WDR63 | 2.364687 | 5.131433 | 3.161603 | 0.001852 | 0.005232 | -2.11464 |
| STK17B | 2.363735 | 10.33166 | 7.633121 | 1.46E-12 | 3.85E-11 | 18.04636 |
| ASB9 | 2.363574 | 5.744589 | 4.941708 | 1.81E-06 | 1.07E-05 | 4.42367 |
| NKG7 | 2.363243 | 6.403371 | 4.717723 | 4.89E-06 | 2.58E-05 | 3.474274 |
| IGFBP4 | 2.36203 | 12.19955 | 5.523422 | 1.20E-07 | 9.33E-07 | 7.039817 |
| HLA-DRB1 | 2.359986 | 13.46518 | 4.719962 | 4.84E-06 | 2.56E-05 | 3.483599 |
| RAB20 | 2.35965 | 9.597236 | 6.304247 | 2.32E-09 | 2.73E-08 | 10.86175 |
| NCF4 | 2.359446 | 8.909912 | 5.350777 | 2.74E-07 | 1.97E-06 | 6.241537 |
| GNG12 | 2.35853 | 12.44581 | 8.87489 | 8.45E-16 | 5.36E-14 | 25.35023 |
| LGALS1 | 2.358074 | 13.89074 | 5.886383 | 1.99E-08 | 1.85E-07 | 8.774826 |
| CAPG | 2.357831 | 12.28347 | 5.955566 | 1.40E-08 | 1.36E-07 | 9.11392 |
| FKBP1C | 2.353639 | 6.774854 | 8.089453 | 9.98E-14 | 3.63E-12 | 20.67458 |
| BTN2A2 | 2.352479 | 9.550344 | 11.68279 | 1.23E-23 | 5.05E-21 | 43.07371 |
| MTTP | 2.351107 | 7.958779 | 3.154404 | 0.001896 | 0.005343 | -2.13606 |
| CTCFL | 2.349895 | 2.657369 | 5.042706 | 1.15E-06 | 7.07E-06 | 4.862594 |
| CD151 | 2.349319 | 13.14748 | 6.604324 | 4.70E-10 | 6.52E-09 | 12.41522 |
| C8orf4 | 2.348466 | 10.52538 | 4.0974 | 6.40E-05 | 0.000261 | 1.026708 |
| LRRC36 | 2.346288 | 6.350384 | 4.340564 | 2.41E-05 | 0.000109 | 1.953321 |
| FCGRT | 2.343038 | 12.84631 | 8.691427 | 2.62E-15 | 1.47E-13 | 24.24267 |
| GPX3 | 2.342796 | 12.07729 | 3.685775 | 0.000305 | 0.001051 | -0.44049 |
| APOC2 | 2.337587 | 6.78821 | 4.507779 | 1.20E-05 | 5.85E-05 | 2.615334 |
| NCF1 | 2.33619 | 7.614976 | 5.316068 | 3.23E-07 | 2.28E-06 | 6.08322 |
| GJC1 | 2.335378 | 10.20004 | 8.452601 | 1.12E-14 | 5.17E-13 | 22.81442 |
| CDC6 | 2.335144 | 9.034735 | 5.711525 | 4.77E-08 | 4.04E-07 | 7.9296 |
| SYTL4 | 2.33391 | 10.53331 | 5.830947 | 2.63E-08 | 2.38E-07 | 8.505013 |
| TES | 2.333506 | 8.514416 | 6.668163 | 3.32E-10 | 4.80E-09 | 12.75129 |
| MYD88 | 2.330616 | 11.01727 | 9.399642 | 3.20E-17 | 2.87E-15 | 28.56305 |
| RNASE6 | 2.329906 | 9.708284 | 5.012353 | 1.32E-06 | 8.02E-06 | 4.729991 |
| FCER1A | 2.329498 | 4.516151 | 2.69664 | 0.007695 | 0.018512 | -3.40737 |
| CABP4 | 2.327589 | 6.739742 | 5.429334 | 1.88E-07 | 1.41E-06 | 6.602549 |
| RUNX1 | 2.326834 | 10.51172 | 4.780873 | 3.71E-06 | 2.02E-05 | 3.738545 |
| TGIF2 | 2.326569 | 9.769347 | 6.917732 | 8.48E-11 | 1.43E-09 | 14.08303 |
| TYROBP | 2.325906 | 11.91798 | 4.942919 | 1.80E-06 | 1.06E-05 | 4.428896 |
| SDC1 | 2.324518 | 9.519275 | 3.807355 | 0.000195 | 0.000709 | -0.02071 |
| PABPC1L | 2.322881 | 9.884806 | 4.459016 | 1.47E-05 | 7.02E-05 | 2.420227 |
| RPL18A | 2.321705 | 13.86475 | 7.528276 | 2.69E-12 | 6.57E-11 | 17.45293 |
| CALD1 | 2.321705 | 13.37938 | 7.885294 | 3.35E-13 | 1.04E-11 | 19.48992 |
| PABPC3 | 2.321304 | 6.673399 | 7.852083 | 4.07E-13 | 1.23E-11 | 19.29853 |
| TRIM21 | 2.320446 | 9.697025 | 7.471152 | 3.73E-12 | 8.86E-11 | 17.13133 |
| APOBEC3G | 2.319966 | 9.009021 | 4.93514 | 1.87E-06 | 1.10E-05 | 4.395355 |
| TRIM6 | 2.319326 | 6.839196 | 4.831709 | 2.96E-06 | 1.66E-05 | 3.953237 |
| STEAP1 | 2.316922 | 7.202429 | 3.161045 | 0.001855 | 0.005241 | -2.1163 |
| HLA-DQB1 | 2.31681 | 11.42703 | 3.92467 | 0.000125 | 0.000476 | 0.395215 |
| DPYD | 2.316447 | 10.77005 | 4.418595 | 1.75E-05 | 8.16E-05 | 2.259774 |
| CHST9 | 2.315405 | 8.925791 | 2.849886 | 0.004903 | 0.012376 | -3.00188 |
| ALX3 | 2.315168 | 3.934811 | 3.067224 | 0.002507 | 0.006864 | -2.39199 |
| HAUS8 | 2.315118 | 8.516022 | 7.766148 | 6.74E-13 | 1.92E-11 | 18.80507 |
| ADIG | 2.31491 | 3.109829 | 3.449387 | 0.000705 | 0.002211 | -1.22315 |
| S100A16 | 2.312135 | 13.06027 | 5.223723 | 4.98E-07 | 3.34E-06 | 5.665635 |
| APOBEC3F | 2.309537 | 7.370264 | 5.40954 | 2.07E-07 | 1.53E-06 | 6.511234 |
| SP140L | 2.308466 | 8.658189 | 5.444671 | 1.75E-07 | 1.32E-06 | 6.673471 |
| FAM83A | 2.307029 | 2.606778 | 5.127547 | 7.78E-07 | 4.99E-06 | 5.236385 |
| SERPINA3 | 2.306986 | 8.822028 | 3.737733 | 0.000252 | 0.000891 | -0.26251 |
| HLA-C | 2.306846 | 14.95324 | 5.693892 | 5.21E-08 | 4.38E-07 | 7.845324 |
| PCDHB9 | 2.306304 | 8.967452 | 5.359712 | 2.63E-07 | 1.90E-06 | 6.282408 |
| DBI | 2.306264 | 14.30851 | 6.086288 | 7.20E-09 | 7.49E-08 | 9.76171 |
| CD63 | 2.304644 | 15.22447 | 7.719566 | 8.85E-13 | 2.47E-11 | 18.53867 |
| EPHB4 | 2.299228 | 10.46701 | 5.0864 | 9.40E-07 | 5.91E-06 | 5.054526 |
| LOXL3 | 2.299137 | 10.48172 | 9.540211 | 1.32E-17 | 1.30E-15 | 29.43389 |
| ETV1 | 2.298748 | 12.81173 | 5.395762 | 2.21E-07 | 1.62E-06 | 6.447813 |
| LGALS9C | 2.298244 | 3.021296 | 4.75557 | 4.14E-06 | 2.22E-05 | 3.632334 |
| HIST1H2AI | 2.297845 | 2.628849 | 3.626886 | 0.000377 | 0.001272 | -0.63964 |
| RNASE3 | 2.296593 | 5.260382 | 3.353926 | 0.000978 | 0.002961 | -1.52643 |
| KDELC2 | 2.296476 | 10.7893 | 6.930734 | 7.89E-11 | 1.34E-09 | 14.15318 |
| CCRL2 | 2.295923 | 7.40098 | 5.716877 | 4.65E-08 | 3.95E-07 | 7.955214 |
| MYCBP | 2.294735 | 9.117268 | 7.99225 | 1.78E-13 | 6.01E-12 | 20.10883 |
| CD72 | 2.293026 | 7.20375 | 4.829037 | 3.00E-06 | 1.67E-05 | 3.941908 |
| DRAM1 | 2.291751 | 9.725607 | 6.212986 | 3.74E-09 | 4.20E-08 | 10.39817 |
| CTSS | 2.288132 | 11.5448 | 4.61244 | 7.70E-06 | 3.90E-05 | 3.039721 |
| GSDMA | 2.287813 | 5.313707 | 2.510602 | 0.012967 | 0.029164 | -3.87188 |
| NEDD1 | 2.28777 | 9.878694 | 10.7564 | 5.33E-21 | 1.29E-18 | 37.10914 |
| MND1 | 2.287433 | 7.039053 | 4.844783 | 2.80E-06 | 1.57E-05 | 4.008733 |
| SIX3 | 2.287248 | 3.07527 | 2.640917 | 0.009023 | 0.021226 | -3.54972 |
| CYTIP | 2.285169 | 7.757757 | 4.816017 | 3.18E-06 | 1.76E-05 | 3.886781 |
| CCL20 | 2.285054 | 5.294955 | 2.40236 | 0.017345 | 0.037556 | -4.12794 |
| DHRSX | 2.283619 | 10.47799 | 8.596192 | 4.68E-15 | 2.44E-13 | 23.67124 |
| HMGB2 | 2.28264 | 12.13835 | 7.364381 | 6.88E-12 | 1.53E-10 | 16.53359 |
| DTYMK | 2.282587 | 11.01075 | 7.957505 | 2.18E-13 | 7.23E-12 | 19.90735 |
| SLC2A10 | 2.282267 | 10.25867 | 5.457866 | 1.64E-07 | 1.24E-06 | 6.734592 |
| MCUB | 2.281649 | 10.60765 | 5.501841 | 1.33E-07 | 1.02E-06 | 6.939059 |
| EDN2 | 2.279606 | 2.365065 | 2.998372 | 0.003113 | 0.008329 | -2.58958 |
| ZIC4 | 2.277836 | 7.856042 | 4.992407 | 1.44E-06 | 8.69E-06 | 4.643176 |
| MTRNR2L11 | 2.276837 | 2.441788 | 5.48438 | 1.45E-07 | 1.11E-06 | 6.857735 |
| FBP1 | 2.276703 | 8.474158 | 4.834549 | 2.93E-06 | 1.64E-05 | 3.965282 |
| CILP | 2.275802 | 5.646029 | 3.626095 | 0.000378 | 0.001275 | -0.64229 |
| PLAC1 | 2.274149 | 2.919226 | 3.808993 | 0.000193 | 0.000705 | -0.01498 |
| SUSD2 | 2.272742 | 8.683741 | 4.505634 | 1.21E-05 | 5.90E-05 | 2.606719 |
| CCR2 | 2.271748 | 5.323543 | 3.018028 | 0.002928 | 0.00789 | -2.53358 |
| SAMD9 | 2.271476 | 9.812547 | 4.884702 | 2.34E-06 | 1.34E-05 | 4.178872 |
| KDELR3 | 2.271218 | 8.195405 | 3.596918 | 0.00042 | 0.0014 | -0.73992 |
| TRIB3 | 2.271114 | 9.874175 | 4.648124 | 6.61E-06 | 3.38E-05 | 3.186154 |
| HMCN1 | 2.269509 | 7.398461 | 4.137031 | 5.47E-05 | 0.000227 | 1.174758 |
| CALU | 2.268656 | 13.48517 | 7.248572 | 1.33E-11 | 2.74E-10 | 15.89031 |
| EPO | 2.267376 | 3.455099 | 3.66071 | 0.000334 | 0.00114 | -0.52559 |
| RPS5 | 2.266798 | 14.58432 | 6.932874 | 7.80E-11 | 1.33E-09 | 14.16473 |
| C17orf53 | 2.266185 | 7.51613 | 6.731787 | 2.35E-10 | 3.53E-09 | 13.08812 |
| MYB | 2.265686 | 5.414909 | 3.931952 | 0.000122 | 0.000465 | 0.421384 |
| BMP1 | 2.265583 | 11.14372 | 7.104539 | 2.99E-11 | 5.63E-10 | 15.09785 |
| PLTP | 2.263754 | 13.91266 | 5.150448 | 7.00E-07 | 4.54E-06 | 5.33807 |
| TAS1R1 | 2.263483 | 5.392761 | 4.505102 | 1.22E-05 | 5.91E-05 | 2.604578 |
| CENPW | 2.261352 | 7.513455 | 5.248247 | 4.44E-07 | 3.02E-06 | 5.77602 |
| FAM26F | 2.261043 | 7.86218 | 3.876395 | 0.00015 | 0.000561 | 0.222778 |
| FAM72C | 2.260969 | 3.99032 | 4.51979 | 1.14E-05 | 5.60E-05 | 2.66365 |
| HSPG2 | 2.259809 | 11.52109 | 4.26669 | 3.26E-05 | 0.000143 | 1.667238 |
| CD300A | 2.255535 | 9.943877 | 5.046258 | 1.13E-06 | 6.97E-06 | 4.878154 |
| CLEC18A | 2.255026 | 5.04538 | 3.41097 | 0.000805 | 0.002483 | -1.34609 |
| RETN | 2.254936 | 2.946637 | 2.69691 | 0.007689 | 0.0185 | -3.40668 |
| RPS19 | 2.254213 | 14.71263 | 8.55844 | 5.90E-15 | 2.99E-13 | 23.44541 |
| NPIPB5 | 2.253252 | 8.187495 | 5.750861 | 3.93E-08 | 3.40E-07 | 8.118247 |
| PXDN | 2.250884 | 11.50177 | 4.419166 | 1.74E-05 | 8.14E-05 | 2.262031 |
| DNAAF3 | 2.247794 | 5.741043 | 3.359168 | 0.000961 | 0.002913 | -1.50996 |
| RPEL1 | 2.247268 | 2.966649 | 6.287668 | 2.53E-09 | 2.95E-08 | 10.77722 |
| RIBC1 | 2.246921 | 7.244474 | 3.932165 | 0.000122 | 0.000464 | 0.422147 |
| GZMH | 2.246754 | 4.63925 | 3.554433 | 0.000488 | 0.001593 | -0.88087 |
| RACK1 | 2.246563 | 15.15451 | 12.50552 | 5.38E-26 | 4.23E-23 | 48.41477 |
| PPIAL4G | 2.246224 | 2.452134 | 6.052048 | 8.58E-09 | 8.73E-08 | 9.59115 |
| C1orf162 | 2.246207 | 9.750021 | 5.341967 | 2.86E-07 | 2.05E-06 | 6.20128 |
| TMEM253 | 2.245451 | 3.800437 | 4.876166 | 2.43E-06 | 1.39E-05 | 4.142401 |
| HES2 | 2.244635 | 3.481662 | 3.160538 | 0.001859 | 0.005249 | -2.11781 |
| MYC | 2.244386 | 10.7588 | 5.697388 | 5.12E-08 | 4.31E-07 | 7.862018 |
| LRRC46 | 2.242683 | 6.650282 | 4.773488 | 3.83E-06 | 2.07E-05 | 3.707501 |
| RAD54L | 2.242609 | 7.97647 | 4.842934 | 2.82E-06 | 1.58E-05 | 4.000878 |
| SQOR | 2.241031 | 9.3293 | 5.601888 | 8.18E-08 | 6.58E-07 | 7.408486 |
| AL049839.2 | 2.236106 | 6.700214 | 3.342379 | 0.001017 | 0.003063 | -1.5626 |
| PARP9 | 2.235442 | 11.6195 | 6.673035 | 3.24E-10 | 4.69E-09 | 12.77701 |
| HTRA3 | 2.235387 | 8.216099 | 4.221861 | 3.90E-05 | 0.000168 | 1.495568 |
| C5orf46 | 2.233882 | 2.320656 | 2.293254 | 0.023033 | 0.04818 | -4.37535 |
| HAL | 2.232683 | 3.440984 | 3.761724 | 0.000231 | 0.000823 | -0.17962 |
| IL13RA2 | 2.228795 | 9.142618 | 2.416105 | 0.016725 | 0.036389 | -4.096 |
| CER1 | 2.228629 | 2.444484 | 3.005757 | 0.003042 | 0.008164 | -2.56858 |
| RTP4 | 2.228517 | 7.790359 | 4.598002 | 8.19E-06 | 4.13E-05 | 2.980724 |
| LILRA2 | 2.228247 | 8.515546 | 4.894823 | 2.24E-06 | 1.29E-05 | 4.22218 |
| LAPTM5 | 2.224023 | 13.97661 | 5.008643 | 1.34E-06 | 8.13E-06 | 4.713823 |
| CXCR6 | 2.223759 | 4.761681 | 3.206354 | 0.001601 | 0.004594 | -1.98052 |
| SLFN13 | 2.222551 | 7.136888 | 3.595922 | 0.000421 | 0.001404 | -0.74324 |
| ETV2 | 2.222266 | 5.608823 | 5.382567 | 2.36E-07 | 1.72E-06 | 6.387177 |
| C11orf88 | 2.221903 | 3.597444 | 2.520522 | 0.01262 | 0.028486 | -3.84788 |
| RPS15A | 2.22121 | 13.69929 | 8.018491 | 1.52E-13 | 5.27E-12 | 20.26125 |
| HYAL2 | 2.220187 | 11.52356 | 9.474769 | 1.99E-17 | 1.87E-15 | 29.02797 |
| ZNF93 | 2.219731 | 8.268966 | 6.406756 | 1.35E-09 | 1.68E-08 | 11.38747 |
| GPC2 | 2.218433 | 8.958386 | 3.346187 | 0.001004 | 0.003033 | -1.55068 |
| CDCA3 | 2.215402 | 8.945801 | 6.073873 | 7.68E-09 | 7.90E-08 | 9.699793 |
| RHAG | 2.215217 | 2.271536 | 4.75393 | 4.17E-06 | 2.24E-05 | 3.625464 |
| C11orf70 | 2.214374 | 6.641633 | 2.883591 | 0.00443 | 0.01133 | -2.90995 |
| IGSF5 | 2.213387 | 6.354593 | 4.360809 | 2.22E-05 | 0.000101 | 2.032409 |
| TM6SF2 | 2.21141 | 5.960656 | 4.363133 | 2.20E-05 | 0.0001 | 2.041506 |
| IL15 | 2.209333 | 6.304694 | 4.310341 | 2.73E-05 | 0.000122 | 1.835802 |
| FMO1 | 2.209065 | 4.4595 | 3.638774 | 0.000361 | 0.001223 | -0.59966 |
| MYL12A | 2.208526 | 12.32785 | 6.492259 | 8.57E-10 | 1.12E-08 | 11.82994 |
| RGS1 | 2.207779 | 11.7888 | 3.503772 | 0.000584 | 0.001867 | -1.04705 |
| NCF2 | 2.20621 | 9.489839 | 5.386983 | 2.31E-07 | 1.69E-06 | 6.407457 |
| CLDN23 | 2.202744 | 6.096972 | 3.625308 | 0.000379 | 0.001279 | -0.64494 |
| COL6A1 | 2.198593 | 13.9011 | 4.014021 | 8.87E-05 | 0.00035 | 0.719065 |
| OAS1 | 2.198098 | 10.38882 | 3.592734 | 0.000426 | 0.001417 | -0.75387 |
| LDLRAD2 | 2.197376 | 5.496342 | 4.481565 | 1.34E-05 | 6.45E-05 | 2.510241 |
| DZIP1L | 2.19713 | 7.683039 | 8.142 | 7.29E-14 | 2.73E-12 | 20.9817 |
| ARSJ | 2.196161 | 8.811621 | 3.170738 | 0.001798 | 0.005092 | -2.0874 |
| TSPAN10 | 2.195669 | 6.640789 | 5.852044 | 2.37E-08 | 2.16E-07 | 8.607491 |
| AP000781.2 | 2.193022 | 2.409901 | 4.451159 | 1.52E-05 | 7.23E-05 | 2.388948 |
| CATSPER1 | 2.191751 | 4.750212 | 3.741763 | 0.000248 | 0.000879 | -0.24862 |
| LAIR1 | 2.189173 | 10.79604 | 4.582825 | 8.74E-06 | 4.38E-05 | 2.918863 |
| CD3D | 2.185247 | 4.988279 | 3.178251 | 0.001755 | 0.004982 | -2.06494 |
| LCTL | 2.184728 | 7.409071 | 4.535875 | 1.07E-05 | 5.26E-05 | 2.72851 |
| SNRPG | 2.184562 | 11.18657 | 7.012557 | 5.01E-11 | 8.85E-10 | 14.59629 |
| TEAD4 | 2.184392 | 8.245937 | 4.698736 | 5.31E-06 | 2.78E-05 | 3.395345 |
| LYPLA1 | 2.18389 | 11.54093 | 7.453943 | 4.12E-12 | 9.68E-11 | 17.03469 |
| TNFAIP8 | 2.18275 | 8.059475 | 4.958497 | 1.68E-06 | 9.98E-06 | 4.496174 |
| KIRREL | 2.181773 | 10.64911 | 5.384064 | 2.34E-07 | 1.71E-06 | 6.394052 |
| PLSCR1 | 2.181011 | 11.19326 | 5.614099 | 7.70E-08 | 6.23E-07 | 7.466183 |
| SP6 | 2.179685 | 6.327333 | 4.080082 | 6.85E-05 | 0.000278 | 0.962383 |
| MAD2L1 | 2.178298 | 10.1774 | 5.274007 | 3.94E-07 | 2.71E-06 | 5.89237 |
| GMNN | 2.17779 | 10.17198 | 7.123872 | 2.69E-11 | 5.10E-10 | 15.20372 |
| FNDC3B | 2.176363 | 10.89828 | 6.660227 | 3.47E-10 | 4.99E-09 | 12.70941 |
| ATP6V0E1 | 2.175761 | 12.8471 | 8.684105 | 2.74E-15 | 1.52E-13 | 24.19865 |
| RPS18 | 2.175383 | 15.36659 | 6.58445 | 5.23E-10 | 7.15E-09 | 12.31099 |
| GPNMB | 2.175119 | 12.18084 | 3.750348 | 0.00024 | 0.000854 | -0.21898 |
| FAM109B | 2.17499 | 8.444165 | 5.482036 | 1.46E-07 | 1.12E-06 | 6.846832 |
| ITGA1 | 2.173844 | 10.58052 | 5.623131 | 7.37E-08 | 5.98E-07 | 7.508917 |
| IL1RAP | 2.172679 | 11.28305 | 3.78171 | 0.000214 | 0.000771 | -0.11022 |
| PTBP1 | 2.172155 | 13.04136 | 10.31852 | 9.10E-20 | 1.56E-17 | 34.32041 |
| VAMP5 | 2.171466 | 11.19513 | 4.553623 | 9.90E-06 | 4.91E-05 | 2.800285 |
| AC011005.1 | 2.171135 | 5.690319 | 7.261821 | 1.23E-11 | 2.56E-10 | 15.96363 |
| TKTL1 | 2.171075 | 5.951277 | 2.595722 | 0.010248 | 0.023714 | -3.66315 |
| SNCAIP | 2.170434 | 9.895132 | 4.524469 | 1.12E-05 | 5.49E-05 | 2.682498 |
| STK33 | 2.169768 | 9.325158 | 5.203453 | 5.47E-07 | 3.64E-06 | 5.574686 |
| FZD2 | 2.168993 | 8.286579 | 5.794303 | 3.16E-08 | 2.80E-07 | 8.327598 |
| PLA2G5 | 2.168769 | 9.431867 | 2.510029 | 0.012987 | 0.029205 | -3.87326 |
| MNS1 | 2.167523 | 8.101568 | 5.119795 | 8.06E-07 | 5.15E-06 | 5.20204 |
| RAC2 | 2.166801 | 9.344167 | 5.028218 | 1.23E-06 | 7.50E-06 | 4.799228 |
| MFNG | 2.163956 | 9.886099 | 6.613496 | 4.47E-10 | 6.23E-09 | 12.46338 |
| CARD6 | 2.161052 | 8.405156 | 5.30761 | 3.36E-07 | 2.36E-06 | 6.044756 |
| IFI16 | 2.160791 | 12.04274 | 5.606345 | 8.00E-08 | 6.44E-07 | 7.429539 |
| TYMP | 2.158806 | 10.50932 | 4.532092 | 1.08E-05 | 5.34E-05 | 2.713238 |
| PIFO | 2.158103 | 9.851459 | 3.543118 | 0.000508 | 0.00165 | -0.91817 |
| CD28 | 2.15566 | 4.979469 | 3.761501 | 0.000231 | 0.000823 | -0.18039 |
| STK17A | 2.154632 | 11.61901 | 5.917644 | 1.70E-08 | 1.61E-07 | 8.927725 |
| PCDHB1 | 2.152984 | 5.034636 | 3.149615 | 0.001925 | 0.005423 | -2.15029 |
| STAB1 | 2.152825 | 12.35835 | 4.324703 | 2.57E-05 | 0.000116 | 1.891566 |
| HLA-DOA | 2.152609 | 10.52502 | 4.336683 | 2.45E-05 | 0.000111 | 1.938191 |
| TAP1 | 2.15135 | 12.09559 | 5.356072 | 2.67E-07 | 1.93E-06 | 6.265753 |
| NEDD4 | 2.15105 | 9.310599 | 8.215812 | 4.69E-14 | 1.85E-12 | 21.41457 |
| LAMA4 | 2.149649 | 11.63061 | 5.032845 | 1.20E-06 | 7.36E-06 | 4.819452 |
| MAP7D3 | 2.148715 | 8.996183 | 7.127089 | 2.64E-11 | 5.02E-10 | 15.22135 |
| CASZ1 | 2.147676 | 6.899752 | 4.486855 | 1.31E-05 | 6.32E-05 | 2.531407 |
| GZMK | 2.146517 | 4.286919 | 3.005097 | 0.003048 | 0.008179 | -2.57045 |
| TNFSF8 | 2.145512 | 6.974762 | 4.183198 | 4.56E-05 | 0.000193 | 1.348695 |
| ARHGDIB | 2.144564 | 12.42694 | 6.135697 | 5.58E-09 | 5.96E-08 | 10.00892 |
| C1S | 2.14438 | 12.69527 | 3.628234 | 0.000375 | 0.001267 | -0.63511 |
| UBQLNL | 2.144283 | 4.246964 | 3.8206 | 0.000185 | 0.000678 | 0.025714 |
| MCM5 | 2.144217 | 11.33232 | 7.578454 | 2.01E-12 | 5.12E-11 | 17.73643 |
| MPZL2 | 2.143869 | 7.705906 | 4.21538 | 4.00E-05 | 0.000172 | 1.470871 |
| MAGEA12 | 2.143418 | 4.671348 | 4.000537 | 9.34E-05 | 0.000366 | 0.669805 |
| YBX3 | 2.142712 | 11.84965 | 6.247265 | 3.13E-09 | 3.56E-08 | 10.5718 |
| CACNA2D4 | 2.140458 | 7.659185 | 4.807739 | 3.29E-06 | 1.81E-05 | 3.85179 |
| ATRAID | 2.140324 | 12.47896 | 8.164026 | 6.39E-14 | 2.45E-12 | 21.11068 |
| FCN3 | 2.138327 | 5.176036 | 3.238116 | 0.001442 | 0.00419 | -1.88431 |
| TBX21 | 2.137437 | 3.945197 | 3.754414 | 0.000237 | 0.000843 | -0.20492 |
| DNALI1 | 2.137358 | 11.19969 | 5.292697 | 3.60E-07 | 2.51E-06 | 5.977042 |
| ERI1 | 2.136517 | 10.21895 | 8.418126 | 1.38E-14 | 6.24E-13 | 22.60957 |
| STIL | 2.135567 | 8.795516 | 6.31344 | 2.21E-09 | 2.62E-08 | 10.90868 |
| TRIP13 | 2.135068 | 8.92079 | 5.507092 | 1.30E-07 | 1.00E-06 | 6.96355 |
| C1QTNF6 | 2.134403 | 9.684819 | 4.743798 | 4.36E-06 | 2.33E-05 | 3.583065 |
| MMRN1 | 2.132101 | 7.942736 | 4.392687 | 1.94E-05 | 9.01E-05 | 2.157541 |
| AC013470.2 | 2.129903 | 2.19763 | 2.405473 | 0.017203 | 0.037269 | -4.12072 |
| BGN | 2.129713 | 13.51589 | 5.065174 | 1.04E-06 | 6.46E-06 | 4.961137 |
| DNAJC5B | 2.129543 | 3.889188 | 3.537045 | 0.000519 | 0.001683 | -0.93814 |
| TMEM176B | 2.128543 | 11.9219 | 3.719399 | 0.000269 | 0.000943 | -0.32556 |
| FAM92B | 2.126426 | 3.017334 | 2.329708 | 0.020973 | 0.04439 | -4.29389 |
| FCGR1A | 2.126099 | 9.95891 | 4.270413 | 3.21E-05 | 0.000141 | 1.681559 |
| LY9 | 2.126044 | 3.898821 | 4.029637 | 8.35E-05 | 0.000331 | 0.776288 |
| FP565260.6 | 2.125666 | 6.984727 | 3.790845 | 0.000207 | 0.000748 | -0.07839 |
| RARRES1 | 2.125264 | 7.494792 | 2.792971 | 0.005809 | 0.014404 | -3.15487 |
| SP100 | 2.125151 | 10.57041 | 5.259697 | 4.21E-07 | 2.88E-06 | 5.827685 |
| CD8B | 2.123256 | 4.359729 | 3.188627 | 0.001696 | 0.004835 | -2.03385 |
| ZYX | 2.123154 | 13.77721 | 6.024471 | 9.89E-09 | 9.90E-08 | 9.454239 |
| RNF135 | 2.122804 | 9.769614 | 7.220215 | 1.56E-11 | 3.17E-10 | 15.73362 |
| LRGUK | 2.121109 | 5.887402 | 4.934531 | 1.87E-06 | 1.10E-05 | 4.392734 |
| PDLIM3 | 2.120321 | 11.32647 | 4.789083 | 3.58E-06 | 1.95E-05 | 3.7731 |
| RNASE10 | 2.120041 | 2.425163 | 4.297737 | 2.87E-05 | 0.000128 | 1.786989 |
| ODF3B | 2.119125 | 7.516674 | 4.046888 | 7.80E-05 | 0.000312 | 0.839713 |
| MT2A | 2.11894 | 14.22547 | 4.184044 | 4.54E-05 | 0.000193 | 1.351898 |
| PXDNL | 2.117208 | 6.703686 | 3.206233 | 0.001601 | 0.004595 | -1.98088 |
| CLIC4 | 2.116 | 14.40967 | 7.520682 | 2.81E-12 | 6.84E-11 | 17.4101 |
| KCNJ15 | 2.115766 | 4.783887 | 2.970643 | 0.003393 | 0.008979 | -2.66801 |
| IGFLR1 | 2.11569 | 7.173173 | 5.796833 | 3.12E-08 | 2.77E-07 | 8.33982 |
| SIRPB2 | 2.115683 | 8.070438 | 4.15299 | 5.14E-05 | 0.000215 | 1.234706 |
| ANKRD53 | 2.115192 | 6.671262 | 4.569301 | 9.26E-06 | 4.62E-05 | 2.863873 |
| GPR84 | 2.114796 | 6.939082 | 3.647639 | 0.00035 | 0.001188 | -0.56977 |
| TRIP6 | 2.114378 | 12.03436 | 6.281514 | 2.61E-09 | 3.03E-08 | 10.74587 |
| DPPA4 | 2.114257 | 3.418233 | 3.011009 | 0.002993 | 0.008042 | -2.55361 |
| CCDC140 | 2.113569 | 2.052835 | 2.519419 | 0.012658 | 0.028556 | -3.85055 |
| NBPF10 | 2.112971 | 6.619487 | 5.507716 | 1.29E-07 | 9.98E-07 | 6.96646 |
| TCF12 | 2.111956 | 13.21876 | 6.459113 | 1.02E-09 | 1.31E-08 | 11.65799 |
| SERF1A | 2.110203 | 3.762814 | 3.446662 | 0.000712 | 0.002229 | -1.23191 |
| ZNF311 | 2.109279 | 7.611565 | 4.854742 | 2.67E-06 | 1.52E-05 | 4.05108 |
| AC036214.3 | 2.108482 | 3.74345 | 4.606279 | 7.91E-06 | 4.00E-05 | 3.014529 |
| CD33 | 2.107541 | 8.371176 | 4.620916 | 7.43E-06 | 3.77E-05 | 3.074424 |
| ADAMTS9 | 2.107364 | 10.42833 | 4.325856 | 2.56E-05 | 0.000115 | 1.896047 |
| FANCC | 2.107315 | 9.218777 | 6.842731 | 1.28E-10 | 2.05E-09 | 13.67987 |
| TEAD3 | 2.103848 | 9.098118 | 5.010257 | 1.33E-06 | 8.08E-06 | 4.720855 |
| GLIPR2 | 2.103664 | 11.80407 | 6.388141 | 1.49E-09 | 1.83E-08 | 11.29161 |
| SNAI2 | 2.103635 | 8.547236 | 3.637682 | 0.000363 | 0.001228 | -0.60333 |
| C1orf112 | 2.103556 | 8.411682 | 8.345144 | 2.15E-14 | 9.26E-13 | 22.17707 |
| FAM20A | 2.102692 | 8.868524 | 3.438716 | 0.000732 | 0.002284 | -1.25742 |
| ALPK2 | 2.099565 | 5.813996 | 2.352294 | 0.019779 | 0.042196 | -4.24281 |
| MICB | 2.099482 | 7.524775 | 4.925437 | 1.95E-06 | 1.14E-05 | 4.353582 |
| NUP37 | 2.098979 | 9.439066 | 8.457568 | 1.09E-14 | 5.04E-13 | 22.84396 |
| LRRC74A | 2.096433 | 2.789981 | 4.792351 | 3.52E-06 | 1.93E-05 | 3.786867 |
| ZMYND10 | 2.096281 | 8.463277 | 2.904998 | 0.004151 | 0.010711 | -2.85106 |
| SERPING1 | 2.095335 | 12.9166 | 4.171005 | 4.78E-05 | 0.000202 | 1.302604 |
| PLEKHN1 | 2.09445 | 3.033016 | 3.325106 | 0.001078 | 0.003227 | -1.61652 |
| CEBPD | 2.093405 | 11.51845 | 4.641758 | 6.79E-06 | 3.47E-05 | 3.159969 |
| BPIFB4 | 2.09292 | 2.445244 | 2.311347 | 0.021989 | 0.04625 | -4.33507 |
| GLI2 | 2.092642 | 8.698139 | 3.917194 | 0.000129 | 0.000489 | 0.368396 |
| LRRC71 | 2.091987 | 4.388251 | 3.596519 | 0.00042 | 0.001401 | -0.74125 |
| NXT2 | 2.091809 | 9.899726 | 6.678089 | 3.15E-10 | 4.58E-09 | 12.80372 |
| SRGN | 2.091455 | 12.31077 | 4.150941 | 5.18E-05 | 0.000216 | 1.227 |
| RPS7 | 2.088479 | 14.14716 | 7.199396 | 1.76E-11 | 3.52E-10 | 15.61879 |
| EVX1 | 2.088403 | 2.526073 | 2.752265 | 0.006548 | 0.016017 | -3.26256 |
| ODC1 | 2.087719 | 13.02864 | 6.710093 | 2.65E-10 | 3.90E-09 | 12.97306 |
| SIGLEC14 | 2.086986 | 7.9442 | 3.00435 | 0.003056 | 0.008196 | -2.57258 |
| DPYSL3 | 2.08666 | 14.81981 | 5.954228 | 1.41E-08 | 1.37E-07 | 9.107337 |
| SPON2 | 2.086394 | 9.201743 | 3.618132 | 0.000389 | 0.001309 | -0.66901 |
| SOX2 | 2.08619 | 13.59674 | 5.257845 | 4.25E-07 | 2.90E-06 | 5.819323 |
| DSCC1 | 2.085822 | 8.46795 | 7.566103 | 2.16E-12 | 5.42E-11 | 17.66656 |
| PLIN2 | 2.085474 | 11.23926 | 3.881368 | 0.000147 | 0.000552 | 0.24046 |
| TRIP10 | 2.083024 | 10.3289 | 8.222655 | 4.50E-14 | 1.79E-12 | 21.45478 |
| BCHE | 2.082686 | 11.12425 | 4.031222 | 8.29E-05 | 0.000329 | 0.782104 |
| RPS10 | 2.080098 | 12.92109 | 6.608538 | 4.59E-10 | 6.38E-09 | 12.43734 |
| UCP2 | 2.078746 | 11.34744 | 5.150708 | 6.99E-07 | 4.54E-06 | 5.339223 |
| EMILIN3 | 2.076498 | 9.514114 | 2.372192 | 0.018778 | 0.040313 | -4.19743 |
| GINS3 | 2.075901 | 9.116823 | 7.153296 | 2.28E-11 | 4.41E-10 | 15.36515 |
| CENPL | 2.07378 | 8.758797 | 7.322893 | 8.72E-12 | 1.88E-10 | 16.30252 |
| ADGRL4 | 2.073546 | 9.902748 | 5.7726 | 3.52E-08 | 3.08E-07 | 8.222874 |
| OSTC | 2.073194 | 11.38691 | 6.732045 | 2.35E-10 | 3.53E-09 | 13.08948 |
| CLEC10A | 2.073021 | 5.082962 | 2.628754 | 0.009339 | 0.021878 | -3.58042 |
| MCM7 | 2.072911 | 12.67512 | 5.837862 | 2.54E-08 | 2.30E-07 | 8.538575 |
| PRSS23 | 2.072441 | 11.1528 | 4.466101 | 1.43E-05 | 6.84E-05 | 2.448471 |
| CTSK | 2.071551 | 9.834949 | 3.601967 | 0.000412 | 0.001378 | -0.72308 |
| TMEM218 | 2.070276 | 10.36936 | 7.62684 | 1.52E-12 | 3.97E-11 | 18.01069 |
| RPL29 | 2.070009 | 14.10338 | 9.195134 | 1.15E-16 | 8.97E-15 | 27.30349 |
| TCAF2 | 2.06928 | 7.960189 | 3.866984 | 0.000156 | 0.000579 | 0.18937 |
| WNT5A | 2.067768 | 10.34836 | 5.180793 | 6.08E-07 | 4.01E-06 | 5.473313 |
| ARHGEF19 | 2.065545 | 7.091933 | 5.26542 | 4.10E-07 | 2.81E-06 | 5.853538 |
| MASP1 | 2.065172 | 11.27938 | 3.263424 | 0.001326 | 0.003885 | -1.80705 |
| AC007906.2 | 2.065148 | 3.947319 | 3.024579 | 0.002868 | 0.007741 | -2.51485 |
| GRAP | 2.06514 | 5.977475 | 5.358433 | 2.64E-07 | 1.91E-06 | 6.276557 |
| ERAP2 | 2.063773 | 9.744955 | 2.67627 | 0.008158 | 0.019474 | -3.45972 |
| BAZ1A | 2.063388 | 10.01112 | 7.895292 | 3.16E-13 | 9.88E-12 | 19.54761 |
| CD14 | 2.061818 | 12.56459 | 3.87072 | 0.000153 | 0.000572 | 0.202625 |
| MS4A14 | 2.059786 | 6.578801 | 4.112179 | 6.04E-05 | 0.000248 | 1.081781 |
| MBOAT1 | 2.059022 | 6.460019 | 4.505301 | 1.21E-05 | 5.91E-05 | 2.60538 |
| FPR1 | 2.058565 | 10.73664 | 3.281626 | 0.001248 | 0.003679 | -1.75115 |
| ANO6 | 2.05807 | 11.94926 | 7.583659 | 1.95E-12 | 4.98E-11 | 17.76589 |
| TLE6 | 2.056196 | 6.820698 | 3.8489 | 0.000167 | 0.000616 | 0.125366 |
| PHLDA2 | 2.055788 | 6.409063 | 2.827716 | 0.00524 | 0.013151 | -3.0618 |
| HFE | 2.055147 | 8.855662 | 4.569754 | 9.24E-06 | 4.61E-05 | 2.865716 |
| CNN2 | 2.054571 | 10.22064 | 3.791308 | 0.000207 | 0.000747 | -0.07678 |
| NBPF14 | 2.053805 | 8.388991 | 6.177192 | 4.50E-09 | 4.93E-08 | 10.21752 |
| AC138811.2 | 2.053743 | 3.862376 | 4.423606 | 1.71E-05 | 8.02E-05 | 2.279602 |
| CDCA5 | 2.051974 | 9.838265 | 4.792677 | 3.52E-06 | 1.93E-05 | 3.788243 |
| DPP4 | 2.051127 | 7.86234 | 2.663025 | 0.008473 | 0.020094 | -3.49357 |
| OMP | 2.05092 | 2.24089 | 4.195986 | 4.33E-05 | 0.000184 | 1.397153 |
| RIPOR3 | 2.050365 | 8.299729 | 3.474708 | 0.000646 | 0.002045 | -1.14145 |
| EFEMP2 | 2.050351 | 12.04631 | 4.984609 | 1.49E-06 | 8.98E-06 | 4.609303 |
| TLR3 | 2.049913 | 8.71741 | 5.130619 | 7.67E-07 | 4.92E-06 | 5.250008 |
| LAMB2 | 2.048612 | 13.23782 | 6.564974 | 5.80E-10 | 7.87E-09 | 12.20902 |
| TREML2 | 2.048198 | 3.328954 | 3.000797 | 0.00309 | 0.008275 | -2.58269 |
| VCAN | 2.047104 | 13.29597 | 5.700372 | 5.04E-08 | 4.25E-07 | 7.876273 |
| TTC26 | 2.045448 | 9.181749 | 6.966951 | 6.45E-11 | 1.12E-09 | 14.34895 |
| CCDC15 | 2.044375 | 7.220602 | 5.861478 | 2.26E-08 | 2.07E-07 | 8.653398 |
| IL2RB | 2.041921 | 6.526139 | 3.846378 | 0.000168 | 0.000621 | 0.116461 |
| ICAM3 | 2.040791 | 4.836451 | 4.996097 | 1.42E-06 | 8.57E-06 | 4.659214 |
| LAMB1 | 2.040305 | 11.79028 | 3.762841 | 0.00023 | 0.00082 | -0.17575 |
| ACTA2 | 2.040292 | 11.38735 | 4.044379 | 7.88E-05 | 0.000315 | 0.830472 |
| PALLD | 2.039171 | 12.67374 | 7.440674 | 4.45E-12 | 1.04E-10 | 16.96025 |
| POPDC2 | 2.039012 | 7.1071 | 7.195961 | 1.79E-11 | 3.58E-10 | 15.59986 |
| PARP15 | 2.03796 | 5.685844 | 4.52225 | 1.13E-05 | 5.54E-05 | 2.673556 |
| RPL10 | 2.03781 | 15.2676 | 10.1933 | 2.04E-19 | 3.08E-17 | 33.52758 |
| RPS14 | 2.03722 | 14.63517 | 8.358585 | 1.98E-14 | 8.59E-13 | 22.25661 |
| REEP4 | 2.035533 | 9.828084 | 8.575181 | 5.33E-15 | 2.74E-13 | 23.54551 |
| CDK15 | 2.035211 | 4.411847 | 3.205071 | 0.001607 | 0.004609 | -1.98438 |
| ZNF683 | 2.034651 | 3.247933 | 3.172726 | 0.001786 | 0.005063 | -2.08146 |
| TBXA2R | 2.033836 | 7.591945 | 5.080902 | 9.64E-07 | 6.05E-06 | 5.030309 |
| HSD3B7 | 2.033771 | 8.546516 | 5.047692 | 1.12E-06 | 6.94E-06 | 4.884436 |
| SAMD9L | 2.032183 | 10.57021 | 4.116611 | 5.94E-05 | 0.000244 | 1.09833 |
| PHLDA1 | 2.031493 | 13.04047 | 5.15874 | 6.74E-07 | 4.40E-06 | 5.374966 |
| PRCP | 2.030429 | 13.25691 | 6.365164 | 1.68E-09 | 2.05E-08 | 11.17353 |
| TCF7 | 2.030336 | 8.508842 | 5.672983 | 5.77E-08 | 4.80E-07 | 7.745621 |
| TBX4 | 2.029637 | 2.946336 | 3.016552 | 0.002941 | 0.007921 | -2.5378 |
| ABCA1 | 2.029245 | 12.11178 | 5.221027 | 5.04E-07 | 3.37E-06 | 5.653525 |
| HIST1H1D | 2.028935 | 2.756685 | 3.210172 | 0.001581 | 0.004541 | -1.969 |
| HIST1H3J | 2.028619 | 2.212731 | 3.032972 | 0.002793 | 0.007556 | -2.49079 |
| GUSB | 2.027992 | 11.72331 | 7.306156 | 9.59E-12 | 2.04E-10 | 16.2095 |
| SMIM11A | 2.026816 | 2.718549 | 5.947928 | 1.46E-08 | 1.41E-07 | 9.076357 |
| A2M | 2.025701 | 15.28122 | 5.230397 | 4.83E-07 | 3.25E-06 | 5.695641 |
| MAML2 | 2.024796 | 11.13876 | 7.571142 | 2.10E-12 | 5.29E-11 | 17.69506 |
| KLF17 | 2.024141 | 4.059507 | 2.944985 | 0.003673 | 0.009631 | -2.74 |
| FOXD2 | 2.023768 | 5.160072 | 3.575523 | 0.000453 | 0.001493 | -0.81108 |
| TEX26 | 2.021345 | 6.766626 | 2.811279 | 0.005502 | 0.013747 | -3.10596 |
| TLR2 | 2.020299 | 10.05081 | 4.30767 | 2.76E-05 | 0.000123 | 1.825449 |
| CCL25 | 2.019949 | 3.458465 | 3.37876 | 0.000899 | 0.002743 | -1.44824 |
| SAGE1 | 2.019702 | 2.207733 | 3.039313 | 0.002738 | 0.00742 | -2.47257 |
| IL17B | 2.019186 | 3.395936 | 3.674941 | 0.000317 | 0.001089 | -0.47733 |
| PLK4 | 2.018385 | 8.293242 | 5.069512 | 1.02E-06 | 6.34E-06 | 4.980199 |
| CPA6 | 2.01809 | 3.255102 | 3.044627 | 0.002693 | 0.007313 | -2.45729 |
| PPP1R2P3 | 2.017949 | 2.916382 | 5.312126 | 3.29E-07 | 2.32E-06 | 6.065291 |
| EDNRA | 2.015879 | 9.943515 | 4.789251 | 3.57E-06 | 1.95E-05 | 3.773808 |
| PTN | 2.015628 | 14.80635 | 4.316176 | 2.66E-05 | 0.000119 | 1.85844 |
| RARRES3 | 2.015326 | 11.07357 | 3.757236 | 0.000234 | 0.000835 | -0.19516 |
| DTX3L | 2.012832 | 11.1143 | 5.950445 | 1.44E-08 | 1.39E-07 | 9.08873 |
| KNTC1 | 2.012381 | 9.668535 | 5.825432 | 2.71E-08 | 2.43E-07 | 8.478264 |
| NME4 | 2.011834 | 12.18154 | 9.966574 | 8.74E-19 | 1.19E-16 | 32.09821 |
| LYPD1 | 2.011343 | 10.96968 | 4.081614 | 6.81E-05 | 0.000276 | 0.968062 |
| COL14A1 | 2.011276 | 8.840315 | 2.6519 | 0.008746 | 0.020651 | -3.52188 |
| CYBA | 2.010871 | 11.59495 | 4.893966 | 2.25E-06 | 1.30E-05 | 4.21851 |
| UCN2 | 2.009648 | 3.792148 | 2.797117 | 0.005738 | 0.014253 | -3.14382 |
| TGIF2-C20orf24 | 2.008974 | 2.500275 | 3.796307 | 0.000203 | 0.000735 | -0.05934 |
| ADPRH | 2.008373 | 9.071752 | 7.29666 | 1.01E-11 | 2.14E-10 | 16.15677 |
| ADAM8 | 2.007806 | 8.571875 | 3.212705 | 0.001568 | 0.004507 | -1.96134 |
| IGFBP5 | 2.007714 | 15.26137 | 3.77336 | 0.000221 | 0.000792 | -0.13925 |
| HIST1H2AM | 2.005912 | 2.584771 | 3.206946 | 0.001597 | 0.004587 | -1.97873 |
| NXF3 | 2.004814 | 3.589012 | 3.359521 | 0.00096 | 0.002911 | -1.50886 |
| HSPB1 | 2.004671 | 13.50848 | 5.691283 | 5.27E-08 | 4.42E-07 | 7.832868 |
| PSORS1C1 | 2.003693 | 6.587119 | 3.210305 | 0.00158 | 0.00454 | -1.96859 |
| FREM2 | 2.003228 | 9.745979 | 3.014309 | 0.002962 | 0.00797 | -2.5442 |
| DTHD1 | 2.002626 | 4.635198 | 2.344954 | 0.02016 | 0.042883 | -4.25946 |
| ATP5L2 | 2.001752 | 3.071356 | 4.897206 | 2.21E-06 | 1.28E-05 | 4.232387 |
| FABP7 | 2.00166 | 13.18521 | 2.894586 | 0.004284 | 0.011003 | -2.87975 |
| METTL11B | 2.00042 | 2.201752 | 2.815494 | 0.005434 | 0.013599 | -3.09466 |
| SSR2 | 2.000133 | 13.09637 | 8.446796 | 1.16E-14 | 5.34E-13 | 22.7799 |
| KCNJ13 | -2.00025 | 1.624506 | -3.47397 | 0.000648 | 0.00205 | -1.14384 |
| PRR36 | -2.00156 | 9.491175 | -3.25848 | 0.001348 | 0.003944 | -1.82217 |
| RTN2 | -2.00158 | 9.100279 | -6.20657 | 3.86E-09 | 4.31E-08 | 10.36574 |
| SHC3 | -2.00204 | 10.82319 | -4.37092 | 2.13E-05 | 9.75E-05 | 2.072017 |
| ZFP92 | -2.0025 | 6.84524 | -5.24858 | 4.43E-07 | 3.02E-06 | 5.777499 |
| SPINK9 | -2.00283 | 0.985419 | -4.98455 | 1.49E-06 | 8.98E-06 | 4.609054 |
| CRY2 | -2.00343 | 10.49396 | -7.39993 | 5.62E-12 | 1.26E-10 | 16.73212 |
| BAIAP2L2 | -2.00375 | 6.148652 | -6.18214 | 4.39E-09 | 4.83E-08 | 10.24244 |
| HIVEP2 | -2.00399 | 10.31609 | -6.26635 | 2.83E-09 | 3.25E-08 | 10.66872 |
| HERC1 | -2.00648 | 10.89919 | -8.47043 | 1.01E-14 | 4.72E-13 | 22.92049 |
| MSC | -2.00736 | 6.544 | -3.21438 | 0.001559 | 0.004485 | -1.9563 |
| PREPL | -2.00867 | 12.07888 | -10.2395 | 1.51E-19 | 2.40E-17 | 33.81988 |
| ANKRD34B | -2.00943 | 2.424619 | -3.34337 | 0.001014 | 0.003057 | -1.5595 |
| RYR1 | -2.01038 | 9.008654 | -4.52962 | 1.10E-05 | 5.39E-05 | 2.703259 |
| SERTAD4 | -2.01099 | 6.40734 | -2.85841 | 0.004779 | 0.012105 | -2.97873 |
| WNT7A | -2.01113 | 7.581812 | -2.71116 | 0.007379 | 0.017833 | -3.36984 |
| RIMS4 | -2.01135 | 9.77672 | -2.69174 | 0.007804 | 0.018729 | -3.41999 |
| PLEKHG3 | -2.01145 | 9.708978 | -4.67481 | 5.89E-06 | 3.05E-05 | 3.296237 |
| FAM124A | -2.01205 | 8.657814 | -5.08701 | 9.38E-07 | 5.90E-06 | 5.057219 |
| CALM1 | -2.01354 | 15.08253 | -7.32842 | 8.45E-12 | 1.83E-10 | 16.33325 |
| ARHGEF33 | -2.01438 | 5.063578 | -7.42897 | 4.76E-12 | 1.10E-10 | 16.89466 |
| KRT77 | -2.01566 | 0.554696 | -5.43032 | 1.88E-07 | 1.40E-06 | 6.607101 |
| BTBD9 | -2.01566 | 9.919758 | -7.81339 | 5.11E-13 | 1.50E-11 | 19.07605 |
| SPEF2 | -2.01586 | 6.904348 | -2.94114 | 0.003716 | 0.009733 | -2.75075 |
| UCMA | -2.01637 | 0.438811 | -5.6066 | 7.99E-08 | 6.44E-07 | 7.430744 |
| KCNK10 | -2.01837 | 7.616056 | -3.45188 | 0.000699 | 0.002196 | -1.21513 |
| HTR7 | -2.01848 | 5.124824 | -4.38384 | 2.02E-05 | 9.32E-05 | 2.122758 |
| THEG | -2.0204 | 0.950317 | -5.20329 | 5.48E-07 | 3.64E-06 | 5.573936 |
| MLIP | -2.02042 | 7.039067 | -2.62533 | 0.009429 | 0.022064 | -3.58903 |
| DGKZ | -2.02112 | 11.93932 | -7.68049 | 1.11E-12 | 3.03E-11 | 18.31582 |
| OPRL1 | -2.02164 | 8.631295 | -7.29682 | 1.01E-11 | 2.14E-10 | 16.15767 |
| TNR | -2.02434 | 11.2583 | -2.28451 | 0.023553 | 0.049069 | -4.39472 |
| TMEFF1 | -2.02438 | 5.009892 | -3.53667 | 0.00052 | 0.001685 | -0.93937 |
| NHLH2 | -2.02443 | 4.62204 | -2.4385 | 0.015756 | 0.034554 | -4.04362 |
| GDAP1 | -2.02463 | 10.25525 | -7.53444 | 2.59E-12 | 6.38E-11 | 17.48768 |
| YJEFN3 | -2.02666 | 8.066457 | -4.049 | 7.74E-05 | 0.00031 | 0.84751 |
| GABRQ | -2.02679 | 7.792944 | -2.48891 | 0.013756 | 0.030715 | -3.92403 |
| LDB2 | -2.02748 | 9.303307 | -4.76477 | 3.98E-06 | 2.15E-05 | 3.670918 |
| C10orf67 | -2.02757 | 2.70215 | -3.17288 | 0.001786 | 0.005062 | -2.08101 |
| PLK2 | -2.02785 | 10.07838 | -4.70606 | 5.14E-06 | 2.70E-05 | 3.425756 |
| PTK2B | -2.02811 | 11.16907 | -5.6728 | 5.78E-08 | 4.80E-07 | 7.744754 |
| PRKN | -2.02846 | 8.118808 | -8.00545 | 1.64E-13 | 5.62E-12 | 20.18548 |
| SLC16A7 | -2.02856 | 7.653457 | -3.49261 | 0.000607 | 0.001933 | -1.08339 |
| FLT3 | -2.02976 | 3.685704 | -3.44359 | 0.00072 | 0.00225 | -1.24176 |
| TPRG1L | -2.03098 | 10.93843 | -7.66035 | 1.25E-12 | 3.35E-11 | 18.20115 |
| ACOT7 | -2.03235 | 10.91292 | -6.19802 | 4.04E-09 | 4.49E-08 | 10.32257 |
| TRIM58 | -2.0329 | 3.79829 | -3.57064 | 0.000461 | 0.001515 | -0.82726 |
| MIGA1 | -2.03399 | 9.743747 | -10.2527 | 1.39E-19 | 2.22E-17 | 33.90359 |
| DLX1 | -2.03487 | 7.762146 | -2.5417 | 0.011907 | 0.027075 | -3.79637 |
| CTIF | -2.03936 | 10.92143 | -6.84865 | 1.24E-10 | 1.99E-09 | 13.71161 |
| TSPYL4 | -2.04091 | 11.64488 | -7.18351 | 1.92E-11 | 3.79E-10 | 15.53128 |
| KLC2 | -2.04128 | 10.76851 | -8.48899 | 9.00E-15 | 4.29E-13 | 23.031 |
| AIPL1 | -2.04207 | 1.320678 | -4.17554 | 4.70E-05 | 0.000199 | 1.319725 |
| DKK2 | -2.04256 | 5.015693 | -2.89797 | 0.004241 | 0.010909 | -2.87045 |
| HR | -2.04342 | 9.390314 | -3.59656 | 0.00042 | 0.001401 | -0.74111 |
| SLC22A25 | -2.04354 | 1.057846 | -4.39468 | 1.93E-05 | 8.95E-05 | 2.165371 |
| PNPLA7 | -2.04414 | 8.496087 | -5.05709 | 1.08E-06 | 6.67E-06 | 4.925623 |
| FAM50B | -2.04495 | 8.465043 | -4.16298 | 4.94E-05 | 0.000207 | 1.272324 |
| ANKS4B | -2.04576 | 0.825853 | -6.11003 | 6.37E-09 | 6.72E-08 | 9.880342 |
| SH3TC2 | -2.04734 | 6.967917 | -4.257 | 3.39E-05 | 0.000148 | 1.63001 |
| HHLA2 | -2.04756 | 1.746297 | -4.36613 | 2.17E-05 | 9.92E-05 | 2.053226 |
| SYNGR4 | -2.04997 | 3.195844 | -3.85267 | 0.000164 | 0.000608 | 0.138701 |
| IQCA1 | -2.0514 | 7.322977 | -2.9244 | 0.003912 | 0.010184 | -2.79734 |
| PIP4K2A | -2.05241 | 11.09879 | -5.25948 | 4.21E-07 | 2.88E-06 | 5.826705 |
| RELL2 | -2.05254 | 7.466238 | -6.58576 | 5.19E-10 | 7.11E-09 | 12.31786 |
| ENO2 | -2.05294 | 13.2482 | -5.2635 | 4.14E-07 | 2.83E-06 | 5.844843 |
| OGN | -2.05308 | 6.236017 | -2.36228 | 0.019271 | 0.041236 | -4.22007 |
| IQANK1 | -2.05373 | 3.378662 | -3.03896 | 0.002741 | 0.007426 | -2.47359 |
| BIN1 | -2.0542 | 12.22861 | -6.00806 | 1.08E-08 | 1.07E-07 | 9.372955 |
| PCP4 | -2.0546 | 7.273856 | -2.85184 | 0.004875 | 0.012318 | -2.99657 |
| APBB1 | -2.0552 | 12.30339 | -8.1401 | 7.37E-14 | 2.76E-12 | 20.97056 |
| IFNA6 | -2.05607 | 0.249152 | -7.8636 | 3.80E-13 | 1.16E-11 | 19.36487 |
| WFDC1 | -2.05736 | 6.968498 | -3.77406 | 0.00022 | 0.00079 | -0.13683 |
| COL25A1 | -2.05761 | 5.67201 | -2.40109 | 0.017403 | 0.03767 | -4.13087 |
| CADM2 | -2.05806 | 11.1589 | -3.15453 | 0.001895 | 0.005342 | -2.1357 |
| TEX101 | -2.05836 | 1.299814 | -2.95562 | 0.003554 | 0.009354 | -2.71022 |
| GRK3 | -2.05875 | 9.456302 | -7.35767 | 7.15E-12 | 1.58E-10 | 16.49618 |
| ZNF215 | -2.05908 | 3.90274 | -3.77477 | 0.00022 | 0.000789 | -0.13437 |
| TCHH | -2.05911 | 3.546919 | -3.38075 | 0.000893 | 0.002725 | -1.44196 |
| MICAL3 | -2.05933 | 9.678505 | -5.16525 | 6.54E-07 | 4.28E-06 | 5.403951 |
| GFRA4 | -2.06011 | 0.425961 | -7.03139 | 4.51E-11 | 8.13E-10 | 14.69869 |
| NKX1-2 | -2.06041 | 2.932312 | -2.48352 | 0.013958 | 0.031097 | -3.93693 |
| NAT8L | -2.06112 | 11.84786 | -4.29389 | 2.92E-05 | 0.000129 | 1.772095 |
| TMEM229A | -2.06115 | 6.451565 | -2.50471 | 0.013177 | 0.029571 | -3.88607 |
| FAM126B | -2.06226 | 9.243255 | -11.1776 | 3.40E-22 | 1.12E-19 | 39.81186 |
| NIPSNAP3B | -2.06417 | 6.199553 | -5.82032 | 2.78E-08 | 2.50E-07 | 8.453497 |
| SLC30A8 | -2.0642 | 1.17718 | -3.97765 | 0.000102 | 0.000397 | 0.586526 |
| CAMK2G | -2.06745 | 11.35803 | -6.085 | 7.25E-09 | 7.53E-08 | 9.755295 |
| CD177 | -2.06768 | 2.289171 | -2.46857 | 0.014534 | 0.032237 | -3.97256 |
| MADD | -2.06935 | 11.39203 | -8.67612 | 2.87E-15 | 1.58E-13 | 24.15068 |
| OBSCN | -2.07007 | 7.592746 | -4.14921 | 5.22E-05 | 0.000218 | 1.220487 |
| MAB21L3 | -2.07104 | 0.993479 | -4.61274 | 7.69E-06 | 3.89E-05 | 3.040966 |
| AK7 | -2.07417 | 5.096678 | -2.98157 | 0.00328 | 0.008721 | -2.63717 |
| TRAM1L1 | -2.07844 | 7.111987 | -2.97619 | 0.003335 | 0.008845 | -2.65237 |
| CALML3 | -2.07937 | 0.472844 | -5.52027 | 1.22E-07 | 9.45E-07 | 7.025084 |
| DNAH6 | -2.0811 | 5.909372 | -3.35292 | 0.000982 | 0.00297 | -1.52959 |
| PALM2-AKAP2 | -2.08133 | 3.1414 | -2.82925 | 0.005216 | 0.013093 | -3.05768 |
| PRDM12 | -2.08177 | 2.737031 | -3.60873 | 0.000402 | 0.001348 | -0.70049 |
| RGR | -2.08212 | 6.497684 | -2.87488 | 0.004548 | 0.011595 | -2.93381 |
| WDR17 | -2.08461 | 8.70254 | -6.21165 | 3.76E-09 | 4.22E-08 | 10.39139 |
| FRMPD1 | -2.08543 | 5.996291 | -3.87803 | 0.000149 | 0.000558 | 0.228605 |
| TCEAL2 | -2.08584 | 10.17901 | -3.32661 | 0.001073 | 0.003211 | -1.61184 |
| AKR1C2 | -2.08585 | 5.165226 | -3.06943 | 0.00249 | 0.006821 | -2.38561 |
| RAB6C | -2.08696 | 4.163516 | -5.86274 | 2.24E-08 | 2.06E-07 | 8.659535 |
| LIPE | -2.08932 | 8.673173 | -4.74978 | 4.25E-06 | 2.28E-05 | 3.608087 |
| C19orf81 | -2.08961 | 5.462698 | -4.10007 | 6.34E-05 | 0.000258 | 1.036642 |
| MMD2 | -2.08971 | 7.701929 | -2.55016 | 0.011632 | 0.026512 | -3.77568 |
| YPEL2 | -2.09064 | 9.518545 | -8.78064 | 1.51E-15 | 8.95E-14 | 24.78015 |
| NPPC | -2.09129 | 4.246985 | -2.5899 | 0.010416 | 0.024053 | -3.67764 |
| KLHL2 | -2.09144 | 10.24557 | -7.75238 | 7.30E-13 | 2.06E-11 | 18.72627 |
| SLC26A9 | -2.09292 | 4.278133 | -3.49114 | 0.00061 | 0.001942 | -1.08817 |
| IL9 | -2.09489 | 0.818853 | -4.33261 | 2.49E-05 | 0.000112 | 1.922313 |
| GNAZ | -2.09511 | 10.28006 | -6.26592 | 2.84E-09 | 3.26E-08 | 10.66653 |
| GAL3ST1 | -2.09777 | 7.452455 | -3.08303 | 0.002384 | 0.006562 | -2.34606 |
| SEMA3G | -2.09814 | 7.479299 | -4.32925 | 2.52E-05 | 0.000114 | 1.909254 |
| DNAAF1 | -2.09831 | 6.527004 | -3.57797 | 0.000449 | 0.001482 | -0.80298 |
| CAP2 | -2.09916 | 10.66187 | -7.06479 | 3.74E-11 | 6.86E-10 | 14.88066 |
| RIMKLA | -2.09945 | 8.791356 | -4.63025 | 7.13E-06 | 3.63E-05 | 3.11269 |
| PNMA8A | -2.09975 | 11.52711 | -8.43196 | 1.27E-14 | 5.79E-13 | 22.69172 |
| PLEKHG5 | -2.1007 | 9.21633 | -5.95822 | 1.39E-08 | 1.34E-07 | 9.126958 |
| FAM135B | -2.10218 | 8.447123 | -4.01706 | 8.76E-05 | 0.000346 | 0.730171 |
| SP9 | -2.10262 | 4.660463 | -2.48206 | 0.014014 | 0.031209 | -3.94041 |
| ANKRD65 | -2.10303 | 8.08511 | -4.26496 | 3.28E-05 | 0.000144 | 1.660589 |
| KLHL11 | -2.10537 | 6.581588 | -5.01035 | 1.33E-06 | 8.08E-06 | 4.721247 |
| FNDC10 | -2.10615 | 7.804192 | -4.02647 | 8.45E-05 | 0.000335 | 0.764656 |
| RPS6KL1 | -2.10623 | 9.108429 | -4.49006 | 1.29E-05 | 6.26E-05 | 2.544238 |
| TMEM59L | -2.10676 | 12.14379 | -5.73035 | 4.35E-08 | 3.72E-07 | 8.019782 |
| SRRM3 | -2.10786 | 8.216186 | -3.45855 | 0.000683 | 0.00215 | -1.19364 |
| BCL2L10 | -2.10933 | 2.266766 | -4.50705 | 1.21E-05 | 5.87E-05 | 2.61241 |
| KIAA1217 | -2.11047 | 8.927393 | -4.72697 | 4.69E-06 | 2.49E-05 | 3.512807 |
| RHBDL1 | -2.11096 | 7.4423 | -4.59538 | 8.28E-06 | 4.17E-05 | 2.97003 |
| N4BP3 | -2.11113 | 6.929152 | -5.35691 | 2.66E-07 | 1.92E-06 | 6.26958 |
| CRLF1 | -2.11148 | 7.668833 | -2.94049 | 0.003724 | 0.009747 | -2.75256 |
| TBC1D30 | -2.11227 | 7.964238 | -5.93148 | 1.59E-08 | 1.51E-07 | 8.995578 |
| HCRTR1 | -2.11236 | 2.491378 | -3.63863 | 0.000361 | 0.001224 | -0.60014 |
| MYH14 | -2.11504 | 9.740213 | -3.16687 | 0.001821 | 0.005151 | -2.09893 |
| OTOL1 | -2.12029 | 0.771532 | -5.28795 | 3.69E-07 | 2.56E-06 | 5.955516 |
| RGS14 | -2.12068 | 8.32099 | -5.07012 | 1.01E-06 | 6.32E-06 | 4.982867 |
| ELOVL4 | -2.12199 | 8.518172 | -5.6786 | 5.61E-08 | 4.69E-07 | 7.772402 |
| LDHD | -2.12286 | 8.175443 | -5.04913 | 1.11E-06 | 6.89E-06 | 4.890752 |
| AC131097.2 | -2.12297 | 3.196319 | -2.83515 | 0.005125 | 0.012889 | -3.04177 |
| MOAP1 | -2.12569 | 11.25609 | -8.46204 | 1.06E-14 | 4.93E-13 | 22.87053 |
| EPHX4 | -2.12669 | 6.903407 | -5.59394 | 8.50E-08 | 6.83E-07 | 7.370991 |
| FAM131C | -2.12883 | 5.944284 | -3.5557 | 0.000486 | 0.001587 | -0.8767 |
| KLHL38 | -2.13139 | 2.657763 | -4.31802 | 2.64E-05 | 0.000118 | 1.865613 |
| CGREF1 | -2.13302 | 9.06137 | -4.0436 | 7.90E-05 | 0.000315 | 0.827599 |
| DGKI | -2.13384 | 8.78993 | -4.41989 | 1.74E-05 | 8.12E-05 | 2.264913 |
| COL6A6 | -2.13633 | 2.766188 | -4.59665 | 8.24E-06 | 4.15E-05 | 2.975223 |
| IQSEC1 | -2.13654 | 11.57155 | -7.78575 | 6.01E-13 | 1.74E-11 | 18.91739 |
| GPC5 | -2.13668 | 7.569662 | -3.22728 | 0.001494 | 0.004322 | -1.91722 |
| PLEKHA6 | -2.13753 | 9.828754 | -3.82499 | 0.000182 | 0.000668 | 0.041125 |
| ZNF804B | -2.13767 | 1.340582 | -3.69119 | 0.000299 | 0.001033 | -0.42205 |
| CNTN1 | -2.13774 | 11.78968 | -3.0676 | 0.002504 | 0.006858 | -2.3909 |
| USH1C | -2.13824 | 6.680213 | -2.33274 | 0.020809 | 0.044081 | -4.28706 |
| KBTBD11 | -2.13859 | 10.79992 | -5.09508 | 9.04E-07 | 5.70E-06 | 5.092785 |
| CYP2A6 | -2.13943 | 2.386593 | -3.77375 | 0.00022 | 0.000791 | -0.13789 |
| RASGRP1 | -2.1401 | 8.970079 | -4.04659 | 7.81E-05 | 0.000312 | 0.838602 |
| LY6D | -2.14146 | 0.606098 | -5.44896 | 1.72E-07 | 1.29E-06 | 6.693316 |
| STRC | -2.14159 | 2.540575 | -4.14979 | 5.20E-05 | 0.000217 | 1.222675 |
| FAXC | -2.14263 | 9.004731 | -5.85292 | 2.36E-08 | 2.15E-07 | 8.611774 |
| NIPA1 | -2.14363 | 10.3326 | -7.81792 | 4.97E-13 | 1.47E-11 | 19.10206 |
| MYO15A | -2.14393 | 5.504438 | -5.03296 | 1.20E-06 | 7.36E-06 | 4.819945 |
| FAM171A1 | -2.14418 | 10.81844 | -6.28345 | 2.59E-09 | 3.00E-08 | 10.75575 |
| RHCG | -2.14451 | 5.460586 | -3.84613 | 0.000168 | 0.000621 | 0.115594 |
| MYOM2 | -2.14715 | 8.802525 | -6.34033 | 1.92E-09 | 2.30E-08 | 11.0462 |
| DRP2 | -2.1475 | 8.62512 | -4.38808 | 1.98E-05 | 9.17E-05 | 2.139411 |
| PALM | -2.1476 | 11.4719 | -4.53387 | 1.08E-05 | 5.30E-05 | 2.720401 |
| EMX2 | -2.15031 | 7.774623 | -3.21671 | 0.001547 | 0.004456 | -1.94925 |
| LCN8 | -2.15222 | 1.515749 | -4.01918 | 8.69E-05 | 0.000344 | 0.737949 |
| SLIT1 | -2.15375 | 11.15338 | -2.94654 | 0.003655 | 0.009594 | -2.73566 |
| LZTS3 | -2.15466 | 11.0423 | -6.08346 | 7.31E-09 | 7.57E-08 | 9.747581 |
| MAPRE3 | -2.15533 | 11.21563 | -7.97363 | 1.99E-13 | 6.63E-12 | 20.0008 |
| FMN1 | -2.15682 | 7.312297 | -4.8463 | 2.78E-06 | 1.57E-05 | 4.015178 |
| SH3PXD2A | -2.15774 | 10.29668 | -5.01033 | 1.33E-06 | 8.08E-06 | 4.721185 |
| RPRM | -2.15987 | 7.41776 | -2.39666 | 0.017608 | 0.038067 | -4.14113 |
| RGS11 | -2.16207 | 8.795586 | -3.41869 | 0.000784 | 0.002426 | -1.32148 |
| KRTAP5-7 | -2.16283 | 1.627938 | -4.72717 | 4.69E-06 | 2.49E-05 | 3.513636 |
| PCDHGB5 | -2.16343 | 6.101949 | -2.73313 | 0.006923 | 0.016842 | -3.31267 |
| CAMKK2 | -2.16418 | 11.04324 | -10.6977 | 7.80E-21 | 1.76E-18 | 36.73408 |
| LPO | -2.16516 | 3.340541 | -4.71467 | 4.95E-06 | 2.61E-05 | 3.461549 |
| EPOP | -2.16616 | 8.126067 | -5.17693 | 6.19E-07 | 4.07E-06 | 5.456058 |
| ASTL | -2.16623 | 1.248154 | -4.04725 | 7.79E-05 | 0.000312 | 0.841063 |
| BEX2 | -2.16982 | 10.18882 | -4.12441 | 5.76E-05 | 0.000237 | 1.127469 |
| MAPT | -2.17117 | 12.78482 | -4.11624 | 5.94E-05 | 0.000244 | 1.096936 |
| HDAC11 | -2.17232 | 10.24306 | -7.45792 | 4.03E-12 | 9.50E-11 | 17.05701 |
| MYL2 | -2.17395 | 0.538521 | -6.28699 | 2.54E-09 | 2.96E-08 | 10.77375 |
| GRASP | -2.17434 | 8.878954 | -6.74187 | 2.23E-10 | 3.37E-09 | 13.14166 |
| MESP2 | -2.17644 | 2.907027 | -3.58725 | 0.000434 | 0.001441 | -0.77212 |
| LMO3 | -2.17684 | 10.79947 | -3.07427 | 0.002452 | 0.006727 | -2.37154 |
| RND1 | -2.17736 | 8.883804 | -4.83241 | 2.95E-06 | 1.65E-05 | 3.95621 |
| ELMOD1 | -2.17778 | 9.72679 | -3.66412 | 0.00033 | 0.001127 | -0.51405 |
| PPP1R9A | -2.17905 | 9.65649 | -4.49829 | 1.25E-05 | 6.06E-05 | 2.577239 |
| RYR3 | -2.17963 | 8.968632 | -2.97227 | 0.003376 | 0.008939 | -2.66343 |
| LRP11 | -2.17982 | 9.76054 | -8.76257 | 1.69E-15 | 9.95E-14 | 24.67114 |
| GDAP1L1 | -2.17999 | 8.973135 | -2.96677 | 0.003434 | 0.009072 | -2.67892 |
| TCTE1 | -2.18073 | 4.372576 | -3.88208 | 0.000147 | 0.00055 | 0.242989 |
| XIRP1 | -2.18121 | 4.91526 | -3.11625 | 0.002144 | 0.005961 | -2.24887 |
| TCEAL5 | -2.18171 | 9.134319 | -4.67392 | 5.91E-06 | 3.05E-05 | 3.292553 |
| MYPN | -2.18239 | 1.979204 | -4.49992 | 1.24E-05 | 6.02E-05 | 2.583776 |
| RGS8 | -2.18261 | 7.048258 | -3.6975 | 0.000292 | 0.001013 | -0.40053 |
| TPO | -2.1827 | 1.596869 | -4.13469 | 5.53E-05 | 0.000229 | 1.165972 |
| TDRD6 | -2.18281 | 5.418965 | -4.50485 | 1.22E-05 | 5.91E-05 | 2.603562 |
| ADGRB3 | -2.18415 | 9.824115 | -4.77957 | 3.73E-06 | 2.03E-05 | 3.733046 |
| ME1 | -2.18687 | 8.694575 | -3.46704 | 0.000663 | 0.002095 | -1.16625 |
| ELAVL3 | -2.18766 | 10.76522 | -3.4373 | 0.000735 | 0.002294 | -1.26196 |
| GPR158 | -2.18825 | 9.390882 | -4.50473 | 1.22E-05 | 5.91E-05 | 2.60308 |
| NDRG4 | -2.19269 | 13.03751 | -4.93459 | 1.87E-06 | 1.10E-05 | 4.393007 |
| ADRA1A | -2.19283 | 5.596725 | -2.9151 | 0.004025 | 0.010437 | -2.82313 |
| PCSK6 | -2.19347 | 7.997778 | -2.87564 | 0.004537 | 0.011573 | -2.93173 |
| LCN12 | -2.19396 | 5.089382 | -3.35237 | 0.000984 | 0.002975 | -1.53132 |
| GJB1 | -2.19441 | 8.129249 | -2.30504 | 0.022348 | 0.046928 | -4.34915 |
| PDP1 | -2.19471 | 10.14411 | -7.60681 | 1.71E-12 | 4.40E-11 | 17.89702 |
| LRRC8B | -2.19546 | 10.24111 | -7.18509 | 1.90E-11 | 3.77E-10 | 15.53996 |
| CENPVL3 | -2.19596 | 0.875627 | -4.23729 | 3.67E-05 | 0.000159 | 1.554484 |
| ANXA8L1 | -2.19769 | 2.040686 | -3.2276 | 0.001493 | 0.004318 | -1.91626 |
| LMTK2 | -2.19772 | 10.29282 | -8.03903 | 1.35E-13 | 4.72E-12 | 20.38069 |
| RIIAD1 | -2.19958 | 5.345013 | -4.1716 | 4.77E-05 | 0.000201 | 1.304836 |
| TF | -2.20008 | 11.99476 | -2.79259 | 0.005816 | 0.014418 | -3.15589 |
| KCNJ11 | -2.20079 | 7.158748 | -3.81477 | 0.000189 | 0.000692 | 0.00526 |
| LNX1 | -2.20232 | 8.674335 | -4.38214 | 2.03E-05 | 9.37E-05 | 2.116067 |
| ADARB1 | -2.20347 | 9.240858 | -7.0095 | 5.09E-11 | 8.98E-10 | 14.57969 |
| PLG | -2.20457 | 0.794973 | -5.74522 | 4.04E-08 | 3.49E-07 | 8.091153 |
| C2orf82 | -2.20831 | 7.928911 | -3.31821 | 0.001104 | 0.003297 | -1.63797 |
| AAK1 | -2.20848 | 10.72216 | -8.62355 | 3.96E-15 | 2.10E-13 | 23.83516 |
| KIAA2012 | -2.20853 | 4.386815 | -3.47595 | 0.000643 | 0.002038 | -1.13744 |
| OMG | -2.21111 | 10.11883 | -3.76681 | 0.000226 | 0.000809 | -0.162 |
| FAM83H | -2.21385 | 6.900542 | -4.57555 | 9.01E-06 | 4.51E-05 | 2.889247 |
| CELF6 | -2.21412 | 1.773822 | -4.44367 | 1.57E-05 | 7.44E-05 | 2.359161 |
| ASXL3 | -2.21595 | 7.323178 | -3.72221 | 0.000267 | 0.000935 | -0.31592 |
| YWHAH | -2.21747 | 13.20335 | -8.81915 | 1.19E-15 | 7.28E-14 | 25.01282 |
| ADGRA1 | -2.21981 | 9.151448 | -3.19873 | 0.001641 | 0.004695 | -2.00349 |
| ATP9A | -2.22076 | 12.50595 | -6.82257 | 1.43E-10 | 2.27E-09 | 13.5719 |
| KLHL35 | -2.2222 | 6.029287 | -3.45477 | 0.000692 | 0.002177 | -1.20583 |
| ACY3 | -2.22289 | 6.396288 | -3.1835 | 0.001725 | 0.004906 | -2.04923 |
| CDH16 | -2.22503 | 0.587919 | -6.00962 | 1.07E-08 | 1.06E-07 | 9.380693 |
| RBM24 | -2.22531 | 6.279555 | -4.18005 | 4.61E-05 | 0.000195 | 1.336786 |
| VAMP1 | -2.22721 | 9.17624 | -6.21199 | 3.76E-09 | 4.21E-08 | 10.39312 |
| NIPAL3 | -2.22798 | 10.79265 | -5.67779 | 5.64E-08 | 4.70E-07 | 7.768497 |
| CAPN3 | -2.23201 | 7.068623 | -3.68271 | 0.000308 | 0.001062 | -0.45094 |
| PRCD | -2.23349 | 5.965134 | -4.2305 | 3.77E-05 | 0.000163 | 1.528525 |
| S100A1 | -2.2341 | 10.01364 | -3.48205 | 0.00063 | 0.001999 | -1.11766 |
| HPN | -2.23426 | 5.460302 | -4.24216 | 3.60E-05 | 0.000156 | 1.573123 |
| RXRG | -2.23445 | 6.181456 | -2.86592 | 0.004673 | 0.011862 | -2.95828 |
| DOCK5 | -2.23494 | 8.691097 | -4.17237 | 4.76E-05 | 0.000201 | 1.30775 |
| PTPRD | -2.23619 | 10.74337 | -4.98982 | 1.46E-06 | 8.78E-06 | 4.631948 |
| FUT1 | -2.23711 | 5.58837 | -6.85097 | 1.23E-10 | 1.97E-09 | 13.72406 |
| DBNDD2 | -2.24135 | 8.639104 | -4.36651 | 2.17E-05 | 9.91E-05 | 2.054735 |
| CPNE5 | -2.24361 | 10.42295 | -3.94393 | 0.000116 | 0.000446 | 0.464509 |
| STPG1 | -2.2442 | 8.002614 | -4.15736 | 5.05E-05 | 0.000212 | 1.251138 |
| NAP1L5 | -2.24591 | 10.17581 | -8.32608 | 2.41E-14 | 1.03E-12 | 22.06439 |
| AKAP6 | -2.2491 | 10.8125 | -5.8702 | 2.16E-08 | 2.00E-07 | 8.695867 |
| DUSP9 | -2.24957 | 5.039307 | -3.01257 | 0.002978 | 0.008009 | -2.54916 |
| PLK5 | -2.24958 | 4.427217 | -4.14999 | 5.20E-05 | 0.000217 | 1.223442 |
| AC083902.2 | -2.25051 | 2.128784 | -3.70614 | 0.000283 | 0.000985 | -0.37099 |
| SLC5A8 | -2.25239 | 0.393587 | -8.65855 | 3.20E-15 | 1.73E-13 | 24.0451 |
| TMEM171 | -2.25352 | 3.537608 | -3.9208 | 0.000127 | 0.000483 | 0.381313 |
| FBLN2 | -2.25357 | 8.265043 | -3.04354 | 0.002702 | 0.007335 | -2.46042 |
| CYP7B1 | -2.25466 | 5.27984 | -4.02966 | 8.35E-05 | 0.000331 | 0.776365 |
| PCDHA9 | -2.25593 | 2.983716 | -3.05793 | 0.002582 | 0.007037 | -2.4189 |
| CCL3L1 | -2.25788 | 6.648826 | -2.48108 | 0.014051 | 0.031281 | -3.94275 |
| AATK-AS1 | -2.26021 | 1.020457 | -5.3028 | 3.44E-07 | 2.41E-06 | 6.022884 |
| KCNH4 | -2.26092 | 4.995895 | -3.73956 | 0.00025 | 0.000886 | -0.25622 |
| CACNA1G | -2.26114 | 8.817082 | -3.86163 | 0.000159 | 0.000589 | 0.170406 |
| PLCB1 | -2.26224 | 9.85376 | -4.8756 | 2.44E-06 | 1.39E-05 | 4.139986 |
| ACTRT3 | -2.26226 | 4.305284 | -3.69472 | 0.000295 | 0.001022 | -0.40999 |
| DSG3 | -2.26231 | 0.658748 | -5.21635 | 5.16E-07 | 3.45E-06 | 5.632515 |
| TSPYL1 | -2.26303 | 11.7778 | -10.2378 | 1.53E-19 | 2.41E-17 | 33.80899 |
| DNAJC12 | -2.26403 | 8.12926 | -6.27492 | 2.71E-09 | 3.13E-08 | 10.71229 |
| MEF2C | -2.26417 | 11.36056 | -8.67167 | 2.95E-15 | 1.61E-13 | 24.12395 |
| PPP1R3F | -2.26787 | 9.356439 | -8.57038 | 5.48E-15 | 2.81E-13 | 23.51679 |
| PAQR9 | -2.26796 | 4.299107 | -2.86633 | 0.004667 | 0.011855 | -2.95714 |
| BAAT | -2.26896 | 3.478263 | -4.60338 | 8.00E-06 | 4.04E-05 | 3.002698 |
| CORO2A | -2.26976 | 6.41461 | -4.83807 | 2.88E-06 | 1.62E-05 | 3.980238 |
| DLG3 | -2.27121 | 9.805995 | -8.09185 | 9.84E-14 | 3.58E-12 | 20.68855 |
| WASF1 | -2.27133 | 10.61727 | -5.2768 | 3.88E-07 | 2.69E-06 | 5.905003 |
| SPRYD3 | -2.27217 | 11.5565 | -8.89853 | 7.30E-16 | 4.74E-14 | 25.49358 |
| KRT19 | -2.27614 | 3.289652 | -3.42111 | 0.000778 | 0.002408 | -1.31375 |
| SFTPD | -2.27642 | 4.254027 | -3.84712 | 0.000168 | 0.00062 | 0.119068 |
| FAM13C | -2.2771 | 8.619261 | -7.13596 | 2.51E-11 | 4.81E-10 | 15.27 |
| CADPS | -2.27911 | 10.40661 | -3.61429 | 0.000394 | 0.001324 | -0.68186 |
| PLEKHA1 | -2.28152 | 9.786537 | -10.3906 | 5.71E-20 | 1.03E-17 | 34.77795 |
| AMER2 | -2.28306 | 10.68941 | -3.81213 | 0.000191 | 0.000698 | -0.00398 |
| RANBP17 | -2.28536 | 4.592776 | -3.44963 | 0.000705 | 0.00221 | -1.22236 |
| COL24A1 | -2.28934 | 5.252997 | -3.27107 | 0.001292 | 0.003797 | -1.7836 |
| PGM2L1 | -2.28981 | 10.96433 | -5.7471 | 4.00E-08 | 3.46E-07 | 8.100162 |
| MPO | -2.29058 | 4.171893 | -3.44873 | 0.000707 | 0.002215 | -1.22526 |
| GPR63 | -2.2906 | 6.014981 | -4.30764 | 2.76E-05 | 0.000123 | 1.825312 |
| ANXA3 | -2.29118 | 5.425194 | -3.83064 | 0.000178 | 0.000655 | 0.060987 |
| PELI3 | -2.2917 | 8.93606 | -8.16087 | 6.51E-14 | 2.47E-12 | 21.09222 |
| PLP1 | -2.29241 | 14.46719 | -2.83347 | 0.00515 | 0.012947 | -3.0463 |
| KANK4 | -2.29339 | 3.978177 | -2.99092 | 0.003186 | 0.008498 | -2.61073 |
| PNMA8C | -2.29566 | 7.200069 | -5.78987 | 3.23E-08 | 2.86E-07 | 8.30619 |
| MYH11 | -2.29637 | 7.710087 | -4.11427 | 5.99E-05 | 0.000246 | 1.0896 |
| ZDHHC22 | -2.29763 | 9.579286 | -2.91729 | 0.003998 | 0.010374 | -2.81707 |
| NCAM2 | -2.29796 | 10.63371 | -4.34277 | 2.39E-05 | 0.000108 | 1.961905 |
| LARGE1 | -2.30001 | 10.21185 | -7.40116 | 5.58E-12 | 1.25E-10 | 16.73899 |
| TNFSF9 | -2.30146 | 5.322736 | -4.37762 | 2.07E-05 | 9.53E-05 | 2.098295 |
| OR13J1 | -2.30331 | 1.906153 | -4.66278 | 6.20E-06 | 3.19E-05 | 3.246537 |
| GPR88 | -2.30648 | 6.62975 | -3.1758 | 0.001769 | 0.005017 | -2.07228 |
| ERICH5 | -2.30658 | 2.193836 | -4.6689 | 6.04E-06 | 3.12E-05 | 3.271821 |
| SH2D1B | -2.30905 | 2.855538 | -4.57202 | 9.15E-06 | 4.57E-05 | 2.874922 |
| PPEF1 | -2.3094 | 5.949853 | -4.03256 | 8.25E-05 | 0.000328 | 0.787001 |
| MAP3K10 | -2.30998 | 10.11337 | -7.84847 | 4.16E-13 | 1.25E-11 | 19.27776 |
| ARX | -2.31001 | 7.624367 | -3.2372 | 0.001446 | 0.0042 | -1.8871 |
| C3orf49 | -2.31034 | 3.5175 | -7.03029 | 4.54E-11 | 8.17E-10 | 14.69268 |
| SNX32 | -2.31439 | 7.784431 | -6.43566 | 1.16E-09 | 1.46E-08 | 11.53666 |
| NR4A2 | -2.31483 | 7.823114 | -5.23075 | 4.82E-07 | 3.25E-06 | 5.69722 |
| 3-Sep | -2.31532 | 12.14808 | -4.43926 | 1.60E-05 | 7.56E-05 | 2.341667 |
| PRSS16 | -2.31862 | 3.276685 | -3.07299 | 0.002462 | 0.006752 | -2.37527 |
| PCDHA12 | -2.31913 | 3.502144 | -3.3284 | 0.001067 | 0.003194 | -1.60624 |
| DLK2 | -2.3196 | 6.016917 | -5.1163 | 8.20E-07 | 5.23E-06 | 5.186554 |
| TACR2 | -2.32013 | 4.894376 | -6.7114 | 2.63E-10 | 3.88E-09 | 12.97998 |
| EFNA5 | -2.32021 | 7.360408 | -3.67099 | 0.000321 | 0.001103 | -0.49076 |
| VAT1L | -2.32022 | 9.485482 | -2.875 | 0.004546 | 0.011593 | -2.93349 |
| CALM3 | -2.32112 | 14.21219 | -9.94992 | 9.72E-19 | 1.31E-16 | 31.99353 |
| GUCY1B3 | -2.3228 | 9.998999 | -5.99513 | 1.15E-08 | 1.13E-07 | 9.309016 |
| KCNIP3 | -2.32664 | 9.052093 | -4.52415 | 1.12E-05 | 5.50E-05 | 2.681227 |
| NRG4 | -2.32691 | 3.964996 | -4.97059 | 1.59E-06 | 9.48E-06 | 4.54852 |
| SYNJ1 | -2.32736 | 10.47522 | -10.5442 | 2.11E-20 | 4.08E-18 | 35.75467 |
| TMEM52 | -2.32823 | 3.253527 | -3.36467 | 0.000943 | 0.002865 | -1.49267 |
| LMO7 | -2.32825 | 8.976629 | -7.65028 | 1.32E-12 | 3.53E-11 | 18.14389 |
| ANK1 | -2.32831 | 7.580262 | -4.36045 | 2.22E-05 | 0.000101 | 2.031021 |
| TTC6 | -2.33128 | 1.290275 | -3.78756 | 0.000209 | 0.000757 | -0.08986 |
| RASL11B | -2.33377 | 7.490707 | -3.06248 | 0.002545 | 0.006951 | -2.40575 |
| RUNDC3B | -2.33597 | 7.718635 | -5.81871 | 2.80E-08 | 2.51E-07 | 8.445659 |
| TMOD2 | -2.34222 | 11.8647 | -7.06313 | 3.78E-11 | 6.92E-10 | 14.8716 |
| SH3BP5 | -2.34317 | 7.987293 | -5.74337 | 4.07E-08 | 3.52E-07 | 8.082246 |
| C10orf35 | -2.34524 | 8.17869 | -5.24706 | 4.47E-07 | 3.03E-06 | 5.770672 |
| C9orf24 | -2.34831 | 6.278343 | -2.81496 | 0.005443 | 0.013615 | -3.0961 |
| MYCBP2 | -2.34885 | 10.85282 | -9.20128 | 1.11E-16 | 8.70E-15 | 27.3412 |
| RAB6B | -2.34912 | 12.13099 | -6.01264 | 1.05E-08 | 1.05E-07 | 9.395599 |
| GNAI1 | -2.34984 | 9.961501 | -4.78448 | 3.65E-06 | 1.99E-05 | 3.753713 |
| CFC1 | -2.35122 | 1.104256 | -4.67038 | 6.00E-06 | 3.10E-05 | 3.277908 |
| SLC25A22 | -2.35155 | 10.76271 | -8.2724 | 3.34E-14 | 1.37E-12 | 21.74759 |
| BCAS1 | -2.35278 | 10.61056 | -2.5404 | 0.01195 | 0.027154 | -3.79955 |
| MYH13 | -2.35314 | 0.758238 | -5.63465 | 6.97E-08 | 5.68E-07 | 7.563487 |
| KIF17 | -2.35364 | 7.629109 | -7.08181 | 3.40E-11 | 6.32E-10 | 14.97359 |
| KIF3A | -2.35509 | 10.4939 | -9.56248 | 1.14E-17 | 1.16E-15 | 29.57221 |
| FABP3 | -2.35529 | 9.293063 | -5.82594 | 2.70E-08 | 2.43E-07 | 8.480748 |
| CDYL2 | -2.35532 | 8.445595 | -7.12128 | 2.73E-11 | 5.16E-10 | 15.18949 |
| BFSP1 | -2.35602 | 5.383575 | -5.01224 | 1.32E-06 | 8.02E-06 | 4.729498 |
| KLC3 | -2.35707 | 1.962531 | -5.19658 | 5.65E-07 | 3.75E-06 | 5.543895 |
| DDX25 | -2.35755 | 8.096998 | -3.55173 | 0.000493 | 0.001605 | -0.8898 |
| ARHGEF28 | -2.35856 | 7.572917 | -5.86785 | 2.19E-08 | 2.02E-07 | 8.684422 |
| EML5 | -2.35918 | 4.347998 | -4.15061 | 5.19E-05 | 0.000217 | 1.225759 |
| SH3BGRL2 | -2.35954 | 9.400159 | -6.7286 | 2.39E-10 | 3.58E-09 | 13.07122 |
| IGFL4 | -2.36072 | 5.709627 | -4.24165 | 3.60E-05 | 0.000157 | 1.57115 |
| SPDEF | -2.36209 | 3.180792 | -4.47938 | 1.35E-05 | 6.50E-05 | 2.501488 |
| SHTN1 | -2.36229 | 10.62236 | -5.15731 | 6.78E-07 | 4.42E-06 | 5.36859 |
| CBX6 | -2.36567 | 11.6676 | -7.96629 | 2.07E-13 | 6.91E-12 | 19.95824 |
| BCL2L2 | -2.36843 | 10.6243 | -8.05589 | 1.22E-13 | 4.31E-12 | 20.47889 |
| PGPEP1L | -2.37007 | 1.177264 | -5.04345 | 1.14E-06 | 7.05E-06 | 4.865868 |
| RAB15 | -2.37111 | 10.13849 | -7.02779 | 4.60E-11 | 8.25E-10 | 14.6791 |
| SEZ6L2 | -2.37254 | 11.29198 | -4.18566 | 4.51E-05 | 0.000192 | 1.357997 |
| SYTL5 | -2.37295 | 6.719569 | -3.44986 | 0.000704 | 0.002208 | -1.22163 |
| ZNF540 | -2.37544 | 7.349663 | -8.5851 | 5.01E-15 | 2.60E-13 | 23.60487 |
| POU3F1 | -2.37668 | 5.69581 | -3.8777 | 0.000149 | 0.000558 | 0.2274 |
| ACSL6 | -2.37848 | 9.928753 | -4.38163 | 2.03E-05 | 9.39E-05 | 2.114046 |
| TLN2 | -2.37885 | 11.08054 | -7.80123 | 5.49E-13 | 1.60E-11 | 19.00623 |
| FAM174B | -2.38103 | 7.92258 | -8.41424 | 1.42E-14 | 6.33E-13 | 22.58652 |
| NCR3LG1 | -2.38566 | 6.177889 | -5.14223 | 7.27E-07 | 4.70E-06 | 5.301545 |
| SCAMP5 | -2.386 | 12.0916 | -7.26273 | 1.23E-11 | 2.55E-10 | 15.96865 |
| BRSK1 | -2.3867 | 10.88678 | -6.99762 | 5.44E-11 | 9.51E-10 | 14.51519 |
| DEGS2 | -2.38941 | 5.748613 | -5.44461 | 1.75E-07 | 1.32E-06 | 6.673208 |
| PTGDR2 | -2.39039 | 5.500242 | -4.31875 | 2.63E-05 | 0.000118 | 1.868448 |
| BOK | -2.39224 | 8.945546 | -4.20656 | 4.15E-05 | 0.000178 | 1.437312 |
| SPINT2 | -2.39441 | 9.482442 | -6.08246 | 7.34E-09 | 7.61E-08 | 9.742593 |
| AP1M2 | -2.39462 | 1.658811 | -4.37261 | 2.11E-05 | 9.69E-05 | 2.078636 |
| TRPM3 | -2.39509 | 7.873899 | -3.93616 | 0.00012 | 0.000458 | 0.436513 |
| NLRP2 | -2.39744 | 4.948059 | -3.82298 | 0.000184 | 0.000673 | 0.034075 |
| GLS | -2.39833 | 11.06056 | -9.56163 | 1.15E-17 | 1.16E-15 | 29.56691 |
| LMTK3 | -2.39861 | 9.979455 | -5.7481 | 3.98E-08 | 3.44E-07 | 8.104953 |
| NRIP2 | -2.39959 | 7.336636 | -7.92331 | 2.67E-13 | 8.58E-12 | 19.70946 |
| PRKCQ | -2.40168 | 6.929082 | -5.31403 | 3.26E-07 | 2.30E-06 | 6.073932 |
| PCDHGB4 | -2.40254 | 4.306699 | -4.23991 | 3.63E-05 | 0.000158 | 1.564518 |
| MFSD6 | -2.40449 | 10.39078 | -7.81145 | 5.17E-13 | 1.51E-11 | 19.06488 |
| GARNL3 | -2.40674 | 8.697606 | -9.07749 | 2.41E-16 | 1.74E-14 | 26.58315 |
| RNF165 | -2.40851 | 8.604611 | -3.4175 | 0.000787 | 0.002435 | -1.32528 |
| MYO5A | -2.40903 | 11.44917 | -7.4313 | 4.69E-12 | 1.09E-10 | 16.90773 |
| SLC17A8 | -2.40903 | 4.834906 | -2.43618 | 0.015854 | 0.034734 | -4.04906 |
| OR1F1 | -2.41056 | 1.485396 | -4.44706 | 1.55E-05 | 7.34E-05 | 2.372637 |
| ZNF727 | -2.41357 | 3.077721 | -2.81026 | 0.005519 | 0.013777 | -3.10868 |
| CACNA1D | -2.41592 | 7.650918 | -5.33254 | 2.99E-07 | 2.13E-06 | 6.158266 |
| NAP1L6 | -2.41709 | 2.352366 | -3.45366 | 0.000695 | 0.002183 | -1.20939 |
| GPIHBP1 | -2.41786 | 7.23039 | -4.21307 | 4.04E-05 | 0.000173 | 1.462057 |
| MS4A8 | -2.41985 | 1.943977 | -4.5218 | 1.13E-05 | 5.55E-05 | 2.671761 |
| HCN2 | -2.42003 | 9.910908 | -4.03916 | 8.04E-05 | 0.00032 | 0.811273 |
| VAMP2 | -2.42021 | 12.71358 | -8.30966 | 2.67E-14 | 1.12E-12 | 21.96736 |
| SORCS2 | -2.42063 | 9.160786 | -5.03221 | 1.20E-06 | 7.38E-06 | 4.816671 |
| SLC5A7 | -2.42116 | 0.482786 | -7.84697 | 4.19E-13 | 1.26E-11 | 19.26907 |
| RXFP3 | -2.42149 | 0.647036 | -6.63877 | 3.90E-10 | 5.53E-09 | 12.59632 |
| MYH15 | -2.4218 | 5.810717 | -4.44007 | 1.60E-05 | 7.54E-05 | 2.344866 |
| PIP5K1B | -2.422 | 7.384136 | -5.2135 | 5.22E-07 | 3.49E-06 | 5.619725 |
| GFY | -2.423 | 1.274054 | -3.94312 | 0.000117 | 0.000447 | 0.461583 |
| RAPGEF3 | -2.42305 | 9.200999 | -7.42627 | 4.83E-12 | 1.11E-10 | 16.87951 |
| CYFIP2 | -2.42418 | 11.87391 | -6.77425 | 1.87E-10 | 2.87E-09 | 13.31393 |
| IGSF10 | -2.42608 | 5.179727 | -4.88933 | 2.29E-06 | 1.32E-05 | 4.198677 |
| LGI1 | -2.42705 | 8.802096 | -4.0489 | 7.74E-05 | 0.00031 | 0.847115 |
| VIT | -2.42753 | 4.220478 | -2.88129 | 0.004461 | 0.011405 | -2.91626 |
| LINC02210-CRHR1 | -2.42827 | 0.532921 | -7.32164 | 8.78E-12 | 1.89E-10 | 16.29557 |
| CR2 | -2.42939 | 0.946399 | -4.80678 | 3.31E-06 | 1.82E-05 | 3.847756 |
| S1PR5 | -2.42942 | 7.012134 | -3.97501 | 0.000103 | 0.000401 | 0.57693 |
| CDKN2D | -2.42966 | 9.120561 | -8.66691 | 3.04E-15 | 1.65E-13 | 24.09533 |
| SMIM6 | -2.4327 | 3.24442 | -3.58616 | 0.000436 | 0.001446 | -0.77576 |
| LRRC10B | -2.43319 | 5.958481 | -3.56942 | 0.000463 | 0.001521 | -0.83131 |
| PPP3CB | -2.43463 | 10.91939 | -10.2274 | 1.64E-19 | 2.55E-17 | 33.74346 |
| FBXW7 | -2.43529 | 10.16243 | -9.44269 | 2.44E-17 | 2.25E-15 | 28.82931 |
| PLIN1 | -2.43544 | 5.650761 | -5.32271 | 3.13E-07 | 2.22E-06 | 6.113452 |
| NPAS1 | -2.43566 | 6.843405 | -5.20957 | 5.32E-07 | 3.54E-06 | 5.602123 |
| RIPOR2 | -2.43575 | 8.848207 | -5.03534 | 1.19E-06 | 7.29E-06 | 4.830349 |
| EPB41L1 | -2.43893 | 11.81816 | -8.68418 | 2.73E-15 | 1.52E-13 | 24.19907 |
| SLC2A4 | -2.439 | 5.46621 | -5.88865 | 1.97E-08 | 1.83E-07 | 8.785906 |
| C9orf172 | -2.4414 | 8.720419 | -7.41227 | 5.23E-12 | 1.19E-10 | 16.80113 |
| CHRM5 | -2.44147 | 3.805151 | -4.38874 | 1.98E-05 | 9.15E-05 | 2.142015 |
| MOS | -2.44352 | 0.456118 | -7.99409 | 1.76E-13 | 5.96E-12 | 20.1195 |
| HENMT1 | -2.44467 | 7.14547 | -5.62961 | 7.14E-08 | 5.81E-07 | 7.539611 |
| IQSEC2 | -2.44517 | 10.05915 | -7.70021 | 9.91E-13 | 2.73E-11 | 18.42819 |
| SLC2A13 | -2.44527 | 9.557767 | -6.11519 | 6.20E-09 | 6.57E-08 | 9.906133 |
| RUNX1T1 | -2.44614 | 7.609011 | -5.10705 | 8.55E-07 | 5.43E-06 | 5.145657 |
| DBH | -2.44852 | 4.332494 | -4.64733 | 6.63E-06 | 3.39E-05 | 3.182868 |
| KLHL14 | -2.44938 | 4.027515 | -3.32046 | 0.001095 | 0.003274 | -1.63097 |
| B4GALNT3 | -2.45014 | 6.794293 | -4.0774 | 6.93E-05 | 0.00028 | 0.952435 |
| CPEB1 | -2.45103 | 8.443723 | -6.70585 | 2.71E-10 | 3.98E-09 | 12.95059 |
| FLRT2 | -2.45115 | 8.810681 | -4.13435 | 5.53E-05 | 0.000229 | 1.16472 |
| IQCF3 | -2.45126 | 0.310638 | -8.20717 | 4.94E-14 | 1.93E-12 | 21.36377 |
| SFRP2 | -2.45277 | 7.5662 | -2.46225 | 0.014784 | 0.032721 | -3.98757 |
| ASB2 | -2.45349 | 5.300624 | -4.99777 | 1.41E-06 | 8.51E-06 | 4.666487 |
| DOCK9 | -2.45406 | 10.23637 | -7.18288 | 1.93E-11 | 3.80E-10 | 15.52784 |
| PDZD4 | -2.45463 | 11.67848 | -5.62147 | 7.43E-08 | 6.03E-07 | 7.50105 |
| STOX2 | -2.45905 | 9.507602 | -6.41306 | 1.31E-09 | 1.63E-08 | 11.41996 |
| LRP2 | -2.45942 | 7.062024 | -3.04476 | 0.002692 | 0.007312 | -2.4569 |
| PLPPR4 | -2.45969 | 10.48012 | -5.15423 | 6.88E-07 | 4.48E-06 | 5.354915 |
| SCN5A | -2.46122 | 4.725916 | -4.13796 | 5.45E-05 | 0.000226 | 1.178254 |
| PPP1R17 | -2.467 | 4.300203 | -2.8363 | 0.005107 | 0.012851 | -3.03865 |
| ASPG | -2.46811 | 1.660276 | -4.53987 | 1.05E-05 | 5.18E-05 | 2.744639 |
| CCDC166 | -2.46831 | 0.360856 | -10.5147 | 2.56E-20 | 4.84E-18 | 35.56682 |
| RUSC2 | -2.46844 | 10.86046 | -8.103 | 9.20E-14 | 3.37E-12 | 20.75365 |
| OSTN | -2.4697 | 3.84657 | -2.75387 | 0.006517 | 0.015956 | -3.25833 |
| C2CD4D | -2.47054 | 3.52691 | -4.4689 | 1.41E-05 | 6.76E-05 | 2.459633 |
| NSF | -2.47291 | 11.56841 | -11.3722 | 9.50E-23 | 3.53E-20 | 41.06596 |
| SEMA4D | -2.47444 | 10.56045 | -6.60511 | 4.68E-10 | 6.49E-09 | 12.41935 |
| SPRR2G | -2.4748 | 0.236834 | -12.0784 | 9.07E-25 | 4.78E-22 | 45.63846 |
| CYP26C1 | -2.47677 | 2.705131 | -4.871 | 2.49E-06 | 1.42E-05 | 4.12035 |
| EVPL | -2.47746 | 2.531668 | -4.23299 | 3.73E-05 | 0.000161 | 1.538031 |
| PYGM | -2.4799 | 8.305026 | -3.88619 | 0.000145 | 0.000544 | 0.25761 |
| PLEKHH1 | -2.48056 | 10.24556 | -4.7515 | 4.22E-06 | 2.26E-05 | 3.615286 |
| KY | -2.48065 | 4.843116 | -3.31498 | 0.001116 | 0.003328 | -1.64803 |
| CDHR2 | -2.48066 | 5.75744 | -4.85211 | 2.71E-06 | 1.53E-05 | 4.039897 |
| KCNH6 | -2.48112 | 3.01713 | -5.4006 | 2.16E-07 | 1.59E-06 | 6.470076 |
| KCNC4 | -2.48202 | 8.541817 | -7.32674 | 8.53E-12 | 1.84E-10 | 16.32395 |
| RASGEF1C | -2.48356 | 7.663166 | -3.83332 | 0.000177 | 0.000649 | 0.070415 |
| STEAP2 | -2.48404 | 8.156479 | -3.88054 | 0.000148 | 0.000553 | 0.237516 |
| KCNH7 | -2.48409 | 4.294172 | -3.22052 | 0.001528 | 0.004407 | -1.93771 |
| VGF | -2.48598 | 10.35503 | -2.64695 | 0.00887 | 0.020909 | -3.53443 |
| SLC39A12 | -2.48632 | 7.935912 | -3.59172 | 0.000428 | 0.001422 | -0.75724 |
| ANKRD66 | -2.48648 | 1.748428 | -2.95267 | 0.003587 | 0.009431 | -2.71849 |
| CLIC5 | -2.48766 | 5.740612 | -4.99329 | 1.44E-06 | 8.67E-06 | 4.647017 |
| FBXL2 | -2.48825 | 8.417008 | -4.50334 | 1.22E-05 | 5.94E-05 | 2.597487 |
| B4GALT6 | -2.48871 | 8.29295 | -4.48984 | 1.30E-05 | 6.26E-05 | 2.543353 |
| PI4KA | -2.48878 | 11.95922 | -8.7062 | 2.39E-15 | 1.35E-13 | 24.33152 |
| KIF3C | -2.48956 | 11.61841 | -8.00658 | 1.63E-13 | 5.60E-12 | 20.19206 |
| CACNA1A | -2.48965 | 9.169136 | -4.44695 | 1.55E-05 | 7.34E-05 | 2.372207 |
| SLC8A3 | -2.49014 | 7.932182 | -3.19684 | 0.001651 | 0.004719 | -2.00917 |
| KCNMA1 | -2.49039 | 10.43907 | -7.53987 | 2.51E-12 | 6.21E-11 | 17.51832 |
| STK32C | -2.49238 | 9.547278 | -8.97744 | 4.48E-16 | 3.03E-14 | 25.97305 |
| BUB1B-PAK6 | -2.49282 | 0.3787 | -10.0461 | 5.25E-19 | 7.37E-17 | 32.59884 |
| BAIAP3 | -2.49354 | 10.61809 | -3.82244 | 0.000184 | 0.000674 | 0.032185 |
| KRT81 | -2.49427 | 2.261783 | -4.19945 | 4.27E-05 | 0.000182 | 1.410304 |
| C11orf42 | -2.4943 | 2.310295 | -5.20335 | 5.48E-07 | 3.64E-06 | 5.574241 |
| TTLL7 | -2.4945 | 10.3535 | -7.06939 | 3.65E-11 | 6.70E-10 | 14.90574 |
| SORCS3 | -2.49764 | 8.272338 | -3.53124 | 0.00053 | 0.001714 | -0.95721 |
| TSPAN19 | -2.4979 | 2.543423 | -3.44608 | 0.000713 | 0.002233 | -1.23379 |
| PCBP3 | -2.49988 | 7.279434 | -5.42995 | 1.88E-07 | 1.40E-06 | 6.605393 |
| SLC16A8 | -2.50204 | 5.028838 | -5.75103 | 3.92E-08 | 3.40E-07 | 8.119046 |
| COL10A1 | -2.50434 | 3.58952 | -2.9081 | 0.004112 | 0.010626 | -2.84249 |
| ANO5 | -2.50645 | 7.657439 | -3.57768 | 0.000449 | 0.001484 | -0.80392 |
| EGR3 | -2.50961 | 9.337483 | -4.46837 | 1.42E-05 | 6.77E-05 | 2.457509 |
| CSRNP3 | -2.51032 | 9.240883 | -5.57251 | 9.44E-08 | 7.49E-07 | 7.270025 |
| ITPR1 | -2.51092 | 10.00161 | -7.1517 | 2.30E-11 | 4.45E-10 | 15.35638 |
| CABLES1 | -2.51239 | 9.017797 | -6.42579 | 1.22E-09 | 1.53E-08 | 11.48567 |
| LRIT2 | -2.51439 | 2.49829 | -3.96023 | 0.000109 | 0.000421 | 0.523379 |
| GAS7 | -2.51478 | 12.57043 | -5.13419 | 7.55E-07 | 4.85E-06 | 5.265844 |
| SORBS2 | -2.51481 | 9.510611 | -6.00295 | 1.10E-08 | 1.09E-07 | 9.347697 |
| TBC1D26 | -2.5162 | 1.627227 | -4.91213 | 2.07E-06 | 1.20E-05 | 4.296382 |
| NCOA7 | -2.52072 | 9.934848 | -9.20811 | 1.06E-16 | 8.47E-15 | 27.38314 |
| ATP1B4 | -2.52202 | 1.964309 | -4.10114 | 6.31E-05 | 0.000257 | 1.040636 |
| PCDH20 | -2.52551 | 2.214956 | -3.62384 | 0.000381 | 0.001285 | -0.64985 |
| NECTIN1 | -2.52772 | 10.55297 | -5.79103 | 3.21E-08 | 2.84E-07 | 8.311775 |
| UPP2 | -2.52829 | 4.646269 | -4.91381 | 2.06E-06 | 1.20E-05 | 4.303591 |
| GRAMD1B | -2.52847 | 9.839387 | -6.87309 | 1.08E-10 | 1.78E-09 | 13.84275 |
| KCNJ1 | -2.52894 | 2.901862 | -5.48652 | 1.43E-07 | 1.10E-06 | 6.867709 |
| AMZ1 | -2.5301 | 7.022633 | -4.6663 | 6.11E-06 | 3.15E-05 | 3.261062 |
| NYAP1 | -2.53024 | 9.221799 | -6.13729 | 5.54E-09 | 5.92E-08 | 10.01689 |
| DLG4 | -2.53199 | 11.66045 | -9.18479 | 1.23E-16 | 9.50E-15 | 27.24002 |
| CHST1 | -2.53279 | 9.900295 | -5.56851 | 9.62E-08 | 7.61E-07 | 7.25122 |
| KL | -2.53358 | 5.14035 | -5.42356 | 1.94E-07 | 1.44E-06 | 6.575902 |
| CCL28 | -2.53489 | 4.206484 | -4.27193 | 3.19E-05 | 0.00014 | 1.687398 |
| TTC22 | -2.53489 | 2.241694 | -4.96738 | 1.62E-06 | 9.61E-06 | 4.534608 |
| SNPH | -2.53678 | 10.10847 | -7.2751 | 1.14E-11 | 2.39E-10 | 16.03718 |
| NAP1L3 | -2.53744 | 10.39195 | -7.6723 | 1.17E-12 | 3.16E-11 | 18.26914 |
| FGF12 | -2.54212 | 9.487972 | -3.85474 | 0.000163 | 0.000604 | 0.146017 |
| SYT15 | -2.54237 | 4.60822 | -5.55732 | 1.02E-07 | 8.01E-07 | 7.198638 |
| FOXP2 | -2.54326 | 6.446987 | -3.39798 | 0.000842 | 0.002583 | -1.38738 |
| LINGO1 | -2.5447 | 11.17374 | -4.27687 | 3.12E-05 | 0.000138 | 1.706427 |
| 4-Sep | -2.54558 | 11.35587 | -5.2943 | 3.58E-07 | 2.50E-06 | 5.984296 |
| GPR101 | -2.54756 | 0.668905 | -6.04396 | 8.95E-09 | 9.07E-08 | 9.550973 |
| HID1 | -2.54806 | 9.705541 | -6.96187 | 6.64E-11 | 1.15E-09 | 14.32146 |
| IDS | -2.54815 | 13.47451 | -7.48534 | 3.44E-12 | 8.19E-11 | 17.21109 |
| ERBB4 | -2.54909 | 8.902739 | -4.0635 | 7.31E-05 | 0.000295 | 0.900993 |
| UNC79 | -2.54951 | 8.339861 | -5.20191 | 5.51E-07 | 3.66E-06 | 5.56776 |
| SLC6A1 | -2.54979 | 11.41988 | -4.87427 | 2.45E-06 | 1.40E-05 | 4.134291 |
| TSPYL2 | -2.55004 | 11.0075 | -7.56748 | 2.14E-12 | 5.39E-11 | 17.67432 |
| HECW2 | -2.55024 | 8.700474 | -6.77562 | 1.85E-10 | 2.86E-09 | 13.32124 |
| PKD2L1 | -2.5533 | 4.791719 | -4.14843 | 5.23E-05 | 0.000218 | 1.217573 |
| LINC00672 | -2.55487 | 7.784491 | -4.14821 | 5.24E-05 | 0.000218 | 1.216729 |
| TMC5 | -2.55527 | 3.320523 | -5.86855 | 2.18E-08 | 2.01E-07 | 8.687824 |
| C6orf222 | -2.55551 | 0.86894 | -6.65199 | 3.63E-10 | 5.20E-09 | 12.66597 |
| CAMK2N1 | -2.55814 | 12.50301 | -6.41493 | 1.29E-09 | 1.61E-08 | 11.42959 |
| SUSD5 | -2.55867 | 7.342108 | -3.27794 | 0.001263 | 0.003719 | -1.76249 |
| SLC22A2 | -2.56152 | 1.359214 | -4.1235 | 5.78E-05 | 0.000238 | 1.124095 |
| MAP3K21 | -2.56217 | 5.317137 | -5.93771 | 1.54E-08 | 1.46E-07 | 9.026175 |
| PPFIA4 | -2.56347 | 9.529593 | -5.36151 | 2.60E-07 | 1.89E-06 | 6.290634 |
| GABRB1 | -2.56409 | 5.994812 | -3.61488 | 0.000394 | 0.001322 | -0.6799 |
| CCNA1 | -2.56474 | 6.172375 | -3.93977 | 0.000118 | 0.000452 | 0.449531 |
| NHLRC1 | -2.56583 | 5.311241 | -5.35578 | 2.68E-07 | 1.93E-06 | 6.264416 |
| FBXO27 | -2.56589 | 7.637375 | -5.33505 | 2.95E-07 | 2.11E-06 | 6.169695 |
| SIDT1 | -2.56675 | 7.828578 | -5.03247 | 1.20E-06 | 7.37E-06 | 4.817831 |
| SHANK3 | -2.56695 | 10.18765 | -7.03139 | 4.51E-11 | 8.13E-10 | 14.69868 |
| GABARAPL1 | -2.56762 | 11.6465 | -9.31364 | 5.49E-17 | 4.66E-15 | 28.03228 |
| SLC22A15 | -2.56901 | 7.690194 | -5.81905 | 2.79E-08 | 2.51E-07 | 8.447328 |
| TMEM266 | -2.57055 | 5.959105 | -8.22843 | 4.34E-14 | 1.74E-12 | 21.48875 |
| NRXN1 | -2.57298 | 10.7074 | -3.90734 | 0.000134 | 0.000505 | 0.333103 |
| LGI4 | -2.57337 | 9.245967 | -4.31358 | 2.69E-05 | 0.00012 | 1.848362 |
| OXGR1 | -2.5744 | 2.427508 | -4.43221 | 1.65E-05 | 7.77E-05 | 2.313697 |
| ZDHHC11B | -2.57534 | 7.559575 | -3.96205 | 0.000108 | 0.000419 | 0.529971 |
| TMEM200A | -2.57571 | 6.32899 | -3.63739 | 0.000363 | 0.001229 | -0.60433 |
| PAH | -2.57576 | 4.653746 | -3.64545 | 0.000353 | 0.001197 | -0.57717 |
| SYNDIG1L | -2.57647 | 4.407743 | -4.21904 | 3.95E-05 | 0.00017 | 1.484825 |
| AP3B2 | -2.57965 | 10.01705 | -6.1446 | 5.33E-09 | 5.73E-08 | 10.05362 |
| ENPP2 | -2.58026 | 10.30642 | -3.72063 | 0.000268 | 0.000939 | -0.32133 |
| DSCAML1 | -2.58071 | 8.254781 | -3.22031 | 0.001529 | 0.00441 | -1.93834 |
| FGF17 | -2.58138 | 4.967563 | -3.29359 | 0.001199 | 0.003546 | -1.71425 |
| TRIM67 | -2.58165 | 5.966324 | -2.74064 | 0.006774 | 0.016535 | -3.29304 |
| GJC2 | -2.584 | 7.393553 | -4.57924 | 8.87E-06 | 4.44E-05 | 2.904284 |
| CLEC19A | -2.58426 | 0.523756 | -8.49453 | 8.70E-15 | 4.20E-13 | 23.06401 |
| CYP26A1 | -2.58726 | 4.075528 | -3.84677 | 0.000168 | 0.00062 | 0.117846 |
| CACNB2 | -2.58757 | 8.266671 | -5.18411 | 5.99E-07 | 3.96E-06 | 5.488127 |
| SLITRK6 | -2.58944 | 1.489552 | -3.13283 | 0.002033 | 0.005693 | -2.2 |
| PAX2 | -2.59092 | 2.892729 | -2.92519 | 0.003903 | 0.010164 | -2.79515 |
| CPAMD8 | -2.59106 | 5.809644 | -3.94188 | 0.000117 | 0.000449 | 0.457115 |
| RASD2 | -2.59302 | 8.411303 | -5.82564 | 2.70E-08 | 2.43E-07 | 8.47925 |
| AQP3 | -2.59313 | 6.400921 | -5.07593 | 9.86E-07 | 6.18E-06 | 5.008432 |
| CACNB4 | -2.59495 | 9.038678 | -7.40693 | 5.40E-12 | 1.22E-10 | 16.77128 |
| FAM133A | -2.59502 | 5.390109 | -4.09827 | 6.38E-05 | 0.00026 | 1.029934 |
| NRAP | -2.59545 | 4.170503 | -4.33047 | 2.51E-05 | 0.000113 | 1.913989 |
| AKAIN1 | -2.59569 | 1.207752 | -5.05166 | 1.10E-06 | 6.82E-06 | 4.901813 |
| PLEKHM3 | -2.59833 | 6.377123 | -6.61692 | 4.39E-10 | 6.13E-09 | 12.48136 |
| DAB1 | -2.59968 | 7.444364 | -3.95924 | 0.00011 | 0.000423 | 0.519785 |
| ABCA2 | -2.60047 | 12.72383 | -5.97262 | 1.29E-08 | 1.26E-07 | 9.197905 |
| CLUL1 | -2.60784 | 5.330768 | -3.60247 | 0.000411 | 0.001376 | -0.7214 |
| PHACTR1 | -2.60915 | 9.896387 | -6.22509 | 3.51E-09 | 3.96E-08 | 10.45943 |
| LYPD6B | -2.6099 | 3.542502 | -3.40839 | 0.000812 | 0.002502 | -1.3543 |
| KRT86 | -2.61346 | 3.542911 | -4.54526 | 1.03E-05 | 5.07E-05 | 2.766453 |
| NDST4 | -2.6149 | 1.83958 | -3.2318 | 0.001472 | 0.004269 | -1.90352 |
| PDE11A | -2.61815 | 4.631999 | -4.7538 | 4.17E-06 | 2.24E-05 | 3.624922 |
| MAPK8IP3 | -2.61973 | 11.95449 | -6.81397 | 1.50E-10 | 2.37E-09 | 13.52591 |
| PRAP1 | -2.61995 | 2.066849 | -5.94175 | 1.51E-08 | 1.44E-07 | 9.045973 |
| WNT8A | -2.62128 | 1.129055 | -6.42427 | 1.23E-09 | 1.54E-08 | 11.47783 |
| KIAA1211L | -2.6215 | 9.252762 | -6.14129 | 5.42E-09 | 5.81E-08 | 10.03697 |
| PGR | -2.62215 | 3.349311 | -4.5605 | 9.61E-06 | 4.78E-05 | 2.828173 |
| ASPA | -2.62315 | 8.082129 | -3.9771 | 0.000102 | 0.000398 | 0.584495 |
| TMEM56 | -2.62411 | 8.276362 | -5.47649 | 1.50E-07 | 1.14E-06 | 6.821039 |
| ARRB1 | -2.62689 | 9.379714 | -7.56796 | 2.14E-12 | 5.38E-11 | 17.67708 |
| FAM237A | -2.62768 | 1.880948 | -5.17679 | 6.20E-07 | 4.07E-06 | 5.455452 |
| BSPRY | -2.62998 | 4.403946 | -4.72772 | 4.68E-06 | 2.48E-05 | 3.515949 |
| MDGA2 | -2.63192 | 5.631732 | -2.91418 | 0.004036 | 0.010461 | -2.82569 |
| PCDHGA12 | -2.63616 | 6.539067 | -3.29724 | 0.001184 | 0.003507 | -1.70297 |
| SYT14 | -2.63628 | 7.009364 | -4.48641 | 1.31E-05 | 6.33E-05 | 2.52962 |
| FXYD1 | -2.64284 | 6.602053 | -3.74756 | 0.000243 | 0.000863 | -0.2286 |
| RTL4 | -2.64331 | 0.750929 | -6.33275 | 2.00E-09 | 2.39E-08 | 11.00741 |
| TMEM63C | -2.64554 | 8.377079 | -5.65555 | 6.29E-08 | 5.18E-07 | 7.66268 |
| UTS2R | -2.6466 | 1.596847 | -4.54779 | 1.01E-05 | 5.02E-05 | 2.776657 |
| LOR | -2.6473 | 0.907448 | -6.71533 | 2.57E-10 | 3.82E-09 | 13.00078 |
| ADRA2A | -2.64989 | 6.279225 | -3.91143 | 0.000131 | 0.000498 | 0.347753 |
| CEND1 | -2.65085 | 10.54095 | -5.77692 | 3.45E-08 | 3.03E-07 | 8.243691 |
| BARX2 | -2.65495 | 3.179028 | -4.28107 | 3.07E-05 | 0.000136 | 1.72263 |
| MEPE | -2.65647 | 4.357806 | -2.9576 | 0.003533 | 0.009305 | -2.70467 |
| KRT83 | -2.65731 | 1.784382 | -4.00133 | 9.31E-05 | 0.000365 | 0.672684 |
| SEMA6B | -2.66258 | 10.16184 | -5.4359 | 1.83E-07 | 1.37E-06 | 6.632911 |
| ABCC8 | -2.66272 | 7.669189 | -3.52038 | 0.000551 | 0.001773 | -0.99281 |
| AC138647.1 | -2.66494 | 0.410353 | -8.46956 | 1.01E-14 | 4.73E-13 | 22.91529 |
| PLCL1 | -2.66521 | 8.439326 | -5.72777 | 4.40E-08 | 3.76E-07 | 8.007398 |
| SLC25A48 | -2.66685 | 6.745506 | -3.25668 | 0.001356 | 0.003967 | -1.82769 |
| HRK | -2.66786 | 7.579506 | -4.55185 | 9.97E-06 | 4.95E-05 | 2.79312 |
| ZNF483 | -2.66834 | 6.259374 | -7.01993 | 4.81E-11 | 8.57E-10 | 14.63637 |
| CDHR1 | -2.66952 | 8.241095 | -4.20123 | 4.24E-05 | 0.000181 | 1.41705 |
| LAMP5 | -2.67176 | 9.347117 | -4.42483 | 1.70E-05 | 7.98E-05 | 2.284464 |
| PPL | -2.67767 | 8.19721 | -3.81457 | 0.000189 | 0.000692 | 0.004574 |
| GOT1 | -2.67948 | 10.63763 | -10.1375 | 2.92E-19 | 4.32E-17 | 33.17523 |
| CACNA1F | -2.68118 | 4.117514 | -7.0968 | 3.13E-11 | 5.85E-10 | 15.05549 |
| DGKB | -2.68291 | 8.928069 | -4.16359 | 4.93E-05 | 0.000207 | 1.274626 |
| GREM1 | -2.68359 | 6.574669 | -3.09722 | 0.002279 | 0.006299 | -2.30465 |
| CDH13 | -2.68449 | 9.650769 | -3.95647 | 0.000111 | 0.000427 | 0.509762 |
| SMPD3 | -2.68722 | 7.886814 | -4.57997 | 8.85E-06 | 4.43E-05 | 2.907261 |
| CEP126 | -2.68768 | 7.64596 | -4.00146 | 9.31E-05 | 0.000365 | 0.673176 |
| APLP1 | -2.6877 | 13.09192 | -5.99229 | 1.17E-08 | 1.15E-07 | 9.294962 |
| GLB1L3 | -2.68855 | 6.175656 | -3.89197 | 0.000142 | 0.000533 | 0.278215 |
| RIPPLY2 | -2.68953 | 5.371853 | -3.39057 | 0.000864 | 0.002643 | -1.41088 |
| GRIA2 | -2.69003 | 10.86656 | -4.00804 | 9.08E-05 | 0.000357 | 0.697188 |
| TDRD9 | -2.69504 | 5.454865 | -5.49901 | 1.35E-07 | 1.04E-06 | 6.92588 |
| FOSB | -2.69589 | 8.71737 | -3.16474 | 0.001833 | 0.005182 | -2.1053 |
| MMP17 | -2.69618 | 8.328112 | -4.02209 | 8.59E-05 | 0.00034 | 0.748623 |
| CLSTN3 | -2.6967 | 11.41149 | -8.94491 | 5.48E-16 | 3.60E-14 | 25.77522 |
| RASL10A | -2.69825 | 7.562874 | -5.35019 | 2.75E-07 | 1.97E-06 | 6.238864 |
| KCNIP4 | -2.69919 | 8.550908 | -4.37279 | 2.11E-05 | 9.69E-05 | 2.079367 |
| DYDC2 | -2.70033 | 3.633283 | -3.1283 | 0.002063 | 0.005767 | -2.21335 |
| FLRT1 | -2.70263 | 7.765504 | -4.16138 | 4.97E-05 | 0.000209 | 1.266285 |
| CABYR | -2.70938 | 6.341223 | -6.72754 | 2.41E-10 | 3.60E-09 | 13.06556 |
| ATL1 | -2.70974 | 9.659315 | -9.56684 | 1.11E-17 | 1.13E-15 | 29.59934 |
| PAIP2B | -2.70986 | 8.449009 | -4.69429 | 5.41E-06 | 2.82E-05 | 3.376881 |
| DSG1 | -2.7112 | 0.901522 | -6.39629 | 1.43E-09 | 1.76E-08 | 11.33353 |
| ATP2C2 | -2.71466 | 5.216916 | -5.57392 | 9.37E-08 | 7.44E-07 | 7.276653 |
| TMCC2 | -2.7157 | 9.530665 | -6.4301 | 1.19E-09 | 1.50E-08 | 11.50791 |
| NBEA | -2.71599 | 9.536622 | -7.11818 | 2.77E-11 | 5.24E-10 | 15.17253 |
| PKP2 | -2.71889 | 5.169235 | -3.24112 | 0.001427 | 0.004151 | -1.87515 |
| LRRC38 | -2.72347 | 2.627793 | -3.88557 | 0.000145 | 0.000545 | 0.255416 |
| MAST1 | -2.72709 | 9.48199 | -4.65167 | 6.51E-06 | 3.34E-05 | 3.200753 |
| HCN4 | -2.72711 | 5.313521 | -4.23757 | 3.66E-05 | 0.000159 | 1.555571 |
| KLK6 | -2.72718 | 8.272161 | -2.64227 | 0.008988 | 0.021158 | -3.5463 |
| DUSP2 | -2.72789 | 6.626853 | -4.71524 | 4.94E-06 | 2.60E-05 | 3.463955 |
| CD164L2 | -2.7285 | 1.087125 | -5.75008 | 3.94E-08 | 3.41E-07 | 8.114493 |
| C10orf142 | -2.72992 | 1.653022 | -5.41611 | 2.01E-07 | 1.49E-06 | 6.541529 |
| MYRF | -2.73007 | 10.37384 | -3.41546 | 0.000793 | 0.002449 | -1.33177 |
| ROS1 | -2.73036 | 2.674353 | -3.04602 | 0.002681 | 0.007287 | -2.45328 |
| PDXP | -2.73409 | 6.681132 | -6.67501 | 3.20E-10 | 4.65E-09 | 12.78744 |
| UNC80 | -2.73545 | 9.261065 | -5.72243 | 4.52E-08 | 3.85E-07 | 7.981787 |
| ZNF488 | -2.73738 | 8.01804 | -2.90794 | 0.004114 | 0.010626 | -2.84294 |
| TEF | -2.74002 | 10.02011 | -8.08855 | 1.00E-13 | 3.64E-12 | 20.66932 |
| KCNA2 | -2.74005 | 7.499616 | -5.70798 | 4.86E-08 | 4.11E-07 | 7.912648 |
| TRPM6 | -2.74085 | 5.190144 | -5.45086 | 1.70E-07 | 1.28E-06 | 6.702104 |
| TMEM244 | -2.74134 | 0.822247 | -5.98005 | 1.24E-08 | 1.22E-07 | 9.234562 |
| SCUBE1 | -2.74234 | 4.034014 | -4.21347 | 4.04E-05 | 0.000173 | 1.463581 |
| RAPGEFL1 | -2.74328 | 9.200522 | -8.28149 | 3.16E-14 | 1.30E-12 | 21.80114 |
| RAB26 | -2.74704 | 7.695361 | -5.78244 | 3.35E-08 | 2.96E-07 | 8.270338 |
| NPFFR2 | -2.74801 | 0.889485 | -5.51884 | 1.22E-07 | 9.51E-07 | 7.018413 |
| ADRB1 | -2.74803 | 6.807126 | -5.39677 | 2.20E-07 | 1.62E-06 | 6.452461 |
| GABBR1 | -2.7481 | 12.15273 | -6.15041 | 5.17E-09 | 5.58E-08 | 10.08277 |
| RNF212 | -2.75084 | 4.295763 | -3.45428 | 0.000693 | 0.00218 | -1.2074 |
| SMIM22 | -2.7511 | 2.274653 | -4.9097 | 2.09E-06 | 1.22E-05 | 4.285948 |
| PDE4C | -2.75124 | 6.224323 | -4.69242 | 5.45E-06 | 2.84E-05 | 3.369148 |
| EFCAB1 | -2.75178 | 4.932962 | -2.78582 | 0.005933 | 0.014689 | -3.1739 |
| CRTAC1 | -2.75308 | 7.974094 | -3.86309 | 0.000158 | 0.000587 | 0.175574 |
| MAP7 | -2.75331 | 9.405894 | -6.16687 | 4.75E-09 | 5.17E-08 | 10.16554 |
| ZNF208 | -2.75695 | 2.973178 | -3.61184 | 0.000398 | 0.001335 | -0.69007 |
| DISP2 | -2.75954 | 8.876413 | -5.53433 | 1.14E-07 | 8.88E-07 | 7.090836 |
| SYT12 | -2.75998 | 8.643415 | -5.26033 | 4.20E-07 | 2.87E-06 | 5.830545 |
| ZFP57 | -2.76173 | 3.807448 | -3.27282 | 0.001285 | 0.003777 | -1.77822 |
| MCTP1 | -2.76386 | 8.13244 | -6.58735 | 5.15E-10 | 7.06E-09 | 12.32617 |
| HTR1B | -2.76473 | 2.538169 | -5.04211 | 1.15E-06 | 7.09E-06 | 4.859977 |
| GREB1L | -2.77064 | 5.248475 | -3.52404 | 0.000543 | 0.001753 | -0.98081 |
| CADM3 | -2.77083 | 11.41366 | -3.35114 | 0.000988 | 0.002986 | -1.53515 |
| PRKAR1B | -2.7724 | 11.81058 | -8.31768 | 2.54E-14 | 1.07E-12 | 22.01476 |
| SYNJ2 | -2.77309 | 9.801211 | -6.0092 | 1.07E-08 | 1.06E-07 | 9.378611 |
| DNAJA4 | -2.77344 | 9.747707 | -7.56337 | 2.19E-12 | 5.49E-11 | 17.65112 |
| ZNF728 | -2.77876 | 1.44893 | -3.80187 | 0.000199 | 0.000722 | -0.03989 |
| NKX6-3 | -2.78148 | 0.889855 | -6.67647 | 3.18E-10 | 4.62E-09 | 12.79519 |
| KCNA5 | -2.78169 | 4.700763 | -3.70872 | 0.00028 | 0.000977 | -0.36216 |
| MSANTD1 | -2.78311 | 4.953862 | -7.98863 | 1.82E-13 | 6.12E-12 | 20.08782 |
| KLHL3 | -2.78313 | 7.861809 | -7.75451 | 7.21E-13 | 2.05E-11 | 18.73846 |
| MGAT5B | -2.78646 | 9.201308 | -4.99491 | 1.43E-06 | 8.61E-06 | 4.654061 |
| PANX2 | -2.78681 | 7.623298 | -6.78729 | 1.74E-10 | 2.70E-09 | 13.38348 |
| KALRN | -2.78719 | 10.20616 | -8.50425 | 8.20E-15 | 4.02E-13 | 23.12194 |
| PLCXD2 | -2.78723 | 3.411851 | -3.99333 | 9.61E-05 | 0.000375 | 0.643527 |
| FAAH | -2.79069 | 8.450526 | -6.26859 | 2.80E-09 | 3.22E-08 | 10.68012 |
| PAK1 | -2.79127 | 11.10962 | -10.1087 | 3.51E-19 | 5.12E-17 | 32.99352 |
| GNAO1 | -2.79348 | 12.07742 | -5.5763 | 9.27E-08 | 7.36E-07 | 7.287885 |
| INPP5F | -2.7938 | 10.43659 | -7.94144 | 2.40E-13 | 7.77E-12 | 19.8143 |
| FCHO1 | -2.79401 | 8.325241 | -7.32205 | 8.76E-12 | 1.88E-10 | 16.29781 |
| FO681492.1 | -2.79467 | 4.83512 | -4.83423 | 2.93E-06 | 1.64E-05 | 3.963917 |
| 5-Sep | -2.79885 | 11.60242 | -8.03496 | 1.38E-13 | 4.82E-12 | 20.35701 |
| F7 | -2.79899 | 2.909053 | -3.87083 | 0.000153 | 0.000572 | 0.203024 |
| KRT3 | -2.80492 | 0.522874 | -7.0419 | 4.25E-11 | 7.71E-10 | 14.75589 |
| PSD3 | -2.8057 | 11.33611 | -8.83183 | 1.10E-15 | 6.80E-14 | 25.0895 |
| CA4 | -2.8063 | 7.183334 | -4.50498 | 1.22E-05 | 5.91E-05 | 2.604107 |
| NTNG2 | -2.80729 | 8.74971 | -5.61348 | 7.73E-08 | 6.24E-07 | 7.463241 |
| PRR18 | -2.80733 | 8.445317 | -5.55688 | 1.02E-07 | 8.02E-07 | 7.196587 |
| INPP5J | -2.80907 | 7.115857 | -7.04374 | 4.21E-11 | 7.64E-10 | 14.76594 |
| BMP3 | -2.81148 | 1.596359 | -3.91366 | 0.00013 | 0.000495 | 0.355725 |
| EHD3 | -2.81275 | 10.32003 | -7.93608 | 2.48E-13 | 8.01E-12 | 19.78332 |
| BRSK2 | -2.81571 | 9.589189 | -4.4084 | 1.82E-05 | 8.47E-05 | 2.219485 |
| SFRP1 | -2.81597 | 7.743056 | -2.98017 | 0.003294 | 0.008752 | -2.64112 |
| TRIM54 | -2.81605 | 4.078666 | -4.69643 | 5.36E-06 | 2.80E-05 | 3.385794 |
| APBA1 | -2.8162 | 9.868474 | -7.20149 | 1.74E-11 | 3.49E-10 | 15.63032 |
| FBLL1 | -2.81839 | 7.513492 | -3.79014 | 0.000207 | 0.00075 | -0.08086 |
| TRIML2 | -2.81917 | 1.484054 | -4.92679 | 1.94E-06 | 1.13E-05 | 4.359408 |
| CLCN4 | -2.82022 | 10.22871 | -8.75828 | 1.73E-15 | 1.02E-13 | 24.64527 |
| OVOL2 | -2.82163 | 1.14537 | -5.84785 | 2.42E-08 | 2.20E-07 | 8.587098 |
| CYP2E1 | -2.82216 | 5.556542 | -5.35425 | 2.69E-07 | 1.94E-06 | 6.257402 |
| HAPLN2 | -2.82366 | 8.535246 | -3.35717 | 0.000968 | 0.002931 | -1.51624 |
| VWC2 | -2.8254 | 6.522185 | -3.11146 | 0.002177 | 0.006047 | -2.26293 |
| SMIM17 | -2.82543 | 5.272665 | -6.62635 | 4.17E-10 | 5.86E-09 | 12.53094 |
| SLIT2 | -2.82552 | 7.669365 | -4.09569 | 6.45E-05 | 0.000262 | 1.020337 |
| TRABD2A | -2.82883 | 5.58628 | -5.89799 | 1.88E-08 | 1.75E-07 | 8.831558 |
| LYNX1 | -2.82907 | 10.88322 | -6.87138 | 1.09E-10 | 1.79E-09 | 13.83356 |
| CSMD1 | -2.82936 | 8.255632 | -4.83403 | 2.93E-06 | 1.64E-05 | 3.963101 |
| PLCH1 | -2.8308 | 7.507223 | -5.59334 | 8.53E-08 | 6.84E-07 | 7.368161 |
| PRB2 | -2.83234 | 0.805663 | -7.84036 | 4.36E-13 | 1.31E-11 | 19.23107 |
| SGCZ | -2.83377 | 2.105277 | -3.39238 | 0.000858 | 0.002628 | -1.40515 |
| SYT2 | -2.83403 | 5.199713 | -4.8968 | 2.22E-06 | 1.28E-05 | 4.230645 |
| RAB40B | -2.83457 | 9.656807 | -8.21132 | 4.81E-14 | 1.89E-12 | 21.38815 |
| KCNQ2 | -2.83475 | 11.33763 | -3.5045 | 0.000582 | 0.001862 | -1.04467 |
| CACNA2D2 | -2.83535 | 7.169565 | -4.87485 | 2.45E-06 | 1.40E-05 | 4.136798 |
| BASP1 | -2.83545 | 11.14376 | -5.09301 | 9.12E-07 | 5.75E-06 | 5.083673 |
| GCNT4 | -2.83821 | 5.667856 | -5.44801 | 1.72E-07 | 1.30E-06 | 6.688931 |
| CDS1 | -2.83851 | 7.77196 | -5.5682 | 9.64E-08 | 7.62E-07 | 7.249769 |
| PKP1 | -2.84175 | 4.321231 | -3.81881 | 0.000186 | 0.000682 | 0.01944 |
| SHISA9 | -2.84245 | 8.42033 | -3.95329 | 0.000112 | 0.000432 | 0.498285 |
| KCNC3 | -2.8453 | 8.592969 | -6.83698 | 1.32E-10 | 2.11E-09 | 13.64904 |
| CBX7 | -2.84644 | 9.915589 | -8.43822 | 1.23E-14 | 5.60E-13 | 22.72895 |
| EPB41L3 | -2.84739 | 10.64822 | -5.7761 | 3.46E-08 | 3.04E-07 | 8.239768 |
| PCDHGC5 | -2.84764 | 9.014979 | -5.55434 | 1.03E-07 | 8.12E-07 | 7.184657 |
| SLC5A11 | -2.84952 | 6.418006 | -3.41846 | 0.000785 | 0.002427 | -1.32222 |
| GFRA2 | -2.85086 | 7.602041 | -4.39344 | 1.94E-05 | 8.99E-05 | 2.160522 |
| SP7 | -2.851 | 2.340183 | -5.22903 | 4.86E-07 | 3.27E-06 | 5.689502 |
| BEGAIN | -2.85132 | 8.223216 | -5.38503 | 2.33E-07 | 1.70E-06 | 6.398499 |
| UNC5C | -2.85304 | 8.062596 | -4.29907 | 2.85E-05 | 0.000127 | 1.792147 |
| RESP18 | -2.85344 | 1.476681 | -4.65513 | 6.41E-06 | 3.29E-05 | 3.215003 |
| NUAK1 | -2.85367 | 10.0347 | -9.33384 | 4.84E-17 | 4.17E-15 | 28.15678 |
| SHISA8 | -2.85392 | 3.56228 | -5.66687 | 5.95E-08 | 4.93E-07 | 7.716541 |
| SELE | -2.85607 | 3.714107 | -3.61373 | 0.000395 | 0.001327 | -0.68374 |
| ACBD7 | -2.86141 | 7.058746 | -4.79123 | 3.54E-06 | 1.94E-05 | 3.782125 |
| MOG | -2.86288 | 9.661766 | -2.75259 | 0.006542 | 0.016006 | -3.26171 |
| RNF43 | -2.86341 | 4.778334 | -4.17038 | 4.80E-05 | 0.000202 | 1.30023 |
| DUSP8 | -2.8665 | 9.391517 | -6.23839 | 3.27E-09 | 3.71E-08 | 10.52681 |
| NT5C1A | -2.86897 | 3.066133 | -3.70318 | 0.000286 | 0.000995 | -0.38109 |
| NTSR2 | -2.87416 | 7.695497 | -3.28633 | 0.001228 | 0.003626 | -1.73665 |
| PGBD5 | -2.87558 | 9.966357 | -7.4451 | 4.34E-12 | 1.01E-10 | 16.98509 |
| GALNT14 | -2.87605 | 6.510534 | -5.36576 | 2.55E-07 | 1.85E-06 | 6.310084 |
| THRB | -2.8795 | 9.195222 | -6.97994 | 6.00E-11 | 1.04E-09 | 14.41932 |
| LRRTM4 | -2.8804 | 7.216872 | -3.56801 | 0.000465 | 0.001528 | -0.83599 |
| KIF19 | -2.8827 | 6.31179 | -4.15671 | 5.06E-05 | 0.000212 | 1.248701 |
| LYZL4 | -2.88333 | 0.946047 | -6.4869 | 8.82E-10 | 1.15E-08 | 11.80209 |
| CLDN16 | -2.88428 | 1.689137 | -6.24064 | 3.24E-09 | 3.67E-08 | 10.53818 |
| ELAVL4 | -2.89052 | 8.745525 | -3.61542 | 0.000393 | 0.00132 | -0.6781 |
| DCLK3 | -2.89118 | 4.807253 | -5.16782 | 6.46E-07 | 4.24E-06 | 5.41542 |
| SYT3 | -2.8919 | 7.676224 | -5.48347 | 1.45E-07 | 1.11E-06 | 6.853489 |
| MICU3 | -2.8962 | 8.304695 | -9.77456 | 2.98E-18 | 3.51E-16 | 30.8943 |
| CACNA2D1 | -2.90083 | 9.275431 | -4.4639 | 1.44E-05 | 6.89E-05 | 2.439696 |
| TSPYL5 | -2.90363 | 8.439755 | -5.72102 | 4.55E-08 | 3.88E-07 | 7.975051 |
| IL34 | -2.90435 | 7.775195 | -7.0005 | 5.36E-11 | 9.37E-10 | 14.53084 |
| FBXO41 | -2.90462 | 9.908157 | -7.15891 | 2.21E-11 | 4.30E-10 | 15.39599 |
| MGAT3 | -2.90596 | 9.994064 | -6.1543 | 5.07E-09 | 5.48E-08 | 10.10232 |
| SLC26A4 | -2.90716 | 5.848363 | -5.90515 | 1.81E-08 | 1.70E-07 | 8.86654 |
| KIAA1549L | -2.90938 | 10.01132 | -6.96956 | 6.36E-11 | 1.10E-09 | 14.36308 |
| SERPINA10 | -2.91119 | 0.869935 | -7.42835 | 4.77E-12 | 1.10E-10 | 16.89116 |
| CACNA1C | -2.91244 | 8.728435 | -5.89667 | 1.89E-08 | 1.76E-07 | 8.825065 |
| USP43 | -2.91312 | 6.285647 | -3.88412 | 0.000146 | 0.000547 | 0.250267 |
| SPPL2C | -2.91969 | 1.344366 | -6.07882 | 7.48E-09 | 7.72E-08 | 9.724436 |
| SLITRK5 | -2.9205 | 8.138551 | -4.85055 | 2.73E-06 | 1.54E-05 | 4.033255 |
| CACNB1 | -2.92153 | 9.630501 | -8.49386 | 8.74E-15 | 4.20E-13 | 23.06002 |
| PENK | -2.92355 | 5.874851 | -3.30545 | 0.001152 | 0.003425 | -1.67758 |
| TLL2 | -2.92402 | 5.748935 | -5.52289 | 1.20E-07 | 9.35E-07 | 7.037319 |
| RAP1GAP | -2.92586 | 10.76385 | -5.80358 | 3.02E-08 | 2.69E-07 | 8.372455 |
| RBP3 | -2.92729 | 2.391982 | -3.59866 | 0.000417 | 0.001392 | -0.73413 |
| RAPGEF5 | -2.92916 | 10.116 | -6.49949 | 8.24E-10 | 1.08E-08 | 11.86752 |
| KCNK3 | -2.93092 | 7.329511 | -5.10031 | 8.82E-07 | 5.58E-06 | 5.11589 |
| SLC24A4 | -2.93162 | 7.691116 | -4.23784 | 3.66E-05 | 0.000159 | 1.556587 |
| PARM1 | -2.93305 | 9.052525 | -4.9165 | 2.03E-06 | 1.18E-05 | 4.315151 |
| SLCO4C1 | -2.93422 | 1.6982 | -5.78738 | 3.27E-08 | 2.89E-07 | 8.294187 |
| GPR27 | -2.93425 | 7.339124 | -3.93657 | 0.000119 | 0.000457 | 0.437983 |
| SPTBN4 | -2.93919 | 9.461316 | -7.32685 | 8.53E-12 | 1.84E-10 | 16.32452 |
| ADAMTS19 | -2.94113 | 3.66103 | -3.50924 | 0.000572 | 0.001834 | -1.02923 |
| GLP1R | -2.9412 | 2.304855 | -4.04359 | 7.90E-05 | 0.000315 | 0.82756 |
| ITPKA | -2.94167 | 8.075611 | -6.86837 | 1.11E-10 | 1.81E-09 | 13.8174 |
| MTURN | -2.94528 | 12.7626 | -5.93021 | 1.60E-08 | 1.52E-07 | 8.989335 |
| CYGB | -2.95166 | 7.407685 | -5.13692 | 7.45E-07 | 4.80E-06 | 5.277946 |
| EPHA5 | -2.95338 | 7.924127 | -4.75103 | 4.23E-06 | 2.26E-05 | 3.613324 |
| GPRIN2 | -2.9535 | 1.313627 | -4.71972 | 4.84E-06 | 2.56E-05 | 3.482604 |
| KLHL32 | -2.95371 | 8.026491 | -4.47835 | 1.36E-05 | 6.53E-05 | 2.497371 |
| LPA | -2.95425 | 1.817183 | -5.38509 | 2.33E-07 | 1.70E-06 | 6.398756 |
| ASPHD1 | -2.95551 | 9.154981 | -6.32599 | 2.07E-09 | 2.46E-08 | 10.97284 |
| CAMK1D | -2.95839 | 10.59647 | -9.68965 | 5.11E-18 | 5.58E-16 | 30.36394 |
| ST18 | -2.95929 | 7.36406 | -3.73744 | 0.000252 | 0.000892 | -0.26353 |
| ATP1B1 | -2.96173 | 12.23466 | -6.4644 | 9.94E-10 | 1.27E-08 | 11.6854 |
| EPN3 | -2.96242 | 3.902415 | -5.37401 | 2.45E-07 | 1.79E-06 | 6.34793 |
| NCS1 | -2.96245 | 12.08652 | -8.20795 | 4.91E-14 | 1.92E-12 | 21.36839 |
| ABLIM2 | -2.96302 | 8.516574 | -8.07429 | 1.09E-13 | 3.90E-12 | 20.58614 |
| CNKSR2 | -2.96587 | 9.25656 | -7.75267 | 7.29E-13 | 2.06E-11 | 18.72792 |
| ADTRP | -2.96705 | 3.980617 | -4.81541 | 3.18E-06 | 1.76E-05 | 3.884197 |
| PNLDC1 | -2.96717 | 3.188116 | -5.80999 | 2.92E-08 | 2.61E-07 | 8.403473 |
| B3GALT2 | -2.96771 | 7.849378 | -4.616 | 7.58E-06 | 3.85E-05 | 3.054291 |
| FXYD4 | -2.96877 | 1.555849 | -4.64001 | 6.84E-06 | 3.49E-05 | 3.152784 |
| RTP1 | -2.96945 | 3.327878 | -3.62548 | 0.000379 | 0.001278 | -0.64435 |
| WNT2 | -2.96966 | 4.31293 | -3.47885 | 0.000637 | 0.00202 | -1.12804 |
| GFRA1 | -2.98072 | 7.335044 | -3.43087 | 0.000752 | 0.002337 | -1.28255 |
| KIAA1324 | -2.98089 | 7.895112 | -6.92214 | 8.27E-11 | 1.40E-09 | 14.10683 |
| SLC5A5 | -2.98656 | 2.431734 | -5.69539 | 5.17E-08 | 4.35E-07 | 7.852464 |
| POPDC3 | -2.99145 | 6.057573 | -4.27362 | 3.17E-05 | 0.000139 | 1.693898 |
| PTPN20 | -2.99169 | 3.924913 | -4.1012 | 6.31E-05 | 0.000257 | 1.040846 |
| CLPSL1 | -2.99196 | 0.520368 | -9.8209 | 2.22E-18 | 2.69E-16 | 31.18424 |
| LRRC73 | -2.99504 | 6.824687 | -7.35135 | 7.41E-12 | 1.63E-10 | 16.46095 |
| CES5A | -2.99675 | 2.105473 | -5.56175 | 9.95E-08 | 7.85E-07 | 7.21944 |
| ADAMTS16 | -2.99713 | 5.021432 | -4.83927 | 2.86E-06 | 1.61E-05 | 3.985296 |
| YPEL4 | -2.99948 | 7.881697 | -7.52834 | 2.69E-12 | 6.57E-11 | 17.45328 |
| KCNIP2 | -3.00119 | 8.310658 | -4.97741 | 1.54E-06 | 9.23E-06 | 4.578057 |
| DUSP26 | -3.00527 | 8.64651 | -4.59523 | 8.29E-06 | 4.17E-05 | 2.969415 |
| NUPR2 | -3.00662 | 2.282491 | -4.46107 | 1.46E-05 | 6.97E-05 | 2.428429 |
| CYP2C18 | -3.00854 | 0.4344 | -11.0941 | 5.88E-22 | 1.78E-19 | 39.27472 |
| PPP1R1B | -3.01334 | 9.912251 | -3.47899 | 0.000636 | 0.002019 | -1.12758 |
| ADCYAP1 | -3.01469 | 5.8961 | -4.49893 | 1.25E-05 | 6.04E-05 | 2.579792 |
| HS3ST5 | -3.01628 | 5.406408 | -4.93346 | 1.88E-06 | 1.10E-05 | 4.388138 |
| RHOV | -3.01637 | 3.669541 | -6.58749 | 5.14E-10 | 7.06E-09 | 12.32692 |
| FER1L6 | -3.01747 | 2.171106 | -5.64389 | 6.66E-08 | 5.46E-07 | 7.607288 |
| COL19A1 | -3.02109 | 3.566361 | -3.90222 | 0.000136 | 0.000515 | 0.314791 |
| SPX | -3.02274 | 6.943822 | -3.66877 | 0.000324 | 0.001111 | -0.49828 |
| PCDHGA3 | -3.02338 | 5.622922 | -4.73813 | 4.47E-06 | 2.38E-05 | 3.559384 |
| SFTPC | -3.02587 | 4.648349 | -4.75928 | 4.08E-06 | 2.19E-05 | 3.647886 |
| FAM189A1 | -3.02758 | 7.955449 | -5.80266 | 3.03E-08 | 2.70E-07 | 8.367983 |
| GJD4 | -3.02902 | 1.704852 | -5.83524 | 2.58E-08 | 2.33E-07 | 8.525836 |
| SMIM10L2A | -3.02917 | 8.951864 | -7.08736 | 3.30E-11 | 6.14E-10 | 15.0039 |
| PLIN4 | -3.02975 | 6.065232 | -5.54811 | 1.06E-07 | 8.34E-07 | 7.155438 |
| CLVS1 | -3.03057 | 6.20842 | -3.7633 | 0.000229 | 0.000818 | -0.17414 |
| PPFIA3 | -3.03378 | 9.441928 | -8.28198 | 3.15E-14 | 1.30E-12 | 21.80405 |
| ANKRD24 | -3.0352 | 7.750576 | -6.3747 | 1.60E-09 | 1.95E-08 | 11.22248 |
| ALOX12B | -3.04269 | 2.591811 | -5.32279 | 3.13E-07 | 2.22E-06 | 6.113823 |
| CHP2 | -3.04535 | 1.277934 | -6.32518 | 2.08E-09 | 2.47E-08 | 10.96866 |
| VWC2L | -3.04784 | 5.349123 | -4.58714 | 8.58E-06 | 4.31E-05 | 2.936425 |
| NPTX2 | -3.0489 | 9.949605 | -3.53903 | 0.000515 | 0.001672 | -0.93161 |
| JPH1 | -3.04961 | 6.845202 | -4.44675 | 1.55E-05 | 7.35E-05 | 2.371406 |
| LRRC53 | -3.05385 | 2.196808 | -4.49486 | 1.27E-05 | 6.14E-05 | 2.563465 |
| FAM81A | -3.05571 | 8.031631 | -6.6018 | 4.76E-10 | 6.59E-09 | 12.40196 |
| CFAP74 | -3.05575 | 5.081856 | -5.77155 | 3.54E-08 | 3.09E-07 | 8.2178 |
| MRAP2 | -3.05686 | 7.43674 | -4.32392 | 2.58E-05 | 0.000116 | 1.888516 |
| GUCA1C | -3.05699 | 0.401634 | -10.83 | 3.30E-21 | 8.32E-19 | 37.58003 |
| DRGX | -3.05892 | 1.554277 | -5.03748 | 1.18E-06 | 7.22E-06 | 4.839703 |
| RHO | -3.05999 | 2.172684 | -6.19873 | 4.03E-09 | 4.48E-08 | 10.32612 |
| TH | -3.06386 | 2.368133 | -4.5562 | 9.79E-06 | 4.86E-05 | 2.810739 |
| PHACTR3 | -3.06406 | 9.24729 | -4.18287 | 4.56E-05 | 0.000194 | 1.347466 |
| KCNQ3 | -3.06782 | 9.384448 | -7.55477 | 2.31E-12 | 5.75E-11 | 17.60249 |
| RNF128 | -3.06882 | 5.772286 | -3.37851 | 0.0009 | 0.002744 | -1.44904 |
| RAD51AP2 | -3.07037 | 1.599989 | -4.68853 | 5.55E-06 | 2.89E-05 | 3.353024 |
| ATP6V1G2 | -3.07369 | 10.21573 | -6.10218 | 6.64E-09 | 6.97E-08 | 9.841059 |
| FRAS1 | -3.07375 | 6.491463 | -3.47792 | 0.000639 | 0.002025 | -1.13106 |
| MYRIP | -3.07594 | 8.527338 | -6.05595 | 8.41E-09 | 8.58E-08 | 9.610565 |
| GUCA1A | -3.07767 | 3.629063 | -3.61326 | 0.000396 | 0.001329 | -0.68534 |
| MAGEC3 | -3.07797 | 2.311356 | -4.91681 | 2.03E-06 | 1.18E-05 | 4.316486 |
| P2RX5 | -3.07825 | 4.877016 | -6.44084 | 1.13E-09 | 1.43E-08 | 11.56341 |
| CHN1 | -3.07854 | 11.8772 | -8.67969 | 2.81E-15 | 1.55E-13 | 24.17209 |
| SPOCK1 | -3.07947 | 11.1288 | -4.54692 | 1.02E-05 | 5.04E-05 | 2.77314 |
| PEG3 | -3.0805 | 10.80976 | -5.32596 | 3.08E-07 | 2.19E-06 | 6.128258 |
| NAV3 | -3.08151 | 8.682114 | -6.11981 | 6.06E-09 | 6.42E-08 | 9.929278 |
| MKX | -3.08231 | 4.864638 | -3.5962 | 0.000421 | 0.001402 | -0.74231 |
| GPR149 | -3.08284 | 0.646987 | -8.79994 | 1.34E-15 | 8.02E-14 | 24.89669 |
| HRH2 | -3.08307 | 5.558946 | -4.77738 | 3.76E-06 | 2.04E-05 | 3.723846 |
| CYS1 | -3.0842 | 5.821179 | -6.19404 | 4.12E-09 | 4.58E-08 | 10.30247 |
| CYP46A1 | -3.08637 | 9.306867 | -7.14052 | 2.45E-11 | 4.69E-10 | 15.29499 |
| LONRF2 | -3.09011 | 10.65808 | -6.88628 | 1.01E-10 | 1.66E-09 | 13.91365 |
| PNMA6A | -3.09343 | 7.045974 | -5.65339 | 6.35E-08 | 5.23E-07 | 7.652421 |
| KCNA3 | -3.09382 | 4.560646 | -4.81187 | 3.23E-06 | 1.78E-05 | 3.869253 |
| NELL2 | -3.09555 | 10.86079 | -5.48007 | 1.48E-07 | 1.13E-06 | 6.837695 |
| GSTO2 | -3.09725 | 6.385637 | -8.00642 | 1.63E-13 | 5.60E-12 | 20.19112 |
| CNDP1 | -3.09761 | 9.166678 | -3.05813 | 0.00258 | 0.007034 | -2.41831 |
| CDK5R1 | -3.09818 | 10.35771 | -5.86461 | 2.22E-08 | 2.04E-07 | 8.66866 |
| NALCN | -3.09995 | 8.94636 | -6.49457 | 8.46E-10 | 1.10E-08 | 11.84193 |
| KCNH8 | -3.10128 | 6.847857 | -3.88466 | 0.000146 | 0.000546 | 0.252182 |
| NKX6-2 | -3.10325 | 7.36895 | -3.08231 | 0.00239 | 0.006576 | -2.34816 |
| GPRASP1 | -3.10357 | 10.19758 | -8.01988 | 1.51E-13 | 5.23E-12 | 20.26933 |
| ESYT3 | -3.10452 | 5.46381 | -7.23961 | 1.40E-11 | 2.87E-10 | 15.84076 |
| SGIP1 | -3.10751 | 8.92835 | -8.37802 | 1.76E-14 | 7.74E-13 | 22.37173 |
| KRT17 | -3.10754 | 5.378144 | -3.81573 | 0.000189 | 0.00069 | 0.008639 |
| ANKRD33B | -3.10756 | 7.703241 | -7.02461 | 4.68E-11 | 8.37E-10 | 14.66179 |
| EXD1 | -3.10818 | 1.621468 | -7.13327 | 2.55E-11 | 4.88E-10 | 15.25523 |
| PCDHAC2 | -3.11363 | 6.609704 | -7.01936 | 4.82E-11 | 8.59E-10 | 14.63328 |
| PCDH7 | -3.11421 | 8.985572 | -4.19358 | 4.37E-05 | 0.000186 | 1.388024 |
| CRYGN | -3.11467 | 2.712821 | -6.5504 | 6.28E-10 | 8.43E-09 | 12.13284 |
| HPSE2 | -3.11468 | 4.449418 | -3.49235 | 0.000607 | 0.001935 | -1.08422 |
| FGF22 | -3.11834 | 3.282911 | -6.07301 | 7.71E-09 | 7.92E-08 | 9.695483 |
| MYADML2 | -3.11946 | 4.320358 | -7.42211 | 4.95E-12 | 1.14E-10 | 16.85625 |
| KIF5C | -3.11969 | 12.02868 | -7.19011 | 1.85E-11 | 3.69E-10 | 15.56765 |
| ATCAY | -3.1198 | 10.59996 | -3.26795 | 0.001306 | 0.003834 | -1.79317 |
| STXBP5 | -3.12467 | 8.70187 | -9.64653 | 6.72E-18 | 7.22E-16 | 30.09514 |
| GOLGA7B | -3.12956 | 8.73961 | -5.35656 | 2.67E-07 | 1.92E-06 | 6.26798 |
| RPS6KA6 | -3.12983 | 5.862274 | -3.76627 | 0.000227 | 0.00081 | -0.16385 |
| CEP170B | -3.13148 | 10.10693 | -7.62025 | 1.58E-12 | 4.10E-11 | 17.97326 |
| STMN4 | -3.13789 | 10.41767 | -4.53163 | 1.09E-05 | 5.35E-05 | 2.711383 |
| PRRT2 | -3.13874 | 9.405884 | -6.17371 | 4.58E-09 | 5.02E-08 | 10.19998 |
| TMEM132C | -3.14293 | 7.059631 | -3.29867 | 0.001179 | 0.003493 | -1.69856 |
| OR2W3 | -3.14295 | 2.571235 | -5.82034 | 2.78E-08 | 2.50E-07 | 8.453577 |
| FAM155A | -3.14435 | 7.909801 | -4.77248 | 3.85E-06 | 2.08E-05 | 3.703284 |
| KIAA2022 | -3.14458 | 7.377721 | -3.73191 | 0.000257 | 0.000907 | -0.28257 |
| SYT9 | -3.14551 | 6.486726 | -4.37302 | 2.11E-05 | 9.68E-05 | 2.080256 |
| MGAT4C | -3.14701 | 6.252058 | -3.89714 | 0.000139 | 0.000523 | 0.296658 |
| PNMT | -3.14701 | 4.940946 | -3.73014 | 0.000259 | 0.000912 | -0.28866 |
| TRIM17 | -3.15067 | 5.326928 | -7.18293 | 1.93E-11 | 3.80E-10 | 15.52809 |
| SPRN | -3.15387 | 7.754765 | -8.08027 | 1.05E-13 | 3.79E-12 | 20.62101 |
| ABCG8 | -3.15621 | 1.085795 | -7.19115 | 1.84E-11 | 3.67E-10 | 15.57338 |
| TUBA4A | -3.15768 | 10.10944 | -6.20883 | 3.82E-09 | 4.28E-08 | 10.37717 |
| BCL11B | -3.15949 | 6.279622 | -5.50151 | 1.33E-07 | 1.02E-06 | 6.937523 |
| ANKRD34A | -3.16668 | 7.515409 | -7.88779 | 3.30E-13 | 1.03E-11 | 19.50432 |
| EPHA7 | -3.17268 | 6.469308 | -4.01586 | 8.80E-05 | 0.000348 | 0.725797 |
| SLC38A8 | -3.17388 | 2.427275 | -6.01083 | 1.06E-08 | 1.06E-07 | 9.386657 |
| CYP4Z1 | -3.17415 | 0.912283 | -7.48677 | 3.41E-12 | 8.14E-11 | 17.21912 |
| TNIP3 | -3.17807 | 2.024581 | -5.16036 | 6.69E-07 | 4.37E-06 | 5.382191 |
| AGBL4 | -3.18319 | 6.08509 | -6.46996 | 9.65E-10 | 1.24E-08 | 11.71422 |
| ANKRD18B | -3.18551 | 2.037097 | -4.72407 | 4.75E-06 | 2.52E-05 | 3.500732 |
| PRKCE | -3.1865 | 9.484888 | -10.6469 | 1.09E-20 | 2.30E-18 | 36.40958 |
| CHRM4 | -3.18831 | 5.21811 | -5.8726 | 2.14E-08 | 1.98E-07 | 8.707608 |
| C17orf107 | -3.18975 | 5.105061 | -10.4445 | 4.03E-20 | 7.41E-18 | 35.12035 |
| KCNC1 | -3.1898 | 8.580779 | -5.8563 | 2.32E-08 | 2.12E-07 | 8.628212 |
| EEF1A2 | -3.19018 | 11.85886 | -4.79669 | 3.46E-06 | 1.90E-05 | 3.805159 |
| TPBGL | -3.19683 | 5.376459 | -6.84892 | 1.24E-10 | 1.99E-09 | 13.71304 |
| TSPOAP1 | -3.20221 | 9.815618 | -5.81098 | 2.91E-08 | 2.60E-07 | 8.408251 |
| TLL1 | -3.20503 | 4.105598 | -4.55 | 1.00E-05 | 4.98E-05 | 2.785636 |
| FRMPD2 | -3.20538 | 4.041988 | -5.40036 | 2.16E-07 | 1.59E-06 | 6.468979 |
| SGCD | -3.20553 | 6.963886 | -4.07086 | 7.11E-05 | 0.000287 | 0.928209 |
| MAPK8IP2 | -3.20973 | 10.5734 | -6.9167 | 8.52E-11 | 1.43E-09 | 14.07749 |
| TDRD5 | -3.21118 | 2.684241 | -5.42216 | 1.95E-07 | 1.45E-06 | 6.569438 |
| MBP | -3.21446 | 13.88977 | -3.27553 | 0.001273 | 0.003746 | -1.7699 |
| LRRTM1 | -3.21575 | 7.365541 | -5.07446 | 9.93E-07 | 6.21E-06 | 5.00196 |
| RAB27B | -3.21938 | 7.928464 | -5.07293 | 1.00E-06 | 6.25E-06 | 4.995234 |
| B3GNT4 | -3.22004 | 5.503218 | -9.61929 | 7.99E-18 | 8.31E-16 | 29.92549 |
| FUT9 | -3.22346 | 8.832089 | -4.18346 | 4.55E-05 | 0.000193 | 1.349673 |
| STAT4 | -3.22428 | 5.75882 | -6.43335 | 1.17E-09 | 1.48E-08 | 11.52472 |
| PPM1H | -3.22432 | 8.949363 | -8.22575 | 4.42E-14 | 1.76E-12 | 21.47298 |
| IL1RL2 | -3.22434 | 3.156322 | -5.46344 | 1.60E-07 | 1.21E-06 | 6.76046 |
| EXTL1 | -3.22587 | 7.962552 | -6.20765 | 3.84E-09 | 4.30E-08 | 10.37118 |
| CNTN2 | -3.22719 | 10.79793 | -4.34978 | 2.32E-05 | 0.000106 | 1.989273 |
| KCTD8 | -3.22799 | 6.256169 | -5.00534 | 1.36E-06 | 8.24E-06 | 4.699434 |
| VSTM5 | -3.22916 | 4.913494 | -5.68126 | 5.54E-08 | 4.63E-07 | 7.785059 |
| ERMN | -3.23319 | 9.968241 | -3.83751 | 0.000174 | 0.00064 | 0.085191 |
| EDIL3 | -3.23449 | 10.46838 | -4.75852 | 4.09E-06 | 2.20E-05 | 3.644696 |
| NPHS1 | -3.23502 | 2.458906 | -8.05351 | 1.24E-13 | 4.36E-12 | 20.46504 |
| FGF9 | -3.23557 | 5.388441 | -4.06447 | 7.29E-05 | 0.000294 | 0.904581 |
| TMEFF2 | -3.24088 | 8.474679 | -3.63675 | 0.000364 | 0.001231 | -0.60647 |
| RTN4RL2 | -3.24112 | 7.746234 | -5.77879 | 3.42E-08 | 3.00E-07 | 8.252731 |
| ZNF365 | -3.24141 | 9.240873 | -7.53184 | 2.63E-12 | 6.46E-11 | 17.47302 |
| ADAMTS8 | -3.24243 | 7.25017 | -6.18745 | 4.27E-09 | 4.71E-08 | 10.26921 |
| CBFA2T3 | -3.244 | 6.468391 | -7.64415 | 1.37E-12 | 3.64E-11 | 18.10902 |
| OLFM1 | -3.24481 | 11.86278 | -4.95977 | 1.67E-06 | 9.92E-06 | 4.50166 |
| XKR4 | -3.245 | 7.54572 | -4.3929 | 1.94E-05 | 9.00E-05 | 2.158394 |
| AGAP2 | -3.24791 | 10.87912 | -3.99223 | 9.65E-05 | 0.000377 | 0.639528 |
| PTGDS | -3.24806 | 12.58322 | -4.5417 | 1.04E-05 | 5.14E-05 | 2.752031 |
| SLC1A2 | -3.25565 | 13.52704 | -5.29842 | 3.51E-07 | 2.45E-06 | 6.002993 |
| DLGAP1 | -3.2577 | 9.874325 | -5.6819 | 5.52E-08 | 4.62E-07 | 7.788094 |
| DOCK3 | -3.26063 | 9.710493 | -6.22011 | 3.60E-09 | 4.05E-08 | 10.4342 |
| FA2H | -3.26155 | 7.853046 | -3.68336 | 0.000307 | 0.00106 | -0.44869 |
| DNM3 | -3.26278 | 9.670494 | -6.69161 | 2.93E-10 | 4.27E-09 | 12.87521 |
| SORCS1 | -3.2642 | 7.578984 | -4.24783 | 3.51E-05 | 0.000153 | 1.594824 |
| CNTN5 | -3.26426 | 5.862913 | -4.5246 | 1.12E-05 | 5.49E-05 | 2.683022 |
| TMEM179 | -3.26531 | 8.392661 | -4.56146 | 9.57E-06 | 4.76E-05 | 2.832052 |
| FABP6 | -3.26636 | 4.234469 | -5.00026 | 1.39E-06 | 8.42E-06 | 4.677314 |
| POLR2F | -3.26905 | 6.214721 | -5.57741 | 9.22E-08 | 7.34E-07 | 7.293104 |
| HHIP | -3.26982 | 7.506847 | -4.28838 | 2.98E-05 | 0.000132 | 1.750809 |
| KLK10 | -3.27135 | 2.464233 | -5.96977 | 1.31E-08 | 1.28E-07 | 9.183884 |
| ZNF98 | -3.27203 | 2.401731 | -3.83246 | 0.000177 | 0.000651 | 0.067391 |
| MAP1A | -3.2753 | 12.99382 | -8.48583 | 9.18E-15 | 4.35E-13 | 23.01221 |
| TYRP1 | -3.27836 | 2.795263 | -4.19748 | 4.30E-05 | 0.000183 | 1.402811 |
| RTL1 | -3.27859 | 3.069756 | -4.48926 | 1.30E-05 | 6.27E-05 | 2.541059 |
| SH3RF2 | -3.2853 | 3.760068 | -4.77443 | 3.81E-06 | 2.07E-05 | 3.711478 |
| RASGEF1A | -3.28544 | 7.871244 | -6.4017 | 1.39E-09 | 1.72E-08 | 11.3614 |
| RTN1 | -3.28709 | 11.93255 | -5.75652 | 3.82E-08 | 3.32E-07 | 8.145442 |
| WNT7B | -3.28832 | 6.989053 | -4.05736 | 7.49E-05 | 0.000301 | 0.878339 |
| GPR150 | -3.28965 | 3.861515 | -6.26752 | 2.81E-09 | 3.23E-08 | 10.6747 |
| SLCO1A2 | -3.29035 | 8.466428 | -4.35289 | 2.29E-05 | 0.000104 | 2.001423 |
| STUM | -3.29322 | 9.498521 | -5.28723 | 3.70E-07 | 2.57E-06 | 5.952254 |
| GABRA6 | -3.30099 | 0.743262 | -8.03298 | 1.40E-13 | 4.87E-12 | 20.34552 |
| C11orf97 | -3.30194 | 1.509231 | -4.53591 | 1.07E-05 | 5.26E-05 | 2.728651 |
| PCDHA5 | -3.30399 | 3.505638 | -5.13385 | 7.56E-07 | 4.86E-06 | 5.264355 |
| CACNA1S | -3.30641 | 3.011063 | -5.2278 | 4.89E-07 | 3.28E-06 | 5.683968 |
| AC005885.1 | -3.30666 | 0.706488 | -8.68646 | 2.70E-15 | 1.51E-13 | 24.21278 |
| ADRB3 | -3.3088 | 1.698563 | -6.81609 | 1.48E-10 | 2.34E-09 | 13.53727 |
| PRRT1 | -3.31335 | 9.0446 | -10.7584 | 5.26E-21 | 1.29E-18 | 37.1217 |
| MICAL2 | -3.31924 | 9.829128 | -6.83624 | 1.33E-10 | 2.11E-09 | 13.6451 |
| C2CD4C | -3.32438 | 7.590745 | -6.37674 | 1.58E-09 | 1.93E-08 | 11.233 |
| SLC25A27 | -3.32823 | 8.089934 | -6.16347 | 4.83E-09 | 5.25E-08 | 10.14844 |
| SLC6A13 | -3.33224 | 5.377577 | -6.22889 | 3.44E-09 | 3.89E-08 | 10.47864 |
| IL1RAPL1 | -3.33262 | 4.349214 | -4.78397 | 3.66E-06 | 1.99E-05 | 3.751578 |
| NECAB1 | -3.33648 | 9.4823 | -8.30082 | 2.81E-14 | 1.18E-12 | 21.91517 |
| RAP1GAP2 | -3.33726 | 9.389456 | -6.67415 | 3.22E-10 | 4.66E-09 | 12.78292 |
| KCNAB1 | -3.33948 | 8.118881 | -9.8598 | 1.73E-18 | 2.19E-16 | 31.42798 |
| NOS1AP | -3.34735 | 7.975099 | -7.83087 | 4.61E-13 | 1.37E-11 | 19.17646 |
| SERPIND1 | -3.34767 | 2.824269 | -4.76071 | 4.05E-06 | 2.18E-05 | 3.653885 |
| DIRAS2 | -3.34843 | 9.55559 | -5.57654 | 9.25E-08 | 7.36E-07 | 7.289013 |
| PTH2R | -3.34922 | 4.053061 | -5.13546 | 7.50E-07 | 4.83E-06 | 5.271502 |
| CHRNB3 | -3.35182 | 1.026845 | -6.62853 | 4.12E-10 | 5.80E-09 | 12.54244 |
| TMEM125 | -3.35629 | 6.424702 | -3.36978 | 0.000927 | 0.002821 | -1.47657 |
| GAD1 | -3.35754 | 9.446254 | -4.84579 | 2.78E-06 | 1.57E-05 | 4.013014 |
| SCN8A | -3.3621 | 8.919118 | -8.71237 | 2.30E-15 | 1.31E-13 | 24.36865 |
| TRIM72 | -3.36478 | 3.107446 | -8.88816 | 7.79E-16 | 5.00E-14 | 25.43067 |
| CHAD | -3.36949 | 4.448504 | -5.26591 | 4.09E-07 | 2.81E-06 | 5.855771 |
| KCNN1 | -3.37024 | 7.973073 | -6.10179 | 6.65E-09 | 6.97E-08 | 9.839135 |
| PVALB | -3.37072 | 3.926544 | -3.57752 | 0.00045 | 0.001484 | -0.80447 |
| HHATL | -3.3744 | 8.352576 | -4.63662 | 6.94E-06 | 3.54E-05 | 3.138845 |
| CLSTN2 | -3.37825 | 9.754417 | -5.07122 | 1.01E-06 | 6.29E-06 | 4.987711 |
| FFAR1 | -3.37998 | 1.650786 | -5.7347 | 4.25E-08 | 3.65E-07 | 8.040612 |
| CNTN6 | -3.38513 | 5.036122 | -7.03209 | 4.49E-11 | 8.11E-10 | 14.70249 |
| EPCAM | -3.38522 | 4.473554 | -7.12212 | 2.71E-11 | 5.14E-10 | 15.19413 |
| SYNGR1 | -3.39214 | 11.1195 | -10.0019 | 6.97E-19 | 9.58E-17 | 32.32052 |
| ARFGEF3 | -3.3931 | 9.68491 | -6.33263 | 2.00E-09 | 2.39E-08 | 11.0068 |
| GPR62 | -3.39414 | 5.90262 | -4.80095 | 3.39E-06 | 1.86E-05 | 3.823142 |
| ALOXE3 | -3.39472 | 3.169232 | -5.92533 | 1.64E-08 | 1.55E-07 | 8.965418 |
| ATP2B2 | -3.39637 | 10.33685 | -5.66316 | 6.06E-08 | 5.01E-07 | 7.698885 |
| PNOC | -3.39727 | 5.7469 | -3.4245 | 0.000769 | 0.002385 | -1.30293 |
| SEMA4A | -3.39771 | 8.409317 | -9.97151 | 8.47E-19 | 1.16E-16 | 32.12922 |
| GPR12 | -3.39826 | 6.572203 | -3.9251 | 0.000125 | 0.000476 | 0.396755 |
| ADCY5 | -3.40386 | 9.1492 | -6.17839 | 4.47E-09 | 4.91E-08 | 10.22357 |
| ZMAT4 | -3.40607 | 5.797547 | -3.93578 | 0.00012 | 0.000458 | 0.435138 |
| GRIN2C | -3.40767 | 7.758089 | -5.33193 | 3.00E-07 | 2.13E-06 | 6.155478 |
| PDE1B | -3.41113 | 8.559338 | -8.21218 | 4.79E-14 | 1.88E-12 | 21.3932 |
| CHRNB2 | -3.41197 | 8.388281 | -5.36 | 2.62E-07 | 1.90E-06 | 6.283712 |
| RCAN2 | -3.41474 | 9.9061 | -5.16273 | 6.61E-07 | 4.33E-06 | 5.392722 |
| BHMT | -3.41677 | 2.16566 | -6.12897 | 5.78E-09 | 6.15E-08 | 9.97518 |
| GPR78 | -3.41795 | 1.729034 | -6.54032 | 6.63E-10 | 8.85E-09 | 12.08019 |
| XK | -3.42353 | 6.348764 | -5.39219 | 2.25E-07 | 1.65E-06 | 6.431364 |
| NRSN1 | -3.42472 | 8.625943 | -5.36005 | 2.62E-07 | 1.90E-06 | 6.283944 |
| ATRNL1 | -3.42929 | 8.84153 | -5.22852 | 4.87E-07 | 3.27E-06 | 5.687205 |
| NXPH2 | -3.42997 | 3.573965 | -4.32774 | 2.54E-05 | 0.000114 | 1.903385 |
| FBXO2 | -3.43168 | 9.475333 | -5.64968 | 6.47E-08 | 5.32E-07 | 7.634773 |
| TTC9B | -3.43626 | 7.876014 | -4.6023 | 8.04E-06 | 4.06E-05 | 2.998269 |
| ETNPPL | -3.43631 | 8.737576 | -3.96597 | 0.000107 | 0.000413 | 0.544142 |
| ANKRD63 | -3.43792 | 1.753332 | -6.29911 | 2.38E-09 | 2.80E-08 | 10.83555 |
| ANKRD18A | -3.44119 | 2.338261 | -4.59571 | 8.27E-06 | 4.16E-05 | 2.971365 |
| BCL11A | -3.44119 | 7.66457 | -5.71101 | 4.78E-08 | 4.05E-07 | 7.927138 |
| CREB3L3 | -3.44163 | 0.900649 | -8.34679 | 2.13E-14 | 9.19E-13 | 22.18679 |
| ADCY1 | -3.44311 | 9.812256 | -5.74675 | 4.01E-08 | 3.46E-07 | 8.098505 |
| MAG | -3.44387 | 10.00009 | -3.31193 | 0.001127 | 0.003358 | -1.65749 |
| GFOD1 | -3.44921 | 8.868519 | -9.62306 | 7.80E-18 | 8.16E-16 | 29.94902 |
| TINCR | -3.45823 | 6.266191 | -7.94343 | 2.37E-13 | 7.74E-12 | 19.82583 |
| TUBA8 | -3.45942 | 3.549517 | -8.13038 | 7.82E-14 | 2.89E-12 | 20.91369 |
| CORO6 | -3.45944 | 6.391017 | -6.9926 | 5.60E-11 | 9.76E-10 | 14.48794 |
| AMPH | -3.46144 | 9.565744 | -6.55704 | 6.06E-10 | 8.17E-09 | 12.16756 |
| PPP1R1A | -3.46586 | 7.259162 | -4.21538 | 4.00E-05 | 0.000172 | 1.470854 |
| JPH4 | -3.46655 | 9.970969 | -4.82701 | 3.02E-06 | 1.68E-05 | 3.933317 |
| PDIA2 | -3.4682 | 6.862575 | -5.03144 | 1.21E-06 | 7.40E-06 | 4.813304 |
| HTR3A | -3.46905 | 1.864523 | -5.28965 | 3.66E-07 | 2.54E-06 | 5.963233 |
| ARPP21 | -3.48082 | 8.737129 | -5.86031 | 2.27E-08 | 2.08E-07 | 8.6477 |
| TNNT1 | -3.48225 | 5.547084 | -5.39924 | 2.18E-07 | 1.60E-06 | 6.463817 |
| KRT33B | -3.48342 | 0.354375 | -13.9932 | 2.84E-30 | 1.12E-26 | 58.09017 |
| RFPL2 | -3.48448 | 3.428264 | -5.89605 | 1.90E-08 | 1.77E-07 | 8.822066 |
| AARD | -3.48905 | 2.228899 | -5.19591 | 5.67E-07 | 3.76E-06 | 5.540918 |
| NCDN | -3.49234 | 11.93438 | -9.80773 | 2.41E-18 | 2.87E-16 | 31.10177 |
| CCSER1 | -3.49812 | 3.068596 | -6.88334 | 1.02E-10 | 1.69E-09 | 13.89786 |
| STAR | -3.50014 | 4.456075 | -7.82617 | 4.74E-13 | 1.40E-11 | 19.14946 |
| GPR68 | -3.50099 | 5.724084 | -7.06937 | 3.65E-11 | 6.70E-10 | 14.90564 |
| AQP7 | -3.50219 | 1.855227 | -6.74399 | 2.20E-10 | 3.34E-09 | 13.1529 |
| FAM19A4 | -3.50402 | 3.448272 | -5.05925 | 1.06E-06 | 6.61E-06 | 4.935113 |
| SYN3 | -3.50471 | 6.041386 | -6.16864 | 4.71E-09 | 5.13E-08 | 10.17446 |
| LDB3 | -3.50541 | 6.82209 | -5.87277 | 2.13E-08 | 1.97E-07 | 8.708391 |
| RTN4R | -3.50833 | 7.680984 | -6.8282 | 1.39E-10 | 2.20E-09 | 13.60207 |
| HAS1 | -3.50957 | 3.96943 | -4.8313 | 2.97E-06 | 1.66E-05 | 3.951501 |
| PDZD7 | -3.51092 | 6.320216 | -9.26411 | 7.50E-17 | 6.09E-15 | 27.72728 |
| INSM2 | -3.51351 | 3.266628 | -4.64268 | 6.76E-06 | 3.46E-05 | 3.163769 |
| HLF | -3.51507 | 8.822362 | -6.7148 | 2.58E-10 | 3.82E-09 | 12.998 |
| SLC22A10 | -3.52192 | 0.863313 | -8.5166 | 7.61E-15 | 3.77E-13 | 23.19558 |
| NTSR1 | -3.52339 | 4.483959 | -4.20705 | 4.14E-05 | 0.000177 | 1.439191 |
| FAM196B | -3.53055 | 4.74283 | -5.18604 | 5.94E-07 | 3.92E-06 | 5.496753 |
| CAPN13 | -3.54307 | 2.421847 | -5.43867 | 1.80E-07 | 1.35E-06 | 6.645688 |
| MTMR7 | -3.54361 | 7.156987 | -8.80973 | 1.26E-15 | 7.62E-14 | 24.95587 |
| CA11 | -3.54383 | 10.72462 | -7.96552 | 2.08E-13 | 6.93E-12 | 19.95378 |
| KNDC1 | -3.54847 | 9.40358 | -6.42904 | 1.20E-09 | 1.51E-08 | 11.50246 |
| CALB1 | -3.55539 | 6.315443 | -5.07946 | 9.71E-07 | 6.08E-06 | 5.023961 |
| RUNDC3A | -3.55548 | 10.36137 | -5.75916 | 3.77E-08 | 3.28E-07 | 8.158163 |
| POU6F2 | -3.55654 | 4.186219 | -5.21236 | 5.25E-07 | 3.50E-06 | 5.614618 |
| SCARA5 | -3.5582 | 4.00892 | -4.22153 | 3.91E-05 | 0.000168 | 1.494291 |
| CAMK2B | -3.55951 | 10.58291 | -5.63928 | 6.81E-08 | 5.56E-07 | 7.585425 |
| OTUD7A | -3.56152 | 8.08474 | -8.00947 | 1.61E-13 | 5.53E-12 | 20.20886 |
| FAM163A | -3.56169 | 2.42245 | -4.58513 | 8.65E-06 | 4.34E-05 | 2.92823 |
| TGFBR3L | -3.5643 | 4.793806 | -6.31806 | 2.16E-09 | 2.56E-08 | 10.93228 |
| NRIP3 | -3.56561 | 8.34124 | -6.94194 | 7.41E-11 | 1.26E-09 | 14.21368 |
| KIF1A | -3.56587 | 13.04686 | -5.70842 | 4.84E-08 | 4.10E-07 | 7.914738 |
| CD22 | -3.56701 | 6.398499 | -5.33719 | 2.92E-07 | 2.09E-06 | 6.179497 |
| MAL | -3.56833 | 8.48702 | -4.45011 | 1.53E-05 | 7.26E-05 | 2.384787 |
| HNF4A | -3.56964 | 0.997731 | -7.57058 | 2.10E-12 | 5.30E-11 | 17.69187 |
| OPCML | -3.57005 | 9.361594 | -4.52808 | 1.10E-05 | 5.42E-05 | 2.697047 |
| UBE2QL1 | -3.57037 | 8.56366 | -5.35359 | 2.70E-07 | 1.95E-06 | 6.254387 |
| RPH3A | -3.57182 | 9.685826 | -3.69992 | 0.000289 | 0.001005 | -0.39225 |
| TAGLN3 | -3.57216 | 10.21527 | -4.82302 | 3.08E-06 | 1.71E-05 | 3.916427 |
| SCRT1 | -3.57549 | 6.785827 | -4.00351 | 9.24E-05 | 0.000363 | 0.68066 |
| CAMSAP3 | -3.58117 | 7.077706 | -4.35748 | 2.25E-05 | 0.000102 | 2.019398 |
| ST6GALNAC5 | -3.58513 | 6.769619 | -4.69565 | 5.38E-06 | 2.81E-05 | 3.382549 |
| GRIP2 | -3.5889 | 6.050384 | -4.97717 | 1.55E-06 | 9.24E-06 | 4.577046 |
| ASPDH | -3.59064 | 6.380935 | -6.79869 | 1.63E-10 | 2.56E-09 | 13.44431 |
| SHISA7 | -3.59243 | 8.04987 | -4.15664 | 5.06E-05 | 0.000212 | 1.24846 |
| FAIM2 | -3.59623 | 12.2912 | -8.47599 | 9.74E-15 | 4.61E-13 | 22.95357 |
| ANKS1B | -3.59702 | 9.181698 | -5.89089 | 1.95E-08 | 1.81E-07 | 8.79685 |
| GABBR2 | -3.59767 | 9.988177 | -4.69168 | 5.47E-06 | 2.85E-05 | 3.366089 |
| TMEM196 | -3.5982 | 5.411446 | -3.93459 | 0.00012 | 0.00046 | 0.430876 |
| MCF2L2 | -3.60241 | 7.232669 | -6.90398 | 9.15E-11 | 1.52E-09 | 14.00891 |
| OPRD1 | -3.60265 | 6.257502 | -6.00867 | 1.07E-08 | 1.07E-07 | 9.375955 |
| CASKIN1 | -3.60399 | 8.645875 | -6.39089 | 1.47E-09 | 1.81E-08 | 11.30578 |
| NPBWR1 | -3.60608 | 0.86451 | -8.83218 | 1.10E-15 | 6.80E-14 | 25.0916 |
| VAX1 | -3.61012 | 3.610466 | -3.99828 | 9.43E-05 | 0.000369 | 0.661559 |
| SSTR2 | -3.61095 | 8.106981 | -6.7877 | 1.73E-10 | 2.70E-09 | 13.38566 |
| GPR179 | -3.61125 | 5.113261 | -5.74187 | 4.11E-08 | 3.54E-07 | 8.07503 |
| MAGEE1 | -3.6141 | 8.330945 | -6.77249 | 1.88E-10 | 2.90E-09 | 13.30454 |
| SLC9A2 | -3.61573 | 3.388948 | -5.32921 | 3.03E-07 | 2.16E-06 | 6.143058 |
| ADRA2C | -3.61777 | 4.69683 | -5.44338 | 1.76E-07 | 1.32E-06 | 6.667484 |
| BEX5 | -3.61887 | 7.735732 | -5.66456 | 6.01E-08 | 4.98E-07 | 7.705525 |
| RPRML | -3.61891 | 4.730185 | -5.16427 | 6.57E-07 | 4.30E-06 | 5.399578 |
| SMYD1 | -3.62339 | 3.318728 | -5.78173 | 3.37E-08 | 2.97E-07 | 8.266875 |
| MAPK15 | -3.62597 | 4.201294 | -3.86512 | 0.000157 | 0.000583 | 0.182746 |
| SOSTDC1 | -3.62624 | 4.548802 | -5.05427 | 1.09E-06 | 6.75E-06 | 4.913268 |
| RIMS3 | -3.62791 | 9.779416 | -6.87274 | 1.09E-10 | 1.78E-09 | 13.84089 |
| ANO4 | -3.62828 | 6.542281 | -5.73321 | 4.29E-08 | 3.68E-07 | 8.033499 |
| UNC5D | -3.62859 | 6.975201 | -4.46879 | 1.42E-05 | 6.77E-05 | 2.459215 |
| TAC3 | -3.63098 | 5.75196 | -4.26336 | 3.30E-05 | 0.000145 | 1.654427 |
| KCNAB2 | -3.63138 | 10.59691 | -8.95328 | 5.20E-16 | 3.43E-14 | 25.82608 |
| THEM5 | -3.63666 | 4.825978 | -5.95721 | 1.39E-08 | 1.35E-07 | 9.122021 |
| C3orf80 | -3.63681 | 5.947894 | -6.85564 | 1.19E-10 | 1.93E-09 | 13.74909 |
| MOBP | -3.64025 | 9.404722 | -3.31534 | 0.001115 | 0.003324 | -1.6469 |
| KCNB1 | -3.64104 | 8.824307 | -5.39927 | 2.18E-07 | 1.60E-06 | 6.463967 |
| TENM3 | -3.64135 | 8.165964 | -3.73722 | 0.000252 | 0.000892 | -0.26426 |
| ENC1 | -3.64137 | 12.61014 | -8.48905 | 9.00E-15 | 4.29E-13 | 23.03138 |
| ANKRD2 | -3.64181 | 3.158996 | -7.24908 | 1.33E-11 | 2.74E-10 | 15.89314 |
| CLCA4 | -3.64558 | 4.141155 | -3.79245 | 0.000206 | 0.000745 | -0.07279 |
| ACP7 | -3.64583 | 3.076478 | -4.79742 | 3.45E-06 | 1.89E-05 | 3.808235 |
| HSPA12A | -3.65137 | 9.464914 | -8.53696 | 6.72E-15 | 3.38E-13 | 23.31712 |
| CPEB3 | -3.6538 | 7.783407 | -10.8569 | 2.77E-21 | 7.26E-19 | 37.75211 |
| DNAJC6 | -3.65492 | 10.66731 | -8.36951 | 1.86E-14 | 8.10E-13 | 22.32129 |
| PCDH11Y | -3.65585 | 2.7262 | -3.70578 | 0.000283 | 0.000986 | -0.37221 |
| CHRM3 | -3.65595 | 7.984856 | -4.73947 | 4.44E-06 | 2.37E-05 | 3.564965 |
| BICDL1 | -3.65744 | 6.524466 | -6.59543 | 4.93E-10 | 6.80E-09 | 12.36852 |
| DMTN | -3.6577 | 10.3542 | -7.71213 | 9.24E-13 | 2.56E-11 | 18.49624 |
| MAST3 | -3.65983 | 10.32281 | -10.5637 | 1.86E-20 | 3.72E-18 | 35.87899 |
| CORT | -3.66941 | 4.591225 | -8.03838 | 1.35E-13 | 4.73E-12 | 20.37692 |
| KIAA1107 | -3.67124 | 8.028507 | -9.50627 | 1.63E-17 | 1.57E-15 | 29.22325 |
| ERICH3 | -3.67258 | 7.762348 | -5.09661 | 8.97E-07 | 5.67E-06 | 5.099555 |
| C10orf82 | -3.67391 | 2.107202 | -6.642 | 3.83E-10 | 5.46E-09 | 12.61333 |
| HSPB3 | -3.6803 | 3.680562 | -4.39622 | 1.92E-05 | 8.90E-05 | 2.171439 |
| KCNQ5 | -3.68326 | 7.693492 | -4.88589 | 2.33E-06 | 1.34E-05 | 4.183958 |
| ZNF536 | -3.68348 | 7.318532 | -5.04644 | 1.13E-06 | 6.97E-06 | 4.878954 |
| NPTXR | -3.68523 | 11.86018 | -7.59978 | 1.78E-12 | 4.56E-11 | 17.85722 |
| MYH7B | -3.68535 | 6.279838 | -7.713 | 9.20E-13 | 2.55E-11 | 18.50116 |
| PCDHA6 | -3.68877 | 3.768106 | -3.96596 | 0.000107 | 0.000413 | 0.544104 |
| STX1A | -3.69019 | 9.455257 | -8.75488 | 1.77E-15 | 1.03E-13 | 24.62473 |
| GDF10 | -3.69027 | 4.537948 | -4.53286 | 1.08E-05 | 5.32E-05 | 2.716328 |
| SRCIN1 | -3.69052 | 9.652914 | -6.38185 | 1.54E-09 | 1.89E-08 | 11.25926 |
| NPFFR1 | -3.69134 | 3.699842 | -5.94458 | 1.48E-08 | 1.42E-07 | 9.05992 |
| SLC13A5 | -3.69416 | 5.754389 | -4.07095 | 7.10E-05 | 0.000287 | 0.928552 |
| IL1RAPL2 | -3.69712 | 2.063283 | -5.40452 | 2.12E-07 | 1.57E-06 | 6.488123 |
| LCNL1 | -3.69774 | 6.458413 | -4.66396 | 6.17E-06 | 3.18E-05 | 3.251425 |
| PRDM8 | -3.69776 | 7.45198 | -5.29854 | 3.51E-07 | 2.45E-06 | 6.003539 |
| VIPR1 | -3.69814 | 6.729329 | -7.11413 | 2.84E-11 | 5.35E-10 | 15.15035 |
| ABCG4 | -3.69913 | 6.146103 | -7.8583 | 3.92E-13 | 1.19E-11 | 19.33432 |
| PRSS3 | -3.69939 | 6.173773 | -6.29164 | 2.48E-09 | 2.90E-08 | 10.79746 |
| STXBP1 | -3.70377 | 11.66889 | -9.03626 | 3.11E-16 | 2.19E-14 | 26.33145 |
| NRG3 | -3.70533 | 7.224655 | -5.75362 | 3.87E-08 | 3.36E-07 | 8.131496 |
| HCRTR2 | -3.71311 | 0.778353 | -8.6013 | 4.54E-15 | 2.37E-13 | 23.70185 |
| CELF3 | -3.71804 | 8.740003 | -4.39349 | 1.94E-05 | 8.99E-05 | 2.160718 |
| DPP10 | -3.71984 | 7.708123 | -4.45511 | 1.50E-05 | 7.12E-05 | 2.404664 |
| THEMIS | -3.72274 | 4.274865 | -6.91687 | 8.52E-11 | 1.43E-09 | 14.0784 |
| GABRA3 | -3.72589 | 7.56305 | -4.17387 | 4.73E-05 | 0.0002 | 1.313418 |
| ENTPD3 | -3.73241 | 5.855265 | -5.35797 | 2.65E-07 | 1.92E-06 | 6.274418 |
| MACROD2 | -3.73242 | 6.602921 | -6.07276 | 7.72E-09 | 7.93E-08 | 9.69423 |
| OTOF | -3.73282 | 5.025942 | -6.83944 | 1.31E-10 | 2.08E-09 | 13.66224 |
| KIRREL3 | -3.7347 | 8.285018 | -6.17254 | 4.61E-09 | 5.04E-08 | 10.19409 |
| PRKCZ | -3.73626 | 9.112197 | -6.27605 | 2.69E-09 | 3.11E-08 | 10.71806 |
| CNTNAP5 | -3.74261 | 4.65863 | -4.14298 | 5.35E-05 | 0.000223 | 1.197093 |
| ZNF385B | -3.74411 | 5.719715 | -4.52716 | 1.11E-05 | 5.44E-05 | 2.693362 |
| CA10 | -3.75122 | 7.734522 | -3.3882 | 0.000871 | 0.002661 | -1.41838 |
| GLS2 | -3.75394 | 3.781718 | -9.54921 | 1.24E-17 | 1.24E-15 | 29.48976 |
| ZC3H12B | -3.7542 | 6.085658 | -5.18113 | 6.07E-07 | 4.01E-06 | 5.474802 |
| SEMA3D | -3.75445 | 5.60998 | -3.87852 | 0.000149 | 0.000557 | 0.230332 |
| ATP8A1 | -3.75485 | 9.893692 | -7.41658 | 5.11E-12 | 1.16E-10 | 16.82527 |
| PRLHR | -3.75722 | 2.805814 | -4.61933 | 7.48E-06 | 3.79E-05 | 3.067925 |
| NAP1L2 | -3.75817 | 8.398702 | -6.55877 | 6.00E-10 | 8.10E-09 | 12.17658 |
| KCNK4 | -3.75948 | 2.080654 | -6.86813 | 1.11E-10 | 1.81E-09 | 13.8161 |
| ADAP1 | -3.76253 | 8.100576 | -7.85692 | 3.96E-13 | 1.20E-11 | 19.32638 |
| OGDHL | -3.76356 | 8.045148 | -5.91708 | 1.71E-08 | 1.61E-07 | 8.924974 |
| AATK | -3.77335 | 9.70103 | -6.18667 | 4.29E-09 | 4.73E-08 | 10.26528 |
| SPOCK3 | -3.77587 | 8.662819 | -4.37399 | 2.10E-05 | 9.65E-05 | 2.084071 |
| TMEM246 | -3.78086 | 8.546943 | -5.04661 | 1.13E-06 | 6.97E-06 | 4.879692 |
| BDNF | -3.78748 | 6.272695 | -5.1036 | 8.69E-07 | 5.51E-06 | 5.130412 |
| PTPN3 | -3.78918 | 6.527883 | -4.93913 | 1.83E-06 | 1.08E-05 | 4.412535 |
| PITPNM3 | -3.79028 | 8.581265 | -7.61487 | 1.63E-12 | 4.22E-11 | 17.94279 |
| DGKE | -3.79159 | 7.02307 | -9.19519 | 1.15E-16 | 8.97E-15 | 27.30384 |
| CBLN4 | -3.79318 | 6.482013 | -4.21729 | 3.97E-05 | 0.000171 | 1.478141 |
| PNCK | -3.79452 | 7.991301 | -7.54966 | 2.37E-12 | 5.91E-11 | 17.57365 |
| CDKL5 | -3.79524 | 8.882429 | -9.78611 | 2.77E-18 | 3.28E-16 | 30.96652 |
| CYP1A1 | -3.79849 | 2.17415 | -7.05259 | 4.01E-11 | 7.31E-10 | 14.81415 |
| AKAP5 | -3.80401 | 7.375266 | -9.63045 | 7.44E-18 | 7.87E-16 | 29.99501 |
| HBQ1 | -3.80518 | 2.703759 | -6.55288 | 6.19E-10 | 8.33E-09 | 12.14578 |
| WNT1 | -3.8067 | 1.659551 | -7.57397 | 2.06E-12 | 5.22E-11 | 17.71106 |
| C1QTNF4 | -3.80855 | 6.660031 | -6.78003 | 1.81E-10 | 2.81E-09 | 13.34473 |
| CAMKK1 | -3.81174 | 9.217377 | -8.47269 | 9.94E-15 | 4.68E-13 | 22.93394 |
| KIF5A | -3.81284 | 11.92784 | -5.56542 | 9.77E-08 | 7.72E-07 | 7.236694 |
| KLHL34 | -3.81567 | 2.888508 | -6.44672 | 1.09E-09 | 1.39E-08 | 11.59386 |
| PRKG2 | -3.81845 | 3.53663 | -3.97298 | 0.000104 | 0.000403 | 0.569568 |
| SPTBN2 | -3.82248 | 10.12246 | -7.01782 | 4.86E-11 | 8.65E-10 | 14.62489 |
| ATOH7 | -3.82923 | 3.50463 | -7.8869 | 3.32E-13 | 1.03E-11 | 19.49916 |
| VWA7 | -3.83276 | 5.551336 | -8.93287 | 5.91E-16 | 3.86E-14 | 25.70202 |
| CAMK4 | -3.83637 | 8.03495 | -7.59886 | 1.79E-12 | 4.57E-11 | 17.85198 |
| GNG13 | -3.83816 | 3.385898 | -5.98455 | 1.21E-08 | 1.19E-07 | 9.256757 |
| SCN2A | -3.8423 | 9.331779 | -9.32924 | 4.98E-17 | 4.28E-15 | 28.12846 |
| RAPGEF4 | -3.84466 | 9.832613 | -7.97842 | 1.93E-13 | 6.47E-12 | 20.0286 |
| PNMA8B | -3.85203 | 8.712017 | -10.0738 | 4.40E-19 | 6.26E-17 | 32.77321 |
| VSTM2L | -3.85328 | 8.167596 | -5.56186 | 9.94E-08 | 7.85E-07 | 7.219957 |
| EPB41L4B | -3.85655 | 7.366344 | -5.77966 | 3.40E-08 | 2.99E-07 | 8.25691 |
| LY6H | -3.85891 | 8.626782 | -4.77326 | 3.83E-06 | 2.07E-05 | 3.706539 |
| TUBB4A | -3.86059 | 12.00162 | -4.68624 | 5.60E-06 | 2.91E-05 | 3.343535 |
| CTXN3 | -3.86366 | 2.155712 | -4.74413 | 4.35E-06 | 2.33E-05 | 3.584435 |
| GNAL | -3.8703 | 7.678754 | -7.92297 | 2.68E-13 | 8.58E-12 | 19.7075 |
| NETO1 | -3.87501 | 6.832426 | -4.2467 | 3.53E-05 | 0.000154 | 1.590521 |
| KIAA0319 | -3.87638 | 7.451225 | -7.28397 | 1.09E-11 | 2.28E-10 | 16.08639 |
| KCNH3 | -3.88597 | 8.437927 | -8.45901 | 1.08E-14 | 5.01E-13 | 22.85255 |
| ZCCHC12 | -3.88607 | 7.652383 | -6.10688 | 6.48E-09 | 6.82E-08 | 9.864596 |
| SLC7A10 | -3.88653 | 5.239139 | -4.24359 | 3.57E-05 | 0.000156 | 1.578582 |
| DYNC1I1 | -3.88668 | 8.759286 | -5.62589 | 7.27E-08 | 5.91E-07 | 7.521993 |
| OPRM1 | -3.88792 | 0.671677 | -10.6844 | 8.51E-21 | 1.84E-18 | 36.64873 |
| CSMD3 | -3.89163 | 6.00938 | -4.57132 | 9.18E-06 | 4.58E-05 | 2.872078 |
| VWA5B2 | -3.89222 | 6.899868 | -8.78739 | 1.45E-15 | 8.62E-14 | 24.82093 |
| GRM3 | -3.8924 | 8.638533 | -6.59177 | 5.02E-10 | 6.92E-09 | 12.34935 |
| GLRA2 | -3.89344 | 5.561586 | -4.36587 | 2.17E-05 | 9.93E-05 | 2.052244 |
| ANKRD30BL | -3.89364 | 2.009726 | -5.51303 | 1.26E-07 | 9.76E-07 | 6.991283 |
| ERC2 | -3.89558 | 7.899444 | -8.49936 | 8.45E-15 | 4.12E-13 | 23.09279 |
| DOC2B | -3.89602 | 7.509385 | -6.12972 | 5.76E-09 | 6.13E-08 | 9.978919 |
| CYP26B1 | -3.89622 | 7.1642 | -6.13414 | 5.63E-09 | 6.01E-08 | 10.00112 |
| HTR4 | -3.89906 | 3.285503 | -5.48016 | 1.48E-07 | 1.13E-06 | 6.838113 |
| NEFH | -3.9054 | 7.658087 | -6.19295 | 4.15E-09 | 4.60E-08 | 10.29698 |
| RSPO2 | -3.90679 | 6.367945 | -4.97168 | 1.58E-06 | 9.44E-06 | 4.553249 |
| TPH2 | -3.90683 | 1.414511 | -7.77617 | 6.36E-13 | 1.83E-11 | 18.86251 |
| TMEM233 | -3.91027 | 6.006178 | -5.89419 | 1.92E-08 | 1.78E-07 | 8.812942 |
| SLC26A8 | -3.91149 | 3.803741 | -9.28632 | 6.52E-17 | 5.39E-15 | 27.86395 |
| MAP3K9 | -3.91666 | 7.453965 | -6.79206 | 1.69E-10 | 2.64E-09 | 13.40892 |
| ADRA1B | -3.91761 | 6.395157 | -8.41633 | 1.40E-14 | 6.28E-13 | 22.59888 |
| SLIT3 | -3.91788 | 7.731334 | -6.53667 | 6.76E-10 | 9.01E-09 | 12.06119 |
| PALM2 | -3.92036 | 4.827359 | -6.10985 | 6.38E-09 | 6.72E-08 | 9.879439 |
| SLC24A2 | -3.9209 | 9.170313 | -5.94622 | 1.47E-08 | 1.42E-07 | 9.067944 |
| DNAJC5G | -3.9217 | 1.504946 | -7.12989 | 2.60E-11 | 4.95E-10 | 15.23671 |
| P2RX2 | -3.92239 | 2.361039 | -5.50166 | 1.33E-07 | 1.02E-06 | 6.938231 |
| TMEM88B | -3.92533 | 3.388018 | -5.35306 | 2.71E-07 | 1.95E-06 | 6.251973 |
| NMNAT2 | -3.92871 | 9.260881 | -6.30708 | 2.29E-09 | 2.70E-08 | 10.8762 |
| RASGRF2 | -3.93301 | 8.33984 | -10.5292 | 2.33E-20 | 4.45E-18 | 35.65945 |
| JAKMIP3 | -3.93716 | 6.563956 | -7.8753 | 3.55E-13 | 1.09E-11 | 19.43228 |
| RTP5 | -3.94211 | 6.240828 | -4.49931 | 1.25E-05 | 6.04E-05 | 2.581333 |
| ACTL6B | -3.94808 | 7.389553 | -4.26852 | 3.23E-05 | 0.000142 | 1.674292 |
| SLC27A2 | -3.94824 | 3.349258 | -6.55174 | 6.23E-10 | 8.38E-09 | 12.13986 |
| IRS4 | -3.94939 | 1.308662 | -5.96674 | 1.33E-08 | 1.29E-07 | 9.168936 |
| RAB11FIP4 | -3.95115 | 9.736205 | -7.58242 | 1.96E-12 | 5.01E-11 | 17.75886 |
| CRHR1 | -3.95238 | 5.585837 | -6.25038 | 3.08E-09 | 3.51E-08 | 10.5876 |
| PCP4L1 | -3.95928 | 6.366712 | -4.84528 | 2.79E-06 | 1.57E-05 | 4.010857 |
| WNK2 | -3.96027 | 8.457207 | -4.86484 | 2.56E-06 | 1.45E-05 | 4.09409 |
| STXBP5L | -3.96029 | 7.337257 | -5.51487 | 1.25E-07 | 9.68E-07 | 6.999868 |
| PAK3 | -3.96201 | 8.474747 | -5.81302 | 2.88E-08 | 2.58E-07 | 8.418135 |
| LHX6 | -3.96748 | 6.469392 | -7.94143 | 2.40E-13 | 7.77E-12 | 19.81426 |
| CDK5R2 | -3.96773 | 8.005358 | -5.43009 | 1.88E-07 | 1.40E-06 | 6.606035 |
| 4-Mar | -3.97234 | 6.257834 | -5.942 | 1.50E-08 | 1.44E-07 | 9.047208 |
| DNM1 | -3.97477 | 11.59474 | -7.64287 | 1.38E-12 | 3.67E-11 | 18.10173 |
| NPY1R | -3.97817 | 5.328995 | -5.33707 | 2.92E-07 | 2.09E-06 | 6.178913 |
| BHLHA9 | -3.98046 | 0.869755 | -9.12021 | 1.84E-16 | 1.37E-14 | 26.84435 |
| OR2L13 | -3.98208 | 3.243674 | -4.70125 | 5.25E-06 | 2.75E-05 | 3.405785 |
| MFSD4A | -3.991 | 8.471223 | -8.82287 | 1.17E-15 | 7.14E-14 | 25.0353 |
| CLVS2 | -3.99307 | 7.250288 | -4.9304 | 1.91E-06 | 1.12E-05 | 4.374954 |
| CPLX1 | -3.99415 | 8.73928 | -5.77401 | 3.50E-08 | 3.06E-07 | 8.229684 |
| BRINP1 | -3.99438 | 8.36202 | -5.4835 | 1.45E-07 | 1.11E-06 | 6.853634 |
| CARNS1 | -3.99627 | 8.956601 | -4.47286 | 1.39E-05 | 6.66E-05 | 2.475461 |
| DLG2 | -4.00026 | 9.542359 | -9.18244 | 1.25E-16 | 9.60E-15 | 27.2256 |
| HTR6 | -4.00488 | 2.302591 | -6.40686 | 1.35E-09 | 1.68E-08 | 11.38802 |
| ZFR2 | -4.00531 | 5.859781 | -4.84879 | 2.75E-06 | 1.55E-05 | 4.025771 |
| TENM2 | -4.00859 | 7.530169 | -4.88124 | 2.38E-06 | 1.36E-05 | 4.164064 |
| PSD | -4.01332 | 9.949379 | -6.88045 | 1.04E-10 | 1.71E-09 | 13.88232 |
| TAC1 | -4.01955 | 6.145837 | -3.42025 | 0.00078 | 0.002414 | -1.31651 |
| EPHB6 | -4.03159 | 8.891223 | -6.88766 | 1.00E-10 | 1.65E-09 | 13.92109 |
| ANKRD30B | -4.04071 | 1.366683 | -8.07827 | 1.07E-13 | 3.81E-12 | 20.60935 |
| PTPRN | -4.04202 | 10.20891 | -5.29239 | 3.61E-07 | 2.52E-06 | 5.97563 |
| ADAM11 | -4.04572 | 8.081829 | -8.36765 | 1.88E-14 | 8.17E-13 | 22.31027 |
| OPN4 | -4.04584 | 2.062953 | -6.74132 | 2.23E-10 | 3.38E-09 | 13.13874 |
| SMIM10L2B | -4.05142 | 7.374237 | -10.221 | 1.71E-19 | 2.60E-17 | 33.70285 |
| UNC13A | -4.06065 | 9.771199 | -5.89671 | 1.89E-08 | 1.76E-07 | 8.825265 |
| UNC5A | -4.06235 | 8.145129 | -5.75189 | 3.91E-08 | 3.39E-07 | 8.123204 |
| SIAH3 | -4.06339 | 4.345743 | -6.33246 | 2.00E-09 | 2.39E-08 | 11.0059 |
| CACNA2D3 | -4.06785 | 6.697583 | -7.22807 | 1.49E-11 | 3.04E-10 | 15.77701 |
| IPCEF1 | -4.06876 | 7.437078 | -9.00757 | 3.72E-16 | 2.59E-14 | 26.15654 |
| ANK3 | -4.07158 | 9.218849 | -8.71274 | 2.29E-15 | 1.31E-13 | 24.37091 |
| TMEM151A | -4.07532 | 8.326323 | -5.49325 | 1.39E-07 | 1.06E-06 | 6.899022 |
| C1orf115 | -4.0777 | 8.682596 | -8.37088 | 1.84E-14 | 8.05E-13 | 22.32942 |
| PPP1R16B | -4.0833 | 9.348979 | -6.29751 | 2.40E-09 | 2.82E-08 | 10.8274 |
| MCF2 | -4.08447 | 5.056663 | -5.78017 | 3.39E-08 | 2.99E-07 | 8.259372 |
| CDH22 | -4.0849 | 6.963951 | -5.0922 | 9.16E-07 | 5.77E-06 | 5.08011 |
| STX1B | -4.09162 | 9.933793 | -7.67037 | 1.18E-12 | 3.19E-11 | 18.25816 |
| MC4R | -4.09273 | 2.358319 | -5.29847 | 3.51E-07 | 2.45E-06 | 6.003219 |
| ADAD2 | -4.09555 | 4.936165 | -6.74506 | 2.19E-10 | 3.32E-09 | 13.15862 |
| KIAA0513 | -4.09875 | 10.52816 | -11.024 | 9.30E-22 | 2.73E-19 | 38.82447 |
| MATK | -4.09992 | 6.860402 | -9.11428 | 1.91E-16 | 1.42E-14 | 26.80808 |
| AIFM3 | -4.1003 | 7.410029 | -5.7724 | 3.53E-08 | 3.08E-07 | 8.221935 |
| SMPX | -4.1023 | 1.56861 | -6.07242 | 7.73E-09 | 7.94E-08 | 9.692578 |
| L1CAM | -4.10382 | 9.128884 | -4.88873 | 2.30E-06 | 1.32E-05 | 4.196105 |
| STXBP6 | -4.10417 | 7.728961 | -6.5849 | 5.21E-10 | 7.14E-09 | 12.31337 |
| KCNS1 | -4.10594 | 6.341365 | -5.096 | 9.00E-07 | 5.68E-06 | 5.096842 |
| SLC22A6 | -4.11174 | 3.275498 | -5.32148 | 3.15E-07 | 2.23E-06 | 6.107862 |
| CYP4X1 | -4.11748 | 5.523403 | -8.86977 | 8.73E-16 | 5.50E-14 | 25.31919 |
| KCNJ6 | -4.11795 | 6.614798 | -5.57816 | 9.18E-08 | 7.31E-07 | 7.29664 |
| LPAR3 | -4.11816 | 2.482165 | -5.91213 | 1.75E-08 | 1.65E-07 | 8.900706 |
| CHGB | -4.1197 | 9.18795 | -5.92353 | 1.65E-08 | 1.57E-07 | 8.956588 |
| NPY | -4.124 | 6.934278 | -4.70935 | 5.07E-06 | 2.67E-05 | 3.439447 |
| CNTN4 | -4.12582 | 6.094511 | -7.03296 | 4.47E-11 | 8.08E-10 | 14.70724 |
| SLC22A9 | -4.12755 | 0.790366 | -10.2954 | 1.06E-19 | 1.73E-17 | 34.174 |
| COL26A1 | -4.12807 | 6.027253 | -4.94678 | 1.77E-06 | 1.05E-05 | 4.44555 |
| FXYD7 | -4.12845 | 7.324849 | -4.83601 | 2.91E-06 | 1.63E-05 | 3.971486 |
| FGF13 | -4.13383 | 7.549082 | -5.20576 | 5.42E-07 | 3.60E-06 | 5.585028 |
| RGS7 | -4.13386 | 7.105313 | -6.43004 | 1.19E-09 | 1.50E-08 | 11.5076 |
| SGSM1 | -4.14071 | 7.464315 | -7.19764 | 1.77E-11 | 3.55E-10 | 15.60911 |
| FAM19A2 | -4.14218 | 6.761384 | -7.99223 | 1.78E-13 | 6.01E-12 | 20.10873 |
| RBM11 | -4.14318 | 4.117471 | -5.75583 | 3.83E-08 | 3.33E-07 | 8.142144 |
| TMEM151B | -4.14475 | 8.184961 | -6.04191 | 9.04E-09 | 9.16E-08 | 9.540757 |
| CNTNAP4 | -4.14807 | 7.745961 | -4.82161 | 3.10E-06 | 1.72E-05 | 3.910456 |
| SYP | -4.14959 | 11.20045 | -7.67648 | 1.14E-12 | 3.09E-11 | 18.29293 |
| IL12RB2 | -4.15805 | 3.519446 | -9.30886 | 5.66E-17 | 4.78E-15 | 28.00284 |
| GABRA2 | -4.16984 | 7.85135 | -5.23983 | 4.62E-07 | 3.13E-06 | 5.73811 |
| TCEAL6 | -4.17763 | 6.931895 | -5.54361 | 1.09E-07 | 8.50E-07 | 7.13433 |
| STMN2 | -4.18102 | 10.00824 | -4.2538 | 3.43E-05 | 0.00015 | 1.617715 |
| RALYL | -4.18614 | 6.886748 | -5.03384 | 1.20E-06 | 7.33E-06 | 4.823815 |
| SLC4A10 | -4.18771 | 8.335001 | -6.71911 | 2.52E-10 | 3.75E-09 | 13.02087 |
| HS3ST2 | -4.19027 | 7.207224 | -5.54663 | 1.07E-07 | 8.39E-07 | 7.148462 |
| NECAB2 | -4.19297 | 7.942407 | -6.94887 | 7.13E-11 | 1.22E-09 | 14.25114 |
| PRPH2 | -4.19307 | 4.529529 | -8.36095 | 1.96E-14 | 8.49E-13 | 22.2706 |
| REPS2 | -4.19589 | 8.435797 | -7.84897 | 4.14E-13 | 1.25E-11 | 19.28061 |
| SYT16 | -4.19617 | 7.703158 | -5.69616 | 5.15E-08 | 4.34E-07 | 7.856136 |
| PCDH11X | -4.20027 | 4.980067 | -3.9431 | 0.000117 | 0.000447 | 0.461532 |
| NGEF | -4.20398 | 9.236507 | -5.73605 | 4.23E-08 | 3.63E-07 | 8.047092 |
| NPY5R | -4.21378 | 2.903719 | -5.04054 | 1.16E-06 | 7.13E-06 | 4.85313 |
| SLC6A20 | -4.21801 | 3.50908 | -6.20365 | 3.92E-09 | 4.37E-08 | 10.35101 |
| ELAVL2 | -4.21845 | 7.257487 | -4.83424 | 2.93E-06 | 1.64E-05 | 3.963985 |
| LGI3 | -4.22481 | 9.107328 | -5.40233 | 2.14E-07 | 1.58E-06 | 6.478037 |
| PPFIA2 | -4.22979 | 7.688432 | -6.25354 | 3.03E-09 | 3.45E-08 | 10.60363 |
| PAK6 | -4.2315 | 3.997387 | -9.05141 | 2.83E-16 | 2.03E-14 | 26.42389 |
| CES4A | -4.23441 | 6.524275 | -8.67468 | 2.90E-15 | 1.59E-13 | 24.14204 |
| NPAS4 | -4.24095 | 3.363432 | -5.86467 | 2.22E-08 | 2.04E-07 | 8.668954 |
| GSG1L2 | -4.24318 | 0.690859 | -9.63167 | 7.38E-18 | 7.85E-16 | 30.00258 |
| GRIP1 | -4.25138 | 4.505799 | -5.93555 | 1.55E-08 | 1.48E-07 | 9.015544 |
| FBXL16 | -4.2515 | 10.61581 | -6.34704 | 1.85E-09 | 2.23E-08 | 11.08059 |
| GRM2 | -4.25257 | 6.061045 | -7.59964 | 1.78E-12 | 4.56E-11 | 17.85643 |
| GABRD | -4.25902 | 7.681913 | -6.27083 | 2.76E-09 | 3.19E-08 | 10.69152 |
| NAPB | -4.2609 | 10.45388 | -9.74353 | 3.63E-18 | 4.12E-16 | 30.70034 |
| PDE1A | -4.26862 | 7.894521 | -8.27282 | 3.33E-14 | 1.37E-12 | 21.75003 |
| SERPINI1 | -4.27093 | 9.270216 | -7.49521 | 3.25E-12 | 7.79E-11 | 17.26661 |
| SH3GL2 | -4.2798 | 9.055865 | -5.35503 | 2.68E-07 | 1.94E-06 | 6.261006 |
| CDH7 | -4.2816 | 4.162461 | -4.47852 | 1.36E-05 | 6.52E-05 | 2.498076 |
| RS1 | -4.28851 | 2.65287 | -7.02935 | 4.56E-11 | 8.20E-10 | 14.6876 |
| PLPPR3 | -4.29038 | 6.007519 | -4.54709 | 1.02E-05 | 5.04E-05 | 2.773847 |
| CCDC177 | -4.29995 | 4.282867 | -6.56917 | 5.67E-10 | 7.71E-09 | 12.231 |
| SLITRK1 | -4.30235 | 7.401537 | -4.87827 | 2.41E-06 | 1.38E-05 | 4.151408 |
| HPCA | -4.30337 | 8.820172 | -6.39818 | 1.41E-09 | 1.75E-08 | 11.3433 |
| AFF2 | -4.30495 | 5.445546 | -4.60764 | 7.86E-06 | 3.97E-05 | 3.020086 |
| HMGCLL1 | -4.30511 | 5.393378 | -5.66954 | 5.87E-08 | 4.87E-07 | 7.729239 |
| RAB3A | -4.30875 | 9.477784 | -8.14579 | 7.13E-14 | 2.67E-12 | 21.0039 |
| ABCC12 | -4.30884 | 2.904925 | -5.68799 | 5.36E-08 | 4.49E-07 | 7.817143 |
| CCDC85A | -4.31096 | 5.04119 | -7.43214 | 4.67E-12 | 1.08E-10 | 16.91243 |
| SNCA | -4.31242 | 9.318876 | -8.52879 | 7.07E-15 | 3.53E-13 | 23.26833 |
| SSTR4 | -4.31279 | 0.876869 | -9.03953 | 3.05E-16 | 2.16E-14 | 26.35135 |
| LOXHD1 | -4.31788 | 1.988786 | -8.33721 | 2.26E-14 | 9.65E-13 | 22.13014 |
| CACNG8 | -4.33157 | 8.555627 | -5.73656 | 4.21E-08 | 3.63E-07 | 8.049571 |
| SYT7 | -4.33823 | 9.340329 | -6.46483 | 9.92E-10 | 1.27E-08 | 11.6876 |
| PDE2A | -4.34488 | 9.413908 | -7.94779 | 2.31E-13 | 7.60E-12 | 19.85108 |
| CARMIL2 | -4.34812 | 6.324798 | -9.00367 | 3.81E-16 | 2.64E-14 | 26.13279 |
| CELF5 | -4.34822 | 8.45745 | -5.08786 | 9.34E-07 | 5.88E-06 | 5.06098 |
| RAB3B | -4.35184 | 7.271526 | -7.01295 | 5.00E-11 | 8.84E-10 | 14.5984 |
| RTBDN | -4.35339 | 4.697394 | -6.88982 | 9.89E-11 | 1.63E-09 | 13.9327 |
| GPR61 | -4.35511 | 4.967768 | -10.4344 | 4.30E-20 | 7.83E-18 | 35.05626 |
| NKAIN2 | -4.35703 | 7.304454 | -5.45239 | 1.69E-07 | 1.27E-06 | 6.709224 |
| SHANK2 | -4.35917 | 7.269188 | -5.47155 | 1.54E-07 | 1.17E-06 | 6.798104 |
| NEGR1 | -4.3617 | 8.3176 | -5.95954 | 1.38E-08 | 1.34E-07 | 9.133471 |
| KCNK1 | -4.36364 | 7.810418 | -5.77939 | 3.41E-08 | 3.00E-07 | 8.255625 |
| GJD2 | -4.36434 | 3.127782 | -5.23902 | 4.64E-07 | 3.14E-06 | 5.734462 |
| PYDC1 | -4.37057 | 1.799137 | -7.0735 | 3.56E-11 | 6.57E-10 | 14.92819 |
| ELFN2 | -4.3717 | 8.231117 | -4.72628 | 4.71E-06 | 2.50E-05 | 3.50993 |
| DOK6 | -4.37407 | 7.135848 | -6.49921 | 8.26E-10 | 1.08E-08 | 11.86604 |
| RGS7BP | -4.37621 | 6.768943 | -7.44704 | 4.29E-12 | 1.00E-10 | 16.99593 |
| RBP4 | -4.37825 | 5.958021 | -6.92942 | 7.95E-11 | 1.35E-09 | 14.14609 |
| PNMA3 | -4.3833 | 7.169401 | -6.56457 | 5.82E-10 | 7.88E-09 | 12.2069 |
| DLGAP3 | -4.38701 | 7.536631 | -7.21199 | 1.64E-11 | 3.31E-10 | 15.68821 |
| NEURL1 | -4.3887 | 7.641609 | -7.38246 | 6.21E-12 | 1.39E-10 | 16.63451 |
| SOWAHA | -4.3934 | 7.385077 | -7.69655 | 1.01E-12 | 2.78E-11 | 18.40733 |
| PLCH2 | -4.39567 | 7.300645 | -6.03495 | 9.37E-09 | 9.45E-08 | 9.506219 |
| HECW1 | -4.41897 | 6.819595 | -5.86578 | 2.21E-08 | 2.03E-07 | 8.67437 |
| CRHBP | -4.41939 | 5.701207 | -6.53637 | 6.77E-10 | 9.02E-09 | 12.05963 |
| CALB2 | -4.41981 | 6.592822 | -5.21313 | 5.23E-07 | 3.49E-06 | 5.618075 |
| AC009014.1 | -4.42181 | 2.809556 | -6.09423 | 6.91E-09 | 7.21E-08 | 9.801359 |
| JAKMIP1 | -4.42182 | 7.339734 | -6.47982 | 9.16E-10 | 1.19E-08 | 11.76533 |
| SAMD12 | -4.42701 | 6.834494 | -10.4633 | 3.57E-20 | 6.69E-18 | 35.2396 |
| FRRS1L | -4.42787 | 8.347403 | -5.94546 | 1.48E-08 | 1.42E-07 | 9.064243 |
| TACR3 | -4.4441 | 2.196923 | -7.38433 | 6.14E-12 | 1.37E-10 | 16.64494 |
| SPTB | -4.44968 | 6.552793 | -7.40952 | 5.32E-12 | 1.20E-10 | 16.78576 |
| MUM1L1 | -4.45145 | 5.113919 | -5.69132 | 5.27E-08 | 4.42E-07 | 7.833056 |
| PAK5 | -4.4524 | 5.858735 | -4.58847 | 8.53E-06 | 4.29E-05 | 2.941837 |
| VSTM2A | -4.45448 | 7.096041 | -4.44656 | 1.55E-05 | 7.35E-05 | 2.370678 |
| MPP7 | -4.45567 | 5.025974 | -7.9031 | 3.01E-13 | 9.51E-12 | 19.59271 |
| PNPLA5 | -4.45892 | 1.841733 | -7.25466 | 1.28E-11 | 2.66E-10 | 15.924 |
| NOS1 | -4.46483 | 5.987805 | -5.28679 | 3.71E-07 | 2.57E-06 | 5.950252 |
| C1QL2 | -4.46714 | 4.933187 | -4.75632 | 4.13E-06 | 2.22E-05 | 3.635486 |
| GALNT9 | -4.47037 | 7.223257 | -5.4624 | 1.61E-07 | 1.22E-06 | 6.755633 |
| GNG3 | -4.47056 | 8.142843 | -6.13372 | 5.64E-09 | 6.02E-08 | 9.998977 |
| NPM2 | -4.47147 | 5.974172 | -6.98253 | 5.92E-11 | 1.03E-09 | 14.43335 |
| KCNK9 | -4.47289 | 5.026701 | -5.72329 | 4.50E-08 | 3.84E-07 | 7.985943 |
| KRT31 | -4.48063 | 1.05506 | -8.59104 | 4.83E-15 | 2.52E-13 | 23.64038 |
| SLC6A5 | -4.49133 | 2.028768 | -6.14382 | 5.35E-09 | 5.75E-08 | 10.04966 |
| CHRNA4 | -4.49253 | 6.451272 | -4.83112 | 2.97E-06 | 1.66E-05 | 3.95075 |
| CDKL2 | -4.49947 | 5.079129 | -7.51052 | 2.98E-12 | 7.19E-11 | 17.35284 |
| RSPO3 | -4.49965 | 3.823454 | -5.79809 | 3.10E-08 | 2.75E-07 | 8.345903 |
| KCNJ9 | -4.50301 | 8.393981 | -6.49344 | 8.51E-10 | 1.11E-08 | 11.83608 |
| ARHGAP44 | -4.50506 | 7.401534 | -8.64524 | 3.47E-15 | 1.86E-13 | 23.96525 |
| KCNK12 | -4.51135 | 5.540217 | -7.01623 | 4.91E-11 | 8.71E-10 | 14.61626 |
| CABP1 | -4.5137 | 7.359934 | -6.55455 | 6.14E-10 | 8.26E-09 | 12.15454 |
| BSN | -4.51593 | 9.652536 | -7.91934 | 2.74E-13 | 8.74E-12 | 19.68651 |
| PRRG3 | -4.52163 | 5.65563 | -5.16347 | 6.59E-07 | 4.31E-06 | 5.396039 |
| CPNE9 | -4.52184 | 5.149787 | -10.7242 | 6.57E-21 | 1.53E-18 | 36.90339 |
| CLEC4G | -4.52231 | 2.910017 | -5.51254 | 1.26E-07 | 9.77E-07 | 6.98896 |
| CACNA1E | -4.52558 | 8.059081 | -7.27803 | 1.13E-11 | 2.36E-10 | 16.05345 |
| SYNGR3 | -4.5278 | 8.126585 | -6.84248 | 1.28E-10 | 2.05E-09 | 13.67851 |
| PRKCB | -4.53019 | 9.693633 | -8.39305 | 1.61E-14 | 7.12E-13 | 22.4608 |
| KCNH1 | -4.53046 | 5.420485 | -6.02683 | 9.77E-09 | 9.79E-08 | 9.46595 |
| NRXN3 | -4.53704 | 8.242743 | -7.14925 | 2.33E-11 | 4.50E-10 | 15.34293 |
| RIMS1 | -4.53885 | 7.051077 | -6.43488 | 1.16E-09 | 1.47E-08 | 11.53261 |
| SEC14L5 | -4.542 | 7.122896 | -5.91385 | 1.73E-08 | 1.64E-07 | 8.909138 |
| GABRB3 | -4.54202 | 8.340514 | -4.97753 | 1.54E-06 | 9.23E-06 | 4.578598 |
| EPHA8 | -4.54414 | 2.880517 | -8.53467 | 6.82E-15 | 3.41E-13 | 23.30342 |
| SNCG | -4.54638 | 7.749465 | -7.66758 | 1.20E-12 | 3.23E-11 | 18.24227 |
| XKR7 | -4.54819 | 4.872613 | -4.94233 | 1.81E-06 | 1.06E-05 | 4.426341 |
| SCN2B | -4.54819 | 8.134749 | -7.91793 | 2.76E-13 | 8.78E-12 | 19.67838 |
| CKMT1B | -4.55121 | 5.256416 | -5.18036 | 6.10E-07 | 4.02E-06 | 5.471367 |
| KNCN | -4.5554 | 2.290902 | -5.34092 | 2.87E-07 | 2.06E-06 | 6.196509 |
| SYT5 | -4.55586 | 8.192015 | -7.02546 | 4.66E-11 | 8.35E-10 | 14.66641 |
| PCSK2 | -4.55752 | 7.604776 | -4.35414 | 2.28E-05 | 0.000104 | 2.006323 |
| CA7 | -4.56629 | 3.842897 | -6.24873 | 3.10E-09 | 3.53E-08 | 10.57926 |
| PAX7 | -4.56741 | 2.592196 | -5.28887 | 3.67E-07 | 2.55E-06 | 5.959664 |
| CKMT1A | -4.57039 | 4.824558 | -5.63631 | 6.91E-08 | 5.64E-07 | 7.571372 |
| CBLN1 | -4.57394 | 4.346748 | -4.8008 | 3.40E-06 | 1.86E-05 | 3.822491 |
| HMP19 | -4.59125 | 9.683036 | -4.72957 | 4.64E-06 | 2.47E-05 | 3.523651 |
| VSTM2B | -4.60107 | 6.704668 | -4.83304 | 2.95E-06 | 1.65E-05 | 3.958879 |
| ARHGDIG | -4.61383 | 7.760195 | -6.03063 | 9.58E-09 | 9.63E-08 | 9.484763 |
| BEND4 | -4.62839 | 3.392984 | -5.10977 | 8.45E-07 | 5.37E-06 | 5.1577 |
| ADARB2 | -4.644 | 6.874168 | -6.56398 | 5.83E-10 | 7.90E-09 | 12.20385 |
| KCNG3 | -4.64453 | 2.342451 | -6.81554 | 1.49E-10 | 2.35E-09 | 13.53435 |
| NGB | -4.64965 | 4.336689 | -5.15336 | 6.91E-07 | 4.49E-06 | 5.351033 |
| DOC2A | -4.65012 | 7.67888 | -7.14628 | 2.37E-11 | 4.57E-10 | 15.32663 |
| INA | -4.66351 | 8.068573 | -4.66564 | 6.12E-06 | 3.16E-05 | 3.258359 |
| ATP1A3 | -4.66808 | 11.39878 | -5.53358 | 1.14E-07 | 8.90E-07 | 7.087337 |
| PTER | -4.6694 | 7.482397 | -8.97235 | 4.62E-16 | 3.10E-14 | 25.9421 |
| GPR52 | -4.67115 | 0.925606 | -11.1247 | 4.81E-22 | 1.55E-19 | 39.47172 |
| PEX5L | -4.67699 | 8.115895 | -6.64407 | 3.79E-10 | 5.40E-09 | 12.62426 |
| KCTD4 | -4.68021 | 5.642001 | -5.57033 | 9.54E-08 | 7.55E-07 | 7.259786 |
| PTPRT | -4.68204 | 7.775792 | -5.79909 | 3.09E-08 | 2.74E-07 | 8.350716 |
| SH2D5 | -4.68256 | 6.612127 | -8.52472 | 7.24E-15 | 3.60E-13 | 23.24401 |
| TPPP | -4.68316 | 10.43543 | -6.9321 | 7.83E-11 | 1.33E-09 | 14.16058 |
| CDH8 | -4.68368 | 6.514477 | -6.9754 | 6.16E-11 | 1.07E-09 | 14.3947 |
| FNDC9 | -4.68988 | 5.618206 | -5.89147 | 1.94E-08 | 1.81E-07 | 8.799695 |
| CPNE7 | -4.70136 | 5.879095 | -6.68283 | 3.07E-10 | 4.47E-09 | 12.82878 |
| NDST3 | -4.70176 | 4.21269 | -5.11473 | 8.25E-07 | 5.26E-06 | 5.179619 |
| PLCXD3 | -4.70178 | 6.2512 | -6.12572 | 5.88E-09 | 6.25E-08 | 9.958887 |
| SLC7A14 | -4.70363 | 7.905823 | -6.14085 | 5.43E-09 | 5.82E-08 | 10.03477 |
| CDR1 | -4.70966 | 3.177254 | -6.01733 | 1.03E-08 | 1.02E-07 | 9.418827 |
| KCNJ12 | -4.71126 | 4.476306 | -7.6319 | 1.47E-12 | 3.86E-11 | 18.03943 |
| TMEM215 | -4.71733 | 2.091344 | -6.95107 | 7.05E-11 | 1.21E-09 | 14.26303 |
| LRRC7 | -4.72848 | 6.874023 | -9.55904 | 1.17E-17 | 1.17E-15 | 29.55082 |
| CIDEA | -4.73082 | 1.566283 | -8.46411 | 1.05E-14 | 4.88E-13 | 22.88289 |
| FCER2 | -4.73222 | 2.880201 | -5.48504 | 1.44E-07 | 1.10E-06 | 6.860781 |
| ZNF831 | -4.73286 | 3.602503 | -7.80495 | 5.37E-13 | 1.57E-11 | 19.02758 |
| CRHR2 | -4.73729 | 3.303857 | -7.7797 | 6.23E-13 | 1.80E-11 | 18.88269 |
| RGS4 | -4.73986 | 8.971626 | -6.4827 | 9.02E-10 | 1.17E-08 | 11.78029 |
| SYCE1 | -4.7425 | 1.909809 | -7.88012 | 3.45E-13 | 1.07E-11 | 19.46008 |
| ACVR1C | -4.74274 | 5.346674 | -7.29102 | 1.05E-11 | 2.21E-10 | 16.12549 |
| KCNA1 | -4.75069 | 6.084044 | -5.94461 | 1.48E-08 | 1.42E-07 | 9.06003 |
| EPHA10 | -4.75914 | 5.785766 | -6.3304 | 2.02E-09 | 2.41E-08 | 10.9954 |
| SLITRK4 | -4.76291 | 5.914716 | -5.22022 | 5.06E-07 | 3.39E-06 | 5.649919 |
| SHANK1 | -4.76636 | 7.915609 | -6.51425 | 7.62E-10 | 1.01E-08 | 11.94434 |
| FREM3 | -4.77657 | 3.5569 | -4.246 | 3.54E-05 | 0.000154 | 1.587805 |
| GRIN3A | -4.77889 | 5.816529 | -9.87188 | 1.60E-18 | 2.08E-16 | 31.50368 |
| OCA2 | -4.78029 | 2.731172 | -6.19111 | 4.19E-09 | 4.63E-08 | 10.28766 |
| GRM7 | -4.78166 | 5.073971 | -5.9247 | 1.64E-08 | 1.56E-07 | 8.962335 |
| SCN3B | -4.78578 | 9.316832 | -7.42627 | 4.83E-12 | 1.11E-10 | 16.8795 |
| KCTD16 | -4.78675 | 6.802289 | -7.63284 | 1.47E-12 | 3.85E-11 | 18.04474 |
| AJAP1 | -4.79094 | 7.092179 | -7.22926 | 1.48E-11 | 3.03E-10 | 15.78356 |
| NRGN | -4.79124 | 10.63191 | -7.04372 | 4.21E-11 | 7.64E-10 | 14.76581 |
| LINC00282 | -4.7928 | 3.773647 | -7.49676 | 3.22E-12 | 7.74E-11 | 17.27537 |
| CHRNA2 | -4.79319 | 3.164561 | -6.24738 | 3.12E-09 | 3.56E-08 | 10.57239 |
| NYAP2 | -4.79661 | 2.347894 | -6.51322 | 7.66E-10 | 1.01E-08 | 11.93897 |
| KHDRBS2 | -4.79815 | 3.881252 | -6.77737 | 1.83E-10 | 2.84E-09 | 13.33055 |
| IQSEC3 | -4.79858 | 7.558258 | -6.49859 | 8.28E-10 | 1.08E-08 | 11.86282 |
| RIMS2 | -4.80121 | 6.639768 | -5.59188 | 8.59E-08 | 6.88E-07 | 7.361253 |
| SH3GL3 | -4.80937 | 7.042514 | -6.29489 | 2.44E-09 | 2.85E-08 | 10.81401 |
| CNTN3 | -4.81427 | 5.479877 | -5.32049 | 3.16E-07 | 2.24E-06 | 6.10336 |
| GREM2 | -4.82146 | 5.135276 | -6.72065 | 2.50E-10 | 3.72E-09 | 13.029 |
| GRM1 | -4.82187 | 5.743989 | -7.41773 | 5.07E-12 | 1.16E-10 | 16.83172 |
| HPCAL4 | -4.8243 | 9.456951 | -6.22115 | 3.58E-09 | 4.03E-08 | 10.43945 |
| RIMBP2 | -4.82748 | 7.163119 | -6.83898 | 1.31E-10 | 2.09E-09 | 13.65975 |
| PRMT8 | -4.83444 | 5.826951 | -7.17757 | 1.99E-11 | 3.90E-10 | 15.49861 |
| C11orf87 | -4.84021 | 6.85137 | -6.11944 | 6.07E-09 | 6.43E-08 | 9.927425 |
| TRPC5 | -4.8409 | 2.599714 | -7.31393 | 9.18E-12 | 1.96E-10 | 16.25271 |
| FEZF2 | -4.84584 | 4.802852 | -6.11031 | 6.36E-09 | 6.71E-08 | 9.881719 |
| SYN1 | -4.84673 | 9.988357 | -7.29876 | 1.00E-11 | 2.12E-10 | 16.16844 |
| VIP | -4.85079 | 4.238402 | -7.09855 | 3.10E-11 | 5.80E-10 | 15.06508 |
| 11-Mar | -4.85628 | 2.018914 | -6.63573 | 3.96E-10 | 5.61E-09 | 12.58034 |
| PTPRR | -4.85674 | 5.879714 | -7.12756 | 2.63E-11 | 5.01E-10 | 15.22393 |
| ISLR2 | -4.8601 | 6.834312 | -7.01755 | 4.87E-11 | 8.66E-10 | 14.6234 |
| EGR4 | -4.86047 | 3.379842 | -6.25514 | 3.00E-09 | 3.43E-08 | 10.61176 |
| FBXO40 | -4.8706 | 1.984079 | -9.16261 | 1.41E-16 | 1.06E-14 | 27.10405 |
| TMEM235 | -4.87648 | 5.362225 | -5.03032 | 1.21E-06 | 7.43E-06 | 4.808411 |
| GALNTL5 | -4.87705 | 2.329303 | -6.10437 | 6.56E-09 | 6.90E-08 | 9.852045 |
| LHFPL5 | -4.87724 | 1.919775 | -8.03246 | 1.40E-13 | 4.87E-12 | 20.34249 |
| LCN15 | -4.88558 | 2.237874 | -7.23492 | 1.44E-11 | 2.94E-10 | 15.81483 |
| CNTNAP2 | -4.88739 | 8.23729 | -6.53342 | 6.87E-10 | 9.14E-09 | 12.04422 |
| TRPV6 | -4.88752 | 4.358052 | -7.38784 | 6.02E-12 | 1.35E-10 | 16.66452 |
| OR14I1 | -4.88857 | 0.836995 | -9.86572 | 1.66E-18 | 2.14E-16 | 31.46507 |
| BHLHE22 | -4.8938 | 4.200558 | -5.51282 | 1.26E-07 | 9.76E-07 | 6.990285 |
| SPHKAP | -4.90541 | 5.717901 | -5.64364 | 6.67E-08 | 5.46E-07 | 7.606105 |
| CAMK1G | -4.90633 | 6.468854 | -6.04661 | 8.83E-09 | 8.97E-08 | 9.564096 |
| PTPN5 | -4.91291 | 7.731486 | -5.79989 | 3.08E-08 | 2.73E-07 | 8.354615 |
| GLT1D1 | -4.91807 | 6.196793 | -7.87331 | 3.59E-13 | 1.10E-11 | 19.4208 |
| ANO3 | -4.92145 | 5.718487 | -7.31959 | 8.89E-12 | 1.91E-10 | 16.28414 |
| KCNJ4 | -4.92249 | 6.960315 | -6.89152 | 9.80E-11 | 1.62E-09 | 13.94187 |
| HS6ST3 | -4.92743 | 6.968479 | -6.32276 | 2.10E-09 | 2.50E-08 | 10.95631 |
| SLC22A8 | -4.93626 | 0.775832 | -10.8002 | 4.00E-21 | 9.97E-19 | 37.3894 |
| LYPD8 | -4.93627 | 2.635936 | -6.53785 | 6.71E-10 | 8.96E-09 | 12.06734 |
| KLK5 | -4.93926 | 1.230331 | -8.12722 | 7.96E-14 | 2.94E-12 | 20.89525 |
| TUNAR | -4.96139 | 4.606535 | -5.45319 | 1.68E-07 | 1.27E-06 | 6.712899 |
| PHYHIP | -4.9659 | 9.650671 | -7.63605 | 1.44E-12 | 3.80E-11 | 18.06302 |
| SNCB | -4.97397 | 9.013826 | -5.89839 | 1.88E-08 | 1.75E-07 | 8.833495 |
| SV2C | -4.97879 | 5.786468 | -7.0041 | 5.25E-11 | 9.21E-10 | 14.55036 |
| SLC6A15 | -4.98185 | 6.324198 | -5.916 | 1.72E-08 | 1.62E-07 | 8.919681 |
| CAMKV | -4.98231 | 8.508596 | -6.06719 | 7.94E-09 | 8.13E-08 | 9.666503 |
| TMEM155 | -4.9837 | 5.943878 | -7.42643 | 4.83E-12 | 1.11E-10 | 16.88044 |
| SRRM4 | -4.98402 | 6.65489 | -5.39059 | 2.27E-07 | 1.66E-06 | 6.424019 |
| SYT10 | -4.98436 | 0.809808 | -11.3261 | 1.29E-22 | 4.60E-20 | 40.7686 |
| SLC35F3 | -5.00127 | 4.770447 | -6.91624 | 8.55E-11 | 1.43E-09 | 14.07501 |
| PABPC1L2A | -5.0034 | 2.483075 | -6.84635 | 1.26E-10 | 2.02E-09 | 13.69926 |
| AGBL1 | -5.0078 | 1.232244 | -9.65007 | 6.57E-18 | 7.10E-16 | 30.11724 |
| LRFN5 | -5.0163 | 5.591794 | -5.72112 | 4.55E-08 | 3.87E-07 | 7.975513 |
| LINGO2 | -5.02591 | 3.298094 | -5.94385 | 1.49E-08 | 1.43E-07 | 9.056303 |
| RELN | -5.03797 | 6.529287 | -6.70184 | 2.77E-10 | 4.06E-09 | 12.92934 |
| CRH | -5.04064 | 2.605161 | -6.18318 | 4.36E-09 | 4.80E-08 | 10.24771 |
| PABPC1L2B | -5.04296 | 2.550928 | -6.77955 | 1.81E-10 | 2.81E-09 | 13.34218 |
| GALNTL6 | -5.05142 | 4.053356 | -9.49523 | 1.75E-17 | 1.66E-15 | 29.15477 |
| ANKRD34C | -5.05588 | 1.974292 | -8.16255 | 6.45E-14 | 2.45E-12 | 21.10206 |
| CALN1 | -5.05924 | 7.25236 | -6.8201 | 1.45E-10 | 2.30E-09 | 13.5587 |
| CLEC2L | -5.06462 | 5.175864 | -5.81867 | 2.80E-08 | 2.51E-07 | 8.445497 |
| MTUS2 | -5.06593 | 4.936703 | -9.04762 | 2.90E-16 | 2.07E-14 | 26.40076 |
| PROKR2 | -5.06779 | 0.875848 | -10.6962 | 7.88E-21 | 1.76E-18 | 36.72408 |
| TNNT2 | -5.0737 | 3.468703 | -5.8565 | 2.32E-08 | 2.11E-07 | 8.62917 |
| HTR2A | -5.08678 | 6.165019 | -6.75032 | 2.13E-10 | 3.24E-09 | 13.18659 |
| FAM19A1 | -5.09469 | 5.243777 | -6.02963 | 9.63E-09 | 9.67E-08 | 9.479795 |
| SLC1A6 | -5.09693 | 4.751705 | -5.25583 | 4.29E-07 | 2.92E-06 | 5.810217 |
| PPP2R2C | -5.10204 | 8.95295 | -6.63806 | 3.91E-10 | 5.55E-09 | 12.59257 |
| PCLO | -5.10461 | 7.447177 | -6.72537 | 2.44E-10 | 3.63E-09 | 13.05407 |
| SLC17A6 | -5.10806 | 4.494333 | -4.87918 | 2.40E-06 | 1.37E-05 | 4.15529 |
| CDH12 | -5.1106 | 3.454189 | -6.60373 | 4.71E-10 | 6.53E-09 | 12.41212 |
| ICAM5 | -5.11122 | 7.414355 | -7.48574 | 3.43E-12 | 8.18E-11 | 17.21336 |
| MAP7D2 | -5.11217 | 6.907159 | -7.20102 | 1.74E-11 | 3.50E-10 | 15.62776 |
| RASGRF1 | -5.13737 | 7.523692 | -7.33838 | 7.98E-12 | 1.75E-10 | 16.3887 |
| PTGER3 | -5.14189 | 3.741285 | -7.53329 | 2.61E-12 | 6.42E-11 | 17.48118 |
| NPTX1 | -5.14576 | 9.583773 | -5.8025 | 3.04E-08 | 2.70E-07 | 8.367202 |
| DCAF12L2 | -5.15162 | 1.776276 | -6.68428 | 3.05E-10 | 4.43E-09 | 12.83643 |
| FSTL4 | -5.15622 | 5.394674 | -6.87145 | 1.09E-10 | 1.79E-09 | 13.83396 |
| SNAP91 | -5.16003 | 8.594669 | -5.68595 | 5.41E-08 | 4.54E-07 | 7.807415 |
| DACH2 | -5.1629 | 4.915201 | -6.49244 | 8.56E-10 | 1.11E-08 | 11.8309 |
| CARTPT | -5.16437 | 2.572457 | -5.65551 | 6.29E-08 | 5.18E-07 | 7.662514 |
| CBLN2 | -5.17515 | 5.150787 | -5.5516 | 1.04E-07 | 8.21E-07 | 7.171814 |
| GRIN2A | -5.17521 | 7.509091 | -6.29873 | 2.39E-09 | 2.80E-08 | 10.8336 |
| FAM153C | -5.17591 | 2.447694 | -7.00935 | 5.10E-11 | 8.98E-10 | 14.57885 |
| SDR16C5 | -5.18127 | 2.922581 | -6.12416 | 5.92E-09 | 6.29E-08 | 9.951092 |
| SYT4 | -5.18497 | 7.448547 | -5.65945 | 6.17E-08 | 5.10E-07 | 7.681244 |
| CACNG2 | -5.18634 | 3.862911 | -5.97849 | 1.25E-08 | 1.23E-07 | 9.226865 |
| LRFN2 | -5.19219 | 5.115423 | -6.32776 | 2.05E-09 | 2.44E-08 | 10.98187 |
| JPH3 | -5.19613 | 7.729067 | -5.6102 | 7.85E-08 | 6.34E-07 | 7.447743 |
| EPHA6 | -5.22031 | 2.621698 | -6.56496 | 5.80E-10 | 7.87E-09 | 12.20894 |
| HS3ST4 | -5.22243 | 5.645023 | -6.47362 | 9.46E-10 | 1.22E-08 | 11.7332 |
| ST8SIA3 | -5.22349 | 7.708092 | -5.23122 | 4.81E-07 | 3.24E-06 | 5.699361 |
| GRM4 | -5.2271 | 4.056752 | -7.40803 | 5.36E-12 | 1.21E-10 | 16.77741 |
| NELL1 | -5.22838 | 5.8565 | -6.09225 | 6.98E-09 | 7.27E-08 | 9.791457 |
| PPP4R4 | -5.22923 | 6.059456 | -8.55144 | 6.16E-15 | 3.10E-13 | 23.40361 |
| CUX2 | -5.26124 | 5.635102 | -6.0397 | 9.15E-09 | 9.24E-08 | 9.5298 |
| SLC12A5 | -5.26147 | 8.550089 | -8.66017 | 3.17E-15 | 1.72E-13 | 24.05484 |
| RTN4RL1 | -5.26838 | 5.731965 | -7.79983 | 5.53E-13 | 1.61E-11 | 18.9982 |
| NEUROD2 | -5.27028 | 6.12905 | -5.80762 | 2.96E-08 | 2.64E-07 | 8.391977 |
| KSR2 | -5.27456 | 5.436965 | -5.89728 | 1.89E-08 | 1.76E-07 | 8.828087 |
| FSTL5 | -5.27652 | 4.870767 | -6.81143 | 1.52E-10 | 2.40E-09 | 13.51238 |
| CELF4 | -5.27689 | 7.826363 | -6.30325 | 2.33E-09 | 2.74E-08 | 10.85668 |
| CHRM2 | -5.27891 | 3.309453 | -6.64453 | 3.78E-10 | 5.39E-09 | 12.62666 |
| HOOK1 | -5.2825 | 4.629531 | -8.09981 | 9.38E-14 | 3.43E-12 | 20.73505 |
| SLC7A4 | -5.28294 | 3.963929 | -8.60806 | 4.36E-15 | 2.28E-13 | 23.74229 |
| FAM153A | -5.30537 | 3.147305 | -6.51824 | 7.46E-10 | 9.85E-09 | 11.96507 |
| PHF24 | -5.31454 | 7.94323 | -7.05476 | 3.96E-11 | 7.23E-10 | 14.82596 |
| SNAP25 | -5.3203 | 11.31187 | -6.71087 | 2.64E-10 | 3.89E-09 | 12.97719 |
| OLFM3 | -5.32502 | 5.273911 | -6.17681 | 4.51E-09 | 4.94E-08 | 10.21557 |
| KCNT1 | -5.32608 | 6.116773 | -6.1498 | 5.19E-09 | 5.59E-08 | 10.07971 |
| OPRK1 | -5.33908 | 3.818279 | -5.94445 | 1.49E-08 | 1.42E-07 | 9.05925 |
| PDYN | -5.34242 | 6.80264 | -4.90651 | 2.12E-06 | 1.23E-05 | 4.272279 |
| KCNJ3 | -5.35227 | 6.139944 | -6.05778 | 8.34E-09 | 8.51E-08 | 9.619642 |
| SLC30A3 | -5.35317 | 6.570446 | -7.0033 | 5.27E-11 | 9.24E-10 | 14.54602 |
| CCK | -5.35355 | 7.125256 | -5.14208 | 7.28E-07 | 4.71E-06 | 5.300892 |
| EMX1 | -5.36561 | 4.640273 | -7.18631 | 1.89E-11 | 3.75E-10 | 15.54672 |
| TCERG1L | -5.36749 | 3.301382 | -6.40002 | 1.40E-09 | 1.73E-08 | 11.35277 |
| RAB3C | -5.36781 | 7.822162 | -6.11448 | 6.23E-09 | 6.59E-08 | 9.902584 |
| CDH18 | -5.37854 | 5.042881 | -5.62389 | 7.34E-08 | 5.96E-07 | 7.512501 |
| CHGA | -5.3878 | 7.897585 | -5.13954 | 7.36E-07 | 4.75E-06 | 5.289605 |
| GLRA3 | -5.38812 | 4.602063 | -4.79259 | 3.52E-06 | 1.93E-05 | 3.787857 |
| CNNM1 | -5.39147 | 5.386431 | -6.88313 | 1.03E-10 | 1.69E-09 | 13.89671 |
| KIAA1644 | -5.39959 | 7.030833 | -6.44547 | 1.10E-09 | 1.39E-08 | 11.58735 |
| AK5 | -5.40427 | 8.846006 | -7.21764 | 1.58E-11 | 3.22E-10 | 15.7194 |
| TESPA1 | -5.42739 | 5.753298 | -7.75297 | 7.28E-13 | 2.06E-11 | 18.72961 |
| CALHM1 | -5.43013 | 2.390668 | -9.63322 | 7.31E-18 | 7.81E-16 | 30.01227 |
| SYT13 | -5.43357 | 7.622547 | -5.85398 | 2.35E-08 | 2.14E-07 | 8.616908 |
| GABRG1 | -5.45071 | 6.101134 | -5.94399 | 1.49E-08 | 1.43E-07 | 9.056977 |
| ASIC2 | -5.45077 | 5.085091 | -6.41897 | 1.27E-09 | 1.58E-08 | 11.45045 |
| CCBE1 | -5.45411 | 3.323594 | -7.57706 | 2.03E-12 | 5.15E-11 | 17.72855 |
| CRYM | -5.46062 | 7.207154 | -5.61809 | 7.56E-08 | 6.12E-07 | 7.485079 |
| GABRA4 | -5.48984 | 5.926112 | -5.82744 | 2.68E-08 | 2.41E-07 | 8.488007 |
| KCNS2 | -5.49208 | 4.230125 | -6.74172 | 2.23E-10 | 3.37E-09 | 13.14085 |
| CACNA1I | -5.49343 | 5.69844 | -8.10615 | 9.03E-14 | 3.31E-12 | 20.77209 |
| AMER3 | -5.50726 | 4.947761 | -6.1705 | 4.66E-09 | 5.09E-08 | 10.1838 |
| EIF4E1B | -5.52718 | 2.069498 | -8.28763 | 3.04E-14 | 1.26E-12 | 21.83734 |
| FAM153B | -5.5276 | 3.648177 | -6.09647 | 6.83E-09 | 7.14E-08 | 9.812544 |
| SYN2 | -5.54088 | 9.465585 | -7.71099 | 9.30E-13 | 2.57E-11 | 18.48971 |
| UNC13C | -5.54148 | 5.196655 | -6.25479 | 3.01E-09 | 3.43E-08 | 10.60998 |
| TBR1 | -5.54618 | 5.915707 | -7.23905 | 1.40E-11 | 2.88E-10 | 15.83767 |
| GPR83 | -5.54847 | 4.614269 | -7.65972 | 1.25E-12 | 3.36E-11 | 18.19758 |
| WBSCR17 | -5.56181 | 7.620228 | -5.35827 | 2.64E-07 | 1.91E-06 | 6.275802 |
| HTR3B | -5.57647 | 1.38943 | -9.24013 | 8.71E-17 | 7.02E-15 | 27.57982 |
| GRM5 | -5.5774 | 4.459361 | -6.40944 | 1.33E-09 | 1.66E-08 | 11.40131 |
| WIF1 | -5.57795 | 4.746687 | -4.8916 | 2.27E-06 | 1.31E-05 | 4.208398 |
| HTR1E | -5.58753 | 1.793079 | -8.00328 | 1.67E-13 | 5.69E-12 | 20.17286 |
| SOWAHB | -5.59832 | 3.493093 | -9.07972 | 2.37E-16 | 1.72E-14 | 26.59677 |
| KCNA4 | -5.60834 | 3.559417 | -7.43808 | 4.51E-12 | 1.05E-10 | 16.94574 |
| GAD2 | -5.61199 | 6.518109 | -5.67209 | 5.80E-08 | 4.82E-07 | 7.741346 |
| GABRB2 | -5.61337 | 6.937254 | -6.52249 | 7.29E-10 | 9.64E-09 | 11.98725 |
| CALY | -5.6153 | 6.650297 | -6.04174 | 9.05E-09 | 9.16E-08 | 9.539925 |
| GPR22 | -5.63653 | 4.389125 | -6.95472 | 6.91E-11 | 1.19E-09 | 14.28277 |
| WNT10B | -5.64332 | 4.800219 | -7.67415 | 1.15E-12 | 3.13E-11 | 18.27968 |
| TMEM130 | -5.67201 | 8.621091 | -6.91007 | 8.84E-11 | 1.48E-09 | 14.04176 |
| KCNH5 | -5.67594 | 4.114863 | -6.34911 | 1.83E-09 | 2.21E-08 | 11.09121 |
| RBFOX3 | -5.70261 | 6.632622 | -6.70271 | 2.76E-10 | 4.04E-09 | 12.93395 |
| CPLX2 | -5.73902 | 9.57781 | -5.70257 | 4.99E-08 | 4.21E-07 | 7.886767 |
| HIPK4 | -5.75191 | 3.866613 | -9.4989 | 1.71E-17 | 1.63E-15 | 29.17755 |
| VSNL1 | -5.75351 | 9.08486 | -5.97211 | 1.29E-08 | 1.26E-07 | 9.195409 |
| RXFP1 | -5.76703 | 4.343764 | -7.68378 | 1.09E-12 | 2.98E-11 | 18.33453 |
| DRD1 | -5.7683 | 4.359588 | -7.56479 | 2.18E-12 | 5.46E-11 | 17.65911 |
| SLC32A1 | -5.77075 | 5.633738 | -5.83284 | 2.61E-08 | 2.36E-07 | 8.51419 |
| NEFL | -5.7894 | 8.935836 | -5.28293 | 3.77E-07 | 2.61E-06 | 5.932751 |
| CREG2 | -5.79855 | 7.653063 | -7.34068 | 7.88E-12 | 1.73E-10 | 16.40153 |
| GABRG3 | -5.81019 | 4.267296 | -6.8647 | 1.14E-10 | 1.84E-09 | 13.79769 |
| CHRM1 | -5.81273 | 6.35562 | -6.33791 | 1.94E-09 | 2.33E-08 | 11.03382 |
| RBFOX1 | -5.82235 | 7.075154 | -6.52653 | 7.13E-10 | 9.46E-09 | 12.00828 |
| GLP2R | -5.82822 | 3.952013 | -8.41774 | 1.39E-14 | 6.24E-13 | 22.60728 |
| SOHLH1 | -5.83881 | 3.060955 | -6.54753 | 6.37E-10 | 8.54E-09 | 12.11787 |
| CPNE6 | -5.85037 | 7.311118 | -6.20752 | 3.85E-09 | 4.30E-08 | 10.37055 |
| SYNPR | -5.8595 | 6.585851 | -5.91821 | 1.70E-08 | 1.60E-07 | 8.930513 |
| SYT1 | -5.86744 | 9.895021 | -6.82208 | 1.44E-10 | 2.28E-09 | 13.56932 |
| FAM163B | -5.87162 | 6.226778 | -5.79673 | 3.12E-08 | 2.77E-07 | 8.339337 |
| MYT1L | -5.88548 | 6.899663 | -5.98001 | 1.24E-08 | 1.22E-07 | 9.234355 |
| HTR1A | -5.89689 | 1.614756 | -8.45495 | 1.11E-14 | 5.11E-13 | 22.82841 |
| CAMK2A | -5.89724 | 9.760171 | -6.94683 | 7.22E-11 | 1.23E-09 | 14.24011 |
| SLC6A17 | -5.8978 | 8.03531 | -7.18383 | 1.92E-11 | 3.79E-10 | 15.53306 |
| HRH3 | -5.9006 | 4.884437 | -6.45018 | 1.07E-09 | 1.37E-08 | 11.61172 |
| STYK1 | -5.90607 | 3.575486 | -8.07374 | 1.10E-13 | 3.90E-12 | 20.58291 |
| CCKBR | -5.91456 | 4.69928 | -7.10166 | 3.04E-11 | 5.71E-10 | 15.0821 |
| GDA | -5.91458 | 7.48034 | -6.61095 | 4.53E-10 | 6.31E-09 | 12.45004 |
| CNGB1 | -5.91572 | 2.72097 | -8.01623 | 1.54E-13 | 5.33E-12 | 20.2481 |
| GJB6 | -5.9212 | 4.830904 | -5.10862 | 8.49E-07 | 5.40E-06 | 5.1526 |
| ATP2B3 | -5.92802 | 6.142557 | -7.74437 | 7.66E-13 | 2.16E-11 | 18.68045 |
| KCNB2 | -5.93634 | 2.447862 | -7.43399 | 4.62E-12 | 1.07E-10 | 16.92281 |
| GABRG2 | -5.93975 | 6.697372 | -5.3425 | 2.85E-07 | 2.04E-06 | 6.203717 |
| HTR5A | -5.94649 | 3.527462 | -6.71384 | 2.59E-10 | 3.84E-09 | 12.99289 |
| SSTR3 | -5.949 | 3.632184 | -8.3912 | 1.63E-14 | 7.18E-13 | 22.44984 |
| DLGAP2 | -5.96823 | 4.951306 | -7.35464 | 7.28E-12 | 1.61E-10 | 16.4793 |
| FRMPD4 | -5.97589 | 5.288649 | -7.10416 | 3.00E-11 | 5.63E-10 | 15.09577 |
| C4orf50 | -5.97772 | 3.230924 | -8.16018 | 6.54E-14 | 2.48E-12 | 21.08815 |
| SULT4A1 | -5.98615 | 7.648696 | -6.7014 | 2.78E-10 | 4.06E-09 | 12.927 |
| GPR6 | -6.00134 | 1.524802 | -8.49297 | 8.79E-15 | 4.21E-13 | 23.05473 |
| KIF12 | -6.00517 | 1.790237 | -9.52935 | 1.41E-17 | 1.38E-15 | 29.36647 |
| SLC8A2 | -6.00899 | 7.355397 | -7.35055 | 7.45E-12 | 1.64E-10 | 16.45649 |
| WSCD2 | -6.0126 | 5.200225 | -6.47968 | 9.16E-10 | 1.19E-08 | 11.76464 |
| ATP8A2 | -6.01905 | 6.711725 | -7.52059 | 2.81E-12 | 6.84E-11 | 17.40957 |
| DDN | -6.01909 | 7.626766 | -7.94733 | 2.32E-13 | 7.60E-12 | 19.84845 |
| FADS6 | -6.03945 | 2.255119 | -9.07421 | 2.46E-16 | 1.77E-14 | 26.56308 |
| DRD5 | -6.12588 | 1.865708 | -9.08417 | 2.31E-16 | 1.68E-14 | 26.62393 |
| GPR26 | -6.12719 | 2.872305 | -6.36061 | 1.72E-09 | 2.09E-08 | 11.15017 |
| CACNA1B | -6.13209 | 5.954566 | -6.80433 | 1.58E-10 | 2.48E-09 | 13.47442 |
| LRTM2 | -6.13785 | 4.793077 | -6.16506 | 4.79E-09 | 5.21E-08 | 10.15643 |
| SSTR1 | -6.18577 | 4.683762 | -6.77633 | 1.84E-10 | 2.85E-09 | 13.32502 |
| CHD5 | -6.19152 | 7.591366 | -7.51919 | 2.83E-12 | 6.89E-11 | 17.40168 |
| RASAL1 | -6.19865 | 6.188407 | -7.57555 | 2.04E-12 | 5.19E-11 | 17.72 |
| KCNC2 | -6.20176 | 5.462063 | -5.74956 | 3.95E-08 | 3.42E-07 | 8.111969 |
| MPPED1 | -6.21415 | 5.898502 | -7.2865 | 1.07E-11 | 2.26E-10 | 16.10039 |
| SVOP | -6.22911 | 6.60913 | -6.7509 | 2.12E-10 | 3.23E-09 | 13.18965 |
| HCN1 | -6.23821 | 4.759386 | -6.4364 | 1.15E-09 | 1.46E-08 | 11.54045 |
| KRT222 | -6.25801 | 3.079568 | -7.41249 | 5.23E-12 | 1.19E-10 | 16.80238 |
| MAS1 | -6.26054 | 2.492066 | -8.37367 | 1.81E-14 | 7.93E-13 | 22.34596 |
| RYR2 | -6.27431 | 6.00505 | -8.56606 | 5.63E-15 | 2.86E-13 | 23.49095 |
| PACSIN1 | -6.32445 | 8.160975 | -6.91619 | 8.55E-11 | 1.43E-09 | 14.07473 |
| SERTM1 | -6.32792 | 4.362514 | -6.90134 | 9.28E-11 | 1.54E-09 | 13.99469 |
| SST | -6.35049 | 5.610178 | -6.93158 | 7.85E-11 | 1.33E-09 | 14.15776 |
| GRIN2B | -6.35428 | 6.853596 | -7.73557 | 8.06E-13 | 2.26E-11 | 18.6301 |
| CDH9 | -6.36967 | 3.243566 | -7.80922 | 5.24E-13 | 1.53E-11 | 19.05205 |
| MAL2 | -6.41471 | 5.425343 | -6.22436 | 3.52E-09 | 3.97E-08 | 10.45574 |
| NEFM | -6.41538 | 7.000232 | -6.26514 | 2.85E-09 | 3.27E-08 | 10.66259 |
| HTR2C | -6.43635 | 2.785145 | -8.29881 | 2.85E-14 | 1.19E-12 | 21.90334 |
| GABRA1 | -6.44218 | 6.572278 | -5.6136 | 7.72E-08 | 6.24E-07 | 7.463823 |
| KLHL1 | -6.47201 | 2.056509 | -8.63761 | 3.64E-15 | 1.94E-13 | 23.91948 |
| TRHDE | -6.49473 | 3.921656 | -7.55176 | 2.35E-12 | 5.85E-11 | 17.58551 |
| SV2B | -6.57488 | 7.609876 | -6.99341 | 5.57E-11 | 9.73E-10 | 14.49235 |
| SLC17A7 | -6.64764 | 9.405189 | -6.07543 | 7.61E-09 | 7.85E-08 | 9.707533 |
| OPALIN | -6.65781 | 6.193565 | -5.52664 | 1.18E-07 | 9.19E-07 | 7.054886 |
| TMEM132D | -6.6608 | 4.396293 | -6.90516 | 9.09E-11 | 1.51E-09 | 14.0153 |
| NWD2 | -6.66654 | 3.223973 | -8.57911 | 5.20E-15 | 2.69E-13 | 23.569 |
| SLC6A7 | -6.66958 | 4.587999 | -7.25091 | 1.31E-11 | 2.71E-10 | 15.90325 |
| PRKCG | -6.68009 | 6.342577 | -8.55629 | 5.98E-15 | 3.02E-13 | 23.43259 |
| C1QL3 | -6.69673 | 4.64176 | -7.76048 | 6.97E-13 | 1.98E-11 | 18.77264 |
| GABRA5 | -6.70059 | 5.945129 | -6.61103 | 4.53E-10 | 6.31E-09 | 12.45044 |
| MCHR2 | -6.70106 | 1.909579 | -8.68753 | 2.68E-15 | 1.50E-13 | 24.21922 |
| PNMA5 | -6.71312 | 3.596093 | -9.29703 | 6.10E-17 | 5.08E-15 | 27.92995 |
| CACNG3 | -6.85052 | 5.08118 | -6.22379 | 3.53E-09 | 3.98E-08 | 10.45283 |
| KCNV1 | -6.93149 | 3.999546 | -6.34166 | 1.90E-09 | 2.28E-08 | 11.05304 |
| GRIN1 | -7.02702 | 7.755637 | -6.53442 | 6.84E-10 | 9.10E-09 | 12.0494 |
| KLK7 | -7.26384 | 2.626025 | -7.79212 | 5.79E-13 | 1.68E-11 | 18.95395 |
| NEUROD6 | -7.33125 | 2.965155 | -7.98841 | 1.82E-13 | 6.12E-12 | 20.08651 |
| PNMA6F | -7.36734 | 2.313928 | -9.16563 | 1.39E-16 | 1.05E-14 | 27.12251 |
