# Supplementary material for: Construction of Potential Glioblastoma Multiforme-Related miRNA-mRNA Regulatory Network
Source: Front Mol Neurosci. 2019 Mar 26;12:66. doi: 10.3389/fnmol.2019.00066 (PMC6444190; doi:10.3389/fnmol.2019.00066)
Supplement: Supplementary file 3 [file Table_3.DOCX]

Table S3. Candidate target genes for DE-miRNAs.

| Candidate target genes for upregulated DE-miRNAs | Candidate target genes for downregulated DE-miRNAs |
| --- | --- |
| KLHL14 | FSTL1 |
| SEMA4D | CD44 |
| PHYHIP | MMP2 |
| CORO2A | CXCR4 |
| CPEB3 | RPS18 |
| FAXC | RDH10 |
| THRB | TRIB3 |
| CYP1A1 | RUNX3 |
| LONRF2 | A2M |
| SH3PXD2A | COL1A1 |
| TYRP1 | SLFN12 |
| RAB15 | ORC1 |
| NEGR1 | EN1 |
| FGF12 | DPYD |
| KRT33B | GNG12 |
| PDXP | TFAP2B |
| RHO | CMTM3 |
| GRAMD1B | RAB38 |
| SERPINI1 | CD151 |
| NBEA | HOXB5 |
| IPCEF1 | B2M |
| SLC24A4 | GLIPR2 |
| TSPYL1 | TYMS |
| SNCG | MELK |
| SHANK2 | VEGFA |
| KIF3A | SGO1 |
| ANKRD33B | MYC |
| KLC2 | OIP5 |
| SLC27A2 | E2F7 |
| TPPP | CA12 |
| MKX | CBX2 |
| JPH1 | CKS2 |
| ANKRD18A | UCP2 |
| GLP2R | DEPDC1 |
| AAK1 | ASPN |
| MAST3 | YBX1 |
| DUSP8 | ANXA2 |
| MEF2C | F2RL2 |
| CBFA2T3 | LMNB1 |
| CRY2 | BRCA2 |
| CACNA1E | CLIC4 |
| CAMSAP3 | ALOX5AP |
| MRAP2 | EGFR |
| SELE | HOXC4 |
| SLC25A22 | RPS3A |
| ITPR1 | LGALS3 |
| RUNDC3B | RPL18A |
| SCAMP5 | DDIAS |
| MIGA1 | SCIMP |
| FBXL2 | BARHL1 |
| CSRNP3 | CHEK2 |
| PRKCE | RAB32 |
| EDIL3 | BARX1 |
| FUT1 | EN2 |
| HIVEP2 | CDK2 |
| SNPH | PRPH |
| INA | ODC1 |
| SLC5A5 | E2F2 |
| PNMA8B | CXCL10 |
| TBR1 | SLC7A7 |
| MYCBP2 | CKAP2L |
| CDK5R1 | VCAN |
| SLC12A5 | GJC1 |
| NOS1AP | AJUBA |
| SYNPR | MAGEA12 |
| GLB1L3 | FNDC3B |
| DOCK5 | IGFBP3 |
| SYNJ1 | SERPINA3 |
| ZNF98 | TRIM5 |
| MOAP1 | NEK2 |
| TNFSF9 | ABCA13 |
| PPFIA4 | CDC25A |
| MCF2L2 | XRCC2 |
| DLX1 | BCL2L12 |
| SFRP1 | NID1 |
| GNAL | ID3 |
| TPRG1L | SOX4 |
| RAB40B | MSN |
| PCDH7 | CDK15 |
| KL | PTPRZ1 |
| ATP1B4 | MAML2 |
| CPEB1 | PLP2 |
| ACOT7 | TAGLN2 |
| FAM126B | NEDD4 |
| CBX6 | FBLIM1 |
| PPL | EZH2 |
| FMN1 | CHEK1 |
| YWHAH | C8orf4 |
| RUNX1T1 | NCF2 |
| RS1 | IGFBP7 |
| RHOV | TCF12 |
| BCL11B | HAL |
| EEF1A2 | TBXA2R |
| INPP5F | KIFC1 |
| PPP3CB | RUNX1 |
| TRPC5 | RASEF |
| PSD | SLC43A3 |
| TENM3 | COL6A2 |
| DLGAP3 | FKBP10 |
| CAMK4 | VIM |
| FBXW7 | ABCC3 |
| TDRD6 | HOXA10 |
| GABRB3 | SAMD9L |
| NAV3 | HLA-A |
| STXBP1 | GBP2 |
| PREPL | LRRC15 |
| BSPRY | CDCA7 |
| LOXHD1 | CAMP |
| OLFM3 | SLC28A1 |
| TMEM56 | TNFRSF12A |
| ATP1A3 | PRR11 |
| CDH13 | POPDC2 |
| BASP1 | TLR3 |
| CHST1 | HMGB2 |
| CEP170B | PTBP1 |
| RAB27B | PDIA5 |
| RIMS3 | MAGED4B |
| ADRA1B | PLA2G5 |
| XKR7 | HOXC9 |
| STXBP5 | IGFBP1 |
| MYO5A | TUBB6 |
| GABARAPL1 | CCDC80 |
| PLEKHA1 | FAM109B |
| MAP3K10 | TOP2A |
| OBSCN | NR5A2 |
| PTPRT | EFEMP2 |
| RGR | CAV1 |
| CSMD1 | HELLS |
| ANKRD24 | RTP4 |
| CDKL2 | SOCS2 |
| FADS6 | FAM83D |
| ELOVL4 | TSPAN6 |
| CASKIN1 | CSRP2 |
| TEF | PLEKHA4 |
| DOCK9 | STC2 |
| PTPRD | CTHRC1 |
| NSF | UHRF1 |
| GPR27 | PDIA4 |
| RASGRP1 | AR |
| ELAVL4 | CLCF1 |
| RAB3B | IL7 |
| RYR2 | DPYSL3 |
| CYP26B1 | MNX1 |
| KCNC4 | ADAM12 |
| RAB6C | GDF15 |
| HHIP | LBX1 |
| NPTX1 | GINS1 |
| PTPN3 | SMO |
| TLL1 | PALLD |
|  | RHOC |
|  | BIRC5 |
|  | SERPING1 |
|  | HLA-DRA |
|  | POTEG |
|  | AFAP1L1 |
|  | CD300A |
|  | CD99 |
|  | AURKA |
|  | FN1 |
|  | LOXL1 |
|  | PRRX1 |
|  | AURKB |
|  | IFI16 |
|  | MAP3K7CL |
|  | HAND2 |
|  | WNT5A |
|  | CDK4 |
|  | FMOD |
|  | PRDM13 |
|  | ZWINT |
|  | HNRNPCL1 |
|  | RCC1 |
|  | NNMT |
|  | ZNF90 |
|  | IGFBP5 |
|  | NME4 |
|  | WEE1 |
|  | MMP11 |
|  | BEST3 |
|  | CNN2 |
|  | HMOX1 |
|  | NAMPT |
|  | PCLAF |
|  | PPP1R14B |
|  | MS4A4A |
|  | ADAMTS15 |
|  | MCM7 |
|  | C1QTNF6 |
|  | GBP1 |
|  | DTYMK |
|  | BMP1 |
|  | CYTIP |
|  | S100A2 |
|  | EIF4EBP1 |
|  | NID2 |
|  | MACC1 |
|  | TFAP2A |
|  | TFPI |
|  | RAD51AP1 |
|  | ESCO2 |
|  | COL4A1 |
|  | MKI67 |
|  | GLI2 |
|  | TP53 |
|  | LYZ |
|  | FKBP1C |
|  | CDKN2C |
|  | PPIC |
|  | NSUN7 |
|  | HOXC8 |
|  | TGFB1I1 |
|  | NXT2 |
|  | MMP19 |
|  | VNN2 |
|  | PARP9 |
|  | SOX2 |
|  | HTRA4 |
|  | SPC24 |
|  | VAMP8 |
|  | SQOR |
|  | CD1D |
|  | FAM129A |
|  | GPX7 |
|  | TMEM45A |
|  | FBN2 |
|  | HIST1H3B |
|  | ABCA1 |
|  | MUC1 |
|  | TBX2 |
|  | HOXB3 |
|  | SIGLEC14 |
|  | JAG1 |
|  | CNN3 |
|  | AKNAD1 |
|  | SLC1A5 |
|  | HOXA3 |
|  | DNALI1 |
|  | EMP1 |
|  | CDCA7L |
|  | RPS2 |
|  | ADGRL4 |
|  | ZYX |
|  | PPP1R3B |
|  | PCNA |
|  | PTK7 |
|  | KIF2C |
|  | H3F3C |
|  | SLPI |
|  | CHST14 |
|  | DRAM1 |
|  | HTRA3 |
|  | LAMC1 |
|  | S100A11 |
|  | NXT1 |
|  | ANXA5 |
|  | HMGA2 |
|  | SH2D4A |
|  | CPA6 |
|  | MFAP4 |
|  | IKBIP |
|  | ASPM |
|  | SERPINH1 |
|  | RACK1 |
|  | CDCA3 |
|  | SP100 |
|  | LOXL2 |
|  | ADPRH |
|  | CALU |
|  | MDK |
|  | RBM47 |
|  | ANPEP |
|  | KIF15 |
|  | RIPOR3 |
|  | HJURP |
|  | SERPINE1 |
|  | RAD51 |
|  | REEP4 |
|  | PDLIM3 |
|  | CDCP1 |
|  | YBX3 |
|  | KIF20A |
|  | SOX11 |
|  | ITGB3 |
|  | LAMA4 |
|  | IGFBP4 |
|  | HOXC6 |
|  | SNAI2 |
|  | DLEU1 |
|  | HSPG2 |
|  | ATP6V0E1 |
|  | FLNA |
|  | DENND2D |
|  | ANO6 |
|  | SPOCD1 |
|  | MMP9 |
